# Diffusion tensor imaging‐based machine learning for IDH wild‐type glioblastoma stratification to reveal the biological underpinning of radiomic features

**DOI:** 10.1111/cns.14263

**Published:** 2023-05-24

**Authors:** Zilong Wang, Fangzhan Guan, Wenchao Duan, Yu Guo, Dongling Pei, Yuning Qiu, Minkai Wang, Aoqi Xing, Zhongyi Liu, Bin Yu, Hongwei Zheng, Xianzhi Liu, Dongming Yan, Yuchen Ji, Jingliang Cheng, Jing Yan, Zhenyu Zhang

**Affiliations:** ^1^ Department of Neurosurgery The First Affiliated Hospital of Zhengzhou University Zhengzhou China; ^2^ Department of MRI The First Affiliated Hospital of Zhengzhou University Zhengzhou China

**Keywords:** biological pathway, diffusion tensor imaging, glioblastoma, machine learning, prognosis

## Abstract

**Introduction:**

This study addresses the lack of systematic investigation into the prognostic value of hand‐crafted radiomic features derived from diffusion tensor imaging (DTI) in isocitrate dehydrogenase (IDH) wild‐type glioblastoma (GBM), as well as the limited understanding of the biological interpretation of individual DTI radiomic features and metrics.

**Aims:**

To develop and validate a DTI‐based radiomic model for predicting prognosis in patients with IDH wild‐type GBM and reveal the biological underpinning of individual DTI radiomic features and metrics.

**Results:**

The DTI‐based radiomic signature was an independent prognostic factor (*p* < 0.001). Incorporating the radiomic signature into a clinical model resulted in a radiomic‐clinical nomogram that predicted survival better than either the radiomic model or clinical model alone, with a better calibration and classification accuracy. Four categories of pathways (synapse, proliferation, DNA damage response, and complex cellular functions) were significantly correlated with the DTI‐based radiomic features and DTI metrics.

**Conclusion:**

The prognostic radiomic features derived from DTI are driven by distinct pathways involved in synapse, proliferation, DNA damage response, and complex cellular functions of GBM.

## Graphical abstract

The DTI‐based hand‐crafted radiomic signature was an independent prognostic factor. Moreover, four categories of pathways (synapse, proliferation, DNA damage response and complex cellular functions) were significantly correlated with them.

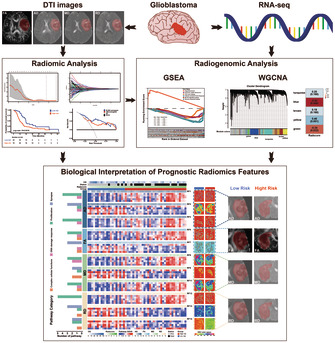

## 1 BACKGROUND

Glioblastoma (GBM) is the most common malignant tumor occurring in the brain, with a median survival of 12–15 months despite treatment comprising surgery followed by concurrent radiochemotherapy and temozolomide chemotherapy.^1^ Previous studies have demonstrated that isocitrate dehydrogenase (IDH) mutations have a considerable impact on the prognosis of patients with GBM,^2^, ^3^ and the role of IDH mutations has been reinforced in classifying IDH wild‐type GBM in the 2021 World Health Organization classification of tumors of the central nervous system.^4^ Nonetheless, evidence has shown that survival outcomes and treatment responses are heterogeneous among patients with IDH wild‐type GBM.^5^, ^6^, ^7^ Thus, preoperative prognostic markers that stratify patients with IDH wild‐type GBM may be useful for improving disease management and guiding individualized therapy.

Radiomics has provided a noninvasive method for characterizing tumors by extracting quantitative features from imaging data. It is hypothesized that medical images reflect the underlying pathophysiological characteristics of cancer, and radiomic features may, therefore function as a surrogate biomarker of the tumor.^8^ Several studies have shown that radiomic features have incremental prognostic value over clinicopathological factors in gliomas.^9^, ^10^, ^11^ Recently, radiogenomic studies have demonstrated that prognostic radiomic features derived from conventional magnetic resonance (MR) sequences are correlated with specific biological pathways.^10^, ^12^, ^13^ Notably, these studies used either gene set enrichment analysis (GSEA) or weighted gene co‐expression network analysis (WGCNA) to identify biological pathways in radiogenomic analysis.^12^, ^13^ In RNA sequencing (RNA‐seq) data analysis, GSEA focused on differentially expressed genes, whereas WGCNA focused on interactions between the genes.^14^, ^15^ Therefore, combining GSEA and WGCNA in radiogenomic analysis may reinforce the reproducibility of the biological underpinning underlying radiomic phenotypes.

Diffusion tensor imaging (DTI) is an advanced MR sequence that detects microstructural tissue changes by assessing water diffusion in vivo.^16^, ^17^ Over the past few years, DTI has been increasingly used to study brain tumors.^18^, ^19^ It contains four main metrics: mean diffusivity (MD), fractional anisotropy (FA), axial diffusivity (AD), and radial diffusivity (RD).^20^ MD is the average of the tensor's eigenvalues, which is sensitive to the initial cellular swelling (cytotoxic edema) which restricts diffusion. This characteristic makes it useful in identifying early strokes.^21^ As the most widely used anisotropy measure, FA measures the fraction of the diffusion that is anisotropic, which is often considered a measure of “white matter integrity” though changes in FA may be caused by many factors.^22^ AD, also called the parallel diffusivity, is equal to the largest eigenvalue. The perpendicular diffusivity measure, also called RD, is equal to the average of the two smaller eigenvalues. These measures are interpreted as diffusivity parallel to and perpendicular to a white matter fiber tract, so they make the most sense in regions of coherently oriented axons with no fiber crossings. These metrics have been previously demonstrated to be capable of predicting survival outcomes in GBM.^23^, ^24^ However, these studies leveraged semiquantitative DTI metrics to perform histogram analysis, and there is a lack of studies that have systematically investigated the prognostic values of hand‐crafted radiomic features derived from DTI in GBM. In addition, the biological interpretation of individual DTI radiomic feature and metrics remains elusive, posing a barrier to the clinical application of DTI‐based radiomics.

This radiogenomic study aimed to (a) develop and validate a DTI‐based radiomic model for predicting the overall survival (OS) of patients with IDH wild‐type GBM and (b) investigate the biological underpinning of the prognostic radiomic features by identifying underlying biological pathways using paired DTI and RNA sequencing data.

## 2 MATERIALS AND METHODS

### 2.1 Study design

**FIGURE 1:**
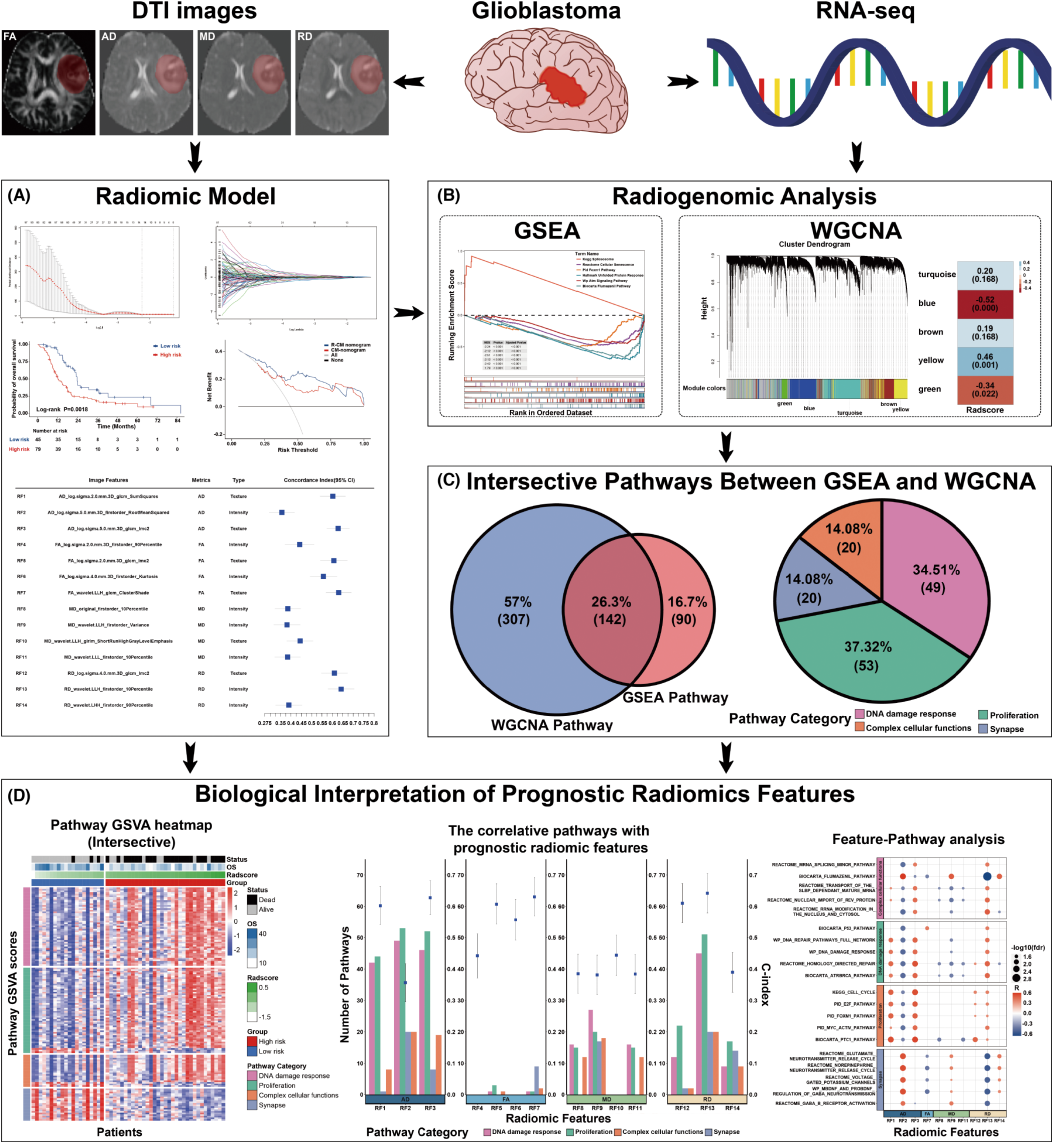
Workflow of this study. (A) Radiomic model construction and validation. (B) Radiogenomic analysis: the RNA‐seq data were analyzed using both GSEA and WGCNA approaches according to the conclusions of radiomic analysis. (C) Categories of intersective pathways. (D) Annotating individual prognostic radiomic feature.

The study procedures are illustrated in Figure 1 and consisted of radiomic model building, radiogenomic analysis, radiomic‐related pathway identification, and biological interpretation of radiomic features. First, we developed and validated a DTI‐based radiomic model to predict the prognosis of patients with GBM. We then used GSEA and WGCNA to identify the biological pathways associated with radiomic features. Third, the intersection of the pathways identified using the two approaches was selected as the final pathways. Finally, the underlying biological underpinning of the individual radiomic features and DTI metrics was revealed using Pearson correlation analysis and the Mantel test.

### 2.2 Study cohorts

A total of 258 adult patients pathologically diagnosed with IDH wild‐type GBM at the First Affiliated Hospital of Zhengzhou University during 2014–2021 were enrolled in this study as the radiomic dataset. The inclusion and exclusion criteria are shown in Figure S1. The radiomic dataset was divided into a training set (*N* = 134) and validation set (*N* = 124) using random sampling at an approximate ratio of 1:1 with balanced clinical parameters. Additionally, 53 patients from the radiomic dataset with RNA‐seq data of fresh frozen tumor tissues were designated as the radiogenomic set.

### 2.3 Image preprocessing, tumor delineation, and radiomic features extraction

We used a MATLAB toolbox named “PANDA” for fully automated processing of the original brain DTI images, including three main parts: stripping the skull, correcting for the eddy‐current effect, and calculating diffusion tensor metrics.^25^ Consequently, we obtained four types of images: AD, RD, MD, and FA maps. Moreover, all images were pre‐processed in the following four steps: (a) N4ITK‐based bias field distortion correction, (b) voxels resampling into 1 × 1 × 1 mm^3^, (c) rigid image alignment with fluid‐attenuated inversion recovery (FLAIR) as a template, and (d) histogram matching. The region of interest (ROI) was manually outlined layer‐by‐layer on the FLAIR images by a neuroradiologist with 12 years of experience (J Yan) using the ITK‐SNAP software (http://www.itksnap.org/pmwiki/pmwiki.php). Meanwhile, 15% (*N* = 39) of the images were randomly selected by a neurosurgeon with 12 years of experience (ZY Zhang) to repeat the ROI delineation process, yielding an inter‐rater test set. An open‐source Python package named “PyRadiomic” was used to extract the radiomic features and visual maps from the AD, RD, MD, and FA images.^26^ Next, the visual maps were visualized using the ITK‐SNAP software with the HSV (hue, saturation, and value) colormap.^27^ Detailed information on the image acquisition and feature extraction is provided in Supplementary A1 and Supplementary A2. This study obeyed image biomarker standardization initiative (IBSI) guidelines.^26^, ^28^ More details are presented in Supplementary A3 to ensure the robustness of the radiomics features.

### 2.4 Statistical analysis

*Radiomic model construction and validation*: We used a three‐step process for image features screening of the training set. Screening began by excluding low repeatability radiomic features. Intraclass correlation coefficients (ICCs) were calculated for each radiomic feature using the inter‐rater test set, and the radiomic features were deleted with an ICC <0.9. Next, we calculated the univariate concordance index (C‐index) of the remaining features to reflect the relationship between the radiomic features and OS. Radiomic features with a *p*‐value <0.05 and univariate C‐index ≥0.55 (positive association) or ≤0.45 (negative association) were retained for further analysis. Finally, least absolute shrinkage and selection operator (LASSO) penalized Cox proportional hazards regression analysis was used to select dependable radiomic features and build the radiomic model. The radiomic risk score (Radscore) was calculated as a linear combination of features with their nonzero coefficients generated by LASSO. The R package survminer was used to calculate the Radscore cutoff value for the training set. Then, the cutoff value was applied to the validation set.

The association between the Radscore and OS was evaluated using Kaplan–Meier analysis. A log‐rank test was used to assess the survival difference, where a *p*‐value <0.05 indicated a significant difference. Calibration curves were plotted to assess the agreement between predicted and observed survival. Decision curves were plotted to evaluate the clinical usefulness of the radiomic‐clinical model (R‐CM). The C‐index was calculated using the R package “survival” to measure the discrimination performance of the model. The net reclassification improvement (NRI) was calculated using the R package “survIDINRI” to assess the practicality improvement added by the radiomic model. The Akaike information criterion (AIC) was computed using R package “stats” to assess the risk of model overfitting. Decision curve analysis was performed using the R package “rmda” to confirm the clinical usefulness of the R‐CM.

*Radiogenomic analysis and radiomic‐related pathways identification*: We used two radiogenomic methods (GSEA and WGCNA) to enhance the reproducibility of the biological pathways. Detailed information on RNA‐seq and the detection of IDH mutations is provided in Supplementary A4 and Supplementary A5.

GSEA: First, Log2FoldChange values for each gene were obtained from differential gene expression analysis between high‐risk and low‐risk groups stratified based on the radiomic model. All genes sorted by Log2FoldChange value from high to low were subjected to GSEA, and pathways with a false discovery rate (FDR)‐adjusted hypergeometric *p*‐value <0.05 indicated significant enrichment. Pearson correlation analysis of the gene set variation analysis (GSVA) value of the significantly enriched pathways and Radscore was performed, and the pathways with an FDR <0.05 were retained. Differential analysis was performed using the R package “DESeq2.” GSEA was performed using the R package “clusterProfiler,” querying the following annotated gene set databases: Kyoto Encyclopedia of Genes and Genomes, Hallmark, Reactome, BioCarta, Pathway Interaction Database, WikiPathways.^29^

WGCNA: Cluster analysis with the “complete” method was used to delete outlier samples. Then, we used the R package “WGCNA” to perform WGCNA on the radiogenomic set. A *β*‐value of 8 (scale‐free *R* ^*2*^ = 0.85) was screened as soft thresholding shown in Figure S8. Five gene modules were also identified. Next, Radscores were subjected to Pearson correlation analysis with the principal components of the modules obtained from WGCNA, and the modules with an FDR <0.05 were retained. The genes in the retained modules were subjected to gene enrichment analysis, and the pathways with an FDR <0.05 were retained. Enrichment analysis was performed as described in the GSEA section.

*Biological interpretation of radiomic features*: First, we investigated the biological pathways underlying the individual radiomic feature. Pearson correlation analysis was performed between the prognostic radiomic features and GSVA scores of the intersective pathways. The pathways with an FDR <0.05 were selected to clarify the biological explanation of the individual prognostic radiomic feature. Second, we investigated the relationship between the DTI metrics and biological pathway categories. The top five most correlated pathways in each pathway category were analyzed using the Mantel test, which was conducted using the R package “vegan” for measuring the correlation between DTI metrics and the categories of intersectional pathways.

## 3 RESULTS

### 3.1 Patient characteristics

The demographic and clinical characteristics of the 258 patients are summarized in Table S1. Shapiro–Wilk test was used to analyze the distribution of training and validation sets. Results of the normality test revealed that none of the continuous variables (Age, KPS, and OS) conform to a normal distribution (*p* < 0.05). As a result, we employed the Wilcoxon rank sum test to compare the distribution differences of these variables (Age, KPS, and OS) between the training and validation sets. Test results showed no significant differences in age, Karnofsky performance score (KPS), and OS between the training and validation sets. The Chi‐square test demonstrated no significant differences in sex, extent of resection (Resection), radiation therapy (Radiation), chemotherapy, and survival status between the training and validation sets.

### 3.2 Radiomic model construction, validation, and its incremental prognostic value

*Radiomic model construction*: A three‐step process for image feature screening was performed. After the inter‐rater robustness was tested, 3173 of 4788 features remained. Univariate selection retained 496 features. Finally, 14 features, RF1‐RF14 selected by LASSO were used to calculate the Radscore as follows: Radscore = 0.0746297 × RF1–0.2063085 × RF2 + 0.0820216 × RF3–0.1122932 × RF4 + 0.0748861 × RF5 + 0.1218078 × RF6 + 0.0708374 × RF7–0.0464313 × RF8–0.1663953 × RF9–0.0845328 × RF10–0.0131809 × RF11 + 0.0209224 × RF12 + 0.0172892 × RF13–0.0176537 × RF14. Details of the LASSO Cox model are shown in Figure S3 and Figure S4. The features of RF1‐RF14 are shown in Figure S2. According to a radiomic training set‐based cutoff value determined by using R package “survminer,” patients were stratified into low‐risk (Radscore ≤ −0.2679513) and high‐risk (Radscore ≥ −0.2679513) groups, as shown in Figure S6.

**FIGURE 2:**
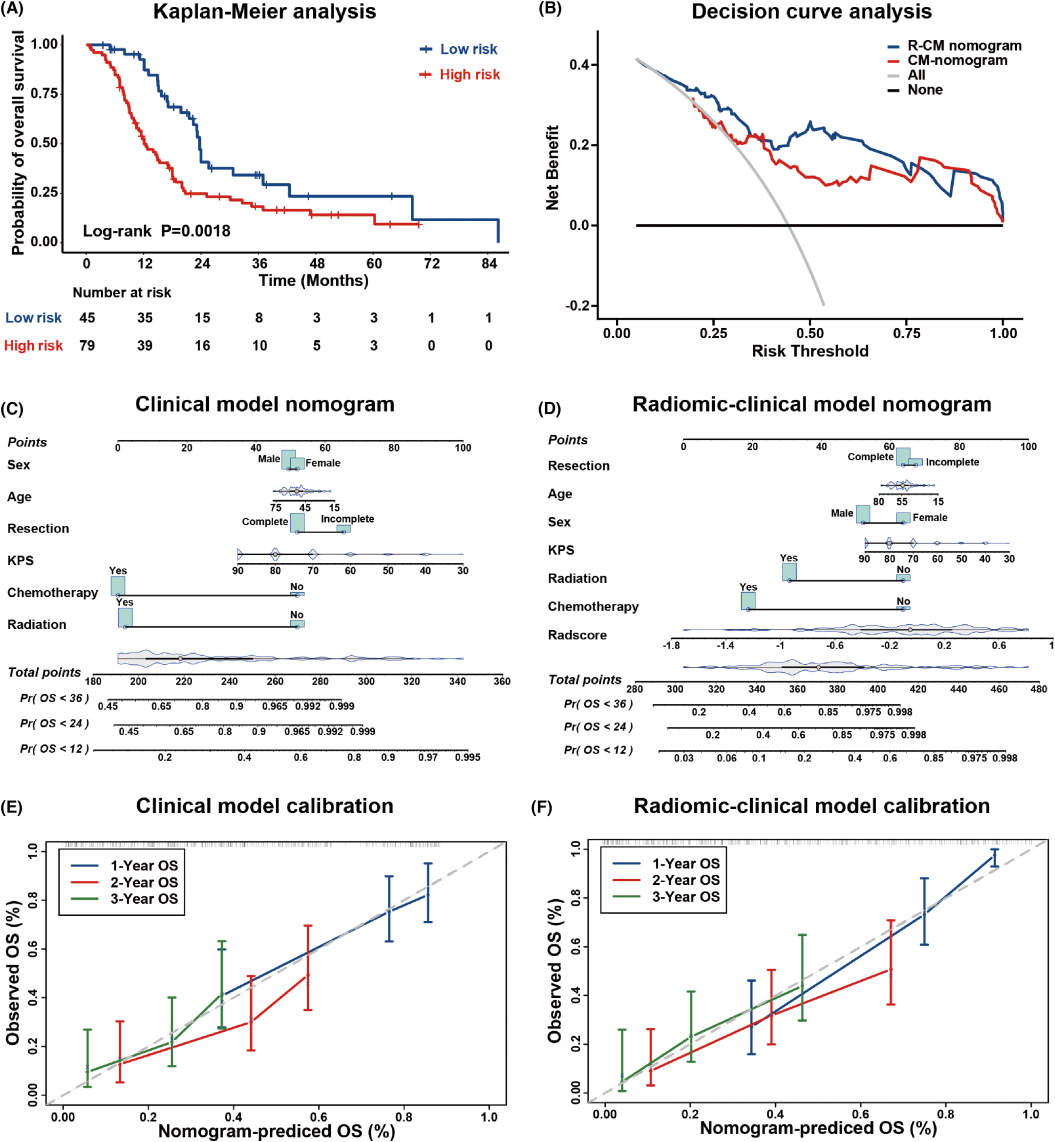
Validation of the radiomic signature. (A) Kaplan–Meier curves for patients stratified by the radiomic signature in the validation set. (B) Decision curve analysis for radiomic‐clinical model nomogram and clinical model nomogram to estimate the OS. The x‐axis represents the threshold probability, and the y‐axis measures the net benefit. (C–F) The clinical model nomogram (C) and the radiomic‐clinical model nomogram (D) for predicting the 1‐, 2‐, and 3‐year OS, along with the calibration curves for assessment of the clinical model nomogram (E) and the radiomic‐clinical model nomogram (F).

*Radiomic model validation*: As shown by Kaplan–Meier curves in Figure 2A and Figure S7A, the Radscore was significantly associated with OS in the training set (log‐rank *p* < 0.0001; hazard ratio [HR] = 6.632, 95% CI: 3.953, 11.130) and validation set (log‐rank *p* = 0.0018; HR = 3.024, 95% CI: 1.798, 5.086). Multivariate Cox analysis demonstrated that the Radscore was an independent risk factor in the training set (HR = 5.07; 95% CI: 3.01, 8.53; *p* < 0.001) and validation set (HR = 3.74; 95% CI: 2.08, 6.74; *p* < 0.001).

*Assessment of the incremental value of the radiomic signature*: The nomograms incorporating the clinical model (CM), or R‐CM for OS prediction are shown in Figure 2C,D, respectively. The calibration curves of the CM and R‐CM nomograms for the probability of 1‐, 2‐, and 3‐year deaths are shown in Figure 2E and Figure 2F, respectively. Compared with the CM nomogram, the R‐CM nomogram showed significantly better agreement. Table S2 demonstrates the C‐index and AIC values for the radiomic model, CM and R‐CM in the training and validation sets. The combination also yielded^1^: The NRI value for OS prediction on the training set is 0.333 (95% CI: 0.121, 0.556, *p* = 0.004) for OS prediction^2^; The NRI value for OS prediction on the validation set is 0.509 (95% CI: 0.224, 0.635, *p* = 0.004) for OS prediction. More details about the incremental value of radiomic model are shown in Figure S5. The decision curves of the validation set, illustrated in Figure 2B and Figure S7B show the clinical usefulness of the R‐CM.

### 3.3 Radiogenomic analysis: GSEA

**FIGURE 3:**
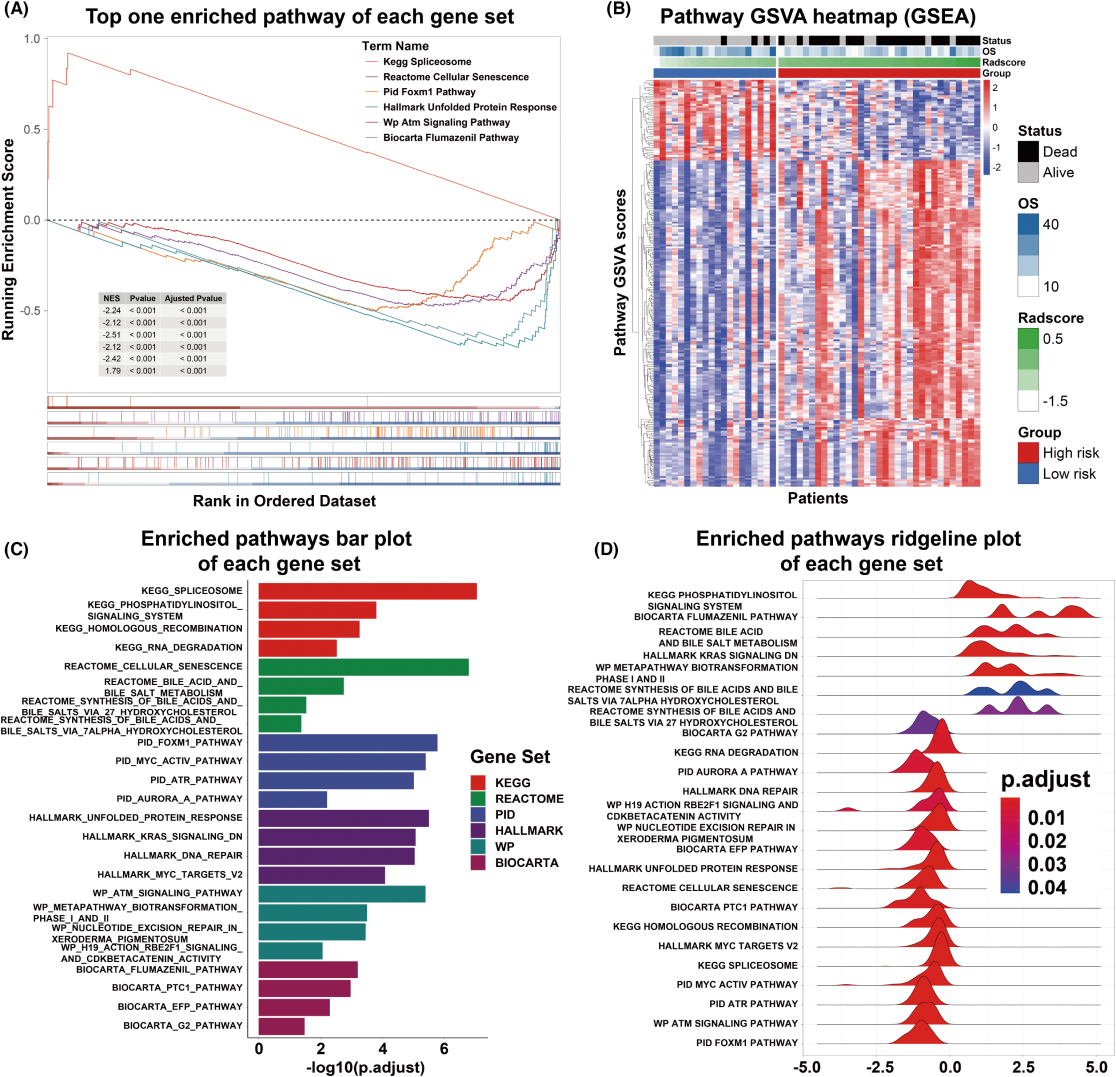
Results of gene set enrichment analysis. (A) Top enriched pathway in Kyoto Encyclopedia of Genes and Genomes (KEGG), Hallmark, Reactome, BioCarta, Pathway Interaction Database (PID), WikiPathways. (B) A heatmap of the gene set variation analysis (GSVA) score of GSEA pathways significantly correlated with the radiomic signature. (C) Bar plot of the top enriched pathways in each gene set. (D) Ridgeline plot of the top enriched pathways in each gene set.

First, 649 pathways were identified using GSEA. Second, the GSVA score of these pathways and Radscore were subjected to Pearson correlation analysis, and 232 pathways with an FDR <0.05 were subsequently screened. The top enriched pathway in each gene set is shown in Figure 3A. A heatmap of the GSVA score of the GSEA pathways in the radiogenomic set is shown in Figure 3B and Table S3. The top enriched pathways in each gene set are shown in Figure 3C and Figure 3D. The exact data points of Figure 3C are shown in Table S8.

### 3.4 Radiogenomic analysis: WGCNA

**FIGURE 4:**
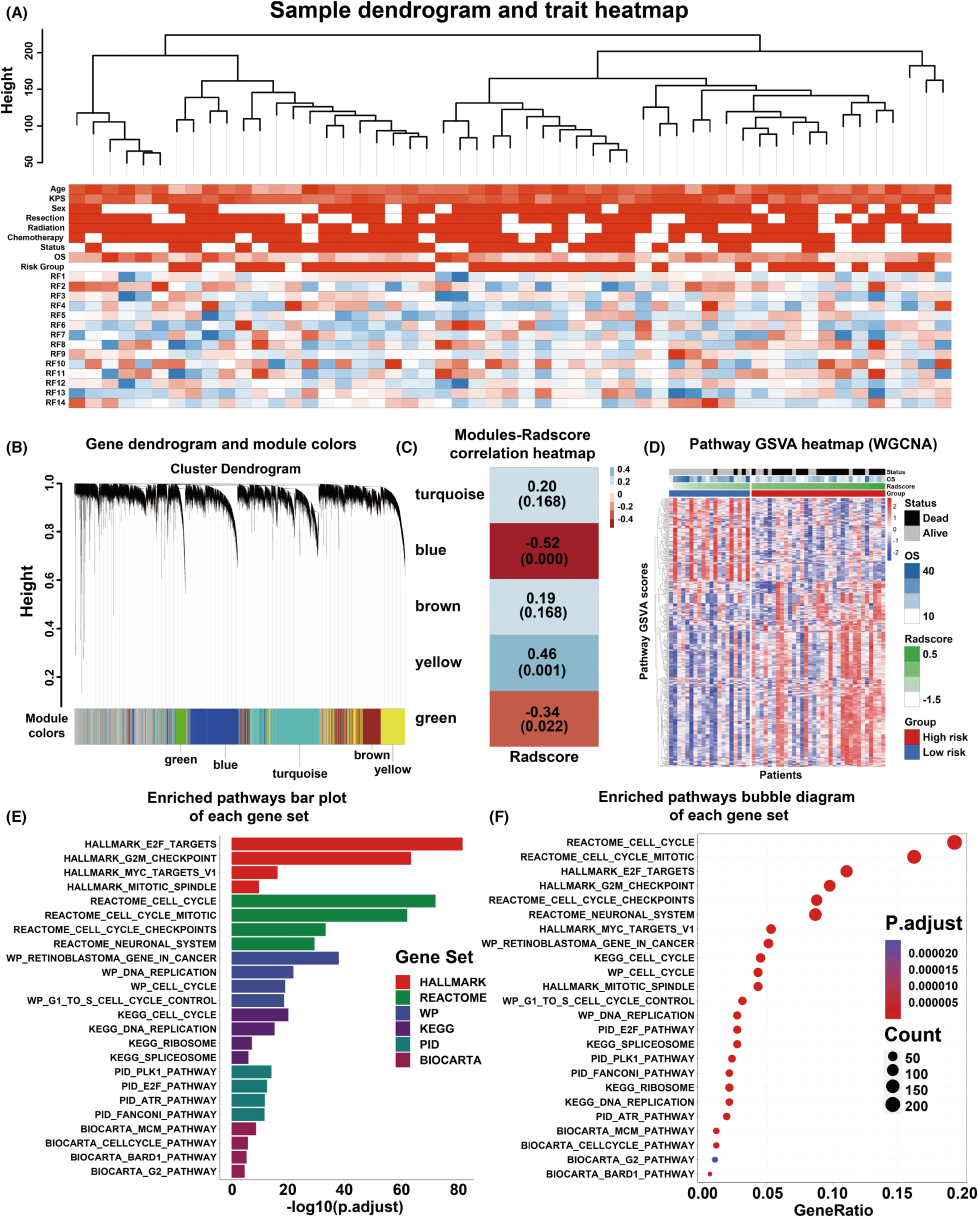
Results of weighted gene co‐expression network analysis. (A) Cluster analysis of patients in the radiogenomic set. (B) The modules obtained from WGCNA. (C) Heatmap of modules correlation with Radscore. (D) Results of pathway enrichment analysis of genes in the Radscore – related modules. (E) Bar plot of the top enriched pathways in each gene set. (F) Bubble diagram of the top enriched pathways in each gene set.

WGCNA yielded 5 gene modules: turquoise (3149 genes), blue (2535 genes), brown (1806 genes), yellow (1804 genes), and green (562 genes), as illustrated in Figure 4B. Correlations between the Radscore and first principal component of these modules were evaluated by Pearson correlation analysis, and the modules with an FDR <0.05 were selected for further pathway enrichment analysis. The Pearson FDR of the modules and paired Pearson correlation coefficients are shown in Figure 4C. Finally, 3 modules (blue, yellow, and green) of the 5 modules were correlated with the Radscore (blue module: Pearson correlation *r* = −0.52, FDR = 0.000; yellow module: Pearson correlation *r* = 0.46, FDR = 0.001; green module: Pearson correlation *r* = −0.34, FDR = 0.022). Genes in the 3 Radscore‐related modules are shown in Table S4. After performing pathway enrichment analysis of the 3 modules, 449 pathways with an FDR <0.05 were obtained, as illustrated in Table S5. A heatmap of the GSVA score of the WGCNA pathways in the radiogenomic set is shown in Figure 4D. The top enriched pathways in each gene set are shown in Figure 4E and Figure 4F. The exact data points of Figure 4E are shown in Table S9.

### 3.5 Intersective pathways of GSEA and WGCNA

**FIGURE 5:**
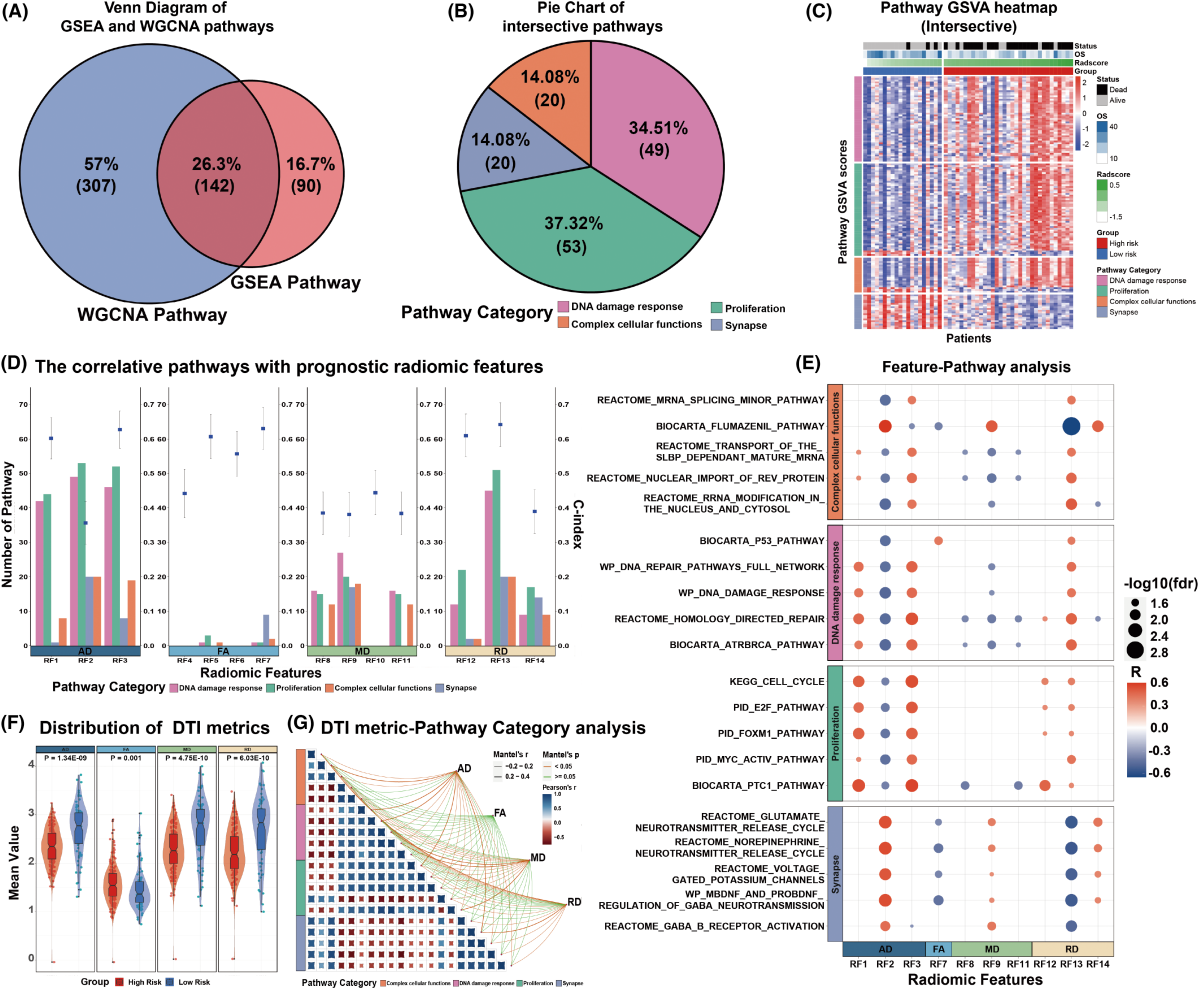
Radiogenomics linking between 14 radiomic features constituting the radiomic signature and their significantly associated pathways. (A) Venn diagram of the two approaches' pathways. (B) Categories of intersective pathways. (C) Heatmap of intersective pathways. (D) The number of relevant pathway species corresponding to each prognostic radiomic feature. (E) A bubble plot of correlation between prognostic radiomic features and classic biological pathways. (F) Violin Plot of the mean value of FA, MD, AD, and RD in the high‐ and low‐risk groups. (G) The correlation between the four DTI metrics and the significant pathways. Pairwise comparisons of biological pathways are shown, with a color gradient denoting Pearson's correlation coefficient.

By comparing the selected pathways from the previous GSEA and WGCNA approaches, 142 intersectional pathways were identified as the final result of the radiogenomic analysis, as illustrated in Figure 5A. These intersectional pathways were then classified into 4 categories: synapse, proliferation, DNA damage response, and complex cellular functions, as shown in Figure 5B and Table S6. A heatmap of the GSVA score of the intersectional pathways in the radiogenomic set is shown in Figure 5C.

### 3.6 Biological interpretation of the radiomic features

Biological interpretation of the radiomic features was performed from 2 perspectives (individual radiomic feature and DTI metrics).

**FIGURE 6:**
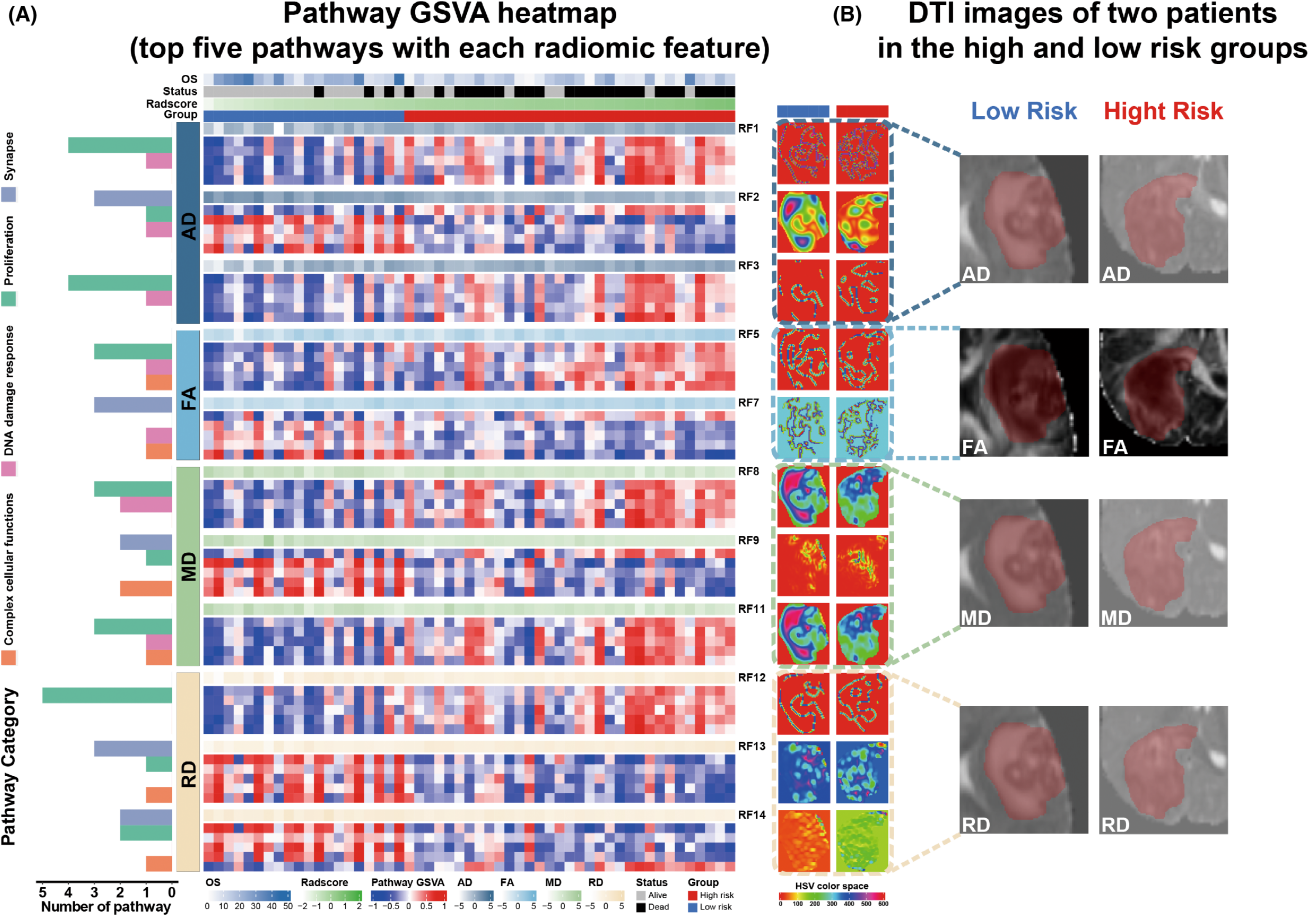
Radiogenomic linking between 14 radiomic features constituting the radiomic signature and their significantly associated pathways. (A) Left panel: Heatmap of 11 radiomics features along with their top significantly associated pathways. The five rows immediately after each radiomic feature indicate the activation level of the top significant pathways. Right panel: Feature maps delineating visual properties of the 11 radiomic features for two patients from the radiogenomic set in high‐ and low‐risk groups, respectively. (B) DTI metrics for the same two patients.

First, the correlation between the individual prognostic radiomic feature (N = 14) and 142 intersective pathways was evaluated using Pearson correlation analysis. As a result, 11 prognostic radiomic features were significantly associated with the intersectional pathways. The exact numbers of pathways that significantly correlated with the individual radiomic feature are shown in Figure 5D and Table S7. Representative pathways that were significantly correlated with the prognostic radiomic features are presented in Figure 5E. A heatmap of the individual prognostic radiomic feature with top pathways in 2 representative patients from the high‐ and low‐risk groups in the radiogenomic set is shown in Figure 6. Radiogenomic analysis showed that 6 radiomic features (i.e., RF1, RF3, RF5 RF8, RF11, and RF12) were mainly associated with the proliferation pathways, whereas the other 3 features (i.e., RF2, RF7, and RF13) were mainly associated with the synapse pathways. RF9 is mainly associated with synapses and complex cellular function pathways, whereas RF14 is mainly associated with synapse and proliferation pathways.

Second, there was a significant difference in the mean value of DTI metrics between the high‐ and low‐risk groups, as shown in Figure 5F. Correlations between the DTI metrics and pathway categories were investigated using the Mantel test, as shown in Figure 5G. The results suggest that AD is associated with DNA damage response, proliferation, synapse, and complex cellular function pathways; MD is mainly associated with DNA damage response and proliferation pathways; RD is mainly associated with synapse pathways; and no significant correlation was observed between FA and the categories of intersective pathways.

## 4 DISCUSSION

This study differs from previous radiogenomic studies in several respects. First, previous studies used either GSEA or WGCNA to identify biological pathways in radiogenomic analysis.^12^, ^13^ Instead of using a single genetic analysis method for pathway identification, we used both GSEA and WGCNA to acquire intersectional pathways for biological interpretation, which enhanced radiogenomic reproducibility. Second, individual radiomic features may be associated with multiple biological pathway categories instead of a single pathway or pathway category. Our study systematically investigated the categories of biological pathways underlying individual radiomic feature and their corresponding distributions. Third, previous studies have shown the significant value of DTI metrics in predicting prognosis in gliomas.^30^ However, the biological meaning of DTI metrics is poorly understood.^23^, ^30^ Herein, the biological underpinning of DTI metrics was investigated.

Studies have suggested that radiomic features are related to biological pathways in central nervous system tumors.^12^, ^13^, ^31^ A radiogenomic study based on WGCNA revealed that the pathways of tumor proliferation, immunity, and treatment response are associated with prognostic radiomic features in histologically diagnosed GBM.^13^ Another study revealed a strong association between the radiomic signature and pathways such as WNT signaling, the P53 pathway, and the PI3K/AKT pathway by differentially expressed gene analysis.^31^ Moreover, a radiogenomic study on histologically diagnosed GBM revealed associations between radiomic features and signaling pathways related to cell differentiation, cell adhesion, and angiogenesis.^12^ Collectively, these studies focus on conventional MR sequences‐based radiomic models and their biological interpretation, and the biological meaning underlying hand‐crafted radiomic features derived from advanced sequences such as DTI, remains elusive.

Our study elaborated on the biological interpretation of a DTI‐based radiomic model from the perspectives of individual radiomic feature and DTI metrics. For individual radiomic feature, we revealed the categories and number of biological pathways behind each feature. Our findings suggest that the biological pathways underlying individual prognostic radiomic feature are complex. For example, multiple biological processes may be involved in the individual feature. Our radiogenomic analysis revealed that 8 prognostic radiomic features (i.e., RF1‐RF3, RF7, RF9, and RF12‐RF14) were associated with DNA damage response, proliferation, synapse, and complex cellular function pathways, whereas the other 3 radiomic features (i.e., RF5, RF8, and RF11) were associated with DNA damage response, proliferation, and complex cellular function pathways. We also found a relationship between the prognostic value (C‐index of univariate Cox regression analysis) of radiomic features and the number of related pathways. The greater the prognostic value of a radiomic feature, the more biological pathways are related to it. More details about the relationship are shown in Figure S9. However, this does not apply to RF5‐RF7, which all belong to FA. A previous study suggested that FA reflects the integrity of nerve fibers and the degree of alignment of cellular structures.^32^ It has been demonstrated that FA could predict the prognosis of patients with GBM.^24^, ^33^ Previous studies have yielded contradictory results indicating that FA may not directly correlate with tumor cellularity.^34^, ^35^ We speculate that this may partly explain why FA has a high prognostic value, although it does not have potent biological significance.

For DTI metrics, we found a significant difference in the mean value of DTI metrics between the high‐ and low‐risk groups. A previous study also suggested that DTI metrics could predict the prognosis of patients with GBM.^33^ We further investigated the relationship between the DTI metrics and biological pathway categories. These results suggest that AD has a broad biological underpinning, consisting of multiple biological pathway categories. MD and RD were associated with specific biological categories, such as DNA damage response, proliferation, and synapse pathways. Unlike other DTI metrics, FA did not demonstrate significant biological significance in this study, which is consistent with previous radiogenomic findings of individual radiomic feature.

Our findings suggest the potential of a biologically explainable radiomic model for therapeutic applications. For example, the high‐risk group identified by the radiomic model was significantly correlated with distinct malignant tumor processes, such as DNA damage response, proliferation, and complex cellular functions, whereas the low‐risk group was significantly associated with synapse‐related processes. In recent years, studies have found that glioma cells can also exhibit synaptic activity and interact with neurons in the brain to promote tumor growth.^36^, ^37^ Glioma cells can express synaptic proteins and neurotransmitter receptors, which allow them to interact with neurons and modulate synaptic activity.^38^, ^39^ In addition to expressing synaptic proteins and neurotransmitter receptors, glioma cells can also release neurotransmitters themselves.^40^ This process can lead to increased neuronal activity, which in turn can stimulate glioma growth and invasion. Furthermore, glutamate released by glioma cells can also promote angiogenesis, which is the formation of new blood vessels that supply the tumor with nutrients and oxygen.^41^ Given the role of synaptic activity in glioma growth, invasion, and treatment resistance, targeting this process may represent a novel therapeutic strategy for the treatment of gliomas.^42^ Therefore, anti‐cellular proliferation therapies are suggested for patients with high radiomic risk scores, whereas therapies inhibiting neuron‐to‐tumor synaptic communication may be more effective in patients with low‐risk GBM defined by the radiomic model.^43^, ^44^

Our study has several limitations. First, this was a retrospective study, so future prospective multicenter studies are required to further corroborate our radiogenomic findings. Second, the current cohort included IDH wild‐type histologically diagnosed GBMs but lacked IDH wild‐type astrocytomas that were positive for TERT promoter mutations, EGFR amplification, or + 7/−10 chromosome copy number changes.^4^ Future studies including IDH wild‐type astrocytomas with molecular markers equal to GBM are needed to fully reflect the intratumor heterogeneity of IDH wild‐type GBM (integrated diagnosis of histology and molecular markers) according to the CNS5.^4^ Last, although the current study revealed the biological underpinning of DTI‐based radiomic features, future experiments at the protein and in vivo levels are required to confirm these findings.

In summary, this radiogenomic study demonstrated that prognostic radiomic features derived from DTI are driven by distinct pathways involved in synapse, proliferation, DNA damage response, and complex cellular functions. The proposed biologically explainable radiomic model may have the potential to inform therapeutic strategies for IDH wild‐type GBM.

## AUTHOR CONTRIBUTIONS

ZYZ, JY, and JlC performed the research conception. ZLW, FZG, WCD, MKW, YNQ, AQX, ZYL, YG, and DLP performed the data acquisition. ZLW and YG performed the data processing. ZLW and FZG performed the statistical analysis. BY, XZL, HWZ, DLY, JLC, and YCJ performed the project administration. ZLW, JY, and ZYZ performed the manuscript drafting. All authors have read and approved the final version of the manuscript.

## FUNDING INFORMATION

This research was funded by the National Natural Science Foundation of China (grant numbers: 82273493, 82102149, and 82173090), the Natural Science Foundation of Henan Province for Excellent Young Scholars (grant number: 232300421057), the Excellent Youth Talent Cultivation Program of Innovation in Health Science and Technology of Henan Province (grant number: YXKC2022061), the Key Program of Medical Science and Technique Foundation of Henan Province (grant number: SBGJ202002062), and the Science and Technology Program of Henan Province (grant numbers: 202102310136, 202102310454, 212102310113, and 192102310390). All funders played no role in the study design, data collection, analysis and interpretation of data, or the writing of this manuscript.

## CONFLICT OF INTEREST STATEMENT

All authors declare no financial or nonfinancial competing interests.

## Supporting information


Appendix S1:

## Data Availability

The datasets used and/or analyzed during the current study are available from the corresponding author upon reasonable request.
