# Supplementary material for: Diffusion tensor imaging‐based machine learning for IDH wild‐type glioblastoma stratification to reveal the biological underpinning of radiomic features
Source: CNS Neurosci Ther. 2023 May 24;29(11):3339–50. doi: 10.1111/cns.14263 (PMC10580329; doi:10.1111/cns.14263)

**Supplementary Material**

**This supplementary material includes:**

Supplementary A1: Image acquisition

Supplementary A2: A summary of the radiomics features extracted

Supplementary A3: A summary of the parameters according to Image Biomarker Standardisation Initiative (IBSI)

Supplementary A4: RNA samples preparation and sequencing

Supplementary A5: Detection of IDH mutation

Table S1. Characteristics of patients in training and validation sets

Table S2. C-indices and AIC values for OS prediction in training and validation sets

Table S3. A summary of the Radscore-related GSEA enriched pathways

Table S4. A summary of the genes in the three Radscore-related modules generated by WGCNA

Table S5. A summary of the pathways enriched by three Radscore-related modules

Table S6. Classification of intersective pathways between GESA and WGCNA approaches

Table S7. The number of relevant pathway species corresponding to each prognostic radiomic feature

Table S8. Exact data points of Figure 3C

Table S9. Exact data points of Figure 4E

Figure S1. The criteria for patients’ inclusion and exclusion

Figure S2. Forest plot of prognostic radiomic features

Figure S3. Radiomics feature selection 1

Figure S4. Radiomics feature selection 2

Figure S5. Incremental value of radiomic model

Figure S6. Radscore for each patient

Figure S7. Construction of the radiomic model

Figure S8. The Soft threshold selection process

Figure S9. The relationship between the prognostic value and the number of pathways

**Supplementary A1: Image acquisition**

Patients in the training and validation sets were all performed on 3.0 T clinical MR scanners with same manufacturers. The MRI protocol of all patients consists of the DTI and T2-FLAIR sequences. DTI was performed axially using a single-shot echo-planar imaging (EPI) sequence with b values of 0 s/mm2 and 1000 s/mm2. Detailed information about the MR machines and imaging parameters are summarized in the following.

| **Training and Validation sets** | |
| --- | --- |
| MR system | Siemens Verio |
| Field strength (T) | 3 |
| FLAIR |  |
| TR (ms) | 9000/6000 |
| TE (ms) | 96/94 |
| TI (ms) | 2500/2030 |
| Section thickness (mm) | 2/5 |
| Intersection gap (mm) | 0/1.5 |
| FA (°) | 150 |
| NEX | 2/1 |
| Pixel size (mm^3^) | 0.4883×0.4883×2/0.4297×0.4297×5.0 |
| Matrix | 256×174/256×157 |
| FOV (mm^2^) | 250×227/220×193 |
| ETL | 15/21 |
| DTI |  |
| TR (ms) | 9900/11600 |
| TE (ms) | 90/91 |
| Section thickness (mm) | 2 |
| Intersection gap (mm) | 0 |
| B value (s/mm^2^) | 0, 1000 |
| FA (°) | 90 |
| NEX | 2 |
| Pixel size (mm^3^) | 2.0×2.0×2.0 |
| Matrix | 128×128 |
| FOV (mm^2^) | 256×256 |
| ETL | 1 |
| Diffusion direction | 20/30 |

**Supplementary A2: A summary of the radiomics features extracted**

Fourteen shape features describing the 3D geometric characteristics of the VOI were extracted. From the original images and the transformed images (applying wavelet transform or LoG filtering), 234 intensity features describing the first-order distribution of the intensities were extracted, while 949 texture features were computed to describe the patterns, or the high-order intensity distributions with five methods, including gray-level co-occurrence matrix (GLCM), gray-level run length matrix (GLRLM), gray level size zone matrix (GLSZM), gray level dependence matrix (GLDM), and neighborhood gray-tone difference matrix (NGTDM). Informational Measure of Correlation have two calculation methods. Totally, 4788 features were extracted from five MR sequences.

| **Feature Classes** | | **Feature Names** |
| --- | --- | --- |
| Shape Features | | Elongation, Flatness, Least Axis Length, Major Axis Length, Maximum 2D Diameter Column, Maximum 2D Diameter Row, Maximum 2D Diameter Slice, Maximum 3D Diameter, Mesh Volume, Minor Axis Length, Sphericity, Surface Area, Surface Volume Ratio, Voxel Volume |
| Intensity Features | | Maximum, Median, Minimum, Mean, Energy, Entropy, Variance, Kurtosis, Root Mean Square, Skewness, 10th Percentile, 90th Percentile, Mean Absolute Deviation, Uniformity, Range, Robust Mean Absolute Deviation, Total Energy, Interquartile Range |
| Texture  Features | GLCM  Features | Contrast, Correlation, Autocorrelation, Cluster Tendency, Sum Average, Sum Entropy, Sum Squares, Difference Average, Difference Variance, Difference Entropy, Cluster Prominence, Cluster Shade, Maximum Probability, Inverse Difference Moment, Informational Measure of Correlation 1/2, Inverse Difference Moment Normalized, Inverse Difference Normalized, Inverse Difference, Inverse Variance, Maximal Correlation Coefficient, Joint Average, Joint Energy, Joint Entropy |
|  | GLDM  Features | Dependence Entropy, Dependence Non-Uniformity, Dependence Non-Uniformity Normalized, Dependence Variance, Gray-Level Non-Uniformity, Gray-Level Variance, High Gray-Level Emphasis, Large Dependence Emphasis, Large Dependence High Gray-Level Emphasis, Large Dependence Low Gray-Level Emphasis, Low Gray-Level Emphasis, Small Dependence Emphasis, Small Dependence High Gray-Level Emphasis, Small Dependence Low Gray-Level Emphasis |
|  | GLRLM  Features | Gray-Level Non-uniformity, Gray-Level Non-uniformity Normalized, Gray-Level Variance, High Gray-Level Run Emphasis, Long Run Emphasis, Long Run High Gray-Level Emphasis, Long Run Low Gray-Level Emphasis, Low Gray-Level Run Emphasis, Run Entropy, Run Length Non-Uniformity, Run Length Non-Uniformity Normalized, Run Percentage, Run Variance, Short Run Emphasis, Short Run High Gray-Level Emphasis, Short Run Low Gray-Level Emphasis |
|  | GLSZM  Features | Gray-Level Non-Uniformity, Gray-Level Non-Uniformity Normalized, Gray-Level Non-Uniformity Normalized, High Gray-Level Zone Emphasis, Large Area Emphasis, Large Area High Gray-Level Emphasis, Large Area Low Gray-Level Emphasis, Low Gray-Level Zone Emphasis, Size Zone Non-Uniformity, Size Zone Non-Uniformity Normalized, Small Area Emphasis, Small Area High Gray-Level Emphasis, Small Area Low Gray-Level Emphasis, Zone Entropy, Zone Percentage, Zone Variance |
|  | NGTDM  Features | Coarseness, Contrast, Busyness, Complexity, Strength |

**Supplementary A3: A summary of the parameters according to Image Biomarker Standardisation Initiative (IBSI)**

Details on image processing and biomarker extraction according to the IBSI guideline.

| **Topic** | **Description** |
| --- | --- |
| **Patients** | |
| Region of interest | Brain tumor |
| Patient preparation | Administration of sedative hypnotics to the patients who cannot cooperate with the examination prior to image acquisition; The use of ear plugs for patient comfort during scanning |
| Contrast agent | See details in MRI Sequence parameters in this supplementary material |
| Comorbidities | None |
| **Acquisition** | |
| Acquisition protocol | See details in MRI Sequence parameters in this supplementary material |
| Scanner type | See details in MRI Sequence parameters in this supplementary material |
| Imaging modality | See details in MRI Sequence parameters in this supplementary material |
| Static/dynamic scans | Static |
| Scanner calibration | Phantom scan, automatic prescan, and coil sensitivity calibration scan are used for calibration |
| Patient instructions | Tell the patient not to move during the acquisition and to be calm and relaxed |
| Anatomical motion correction | The scanned position is head. Normally there is no motion artifacts from heart beating and breathing. We will always tell the patient not to move before scan. And we will check the image right after every scan. If there is a motion, we will discard that scan and rescan |
| Scan duration | From 6 minutes and 46 seconds to 8 minutes and 57 seconds |
| RF coil | See details in MRI Sequence parameters in this supplementary material |
| Scanning sequence | See details in MRI Sequence parameters in this supplementary material |
| Repetition time | See details in MRI Sequence parameters in this supplementary material |
| Echo time | See details in MRI Sequence parameters in this supplementary material |
| Echo train length | See details in MRI Sequence parameters in this supplementary material |
| Inversion time | See details in MRI Sequence parameters in this supplementary material |
| Flip angle | See details in MRI Sequence parameters in this supplementary material |
| Acquisition type | 2D |
| k-space traversal | The acquisition trajectory for AD, FA, MD, and RD image is Cartesian |
| Number of averages/excitations | See details in MRI Sequence parameters in this supplementary material |
| Magnetic field strength | 3.0 T |
| **Reconstruction** | |
| In-plane resolution | We describe in-plane resolution as the field of view and matrix size. See details in MRI Sequence parameters in this supplementary material |
| Image slice thickness | 5.0 mm |
| Image slice spacing | 1.5 mm |
| Reconstruction method | The normal reconstruction method used to reconstruct the image from the k-space information are 2D Fourier transformation. For contrast-enhanced image, we use the difference image. The asset calibration is here used during reconstruction to suppress artifacts from coil sensitivity. |
| Diffusion-weighted imaging | b = 0 and b = 1000 s/mm^2^ |
| **Image registration** | |
| Registration method | Rigid registration was performed using axial resampled FLAIR as a template with mutual information similarity metric |
| **Image processing** | |
| **Data conversion** | |
| ADC computation | See details in MRI Sequence parameters in this supplementary material |
| **Post-acquisition processing** | |
| Anti-aliasing | None |
| Noise suppression | AD, FA, MD, and RD denoising was done by using fast non-local means tool within 3DSlicer |
| Skull stripping | A skull-stripping filter included in ITK software (<https://itk.org/>) |
| Non-uniformity correction | N4ITK-based bias field distortion correction |
| Intensity normalization | Histogram matching |
| Other post-acquisition processing methods | None |
| **Segmentation** | |
| Segmentation method | Manually |
| Conversion to mask | Directly generated by using ITK-SNAP software |
| **Image interpolation** | |
| Interpolation method | Trilinear interpolation |
| Voxel dimensions | 1x1x1 mm^3^ |
| **ROI interpolation** | |
| Interpolation method | Not applicable |
| Partially masked voxels | Not applicable, because the image was interpolated before ROI was segmented |
| **Re-segmentation** | |
| Re-segmentation methods | Not applicable, we did not use re-segmentation |
| **Discretization** | |
| Discretization method | Fix bin number |
| **Image transformation** | |
| Image filter | Wavelet and Laplacian of Gaussian filters, see details in Supplementary material |
| **Image biomarker computation** | |
| Biomarker set | Training data set (n = 258) |
| IBSI compliance | the software used is compliant with the IBSI benchmarks (digit phantom and radiomics CT phantom). |
| Robustness | Test-retest and multi-delineation test |
| Software availability | Pyradiomics 3.0 |
| **Image biomarker computation – texture parameters** | |
| Texture matrix aggregation | GLCM, GLRLM features were computed from a single matrix after merging all 3D directional matrices; GLSZM, GLDM and NGTDM features were computed from a 3D matrix |
| Distance weighting | No weighting |
| CM symmetry | Symmetric |
| CM distance | 1 |
| SZM linkage distance | 1 |
| DZM linkage distance | 1 |
| NGTDM distance | 1 |
| **Machine learning and radiomics analysis** | |
| Diagnostic and prognostic modeling | Our radiomics prognostic study adhered to TRIPOD reporting guideline of a multivariable prediction model for individual prognosis or diagnosis |
| Comparison with known factors | Yes, compared with clinic molecular factors including age, gender (female or male), KPS, extent of resection (complete or incomplete), radiation therapy (yes or no) and chemotherapy (yes or no) |
| Multicollinearity | Limited the possible effect of multicollinearity by using LASSO penalized Cox proportional hazards regression in radiomics signature development; For radiomic-clinical model and clinical model, variance inflation factor (VIF) was used to assess multicollinearity. All VIFs were less than 10, indicating there were no problematic multicollinearity. |
| Model availability | Available via reasonable request from the corresponding authors |
| Data availability | Available via reasonable request from the corresponding authors |

**Supplementary A4: RNA samples preparation and sequencing**

(1) RNA quantification and qualification: RNA degradation and contamination were monitored on 1% agarose gels. RNA purity was checked using the NanoPhotometer® spectrophotometer (IMPLEN, CA, USA). RNA concentration was measured using Qubit® RNA Assay Kit in Qubit® 2.0 Flurometer (Life Technologies, CA, USA). RNA integrity was assessed using the RNA Nano 6000 Assay Kit of the Bioanalyzer 2100 system (Agilent Technologies, CA, USA).

(2) Library preparation for Transcriptome sequencing: A total amount of 3 µg RNA per sample was used as input material for the RNA sample preparations. Sequencing libraries were generated using NEBNext® UltraTM RNA Library Prep Kit for Illumina® (NEB, USA) following manufacturer’s recommendations and index codes were added to attribute sequences to each sample. Briefly, mRNA was purified from total RNA using poly-T oligo-attached magnetic beads. Fragmentation was carried out using divalent cations under elevated temperature in NEBNext First Strand Synthesis Reaction Buffer (5X). First strand cDNA was synthesized using random hexamer primer and M-MuLV Reverse Transcriptase (Rnase H-). Second strand cDNA synthesis was subsequently performed using DNA Polymerase I and RNase H. Remaining overhangs were converted into blunt ends via exonuclease/polymerase activities. After adenylation of 3’ ends of DNA fragments, NEBNext Adaptor with hairpin loop structure were ligated to prepare for hybridization. In order to select cDNA fragments of preferentially 150~200 bp in length, the library fragments were purified with AMPure XP system (Beckman Coulter, Beverly, USA). Then 3 µl USER Enzyme (NEB, USA) was used with size-selected, adaptor-ligated cDNA at 37°C for 15 min followed by 5 min at 95 °C before PCR. Then PCR was performed with Phusion High-Fidelity DNA polymerase, Universal PCR primers and Index (X) Primer. At last, PCR products were purified (AMPure XP system) and library quality was assessed on the Agilent Bioanalyzer 2100 system.

(3) Clustering and sequencing: The clustering of the index-coded samples was performed on a cBot Cluster Generation System using TruSeq PE Cluster Kit v3-cBot-HS (Illumia). After cluster generation, the library preparations were sequenced on an Illumina Hiseq platform and 125 bp/150 bp paired-end reads were generated.

(4) Quality control: Raw data (raw reads) of fastq format were firstly processed through in-house perl scripts. In this step, clean data (clean reads) were obtained by removing reads containing adapter, reads containing ploy-N and low-quality reads from raw data. At the same time, Q20, Q30 and GC content the clean data were calculated. All the downstream analyses were based on the clean data with high quality.

(5) Reads mapping to the reference genome: Reference genome and gene model annotation files were downloaded from genome website directly. Index of the reference genome was built using STAR and paired-end clean reads were aligned to the reference genome using STAR (v2.5.1b). STAR used the method of Maximal Mappable Prefix (MMP) which can generate a precise mapping result for junction reads.

(6) Quantification of gene expression level: HTSeq v0.6.0 was used to count the reads numbers mapped to each gene. And then FPKM (expected number of Fragments Per Kilobase of transcript sequence per Millions base pairs sequenced) of each gene was calculated based on the length of the gene and reads count mapped to this gene.

**Supplementary A5: Detection of IDH mutation**

Mutational hotspots of IDH1/IDH2 were evaluated by direct sequencing. Tissues from representative tumor area (the proportion of tumor cells＞20%) were scrapped off from dewaxed sections and treated with PCR reaction solution A 10μl (reaction mixture containing 1μl of cell lysate, 0.3mM of each dNTP, 2.5mM MgCl2, 0.3μM of each primer and 0.2U of KAPA HiFi HotStart DNA Polymerase (Kapa Biosystems Inc., Wilmington, USA)), Shrimp Alkaline Phosphatase (SAP) enzyme (NEB, Ipswich, MA, USA) 2μl and BigDye (BigDye™ Terminator v3.1 Cycle Sequencing Kit, Thermo Fisher Scientific, Waltham, MA, USA) 1μl for centrifugation at 2000 rpm for 10 sec. The crude cell lysate was centrifuged and supernatant was used for subsequent PCR analysis. The forward primer primers (IDH1-F:5’-CGGTCTTCAGAGAAGCCATT-3’,IDH1-R:5’-CACATTATTGCCAACATGAC-3’,IDH2-F:5’-AGCCCATCATCTGCAAAAAC-3’,IDH2-R:5’-CTAGGCGAGGAGCTCCAGT-3’) were used to amplify the region of mutational hotspots of IDH1/IDH2. ①PCR was performed was initiated at 95°C for 5 min, followed by 40 cycles of 95°C for 20 sec, 57°C for 30 sec and 72°C for 1min, and a final extension of 72°C for 5 min and 10°C for 10 min. ②5μl PCR products were then mixed with 2μl SAP enzyme and reacted at 37°C for 40min and then at 80° C for 15min. ③Then 18μl PCR reaction solution C（CWBIO, Beijing, Chima）, 1μl products from ② step, and 1μl BigDye were mixed and reacted at 96°C for 1 min, followed by 30 cycles of 96°C for 10 sec, 50° C for 5 sec and 60° C for 2 min, and a final extension of 25°C for 1 min and 10°C for 10 min. Then 50μl natrium asceticism-ethanol mixture (3M NaAc: ethanol=1:15) were added and the mixture was centrifuged for 30min (12000 rpm, 4°C), with the supernatant being discarded. Then 70μl 75% ethanol were added and the mixture was centrifugated for 15min (12000 rpm, 4°C), with the supernatant being discarded. After complete volatilization of the ethanol at room temperature, 12μl Hi-Di™ Formamide (Thermo Fisher Scientific, Waltham, MA, USA) were added into the precipitate to dissolve the DNA. The dissolved products were sequenced on Applied Biosystems™ 3500DxGenetic Analyzer (Thermo Fisher Scientific, Waltham, MA, USA), and analyzed by Chromas software (Technelysium, South Brisbane, Australia). The sequencing results were compared with wild-type sequences of IDH1/IDH2 for analysis.

**Table S1.** Characteristics of patients in training and validation sets

|  | Training Set (N = 134) | Validation Set (N = 124) | P value |
| --- | --- | --- | --- |
| **Age (Year)** |  |  |  |
| Mean (SD) | 53.8 (11.6) | 53.3 (11.6) | 0.638 |
| Median [Min, Max] | 54.0 [19.0, 78.0] | 54.0 [19.0, 78.0] |  |
| **Sex** |  |  |  |
| Male | 76 (56.7%) | 78 (62.9%) | 0.376 |
| Female | 58 (43.3%) | 46 (37.1%) |  |
| **KPS** |  |  |  |
| Mean (SD) | 75.7 (13.9) | 74.1 (14.8) | 0.517 |
| Median [Min, Max] | 80.0 [40.0, 100] | 80.0 [30.0, 90.0] |  |
| **Extent of resection** |  |  |  |
| Complete | 93 (69.4%) | 90 (72.6%) | 0.671 |
| Incomplete | 41 (30.6%) | 34 (27.4%) |  |
| **Radiation therapy** |  |  |  |
| Yes | 87 (64.9%) | 92 (74.2%) | 0.139 |
| No | 47 (35.1%) | 32 (25.8%) |  |
| **Chemotherapy** |  |  |  |
| Yes | 113 (84.3%) | 107 (86.3%) | 0.788 |
| No | 21 (15.7%) | 17 (13.7%) |  |
| **Status** |  |  |  |
| Dead | 87 (64.9%) | 91 (73.4%) | 0.182 |
| Alive | 47 (35.1%) | 33 (26.6%) |  |
| **OS (Month)** |  |  |  |
| Mean (SD) | 20.3 (14.1) | 19.7 (16.1) | 0.316 |
| Median [Min, Max] | 17.8 [2.00, 86.6] | 15.0 [0.700, 86.2] |  |

**Table S2.** C-indices and AIC values for OS prediction in training and validation sets

| **Model** | **C-index** | **AIC** |
| --- | --- | --- |
|  | Training Set | |
|  | OS | |
| Radiomics model | 0.753015 (0.705978 0.800053) | 686.5578 |
| Clinical model | 0.745475 (0.692280 0.798670) | 703.2209 |
| Radiomics-clinical model | 0.798597 (0.756331 0.840864) | 665.8608 |
|  |  |  |
|  | Validation Set | |
|  | OS | |
| Radiomics model | 0.677404 (0.622267 0.732540) | 715.3585 |
| Clinical model | 0.697724 (0.639897 0.755550) | 697.9155 |
| Radiomics-clinical model | 0.751962 (0.704714 0.799210) | 678.3935 |

**Table S3.** A summary of the Radscore-related GSEA enriched pathways

GSEA was performed using the R package clusterProfiler, querying the following annotated gene sets: Kyoto Encyclopedia of Genes and Genomes (KEGG), Hallmark, Reactome, BioCarta, Pathway Interaction Database (PID), WikiPathways.

| **Pathway** | **Enrichment Score** | **Enrichment's FDR** | **Core_Enrichment** | | **Pearson Correlation's FDR** | | | | **Database** |
| --- | --- | --- | --- | --- | --- | --- | --- | --- | --- |
| BIOCARTA_ATM_PATHWAY | -0.71073 | 0.001135 | CDKN1A/NFKB1/RELA/ABL1/TP53/RBBP8/JUN/BRCA1/CHEK1/CHEK2/TP73/RAD51/NFKBIA | | 0.004605 | | | | BIOCARTA |
| BIOCARTA_ATRBRCA_PATHWAY | -0.72017 | 0.000231 | RAD9A/TP53/FANCE/BRCA1/FANCA/FANCC/BRCA2/CHEK1/CHEK2/FANCD2/RAD51 | | 0.025179 | | | | BIOCARTA |
| BIOCARTA_EFP_PATHWAY | -0.69353 | 0.004917 | CDK3/TP53/CDK2/CCNB1/CDK4/CDK1/CCNB2/CDK6 | | 0.000614 | | | | BIOCARTA |
| BIOCARTA_FLUMAZENIL_PATHWAY | 0.922103 | 0.000613 | GABRA1/GABRA4/GABRA5/GABRA2/GABRA3/PRKCE | | 0.001359 | | | | BIOCARTA |
| BIOCARTA_G2_PATHWAY | -0.55918 | 0.03252 | WEE1/CDKN1A/CDC25B/TP53/BRCA1/CHEK1/CDC25A/PLK1/CHEK2/CCNB1/CDK1/CDC25C | | 0.002304 | | | | BIOCARTA |
| BIOCARTA_HIVNEF_PATHWAY | -0.47705 | 0.002545 | RIPK1/RB1/FADD/TRADD/CDK11A/TRAF2/BID/MAP3K14/FAS/NFKB1/TNFRSF1A/RELA/DAXX/BIRC2/LMNA/ACTG1/TRAF1/MAP3K1/CASP2/CASP3/LMNB2/TNFRSF1B/LMNB1/NFKBIA/BIRC3 | | 0.043823 | | | | BIOCARTA |
| BIOCARTA_P53_PATHWAY | -0.61741 | 0.031493 | RB1/CDKN1A/BAX/E2F1/CCNE1/TP53/CDK2/PCNA/CDK4 | | 0.040746 | | | | BIOCARTA |
| BIOCARTA_PROTEASOME_PATHWAY | -0.65228 | 0.012247 | PSMA3/PSMA1/PSMB1/PSMB2/PSMA5/PSMB4/PSMB7/PSMB6/PSMA6/PSMA4/PSMB3/PSMA7/PSMA2/RPN1/RPN2 | | 0.00632 | | | | BIOCARTA |
| BIOCARTA_PTC1_PATHWAY | -0.83769 | 0.001042 | CDC25B/CDC25A/CCNB1/CDK1/CDC25C/SHH | | 0.001404 | | | | BIOCARTA |
| BIOCARTA_RACC_PATHWAY | 0.774417 | 0.019545 | KCNQ5/KCNQ3/ADCY1/GUCY1B1/GUCY1A1/TRPC3/KCNQ2/GUCY1A2 | | 0.028309 | | | | BIOCARTA |
| BIOCARTA_RANMS_PATHWAY | -0.78904 | 0.006408 | RANBP1/KPNA2/RCC1/AURKA/TPX2/KIF15 | | 0.01355 | | | | BIOCARTA |
| BIOCARTA_RB_PATHWAY | -0.66853 | 0.03943 | RB1/WEE1/CDC25B/TP53/CDK2/CHEK1/CDC25A/CDK4/CDK1/CDC25C | | 0.0184 | | | | BIOCARTA |
| BIOCARTA_SM_PATHWAY | -0.72283 | 0.006455 | SNRPE/SNRPF/SRSF2/U2AF1/SNRPD1/SNRPC/SNRPA1/SNRPB2/SNRPD2/SNRPG/SNRPA/SNRNP70/SNRPB | | 0.00681 | | | | BIOCARTA |
| HALLMARK_DNA_REPAIR | -0.41172 | 8.66E-06 | CETN2/HCLS1/RALA/SNAPC4/UMPS/POLR2J/PRIM1/ZNF707/NELFCD/IMPDH2/SRSF6/ELOA/CDA/RAE1/DGUOK/POM121/GTF3C5/POLR2D/NELFE/POLH/AAAS/SF3A3/TMED2/DGCR8/CLP1/ADRM1/POLA1/ITPA/NME4/POLR2H/POLD3/TARBP2/NT5C/CANT1/SSRP1/NME1/POLR1C/DDB2/VPS37B/NT5C3A/SEC61A1/GSDME/TP53/PNP/ADA/POLD1/RPA3/FEN1/ALYREF/POLA2/RFC4/ELL/PDE6G/PCNA/RFC2/LIG1/RFC3/ZWINT/TYMS/RAD51 | | 0.003861 | | | | HALLMARK |
| HALLMARK_KRAS_SIGNALING_DN | 0.602166 | 8.16E-06 | RYR2/SNCB/SLC30A3/ARHGDIG/KCNN1/ABCG4/ARPP21/PNMT/CYP39A1/SPTBN2/ADRA2C/CPEB3/TFCP2L1/HTR1D/THRB/MAST3/CAMK1D/NOS1/TENM2/ACTC1/MYOT/PTGFR/PRODH/ZBTB16/CLSTN3/MYO15A/PRKN/FGF22/LYPD3/RYR1/HTR1B/SLC25A23/RSAD2/EPHA5/SLC38A3/MFSD6/NRIP2/SIDT1/PKP1/DLK2/KCNQ2/NUDT11/DTNB/EFHD1/CNTFR/FGFR3/MX1/SELENOP/PCDHB1/SNN/SOX10/IFI44L/P2RX6/CCNA1/RGS11/SLC16A7/PDK2/ZC2HC1C | | 0.005065 | | | | HALLMARK |
| HALLMARK_MYC_TARGETS_V2 | -0.55105 | 7.95E-05 | UNG/RABEPK/PHB/MCM4/DCTPP1/TCOF1/PPRC1/LAS1L/RRP12/NPM1/TFB2M/SRM/MRTO4/DDX18/NIP7/WDR74/PUS1/WDR43/NOC4L/MYBBP1A/UTP20/GRWD1/TBRG4/AIMP2/NOP56/MYC/PPAN/NOP16/TMEM97/GNL3/PES1/RRP9/CBX3/BYSL/MCM5/NOP2/IPO4/PLK1/CDK4/PLK4/HK2 | | 0.000693 | | | | HALLMARK |
| HALLMARK_UNFOLDED_PROTEIN_RESPONSE | -0.47478 | 3.01E-06 | KHSRP/FUS/NPM1/CXXC1/TARS1/SEC31A/IARS1/DNAJC3/EXOSC2/BANF1/SLC30A5/EIF2S1/SEC11A/SRPRB/CEBPG/SRPRA/EXOSC9/EIF4A3/ASNS/NABP1/DKC1/YIF1A/SHC1/ATF4/KDELR3/ZBTB17/NOP56/HYOU1/EXOSC4/EDEM1/DNAJB9/ERN1/PDIA6/H2AX/CALR/RRP9/XBP1/CKS1B/ARFGAP1/FKBP14/EIF4A1/WIPI1/MTHFD2/HSP90B1/DDIT4/PDIA5/ERO1A/HSPA5/EIF4EBP1/STC2/ATF3/CEBPB/CCL2/IGFBP1/VEGFA | | 0.001241 | | | | HALLMARK |
| KEGG_CALCIUM_SIGNALING_PATHWAY | 0.701073 | 6.59E-09 | GRIN1/ATP2B3/CACNA1B/CCKBR/CAMK2A/RYR2/PRKCG/SLC8A2/CHRM1/GRM1/HTR2A/PTGER3/CACNA1I/GRIN2A/CACNA1E/GRM5/GNAL/PRKCB/PDE1A/HTR6/ADRA1A/ADRB1/ADRA1B/DRD1/CAMK4/ATP2B2/PDE1B/CAMK2B/TACR1/ITPKA/P2RX5/CHRM3/ADCY2/NTSR1/ADCY1/CACNA1G/NOS1/CACNA1C/PHKA1/GRIN2C/CAMK2G/ITPR1/PTGFR/PLCB1/PLCB4/PPP3CB/MYLK3/ERBB4/CACNA1D/CALM3/RYR3/RYR1/ADRB2/PPP3R1/PRKACB/GNA14/PPP3CA/TACR2/PTK2B/SLC25A4/CACNA1A/HRH2/CALM1 | | 0.006329 | | | | KEGG |
| KEGG_CELL_CYCLE | -0.54817 | 6.59E-09 | STAG1/CCNE2/ORC5/RB1/WEE1/SKP2/ANAPC11/TGFB3/CDKN1A/HDAC1/RBL1/CCND2/GADD45B/CDC25B/MCM6/ZBTB17/CDC7/MYC/MAD1L1/ABL1/E2F1/CCNE1/TP53/MCM3/ORC6/GADD45G/MCM5/CDK2/PCNA/MCM2/PTTG1/PKMYT1/MAD2L2/MAD2L1/CHEK1/TTK/CDKN2C/CDC25A/PLK1/CCNA2/BUB1B/CHEK2/CCNB1/CDK4/CDK1/MCM7/DBF4/CCNB2/BUB1/CDC6/ORC1/E2F2/CDC20/ESPL1/CDC25C/CDC45/CDK6 | | 0.01358 | | | | KEGG |
| KEGG_HOMOLOGOUS_RECOMBINATION | -0.66157 | 0.000537 | MRE11/POLD4/RAD52/TOP3B/MUS81/RPA1/SSBP1/SEM1/POLD3/POLD2/TOP3A/POLD1/RAD54B/RPA3/BLM/BRCA2/XRCC2/RAD54L/RAD51/EME1 | | 0.001234 | | | | KEGG |
| KEGG_LONG_TERM_DEPRESSION | 0.691784 | 2.5E-05 | PRKCG/GRM1/GRM5/PRKCB/CRHR1/PPP1R17/IGF1/NOS1/GUCY1B1/ITPR1/GNAO1/PLCB1/PLCB4/GUCY1A1/RYR1/GRIA2/GNAI1/PLA2G5/PRKG1/CACNA1A/MAPK3/GUCY1A2/GRIA1/GNAZ/MAP2K1 | | 0.013013 | | | | KEGG |
| KEGG_MISMATCH_REPAIR | -0.67017 | 0.001628 | RFC5/PMS2/POLD4/MLH1/MSH6/RPA1/SSBP1/POLD3/POLD2/POLD1/RPA3/RFC4/PCNA/RFC2/LIG1/RFC3/EXO1 | | 0.028634 | | | | KEGG |
| KEGG_NUCLEOTIDE_EXCISION_REPAIR | -0.48534 | 0.008175 | GTF2H3/RFC5/ERCC1/CUL4A/GTF2H4/POLD4/GTF2H2/CDK7/CETN2/RPA1/POLE3/POLD3/POLD2/DDB2/POLD1/RPA3/RFC4/PCNA/RFC2/LIG1/POLE2/POLE/RFC3 | | 0.019081 | | | | KEGG |
| KEGG_P53_SIGNALING_PATHWAY | -0.56192 | 7.22E-06 | BID/CDKN1A/BBC3/FAS/CCND2/GADD45B/PMAIP1/PIDD1/DDB2/BAX/CCNE1/TP53/CASP3/THBS1/SESN2/TNFRSF10B/GADD45G/CDK2/CHEK1/STEAP3/CHEK2/CCNB1/CDK4/CDK1/MDM4/TP73/CCNB2/GTSE1/CD82/RRM2/CDK6/SERPINE1/IGFBP3 | | 0.023266 | | | | KEGG |
| KEGG_PHOSPHATIDYLINOSITOL_SIGNALING_SYSTEM | 0.645639 | 0.000153 | PRKCG/PRKCB/CDS1/DGKE/PIP5K1B/DGKB/ITPKA/ITPR1/PLCB1/PLCB4/SYNJ1/CALM3/PI4KA/DGKG/INPP5J/INPP5A/CALM1/DGKZ/INPP4B/PIP4K2A/DGKI/ITPK1/PIP4K2C/PIP5K1C/CALM2/PLCD4/INPP4A/PRKCA/PIP4K2B/CDS2/SYNJ2/ITPKB/PIK3R1/DGKQ | | 0.003266 | | | | KEGG |
| KEGG_PROTEASOME | -0.568 | 0.001333 | PSMD8/PSME3/POMP/PSMC3/PSMD6/PSMB9/PSMF1/PSMB8/PSME1/PSMD2/PSMA3/PSMC1/PSMA1/PSMB1/PSME4/PSMB2/PSMA5/PSMB4/PSMB7/PSMD11/PSMD14/PSMD3/PSMB6/PSMA6/PSMD13/PSMA4/PSMD12/PSMC4/PSMC2/PSMB3/PSMA7/PSME2/SEM1/PSMA2 | | 0.011932 | | | | KEGG |
| KEGG_PYRIMIDINE_METABOLISM | -0.40117 | 0.00257 | POLE3/POLR2D/NME1-NME2/RRM1/POLR1B/NME2/POLR3D/CAD/POLR1E/POLA1/ITPA/NME4/POLR2H/POLD3/UCKL1/POLD2/CTPS1/NT5C/CANT1/NT5E/NME1/POLR1C/NT5C3A/PRIM2/PNP/POLD1/POLA2/DPYD/DTYMK/POLE2/POLE/POLR2J2/TYMP/TYMS/RRM2/UPP1/TK1 | | 0.042067 | | | | KEGG |
| KEGG_RNA_DEGRADATION | -0.46304 | 0.002936 | CNOT6/LSM3/XRN1/HSPD1/CNOT1/EXOSC10/ENO1/EXOSC5/MPHOSPH6/ZCCHC7/DCP1A/CNOT2/DCP2/EXOSC7/PAPOLA/LSM1/LSM4/ENO3/EXOSC3/LSM6/SKIV2L/CNOT9/TENT4A/EXOSC2/LSM2/EDC3/EXOSC9/PATL1/LSM8/CNOT3/XRN2/DIS3/EXOSC8/CNOT10/DCPS/EXOSC4/LSM5/LSM7 | | 0.000581 | | | | KEGG |
| KEGG_SPLICEOSOME | -0.50013 | 8.35E-08 | RBM25/RBM8A/TCERG1/PCBP1/PRPF8/SYF2/DDX46/HNRNPK/HNRNPU/DDX5/HNRNPA1/SF3B1/RBM22/SF3A1/PPIL1/PRPF38B/PRPF38A/PPIE/PRPF6/SRSF10/DDX42/HNRNPC/SRSF7/CCDC12/HNRNPA3/DHX15/CRNKL1/LSM4/SNRPE/U2SURP/LSM6/MAGOHB/DHX8/SF3B2/PUF60/THOC2/SNRPF/SF3B5/PRPF40A/CTNNBL1/SNW1/SF3B6/SRSF3/CHERP/SRSF4/SRSF6/MAGOH/SRSF2/TRA2A/THOC1/DHX16/SNRNP200/DDX23/U2AF1/LSM2/PRPF3/HNRNPM/TRA2B/HNRNPA1L2/DDX39B/XAB2/SART1/RBMX/SF3A3/PHF5A/SNRPD1/SNRPC/PPIH/EIF4A3/SF3B4/ACIN1/LSM8/PRPF31/ISY1/SNRPA1/SRSF9/SNRPB2/SNRNP40/SNRPD2/U2AF2/SNRPG/EFTUD2/PRPF4/SF3A2/SNRPA/SNRNP70/BUD31/LSM5/ALYREF/SNRPB/LSM7/HSPA1B/HSPA6 | | 0.001879 | | | | KEGG |
| KEGG_TASTE_TRANSDUCTION | 0.752168 | 0.003784 | CACNA1B/ASIC2/GNG3/PDE1A/KCNB1/PRKACB/CACNA1A/SCNN1B | | 0.023191 | | | | KEGG |
| KEGG_VASCULAR_SMOOTH_MUSCLE_CONTRACTION | 0.547927 | 0.005883 | PRKCG/PRKCB/ADRA1A/ADRA1B/PRKCE/ADCY2/ADCY5/ADCY1/KCNMA1/CACNA1C/GUCY1B1/ITPR1/IRAG1/PLCB1/PLCB4/GUCY1A1/MYLK3/CACNA1D/CALM3/PRKACB/KCNMB2/PLA2G5/PRKG1/CALM1/PRKCQ/MAPK3/GUCY1A2/PPP1R12B/MAP2K1/ADCY9/MYLK/CALM2/KCNMB4/NPR1/PRKCA/PPP1R14A/RAMP3/GNAQ/MAPK1/CALCRL | | 0.032966 | | | | KEGG |
| PID_ATM_PATHWAY | -0.59653 | 0.000752 | RAD9A/TRIM28/ABL1/TOP3A/H2AX/RBBP8/BRCA1/BLM/CDC25A/CHEK2/FANCD2/CDC25C | | 0.023325 | | | | PID |
| PID_ATR_PATHWAY | -0.67353 | 9.26E-06 | RAD9A/TIPIN/RFC4/CDK2/RFC2/MCM2/BRCA2/TIMELESS/CHEK1/RFC3/CDC25A/PLK1/CCNA2/FANCD2/CLSPN/MCM7/CDC6/RAD51/CDC25C | | 0.009207 | | | | PID |
| PID_AURORA_A_PATHWAY | -0.55161 | 0.00606 | TP53/BRCA1/TACC3/AURKA/BIRC5/TPX2/DLGAP5/NFKBIA/CENPA/AURKB | | 0.004668 | | | | PID |
| PID_BARD1_PATHWAY | -0.65549 | 0.000288 | TOPBP1/EWSR1/FANCG/BARD1/CCNE1/TP53/RBBP8/FANCE/BRCA1/CDK2/PCNA/FANCA/FANCC/FANCD2/RAD51 | | 0.009208 | | | | PID |
| PID_E2F_PATHWAY | -0.55068 | 1.08E-05 | HBP1/KAT2A/XRCC1/CCNE2/RB1/TOPBP1/RRM1/CDKN1A/HDAC1/POLA1/RBL1/PRMT5/TRRAP/MYC/TRIM28/DHFR/E2F1/CCNE1/RBBP8/RANBP1/MCM3/MCL1/BRCA1/CDK2/CDKN2C/CDC25A/CCNA2/TYMS/E2F7/CDK1/TP73/CDC6/RRM2/ORC1/E2F2/TK1/PLAU/MYBL2/SERPINE1 | | 0.029683 | | | | PID |
| PID_FOXM1_PATHWAY | -0.70163 | 1.58E-06 | CCNE1/CKS1B/LAMA4/CENPF/CDK2/BRCA2/PLK1/CCNA2/CHEK2/CCNB1/CDK4/CDK1/NEK2/BIRC5/CCNB2/FOXM1/CENPA/ONECUT1/GAS1/AURKB | | 0.008463 | | | | PID |
| PID_LPA4_PATHWAY | 0.736461 | 0.02364 | GNAL/PRKCE/ADCY2/ADCY5/ADCY1/RPS6KA5/LPAR4/ADCY9 | | 0.041882 | | | | PID |
| PID_MYC_ACTIV_PATHWAY | -0.54666 | 3.84E-06 | NME2/RPL11/POLR3D/CAD/MTA1/CCND2/ACTL6A/TRRAP/MYC/PMAIP1/TFRC/RUVBL2/ODC1/GAPDH/NME1/HMGA1/RUVBL1/BAX/TP53/SLC2A1/EIF4A1/RCC1/LDHA/CDCA7/SNAI1/CDC25A/CCNB1/CDK4/BIRC5/PIM1/BCAT1/TK1/FOSL1/TERT/MMP9 | | 0.001424 | | | | PID |
| PID_P73PATHWAY | -0.47946 | 0.000279 | AEN/RELA/MYC/IL4R/RACK1/ABL1/S100A2/BCL2L11/BAX/CASP2/ADA/CDK2/BRCA2/CHEK1/PLK1/CCNA2/GDF15/CCNB1/CDK1/TP73/BUB1/RAD51/SERPINA1/IL1RAP/PLK3/CDK6/SERPINE1 | | 0.045123 | | | | PID |
| PID_RB_1PATHWAY | -0.44645 | 0.005529 | HDAC3/PPP2R3B/SMARCB1/RB1/SPI1/SKP2/RAF1/CDKN1A/HDAC1/CCND2/AATF/RUNX2/DNMT1/SUV39H1/ABL1/E2F1/CCNE1/JUN/CEBPD/CDK2/GSC/CCNA2/CDK4/E2F2/CEBPB/CDK6 | | 0.038187 | | | | PID |
| PID_TELOMERASE_PATHWAY | -0.40277 | 0.012941 | RPS6KB1/SIN3A/SP1/TNKS/ACD/SP3/IFNAR2/TINF2/MRE11/HNRNPC/NCL/XRCC6/SMG6/RBBP4/AKT1/HUS1/PINX1/POT1/TGFB1/IRF1/FOS/HDAC1/DKC1/WRN/NFKB1/SAP30/RAD9A/MYC/ABL1/EGF/E2F1/JUN/BLM/MXD1/TERT/EGFR | | 0.00938 | | | | PID |
| REACTOME_ACTIVATION_OF_ANTERIOR_HOX_GENES_IN_HINDBRAIN_DEVELOPMENT_DURING_EARLY_EMBRYOGENESIS | -0.53288 | 1.47E-06 | RARB/H3-3A/RBBP4/H2BC12/POLR2G/H4C8/CNOT9/POLR2J/AJUBA/HDAC3/H2AC6/H3-3B/POLR2D/SUZ12/HOXA1/H2BC8/POLR2H/WDR5/EGR2/H4C11/PAXIP1/H2AZ1/HOXA4/RARA/H2BC4/H2BC15/H2AX/JUN/MEIS1/H3C6/HOXC4/H2BC5/H2AC20/HOXD4/H2AC8/HOXD3/EZH2/H2BC11/HOXB2/H4C5/H3C10/HOXB4/HOXB3 | | 0.004025 | | | | REACTOME |
| REACTOME_ADRENOCEPTORS | 0.828375 | 0.029403 | ADRA1A/ADRB1/ADRA1B/ADRA2C/ADRB2/ADRA2A | | 0.000827 | | | | REACTOME |
| REACTOME_AMINE_LIGAND_BINDING_RECEPTORS | 0.833416 | 1.5E-06 | CHRM1/HRH3/HTR2A/HTR6/ADRA1A/ADRB1/ADRA1B/DRD1/CHRM4/ADRA2C/GPR143/HTR1D/CHRM3/ADRB2/HTR1B/HRH2/ADRA2A/HTR2B/HTR7 | | 0.01225 | | | | REACTOME |
| REACTOME_APC_C_CDC20_MEDIATED_DEGRADATION_OF_CYCLIN_B | -0.56922 | 0.025972 | UBE2E1/UBC/CDC26/CDC23/ANAPC7/ANAPC2/CDC16/UBA52/ANAPC11/UBE2S/CCNB1/CDK1/CDC20/UBE2C | | 0.000434 | | | | REACTOME |
| REACTOME_APC_C_CDH1_MEDIATED_DEGRADATION_OF_CDC20_AND_OTHER_APC_C_CDH1_TARGETED_PROTEINS_IN_LATE_MITOSIS_EARLY_G1 | -0.55885 | 7.14E-06 | UBE2E1/PSMD2/PSMA3/PSMC1/FZR1/PSMA1/UBC/PSMB1/PSME4/PSMB2/PSMA5/PSMD9/CDC26/PSMB4/CDC23/PSMB7/PSMD11/PSMD14/PSMD3/ANAPC7/ANAPC2/PSMB6/PSMA6/PSMD13/PSMA4/PSMD12/PSMC4/CDC16/PSMD5/PSMC2/PSMB3/RB1/SKP2/UBA52/ANAPC11/PSMA7/PSME2/SEM1/PSMA2/UBE2S/PTTG1/PLK1/AURKA/CDC20/UBE2C/AURKB | | 0.000463 | | | | REACTOME |
| REACTOME_APC_C_MEDIATED_DEGRADATION_OF_CELL_CYCLE_PROTEINS | -0.5221 | 3.58E-06 | UBE2E1/PSMD2/PSMA3/PSMC1/FZR1/PSMA1/UBC/PSMB1/PSME4/PSMB2/PSMA5/PSMD9/CDC26/PSMB4/CDC23/PSMB7/PSMD11/PSMD14/PSMD3/ANAPC7/ANAPC2/PSMB6/PSMA6/PSMD13/PSMA4/PSMD12/PSMC4/CDC16/PSMD5/PSMC2/PSMB3/RB1/SKP2/UBA52/ANAPC11/PSMA7/PSME2/SEM1/PSMA2/FBXO5/UBE2S/CDK2/PTTG1/MAD2L1/PLK1/CCNA2/BUB1B/CCNB1/CDK1/NEK2/AURKA/CDC20/UBE2C/AURKB | | 0.000389 | | | | REACTOME |
| REACTOME_APC_CDC20_MEDIATED_DEGRADATION_OF_NEK2A | -0.59759 | 0.004507 | UBE2E1/UBC/CDC26/CDC23/ANAPC7/ANAPC2/CDC16/UBA52/ANAPC11/UBE2S/MAD2L1/BUB1B/NEK2/CDC20/UBE2C | | 0.000374 | | | | REACTOME |
| REACTOME_ASYMMETRIC_LOCALIZATION_OF_PCP_PROTEINS | -0.44264 | 0.004584 | PSMD6/PSMB9/PSMF1/PSMB8/SMURF2/PSME1/PSMD2/PSMA3/PSMC1/FZD1/PSMA1/UBC/PSMB1/PSME4/SCRIB/PSMB2/PSMA5/PSMD9/PSMB4/FZD3/PSMB7/PSMD11/PSMD14/PSMD3/PSMB6/PSMA6/PSMD13/SMURF1/PSMA4/PSMD12/PSMC4/PSMD5/PSMC2/PSMB3/UBA52/PSMA7/PSME2/SEM1/PSMA2/DVL2/FZD5/VANGL2/FZD7 | | 0.004545 | | | | REACTOME |
| REACTOME_ATF4_ACTIVATES_GENES_IN_RESPONSE_TO_ENDOPLASMIC_RETICULUM_STRESS | -0.6748 | 0.000324 | CEBPG/EXOSC9/ASNS/ATF4/DIS3/EXOSC8/EXOSC4/DDIT3/ATF3/CEBPB/CCL2/IGFBP1/CXCL8 | | 0.003161 | | | | REACTOME |
| REACTOME_ATF6_ATF6_ALPHA_ACTIVATES_CHAPERONE_GENES | -0.80471 | 0.003538 | ATF4/DDIT3/CALR/XBP1/HSP90B1/HSPA5 | | 0.033725 | | | | REACTOME |
| REACTOME_AUF1_HNRNP_D0_BINDS_AND_DESTABILIZES_MRNA | -0.50316 | 0.001268 | PSMD8/PSME3/PSMC3/PSMD6/PSMB9/PSMF1/PABPC1/PSMB8/PSME1/PSMD2/PSMA3/PSMC1/PSMA1/EIF4G1/UBC/PSMB1/PSME4/PSMB2/PSMA5/PSMD9/PSMB4/PSMB7/PSMD11/PSMD14/PSMD3/PSMB6/PSMA6/PSMD13/PSMA4/PSMD12/PSMC4/PSMD5/PSMC2/PSMB3/HSPB1/UBA52/PSMA7/HNRNPD/PSME2/SEM1/PSMA2 | | 0.021028 | | | | REACTOME |
| REACTOME_B_WICH_COMPLEX_POSITIVELY_REGULATES_RRNA_EXPRESSION | -0.49879 | 0.000977 | KAT2A/DDX21/BAZ1B/H2AC6/H3-3B/POLR1B/MYBBP1A/POLR1E/DEK/H2BC8/POLR2H/H4C11/ACTB/H2AZ1/POLR1C/H2BC4/H2BC15/H2AX/H3C6/H2BC5/H2AC20/H2AC8/H2BC11/H4C5/H3C10 | | 0.012523 | | | | REACTOME |
| REACTOME_BASE_EXCISION_REPAIR | -0.53773 | 3.8E-05 | POT1/LIG3/ADPRS/RPA1/XRCC1/H2AC6/APEX1/POLE3/H2BC8/POLD3/H4C11/MUTYH/POLD2/H2AZ1/PNKP/TDG/H2BC4/H2BC15/H2AX/POLD1/RPA3/FEN1/RFC4/NEIL3/H2BC5/PCNA/RFC2/LIG1/H2AC20/POLE2/POLE/H2AC8/RFC3/H2BC11/H4C5 | | 0.0148 | | | | REACTOME |
| REACTOME_BILE_ACID_AND_BILE_SALT_METABOLISM | 0.695606 | 0.001732 | AKR1C2/SLC27A2/AKR1C1/CYP39A1/AKR1C3/CYP46A1/FABP6/CYP7B1/SLCO1A2/OSBPL1A/BAAT | | 7.14E-05 | | | | REACTOME |
| REACTOME_CARDIAC_CONDUCTION | 0.714135 | 6.59E-09 | ATP2B3/CAMK2A/RYR2/SLC8A2/FGF13/SCN2B/SCN3B/KCNJ4/KCNK1/KCNK12/KCNJ12/KCNK4/ATP1A3/KCNK9/SCN9A/SCN2A/KCNIP2/FXYD1/ATP2B2/FGF12/CASQ2/CAMK2B/CACNA2D2/FXYD7/SCN1B/KCNIP4/ATP1B1/SCN4B/NOS1/KCNK3/SCN8A/CACNA1C/CAMK2G/ITPR1/ATP1A2/CACNB2/CACNG8/KCNJ11/NPPC/KCNIP3/SCN5A/RYR3/RYR1/FGF14/SCN7A/CACNB1/CALM1/NPPA/FXYD3/FKBP1B | | 0.007129 | | | | REACTOME |
| REACTOME_CDT1_ASSOCIATION_WITH_THE_CDC6_ORC_ORIGIN_COMPLEX | -0.52486 | 0.000362 | PSMD6/PSMB9/PSMF1/PSMB8/PSME1/PSMD2/PSMA3/PSMC1/PSMA1/UBC/PSMB1/PSME4/PSMB2/PSMA5/PSMD9/PSMB4/PSMB7/PSMD11/PSMD14/PSMD3/PSMB6/PSMA6/PSMD13/PSMA4/PSMD12/PSMC4/PSMD5/PSMC2/PSMB3/ORC5/UBA52/PSMA7/PSME2/SEM1/PSMA2/GMNN/ORC6/MCM8/CDT1/CDC6/ORC1 | | 0.001609 | | | | REACTOME |
| REACTOME_CELLULAR_RESPONSE_TO_HYPOXIA | -0.36947 | 0.03895 | PSMD2/PSMA3/PSMC1/PSMA1/UBC/PSMB1/PSME4/PSMB2/PSMA5/EPAS1/VHL/PSMD9/PSMB4/PSMB7/PSMD11/PSMD14/PSMD3/PSMB6/PSMA6/PSMD13/PSMA4/AJUBA/PSMD12/PSMC4/PSMD5/PSMC2/PSMB3/UBA52/PSMA7/PSME2/SEM1/PSMA2/LIMD1/HIF1A/CA9/VEGFA | | 0.018827 | | | | REACTOME |
| REACTOME_CELLULAR_SENESCENCE | -0.44782 | 1.53E-07 | MAPK7/HMGA1/VENTX/H2BC4/H2BC15/H2AX/E2F1/CCNE1/TP53/ERF/CBX8/JUN/PHC2/H3C6/UBE2S/H2BC5/MAP2K3/CDK2/CBX2/H2AC20/KDM6B/H1-2/H2AC8/CDKN2C/CCNA2/CDK4/EZH2/H2BC11/MDM4/H4C5/H3C10/LMNB1/E2F2/CEBPB/UBE2C/CDK6/IL1A/IL6/CXCL8 | | 0.000117 | | | | REACTOME |
| REACTOME_CGMP_EFFECTS | 0.806894 | 0.002287 | PDE2A/PDE1A/PDE1B/PDE11A/KCNMA1/ITPR1/IRAG1/PDE10A/KCNMB2/PRKG1/PDE5A/KCNMB4 | | 0.002888 | | | | REACTOME |
| REACTOME_CHROMOSOME_MAINTENANCE | -0.62869 | 6.59E-09 | SHQ1/RPA1/H2AC6/TEN1/H3-3B/POLR2D/NOP10/GAR1/DKC1/WRN/POLA1/CENPL/RTEL1/H2BC8/DSCC1/POLR2H/POLD3/CENPQ/H4C11/CENPP/POLD2/H2AZ1/CENPN/DAXX/ITGB3BP/RUVBL2/MIS18A/RUVBL1/H2BC4/H2BC15/H2AX/PRIM2/CENPO/POLD1/RPA3/FEN1/POLA2/CENPX/WRAP53/BLM/RFC4/CHTF18/H2BC5/CDK2/PCNA/RFC2/LIG1/CENPH/H2AC20/H2AC8/RFC3/KNL1/CENPI/CCNA2/H2BC11/CENPM/CENPW/OIP5/PIF1/CENPU/H4C5/CENPK/CENPA/HJURP/TERT | | 0.004773 | | | | REACTOME |
| REACTOME_CONDENSATION_OF_PROPHASE_CHROMOSOMES | -0.62465 | 3.69E-05 | NCAPH2/H2AC6/H3-3B/RB1/SET/SMC2/H2BC8/NCAPD3/H4C11/H2AZ1/H2BC4/H2BC15/H2AX/H3C6/H2BC5/SMC4/H2AC20/NCAPG2/H2AC8/PLK1/CCNB1/CDK1/H2BC11/H4C5/H3C10 | | 0.043415 | | | | REACTOME |
| REACTOME_CROSS_PRESENTATION_OF_SOLUBLE_EXOGENOUS_ANTIGENS_ENDOSOMES | -0.50497 | 0.002185 | CD207/PSMD8/PSME3/PSMC3/PSMD6/PSMB9/PSMF1/PSMB8/PSME1/PSMD2/PSMA3/PSMC1/PSMA1/PSMB1/PSME4/PSMB2/PSMA5/PSMD9/PSMB4/PSMB7/PSMD11/PSMD14/PSMD3/PSMB6/PSMA6/PSMD13/PSMA4/PSMD12/PSMC4/PSMD5/PSMC2/PSMB3/PSMA7/PSME2/SEM1/PSMA2/MRC1/MRC2 | | 0.022373 | | | | REACTOME |
| REACTOME_CYCLIN_A_B1_B2_ASSOCIATED_EVENTS_DURING_G2_M_TRANSITION | -0.7236 | 7.39E-05 | CDK2/PKMYT1/CDC25A/PLK1/CCNA2/CCNB1/CDK1/CCNB2/FOXM1/CDC25C | | 0.029174 | | | | REACTOME |
| REACTOME_CYCLIN_A_CDK2_ASSOCIATED_EVENTS_AT_S_PHASE_ENTRY | -0.41736 | 0.001781 | PSMD2/PSMA3/PSMC1/FZR1/PSMA1/TFDP2/UBC/PSMB1/PSMB2/PSMA5/PSMD9/PSMB4/PSMB7/CDK7/RBBP4/PSMD11/PSMD14/PSMD3/AKT1/PSMB6/PSMA6/E2F4/PSMD13/PSMA4/PSMD12/PSMC4/PSMD5/PSMC2/PSMB3/CCNE2/RB1/WEE1/SKP2/UBA52/LIN37/CDKN1A/PSMA7/PSME2/SEM1/PSMA2/CDC25B/MYC/E2F1/LIN9/CCNE1/CKS1B/PTK6/CDK2/CDC25A/CCNA2/CDK4 | | 0.000908 | | | | REACTOME |
| REACTOME_CYCLIN_D_ASSOCIATED_EVENTS_IN_G1 | -0.43271 | 0.029435 | PPP2R3B/CCNE2/RB1/SKP2/UBA52/CDKN1A/RBL1/CCND2/ABL1/E2F1/CCNE1/CKS1B/PTK6/CDK2/CDKN2C/CDK4/E2F2/CDK6 | | 0.033421 | | | | REACTOME |
| REACTOME_DEADENYLATION_DEPENDENT_MRNA_DECAY | -0.44834 | 0.004562 | CNOT6/LSM3/NT5C3B/XRN1/PAN3/CNOT1/PABPC1/CNOT11/EXOSC5/DCP1A/TUT7/EIF4G1/CNOT2/DCP2/EXOSC7/LSM1/LSM4/EXOSC3/LSM6/SKIV2L/CNOT9/TUT4/PAN2/EXOSC2/TNKS1BP1/LSM2/EDC3/EXOSC9/PATL1/EIF4A3/CNOT3/DIS3/EXOSC8/CNOT10/DCPS/EXOSC4/LSM5/EIF4A1/LSM7 | | 0.001838 | | | | REACTOME |
| REACTOME_DECTIN_1_MEDIATED_NONCANONICAL_NF_KB_SIGNALING | -0.42446 | 0.013312 | PSMD6/PSMB9/PSMF1/PSMB8/CUL1/PSME1/PSMD2/PSMA3/PSMC1/PSMA1/UBC/PSMB1/PSME4/PSMB2/PSMA5/PSMD9/PSMB4/PSMB7/PSMD11/PSMD14/PSMD3/PSMB6/PSMA6/PSMD13/PSMA4/PSMD12/PSMC4/PSMD5/PSMC2/PSMB3/UBA52/PSMA7/MAP3K14/PSME2/SEM1/PSMA2/RELA/NFKB2/RELB | | 0.015541 | | | | REACTOME |
| REACTOME_DEFECTIVE_CFTR_CAUSES_CYSTIC_FIBROSIS | -0.47108 | 0.001937 | DERL1/ERLIN2/RPS27A/RNF185/SEL1L/PSMD8/PSME3/PSMC3/PSMD6/PSMB9/PSMF1/PSMB8/PSME1/PSMD2/PSMA3/PSMC1/ERLIN1/PSMA1/UBC/PSMB1/PSME4/PSMB2/PSMA5/PSMD9/PSMB4/PSMB7/PSMD11/PSMD14/PSMD3/PSMB6/PSMA6/PSMD13/PSMA4/PSMD12/PSMC4/PSMD5/PSMC2/PSMB3/UBA52/PSMA7/PSME2/SEM1/PSMA2/OS9/DERL2/DERL3 | | 0.017442 | | | | REACTOME |
| REACTOME_DEGRADATION_OF_AXIN | -0.42659 | 0.01738 | PSMD8/PSME3/PSMC3/PSMD6/PSMB9/PSMF1/PSMB8/SMURF2/TNKS/PSME1/PSMD2/PSMA3/PSMC1/PSMA1/UBC/PSMB1/PSME4/PSMB2/PSMA5/PSMD9/PSMB4/PSMB7/PSMD11/PSMD14/PSMD3/PSMB6/PSMA6/AXIN1/PSMD13/PSMA4/PSMD12/PSMC4/PSMD5/PSMC2/PSMB3/UBA52/PSMA7/PSME2/SEM1/PSMA2 | | 0.017842 | | | | REACTOME |
| REACTOME_DEPOSITION_OF_NEW_CENPA_CONTAINING_NUCLEOSOMES_AT_THE_CENTROMERE | -0.72278 | 6.59E-09 | CENPL/H2BC8/CENPQ/H4C11/CENPP/H2AZ1/CENPN/ITGB3BP/MIS18A/RUVBL1/H2BC4/H2BC15/H2AX/CENPO/CENPX/H2BC5/CENPH/H2AC20/H2AC8/KNL1/CENPI/H2BC11/CENPM/CENPW/OIP5/CENPU/H4C5/CENPK/CENPA/HJURP | | 0.049164 | | | | REACTOME |
| REACTOME_DISEASES_ASSOCIATED_WITH_N_GLYCOSYLATION_OF_PROTEINS | -0.63189 | 0.021065 | ALG11/ALG1/ALG12/B4GALT1/MGAT2/ALG9/DPAGT1/ALG8/RFT1/ALG2/MPDU1/MOGS/MAN1B1/ALG3 | | 0.011439 | | | | REACTOME |
| REACTOME_DISEASES_OF_MITOTIC_CELL_CYCLE | -0.52066 | 0.006379 | UBE2E1/FZR1/TFDP2/CDC26/CDC23/ANAPC7/ANAPC2/CDC16/CCNE2/RB1/SKP2/ANAPC11/CDKN1A/CCND2/DAXX/E2F1/CCNE1/UBE2S/CDK2/CDK4/E2F2/UBE2C/CDK6 | | 0.003248 | | | | REACTOME |
| REACTOME_DISEASES_OF_PROGRAMMED_CELL_DEATH | -0.54562 | 2.78E-05 | H2AC6/H3-3B/RIPK1/FADD/SUZ12/TRADD/TRAF2/POLA1/H2BC8/CDC25B/H4C11/DNMT1/H2AZ1/LMNA/H2BC4/GSDME/H2BC15/H2AX/BCL2L11/PRIM2/TP53/DNMT3B/JUN/POLA2/H3C6/SOD2/H2BC5/H2AC20/H2AC8/CDC25A/EZH2/H2BC11/H4C5/H3C10/LMNB1/CDC25C | | 0.018951 | | | | REACTOME |
| REACTOME_DNA_DAMAGE_BYPASS | -0.55894 | 0.000259 | USP1/POLI/RPA1/POLE3/UBA52/POLH/UFD1/POLD3/POLD2/POLD1/RPA3/RFC4/RAD18/PCNA/RFC2/MAD2L2/POLE2/POLE/RFC3/DTL/PCLAF | | 0.024762 | | | | REACTOME |
| REACTOME_DNA_DOUBLE_STRAND_BREAK_REPAIR | -0.5433 | 6.59E-09 | H2BC12/ATRIP/H4C8/NSD2/MDC1/HUS1/RMI1/MUS81/RMI2/XRCC4/LIG3/DNA2/EYA2/UIMC1/RPA1/XRCC1/BAZ1B/POLE3/UBA52/TOPBP1/POLH/EYA1/WRN/BARD1/RHNO1/RTEL1/H2BC8/KDM4B/TDP1/POLD3/SLX1A/SIRT6/H4C11/PAXIP1/GEN1/POLD2/RAD9A/ABL1/H2BC4/TOP3A/H2BC15/TIPIN/H2AX/TP53/RBBP8/POLM/PPP4C/KPNA2/POLD1/RPA3/FEN1/BRCA1/BLM/RFC4/H2BC5/CDK2/PCNA/RFC2/BRCA2/TIMELESS/CHEK1/POLE2/POLE/RAD51AP1/RFC3/CCNA2/CHEK2/H2BC11/XRCC2/CLSPN/BRIP1/H4C5/RAD51/EXO1/POLQ/EYA4/EME1 | | 0.000897 | | | | REACTOME |
| REACTOME_DNA_DOUBLE_STRAND_BREAK_RESPONSE | -0.41396 | 0.017039 | PIAS4/H2BC12/H4C8/NSD2/MDC1/EYA2/UIMC1/BAZ1B/UBA52/EYA1/BARD1/H2BC8/KDM4B/H4C11/ABL1/H2BC4/H2BC15/H2AX/TP53/KPNA2/BRCA1/H2BC5/CHEK2/H2BC11/H4C5/EYA4 | | 0.018372 | | | | REACTOME |
| REACTOME_DNA_REPLICATION | -0.57448 | 6.59E-09 | MCM4/POLD4/PSMB2/PSMA5/PSMD9/CDC26/KPNB1/PSMB4/H3-3A/CDC23/PSMB7/PSMD11/PSMD14/PSMD3/H2BC12/ANAPC7/H4C8/ANAPC2/PSMB6/PSMA6/PSMD13/PRIM1/PSMA4/PSMD12/PSMC4/CDC16/DNA2/PSMD5/PSMC2/RPA1/H2AC6/PSMB3/POLE3/H3-3B/CCNE2/ORC5/SKP2/UBA52/ANAPC11/PSMA7/PSME2/POLA1/SEM1/H2BC8/POLD3/PSMA2/H4C11/MCM6/POLD2/CDC7/H2AZ1/GINS3/H2BC4/GMNN/H2BC15/H2AX/E2F1/PRIM2/CCNE1/MCM3/POLD1/RPA3/FEN1/POLA2/H3C6/RFC4/ORC6/UBE2S/MCM5/H2BC5/CDK2/MCM8/PCNA/RFC2/MCM2/LIG1/H2AC20/POLE2/POLE/GINS4/H2AC8/RFC3/GINS2/CDT1/CCNA2/H2BC11/GINS1/MCM7/DBF4/H4C5/CDC6/H3C10/MCM10/ORC1/E2F2/UBE2C/CDC45 | | 0.000528 | | | | REACTOME |
| REACTOME_DNA_REPLICATION_PRE_INITIATION | -0.57025 | 6.59E-09 | PSMD2/PSMA3/PSMC1/PSMA1/UBC/PSMB1/PSME4/MCM4/PSMB2/PSMA5/PSMD9/KPNB1/PSMB4/H3-3A/PSMB7/PSMD11/PSMD14/PSMD3/H2BC12/H4C8/PSMB6/PSMA6/PSMD13/PRIM1/PSMA4/PSMD12/PSMC4/PSMD5/PSMC2/RPA1/H2AC6/PSMB3/POLE3/H3-3B/ORC5/UBA52/PSMA7/PSME2/POLA1/SEM1/H2BC8/PSMA2/H4C11/MCM6/CDC7/H2AZ1/H2BC4/GMNN/H2BC15/H2AX/E2F1/PRIM2/MCM3/RPA3/POLA2/H3C6/ORC6/MCM5/H2BC5/CDK2/MCM8/MCM2/H2AC20/POLE2/POLE/H2AC8/CDT1/H2BC11/MCM7/DBF4/H4C5/CDC6/H3C10/MCM10/ORC1/E2F2/CDC45 | | 0.001122 | | | | REACTOME |
| REACTOME_DUAL_INCISION_IN_GG_NER | -0.4959 | 0.015306 | GTF2H3/RFC5/UBC/ERCC1/CUL4A/GTF2H4/POLD4/GTF2H2/RPA1/POLE3/UBA52/CHD1L/POLD3/POLD2/DDB2/POLD1/RPA3/RFC4/PCNA/RFC2/POLE2/POLE/RFC3 | | 0.005674 | | | | REACTOME |
| REACTOME_DUAL_INCISION_IN_TC_NER | -0.44857 | 0.002757 | GTF2H3/ZNF830/RFC5/PPIE/UBC/ERCC1/CUL4A/GTF2H4/POLD4/TCEA1/GTF2H2/CDK7/POLR2G/HMGN1/POLR2J/RPA1/POLE3/POLR2D/UBA52/XAB2/ISY1/POLR2H/POLD3/POLD2/POLD1/RPA3/RFC4/PCNA/RFC2/POLE2/POLE/RFC3 | | 0.006786 | | | | REACTOME |
| REACTOME_EPIGENETIC_REGULATION_OF_GENE_EXPRESSION | -0.43923 | 6.97E-05 | TAF1D/SF3B1/POLR2F/PHF1/TAF1B/GTF2H3/TET3/MYO1C/TAF1C/POLR1A/MTF2/GTF2H4/RRP8/SAP30BP/TET2/JARID2/GTF2H2/H3-3A/CDK7/RBBP4/H2BC12/H4C8/TAF1A/MBD2/DNMT3A/GATAD2A/TTF1/KAT2A/DDX21/BAZ1B/H2AC6/H3-3B/SUZ12/MBD3/POLR1B/MTA2/HDAC1/MYBBP1A/POLR1E/DEK/MTA1/H2BC8/POLR2H/H4C11/ACTB/DNMT1/SAP30/SUV39H1/H2AZ1/TDG/POLR1C/H2BC4/H2BC15/H2AX/CBX3/DNMT3B/PHF19/H3C6/H2BC5/H2AC20/H2AC8/EZH2/H2BC11/H4C5/H3C10/UHRF1 | | 0.005322 | | | | REACTOME |
| REACTOME_EXPORT_OF_VIRAL_RIBONUCLEOPROTEINS_FROM_NUCLEUS | -0.62056 | 0.000432 | NUP42/NUP155/NUP43/NUP160/NUP153/NUP58/RAN/NUP88/NUP210/NUP98/RAE1/POM121/AAAS/NUP54/NUP35/NUP62/SEC13/POM121C/NDC1/NUP85/NUP188/NUP205/NUP37/NUP107 | | 0.01583 | | | | REACTOME |
| REACTOME_EXTENSION_OF_TELOMERES | -0.6269 | 3.34E-06 | POT1/PRIM1/DNA2/SHQ1/RPA1/TEN1/NOP10/GAR1/DKC1/WRN/POLA1/RTEL1/DSCC1/POLD3/POLD2/RUVBL2/RUVBL1/PRIM2/POLD1/RPA3/FEN1/POLA2/WRAP53/BLM/RFC4/CHTF18/CDK2/PCNA/RFC2/LIG1/RFC3/CCNA2/PIF1/TERT | | 0.006485 | | | | REACTOME |
| REACTOME_FANCONI_ANEMIA_PATHWAY | -0.55766 | 0.001628 | FANCM/UBC/ERCC1/SLX4/CENPS/ATRIP/MUS81/USP1/FAAP20/RPA1/UBA52/FANCG/SLX1A/DCLRE1B/FAAP100/FANCE/FANCB/RPA3/CENPX/UBE2T/FANCA/FANCC/FANCI/FAAP24/FANCD2/EME1 | | 0.0357 | | | | REACTOME |
| REACTOME_FGFR2C_LIGAND_BINDING_AND_ACTIVATION | 0.88284 | 0.015934 | FGF9/FGF17/FGFR2/FGF1/FGF20 | | 0.005141 | | | | REACTOME |
| REACTOME_FGFR3_LIGAND_BINDING_AND_ACTIVATION | 0.838531 | 0.041941 | FGF9/FGF17/FGF1/FGF20/FGFR3 | | 0.024762 | | | | REACTOME |
| REACTOME_G1_S_DNA_DAMAGE_CHECKPOINTS | -0.47014 | 0.001114 | PSMD2/PSMA3/PSMC1/PSMA1/UBC/PSMB1/MDM2/PSME4/PSMB2/PSMA5/PSMD9/PSMB4/PSMB7/PSMD11/PSMD14/PSMD3/PSMB6/PSMA6/PSMD13/PSMA4/PSMD12/PSMC4/PSMD5/PSMC2/PSMB3/CCNE2/UBA52/CDKN1A/PSMA7/PSME2/SEM1/PSMA2/CCNE1/TP53/CDK2/CHEK1/CDC25A/CCNA2/CHEK2/MDM4 | | 0.002972 | | | | REACTOME |
| REACTOME_G2_M_CHECKPOINTS | -0.53044 | 6.59E-09 | PIAS4/PSMD11/PSMD14/PSMD3/H2BC12/ATRIP/H4C8/NSD2/MDC1/HUS1/RMI1/PSMB6/PSMA6/RMI2/PSMD13/PSMA4/PSMD12/PSMC4/DNA2/PSMD5/UIMC1/PSMC2/RPA1/PSMB3/ORC5/WEE1/UBA52/TOPBP1/PSMA7/PSME2/WRN/BARD1/SEM1/RHNO1/H2BC8/PSMA2/H4C11/MCM6/CDC7/RAD9A/H2BC4/TOP3A/H2BC15/H2AX/TP53/RBBP8/MCM3/RPA3/BRCA1/BLM/RFC4/ORC6/MCM5/H2BC5/CDK2/MCM8/RFC2/MCM2/PKMYT1/CHEK1/RFC3/CDC25A/CHEK2/CCNB1/CDK1/H2BC11/CLSPN/MCM7/BRIP1/DBF4/CCNB2/H4C5/GTSE1/CDC6/EXO1/MCM10/ORC1/CDC25C/CDC45 | | 0.00026 | | | | REACTOME |
| REACTOME_G2_M_DNA_DAMAGE_CHECKPOINT | -0.5069 | 4.24E-05 | PIAS4/H2BC12/ATRIP/H4C8/NSD2/MDC1/HUS1/RMI1/RMI2/DNA2/UIMC1/RPA1/WEE1/TOPBP1/WRN/BARD1/RHNO1/H2BC8/H4C11/RAD9A/H2BC4/TOP3A/H2BC15/H2AX/TP53/RBBP8/RPA3/BRCA1/BLM/RFC4/H2BC5/RFC2/CHEK1/RFC3/CHEK2/CCNB1/CDK1/H2BC11/BRIP1/H4C5/EXO1/CDC25C | | 0.010405 | | | | REACTOME |
| REACTOME_GABA_B_RECEPTOR_ACTIVATION | 0.702125 | 0.000681 | KCNJ3/GNG3/KCNJ4/GNAL/KCNJ12/GABBR1/GABBR2/KCNJ9/KCNJ6/ADCY2/ADCY5/ADCY1/KCNJ10/GNB5/GNAI1 | | 0.027398 | | | | REACTOME |
| REACTOME_GABA_RECEPTOR_ACTIVATION | 0.779681 | 9.11E-09 | GABRB2/GABRA1/GABRG2/KCNJ3/GABRA4/GABRA5/GNG3/KCNJ4/GABRA2/GNAL/GABRB3/KCNJ12/GABRB1/GABBR1/GABBR2/GABRA3/KCNJ9/KCNJ6/ADCY2/ADCY5/ADCY1/KCNJ10/GNB5/ARHGEF9/GNAI1 | | 0.004415 | | | | REACTOME |
| REACTOME_GAP_FILLING_DNA_REPAIR_SYNTHESIS_AND_LIGATION_IN_GG_NER | -0.6586 | 0.001223 | LIG3/RPA1/XRCC1/POLE3/UBA52/POLD3/POLD2/POLD1/RPA3/RFC4/PCNA/RFC2/LIG1/POLE2/POLE/RFC3 | | 0.007129 | | | | REACTOME |
| REACTOME_GENE_SILENCING_BY_RNA | -0.3773 | 0.006467 | BCDIN3D/MYBL1/NUP42/AGO2/NUP155/NUP43/H3-3A/NUP160/NUP153/H2BC12/POLR2G/H4C8/NUP58/RAN/NUP88/POLR2J/NUP210/NUP98/RAE1/H2AC6/POM121/H3-3B/POLR2D/AAAS/DGCR8/NUP54/NUP35/NUP62/H2BC8/SEC13/POM121C/POLR2H/NDC1/H4C11/TARBP2/NUP85/H2AZ1/ANG/NUP188/NUP205/XPO5/NUP37/H2BC4/H2BC15/H2AX/NUP107/PIWIL2/H3C6/H2BC5/H2AC20/H2AC8/H2BC11/H4C5/H3C10 | | 0.013186 | | | | REACTOME |
| REACTOME_GLOBAL_GENOME_NUCLEOTIDE_EXCISION_REPAIR_GG_NER | -0.3956 | 0.002901 | GTF2H3/RFC5/UBE2I/PIAS3/UBC/ERCC1/MCRS1/CUL4A/GTF2H4/POLD4/COPS3/COPS6/GTF2H2/NFRKB/CDK7/ACTR5/CETN2/LIG3/RPA1/XRCC1/POLE3/UBA52/CHD1L/POLD3/ACTB/ACTL6A/POLD2/RUVBL1/DDB2/POLD1/RPA3/RFC4/PCNA/RFC2/LIG1/POLE2/POLE/RFC3 | | 0.00319 | | | | REACTOME |
| REACTOME_GLUTAMATE_NEUROTRANSMITTER_RELEASE_CYCLE | 0.850032 | 6.22E-06 | SLC17A7/SYT1/GLS2/SNAP25/RAB3A/CPLX1/RIMS1/PPFIA2/STXBP1/TSPOAP1/SLC1A2/STX1A/SLC1A1/PPFIA3/VAMP2/GLS/SLC1A7/PPFIA4 | | 0.043254 | | | | REACTOME |
| REACTOME_HCMV_EARLY_EVENTS | -0.35673 | 0.019568 | NUP160/RBBP4/NUP153/H2BC12/H4C8/TUBB2B/NUP58/NUP88/TUBB1/NUP210/NUP98/HDAC3/RAE1/H2AC6/GPS2/POM121/SUZ12/TUBB4B/AAAS/NCOR2/NUP54/NUP35/NUP62/H2BC8/SEC13/POM121C/NFKB1/NDC1/H4C11/NUP85/NUP188/NUP205/DAXX/H2AC11/ITGB1/TRIM28/NUP37/H2BC4/H2BC15/NUP107/H3C6/H2BC5/H2AC20/H2AC8/TUBA1C/EZH2/H2BC11/TUBB6/H4C5/H3C10/EGFR | | 0.010127 | | | | REACTOME |
| REACTOME_HCMV_INFECTION | -0.33807 | 0.007952 | NUP210/NUP98/HDAC3/RAE1/H2AC6/GPS2/POM121/SUZ12/TUBB4B/AAAS/MVB12A/NCOR2/NUP54/NUP35/VPS25/NUP62/H2BC8/SEC13/POM121C/NFKB1/NDC1/H4C11/NUP85/NUP188/NUP205/DAXX/H2AC11/ITGB1/TRIM28/NUP37/H2BC4/VPS37B/H2BC15/NUP107/CEBPD/H3C6/H2BC5/H2AC20/H2AC8/TUBA1C/EZH2/H2BC11/TUBB6/H4C5/H3C10/EGFR | | 0.001394 | | | | REACTOME |
| REACTOME_HCMV_LATE_EVENTS | -0.50273 | 6.82E-05 | VPS37C/NUP42/H2BC18/NUP155/NUP43/CHMP2A/CHMP6/NUP160/NUP153/H2BC12/H4C8/NUP58/NUP88/NUP210/NUP98/RAE1/H2AC6/POM121/AAAS/MVB12A/NUP54/NUP35/VPS25/NUP62/H2BC8/SEC13/POM121C/NDC1/H4C11/NUP85/NUP188/NUP205/H2AC11/NUP37/H2BC4/VPS37B/H2BC15/NUP107/CEBPD/H3C6/H2BC5/H2AC20/H2AC8/H2BC11/H4C5/H3C10 | | 0.002756 | | | | REACTOME |
| REACTOME_HDR_THROUGH_HOMOLOGOUS_RECOMBINATION_HRR | -0.63514 | 4.1E-08 | HUS1/RMI1/MUS81/RMI2/DNA2/RPA1/POLE3/UBA52/TOPBP1/POLH/WRN/BARD1/RHNO1/RTEL1/POLD3/SLX1A/GEN1/POLD2/RAD9A/TOP3A/RBBP8/POLD1/RPA3/BRCA1/BLM/RFC4/PCNA/RFC2/BRCA2/CHEK1/POLE2/POLE/RAD51AP1/RFC3/XRCC2/BRIP1/RAD51/EXO1/EME1 | | 0.001954 | | | | REACTOME |
| REACTOME_HDR_THROUGH_SINGLE_STRAND_ANNEALING_SSA | -0.6408 | 0.000106 | RAD52/ATRIP/HUS1/RMI1/RMI2/DNA2/RPA1/TOPBP1/WRN/BARD1/RHNO1/RAD9A/ABL1/TOP3A/RBBP8/RPA3/BRCA1/BLM/RFC4/RFC2/RFC3/BRIP1/RAD51/EXO1 | | 0.026939 | | | | REACTOME |
| REACTOME_HIV_ELONGATION_ARREST_AND_RECOVERY | -0.53665 | 0.011129 | CCNT1/POLR2F/GTF2F1/SUPT16H/NELFB/GTF2F2/TCEA1/POLR2G/POLR2J/NELFCD/CCNK/ELOA/CTDP1/POLR2D/NELFE/POLR2H/SSRP1/ELL | | 0.004702 | | | | REACTOME |
| REACTOME_HIV_LIFE_CYCLE | -0.39642 | 6.94E-05 | TAF13/GTF2A2/NUP50/TAF5/CCNT1/POLR2F/GTF2F1/TAF10/SUPT16H/NUP214/GTF2H3/PDCD6IP/TAF2/NELFB/VPS37D/UBC/VPS37C/GTF2H4/NUP42/XRCC6/NUP155/NUP43/GTF2F2/TCEA1/GTF2H2/CHMP2A/TAF4/CHMP6/CDK7/NUP160/NUP153/POLR2G/TAF11/TAF6/NUP58/RAN/GTF2E2/PPIA/NUP88/POLR2J/CCR5/TAF7/XRCC4/NELFCD/NUP210/CCNK/NUP98/ELOA/BANF1/RAE1/CTDP1/POM121/POLR2D/UBA52/NELFE/AAAS/MVB12A/FURIN/NUP54/NUP35/NUP62/SEC13/POM121C/POLR2H/NDC1/NUP85/NUP188/NUP205/SSRP1/HMGA1/NUP37/VPS37B/RANBP1/NUP107/FEN1/ELL/LIG1/CXCR4/RCC1 | | 0.001649 | | | | REACTOME |
| REACTOME_HIV_TRANSCRIPTION_ELONGATION | -0.42945 | 0.034898 | SUPT5H/NCBP2/ERCC2/MNAT1/CCNT1/POLR2F/GTF2F1/SUPT16H/GTF2H3/NELFB/GTF2H4/GTF2F2/TCEA1/GTF2H2/CDK7/POLR2G/POLR2J/NELFCD/ELOA/CTDP1/POLR2D/NELFE/POLR2H/SSRP1/ELL | | 0.005845 | | | | REACTOME |
| REACTOME_HOMOLOGOUS_DNA_PAIRING_AND_STRAND_EXCHANGE | -0.65917 | 3.28E-06 | ATRIP/HUS1/RMI1/RMI2/DNA2/RPA1/TOPBP1/WRN/BARD1/RHNO1/RAD9A/TOP3A/RBBP8/RPA3/BRCA1/BLM/RFC4/RFC2/BRCA2/CHEK1/RAD51AP1/RFC3/XRCC2/BRIP1/RAD51/EXO1 | | 0.040987 | | | | REACTOME |
| REACTOME_HOMOLOGY_DIRECTED_REPAIR | -0.57274 | 6.59E-09 | H2BC12/ATRIP/H4C8/NSD2/MDC1/HUS1/RMI1/MUS81/RMI2/LIG3/DNA2/UIMC1/RPA1/XRCC1/POLE3/UBA52/TOPBP1/POLH/WRN/BARD1/RHNO1/RTEL1/H2BC8/POLD3/SLX1A/SIRT6/H4C11/GEN1/POLD2/RAD9A/ABL1/H2BC4/TOP3A/H2BC15/TIPIN/H2AX/RBBP8/PPP4C/POLD1/RPA3/FEN1/BRCA1/BLM/RFC4/H2BC5/CDK2/PCNA/RFC2/BRCA2/TIMELESS/CHEK1/POLE2/POLE/RAD51AP1/RFC3/CCNA2/H2BC11/XRCC2/CLSPN/BRIP1/H4C5/RAD51/EXO1/POLQ/EME1 | | 0.000716 | | | | REACTOME |
| REACTOME_HOST_INTERACTIONS_OF_HIV_FACTORS | -0.31515 | 0.037827 | NUP50/PAK2/CCNT1/PSME1/NUP214/PSMD2/PSMA3/PSMC1/PSMA1/UBC/PSMB1/PSME4/NUP42/PSMB2/PSMA5/PSMD9/AP2S1/KPNB1/PSMB4/NUP155/NUP43/PSMB7/NUP160/NUP153/PSMD11/PSMD14/PSMD3/AP1S2/RAC1/NPM1/NUP58/RAN/PSMB6/PPIA/PSMA6/HCK/NUP88/PSMD13/PSMA4/PSMD12/PSMC4/NUP210/NUP98/BANF1/PSMD5/RAE1/PSMC2/PSMB3/POM121/UBA52/AAAS/NUP54/PSMA7/NUP35/PSME2/NUP62/SEM1/SEC13/POM121C/NDC1/PSMA2/NUP85/NUP188/NUP205/HLA-A/HMGA1/NUP37/RANBP1/NUP107/RCC1 | | 0.018827 | | | | REACTOME |
| REACTOME_INHIBITION_OF_THE_PROTEOLYTIC_ACTIVITY_OF_APC_C_REQUIRED_FOR_THE_ONSET_OF_ANAPHASE_BY_MITOTIC_SPINDLE_CHECKPOINT_COMPONENTS | -0.62484 | 0.010234 | CDC26/CDC23/ANAPC7/ANAPC2/CDC16/ANAPC11/UBE2S/MAD2L1/BUB1B/CDC20/UBE2C | | 0.000742 | | | | REACTOME |
| REACTOME_INITIATION_OF_NUCLEAR_ENVELOPE_NE_REFORMATION | -0.65807 | 0.009759 | LEMD2/LBR/ANKLE2/LMNA/TMPO/VRK1/CCNB1/CDK1/CCNB2/LMNB1 | | 0.010135 | | | | REACTOME |
| REACTOME_INOSITOL_PHOSPHATE_METABOLISM | 0.57736 | 0.03943 | MTMR7/ITPKA/PLCH1/PLCH2/PLCB1/PLCB4/SYNJ1/INPP5J/INPP5A/CALM1/PPIP5K1/NUDT11/INPP4B/ITPK1/PLCD4/INPP4A/MTMR9/NUDT3/IP6K3/ITPKB/NUDT10 | | 0.007721 | | | | REACTOME |
| REACTOME_INTERACTIONS_OF_REV_WITH_HOST_CELLULAR_PROTEINS | -0.62206 | 0.000231 | NUP42/KPNB1/NUP155/NUP43/NUP160/NUP153/NPM1/NUP58/RAN/NUP88/NUP210/NUP98/RAE1/POM121/AAAS/NUP54/NUP35/NUP62/SEC13/POM121C/NDC1/NUP85/NUP188/NUP205/NUP37/RANBP1/NUP107/RCC1 | | 0.006063 | | | | REACTOME |
| REACTOME_INTERLEUKIN_1_SIGNALING | -0.41714 | 0.000856 | PSMD2/PSMA3/PSMC1/SQSTM1/AGER/PSMA1/UBC/PSMB1/PSME4/PSMB2/PSMA5/PSMD9/PSMB4/PSMB7/PSMD11/PSMD14/PSMD3/HMGB1/PSMB6/PSMA6/PSMD13/PSMA4/PSMD12/PSMC4/NFKBIB/PELI1/PSMD5/PSMC2/PSMB3/MYD88/IRAK2/UBA52/IRAK1/PSMA7/PSME2/SEM1/IL1R1/NFKB1/TNIP2/PSMA2/RELA/NOD1/NFKB2/MAP3K8/NKIRAS2/RIPK2/S100A12/NOD2/NFKBIA/IL1RAP/IL1A/IL1B/IL1R2/IL1RN | | 0.033725 | | | | REACTOME |
| REACTOME_ION_CHANNEL_TRANSPORT | 0.582896 | 1.39E-05 | ATP2B3/CAMK2A/ATP8A2/RYR2/ASIC2/ANO3/ATP1A3/ATP6V1G2/FXYD1/ATP2B2/TRPV6/ANO5/CASQ2/CLCA4/CAMK2B/WNK2/FXYD7/ANO4/ATP8A1/ATP1B1/UNC80/CLCN4/NALCN/UNC79/CAMK2G/ATP1A2/TRPC3/TRPM3/RYR3/RYR1/ATP6V1H/CALM1/ATP6V1A/FXYD3/FKBP1B/SCNN1B/ATP9A/ATP2C2/TRPM6/TRPV3/ATP6V0A1/ATP7B/ANO2/SGK2/ATP10A/SLC9B2/ATP6V1C1 | | 0.000709 | | | | REACTOME |
| REACTOME_ION_HOMEOSTASIS | 0.703555 | 4.37E-05 | ATP2B3/CAMK2A/RYR2/SLC8A2/ATP1A3/FXYD1/ATP2B2/CASQ2/CAMK2B/FXYD7/ATP1B1/NOS1/CAMK2G/ITPR1/ATP1A2/KCNJ11/RYR3/RYR1/CALM1/FXYD3/FKBP1B/SLC8A3 | | 0.030578 | | | | REACTOME |
| REACTOME_ION_TRANSPORT_BY_P_TYPE_ATPASES | 0.675095 | 0.000307 | ATP2B3/CAMK2A/ATP8A2/ATP1A3/FXYD1/ATP2B2/CAMK2B/FXYD7/ATP8A1/ATP1B1/CAMK2G/ATP1A2/CALM1/FXYD3/ATP9A/ATP2C2/ATP7B/ATP10A | | 0.019447 | | | | REACTOME |
| REACTOME_MEIOSIS | -0.52041 | 3.61E-05 | SUN1/H2BC8/H4C11/H2AZ1/LMNA/H2BC4/TOP3A/H2BC15/H2AX/RBBP8/SYCE2/RPA3/H3C6/BRCA1/BLM/H2BC5/CDK2/MSH5/PSMC3IP/BRCA2/H2AC20/H2AC8/CDK4/H2BC11/H4C5/H3C10/RAD51/LMNB1/MND1 | | 0.02755 | | | | REACTOME |
| REACTOME_MEIOTIC_RECOMBINATION | -0.66186 | 4.96E-07 | H2BC8/H4C11/H2AZ1/H2BC4/TOP3A/H2BC15/H2AX/RBBP8/RPA3/H3C6/BRCA1/BLM/H2BC5/CDK2/MSH5/PSMC3IP/BRCA2/H2AC20/H2AC8/CDK4/H2BC11/H4C5/H3C10/RAD51/MND1 | | 0.016397 | | | | REACTOME |
| REACTOME_METABOLISM_OF_AMINE_DERIVED_HORMONES | 0.879134 | 0.018743 | PNMT/DIO2/DIO3/DBH/DUOX1 | | 0.012437 | | | | REACTOME |
| REACTOME_MITOTIC_G1_PHASE_AND_G1_S_TRANSITION | -0.52195 | 6.59E-09 | E2F6/E2F4/PSMD13/PRIM1/PSMA4/PSMD12/PSMC4/PSMD5/PPP2R3B/PSMC2/RPA1/PSMB3/POLE3/CCNE2/ORC5/RB1/WEE1/SKP2/UBA52/LIN37/CDKN1A/PSMA7/HDAC1/PSME2/POLA1/RBL1/SEM1/CCND2/PSMA2/MCM6/CDC7/MYC/ABL1/DHFR/GMNN/E2F1/LIN9/PRIM2/CCNE1/CKS1B/MCM3/RPA3/POLA2/FBXO5/PTK6/ORC6/MCM5/CDK2/MCM8/PCNA/MCM2/POLE2/POLE/CDT1/CDKN2C/CDC25A/CCNA2/TYMS/CCNB1/CDK4/CDK1/TOP2A/MCM7/DBF4/CDC6/MCM10/RRM2/ORC1/E2F2/TK1/CDC45/CDK6/MYBL2 | | 0.002234 | | | | REACTOME |
| REACTOME_MITOTIC_PROPHASE | -0.39577 | 0.000708 | NUP210/NUP98/NCAPH2/BANF1/RAE1/H2AC6/POM121/H3-3B/RB1/SET/AAAS/CTDNEP1/MASTL/SMC2/NUP54/NUP35/NUP62/LEMD2/H2BC8/SEC13/POM121C/NDC1/NCAPD3/NEK6/H4C11/NUP85/H2AZ1/NUP188/NUP205/LMNA/NUP37/TMPO/H2BC4/H2BC15/H2AX/NUP107/VRK1/H3C6/H2BC5/SMC4/H2AC20/NCAPG2/H2AC8/PLK1/CCNB1/CDK1/H2BC11/CCNB2/H4C5/H3C10/LMNB1 | | 0.011865 | | | | REACTOME |
| REACTOME_MITOTIC_SPINDLE_CHECKPOINT | -0.62238 | 6.59E-09 | RCC2/CENPL/SEC13/DSN1/INCENP/CENPQ/B9D2/CENPP/SPDL1/NUP85/CENPN/MIS12/ITGB3BP/MAD1L1/NDE1/NUP37/NUP107/CENPO/ZWILCH/SPC25/CENPF/KNTC1/UBE2S/CENPH/SGO2/MAD2L1/KIF18A/KNL1/ZWINT/CENPI/PLK1/BUB1B/CENPE/CENPM/NDC80/ERCC6L/CENPU/BIRC5/NUF2/BUB1/CENPK/CDCA8/KIF2C/CDC20/SGO1/SKA1/CENPA/UBE2C/SPC24/AURKB | | 0.036976 | | | | REACTOME |
| REACTOME_MRNA_SPLICING_MINOR_PATHWAY | -0.49031 | 0.002046 | SF3B1/POLR2F/GTF2F1/PRPF6/DDX42/SRSF7/ZMAT5/GTF2F2/SNRPE/SF3B2/POLR2G/SNRPF/SF3B5/SF3B6/POLR2J/SRSF6/SRSF2/SNRNP200/DDX23/LSM2/POLR2D/SNRPD1/SF3B4/POLR2H/SNRNP40/SNRPD2/SNRPG/EFTUD2/YBX1/SNRPB | | 0.013295 | | | | REACTOME |
| REACTOME_MUSCLE_CONTRACTION | 0.652557 | 6.59E-09 | ATP2B3/CAMK2A/RYR2/SLC8A2/FGF13/SCN2B/SCN3B/KCNJ4/KCNK1/KCNK12/KCNJ12/KCNK4/ATP1A3/KCNK9/SCN9A/SCN2A/KCNIP2/FXYD1/ATP2B2/FGF12/CASQ2/CAMK2B/TNNT1/CACNA2D2/FXYD7/SCN1B/MYBPC1/KCNIP4/ATP1B1/SCN4B/NOS1/KCNK3/SCN8A/ACTC1/CACNA1C/GUCY1B1/CAMK2G/ITPR1/PAK1/ATP1A2/CACNB2/GUCY1A1/CACNG8/KCNJ11/TMOD2/NPPC/KCNIP3/SCN5A/RYR3/RYR1/FGF14/SCN7A/CACNB1/CALM1/ACTA1/NPPA/SORBS1/FXYD3/FKBP1B/LMOD1/MYL3 | | 0.012245 | | | | REACTOME |
| REACTOME_N_GLYCAN_TRIMMING_IN_THE_ER_AND_CALNEXIN_CALRETICULIN_CYCLE | -0.47979 | 0.036347 | PSMC1/UBC/UBXN1/CANX/TRIM13/PRKCSH/UBA52/SYVN1/UGGT1/MOGS/EDEM2/NGLY1/OS9/GANAB/PDIA3/MAN1B1/EDEM1/DERL2/UGGT2/CALR | | 0.0277 | | | | REACTOME |
| REACTOME_NEGATIVE_EPIGENETIC_REGULATION_OF_RRNA_EXPRESSION | -0.46345 | 0.000715 | H4C15/SIN3A/TBP/H2AZ2/TAF1D/POLR2F/TAF1B/GTF2H3/TAF1C/POLR1A/GTF2H4/RRP8/SAP30BP/GTF2H2/H3-3A/CDK7/H2BC12/H4C8/TAF1A/MBD2/TTF1/H2AC6/H3-3B/POLR1B/HDAC1/POLR1E/H2BC8/POLR2H/H4C11/DNMT1/SAP30/SUV39H1/H2AZ1/POLR1C/H2BC4/H2BC15/H2AX/DNMT3B/H3C6/H2BC5/H2AC20/H2AC8/H2BC11/H4C5/H3C10 | | 0.003119 | | | | REACTOME |
| REACTOME_NEGATIVE_REGULATION_OF_NOTCH4_SIGNALING | -0.40804 | 0.048186 | PSMD8/PSME3/PSMC3/PSMD6/PSMB9/PSMF1/PSMB8/CUL1/PSME1/PSMD2/PSMA3/PSMC1/PSMA1/UBC/PSMB1/PSMB2/PSMA5/PSMD9/PSMB4/PSMB7/PSMD11/PSMD14/PSMD3/AKT1/PSMB6/PSMA6/PSMD13/PSMA4/PSMD12/PSMC4/PSMD5/PSMC2/PSMB3/UBA52/PSMA7/PSME2/SEM1/PSMA2/TACC3 | | 0.011865 | | | | REACTOME |
| REACTOME_NEUROTRANSMITTER_RELEASE_CYCLE | 0.81496 | 6.59E-09 | SLC17A7/SYN2/SYT1/GLS2/SLC32A1/SNAP25/GAD2/SYN1/RAB3A/SLC6A13/CPLX1/RIMS1/PPFIA2/SYN3/STXBP1/TSPOAP1/SLC1A2/STX1A/SLC6A1/APBA1/SLC1A1/GAD1/SLC6A12/MAOA/PPFIA3/VAMP2/GLS/SLC1A7/PPFIA4/LIN7B/ALDH5A1 | | 0.037496 | | | | REACTOME |
| REACTOME_NITRIC_OXIDE_STIMULATES_GUANYLATE_CYCLASE | 0.721473 | 0.009322 | PDE2A/PDE1A/PDE1B/PDE11A/NOS1/KCNMA1/GUCY1B1/ITPR1/IRAG1/PDE10A/GUCY1A1/KCNMB2/PRKG1/GUCY1A2/PDE5A/KCNMB4/NOS3 | | 0.014421 | | | | REACTOME |
| REACTOME_NOREPINEPHRINE_NEUROTRANSMITTER_RELEASE_CYCLE | 0.873308 | 4.92E-05 | SYT1/SNAP25/RAB3A/CPLX1/RIMS1/PPFIA2/STXBP1/TSPOAP1/STX1A/MAOA/PPFIA3/VAMP2 | | 0.026596 | | | | REACTOME |
| REACTOME_NUCLEAR_IMPORT_OF_REV_PROTEIN | -0.6393 | 0.000164 | NUP42/KPNB1/NUP155/NUP43/NUP160/NUP153/NPM1/NUP58/RAN/NUP88/NUP210/NUP98/RAE1/POM121/AAAS/NUP54/NUP35/NUP62/SEC13/POM121C/NDC1/NUP85/NUP188/NUP205/NUP37/NUP107/RCC1 | | 0.009111 | | | | REACTOME |
| REACTOME_NUCLEAR_PORE_COMPLEX_NPC_DISASSEMBLY | -0.61568 | 0.000411 | NUP42/NUP155/NUP43/NUP160/NUP153/NUP58/NUP88/NUP210/NUP98/RAE1/POM121/AAAS/NUP54/NUP35/NUP62/SEC13/POM121C/NDC1/NEK6/NUP85/NUP188/NUP205/NUP37/NUP107/CCNB1/CDK1/CCNB2 | | 0.032955 | | | | REACTOME |
| REACTOME_NUCLEOTIDE_BIOSYNTHESIS | -0.66415 | 0.024424 | DHODH/PPAT/GART/UMPS/IMPDH2/CAD/ADSL/PAICS/IMPDH1/ADSS1/PFAS | | 0.003706 | | | | REACTOME |
| REACTOME_NUCLEOTIDE_EXCISION_REPAIR | -0.39403 | 0.002595 | GTF2H3/ZNF830/RFC5/UBE2I/PPIE/PIAS3/UBC/ERCC1/MCRS1/CUL4A/GTF2H4/POLD4/COPS3/COPS6/TCEA1/GTF2H2/NFRKB/CDK7/ACTR5/POLR2G/CETN2/HMGN1/POLR2J/LIG3/RPA1/XRCC1/POLE3/POLR2D/UBA52/XAB2/CHD1L/ISY1/POLR2H/POLD3/ACTB/ACTL6A/POLD2/RUVBL1/DDB2/POLD1/RPA3/RFC4/ELL/PCNA/RFC2/LIG1/POLE2/POLE/RFC3 | | 0.003903 | | | | REACTOME |
| REACTOME_ORC1_REMOVAL_FROM_CHROMATIN | -0.51846 | 3.68E-05 | PSMD2/PSMA3/PSMC1/PSMA1/UBC/PSMB1/PSME4/MCM4/PSMB2/PSMA5/PSMD9/PSMB4/PSMB7/PSMD11/PSMD14/PSMD3/PSMB6/PSMA6/PSMD13/PSMA4/PSMD12/PSMC4/PSMD5/PSMC2/PSMB3/ORC5/SKP2/UBA52/PSMA7/PSME2/SEM1/PSMA2/MCM6/MCM3/ORC6/MCM5/CDK2/MCM8/MCM2/CDT1/CCNA2/MCM7/CDC6/ORC1 | | 0.000528 | | | | REACTOME |
| REACTOME_OXIDATIVE_STRESS_INDUCED_SENESCENCE | -0.39226 | 0.004562 | H2BC4/H2BC15/H2AX/E2F1/TP53/CBX8/JUN/PHC2/H3C6/H2BC5/MAP2K3/CBX2/H2AC20/KDM6B/H2AC8/CDKN2C/CDK4/EZH2/H2BC11/MDM4/H4C5/H3C10/E2F2/CDK6 | | 0.041063 | | | | REACTOME |
| REACTOME_PERK_REGULATES_GENE_EXPRESSION | -0.65131 | 0.000279 | DCP2/EXOSC7/EIF2AK3/EXOSC3/EIF2S2/KHSRP/EXOSC2/EIF2S1/CEBPG/EXOSC9/ASNS/ATF4/DIS3/EXOSC8/EXOSC4/DDIT3/HSPA5/ATF3/CEBPB/CCL2/IGFBP1/CXCL8 | | 0.002872 | | | | REACTOME |
| REACTOME_PHOSPHORYLATION_OF_THE_APC_C | -0.62545 | 0.019002 | CDC26/CDC23/ANAPC7/ANAPC2/CDC16/ANAPC11/UBE2S/PLK1/CCNB1/CDK1/UBE2C | | 0.000415 | | | | REACTOME |
| REACTOME_POSITIVE_EPIGENETIC_REGULATION_OF_RRNA_EXPRESSION | -0.45602 | 0.001506 | TTF1/KAT2A/DDX21/BAZ1B/H2AC6/H3-3B/MBD3/POLR1B/MTA2/HDAC1/MYBBP1A/POLR1E/DEK/MTA1/H2BC8/POLR2H/H4C11/ACTB/H2AZ1/POLR1C/H2BC4/H2BC15/H2AX/CBX3/H3C6/H2BC5/H2AC20/H2AC8/H2BC11/H4C5/H3C10 | | 0.022489 | | | | REACTOME |
| REACTOME_POSTMITOTIC_NUCLEAR_PORE_COMPLEX_NPC_REFORMATION | -0.63865 | 0.001336 | TNPO1/UBE2I/KPNB1/NUP155/NUP43/NUP160/NUP58/RAN/NUP98/POM121/NUP54/NUP35/NUP62/SEC13/NDC1/NUP85/NUP188/NUP205/NUP37/NUP107/RCC1 | | 0.001838 | | | | REACTOME |
| REACTOME_POTASSIUM_CHANNELS | 0.76353 | 6.59E-09 | HCN1/KCNJ3/KCNC2/KCNS1/GNG3/KCNJ4/KCNK1/KCNA1/KCNJ12/KCNK4/KCNQ5/KCNB1/KCNH5/KCNN1/KCNK9/KCNH7/KCNH3/KCNA5/KCNAB1/KCNH1/KCNC1/GABBR1/GABBR2/KCNAB2/KCNJ9/KCNJ6/KCNQ3/HCN4/KCNK3/KCNA3/KCNMA1/KCNA6/KCNC3/KCNC4/ABCC8/KCNJ10/KCNJ11/KCNA2/GNB5/HCN2/KCNMB2/KCNQ2/KCNS3/KCNJ16/KCNH8 | | 0.020481 | | | | REACTOME |
| REACTOME_PRE_NOTCH_EXPRESSION_AND_PROCESSING | -0.50452 | 5.29E-05 | KAT2A/H2AC6/H3-3B/NOTCH3/TMED2/FURIN/MAML1/MAML2/H2BC8/POGLUT1/ATP2A1/SIRT6/H4C11/RFNG/H2AZ1/NOTCH1/H2BC4/H2BC15/H2AX/E2F1/POFUT1/TP53/RUNX1/JUN/H3C6/H2BC5/H2AC20/H2AC8/LFNG/H2BC11/H4C5/H3C10/ELF3 | | 0.034743 | | | | REACTOME |
| REACTOME_PROCESSING_OF_DNA_DOUBLE_STRAND_BREAK_ENDS | -0.55205 | 2.11E-06 | PIAS4/H2BC12/ATRIP/H4C8/NSD2/MDC1/HUS1/RMI1/RMI2/DNA2/UIMC1/RPA1/UBA52/TOPBP1/WRN/BARD1/RHNO1/H2BC8/SIRT6/H4C11/RAD9A/H2BC4/TOP3A/H2BC15/TIPIN/H2AX/RBBP8/PPP4C/RPA3/BRCA1/BLM/RFC4/H2BC5/CDK2/RFC2/TIMELESS/CHEK1/RFC3/CCNA2/H2BC11/CLSPN/BRIP1/H4C5/EXO1 | | 0.001279 | | | | REACTOME |
| REACTOME_RECOGNITION_OF_DNA_DAMAGE_BY_PCNA_CONTAINING_REPLICATION_COMPLEX | -0.61408 | 0.001153 | USP1/RPA1/POLE3/UBA52/POLD3/POLD2/POLD1/RPA3/RFC4/RAD18/PCNA/RFC2/POLE2/POLE/RFC3/DTL | | 0.032417 | | | | REACTOME |
| REACTOME_REGULATION_OF_GLUCOKINASE_BY_GLUCOKINASE_REGULATORY_PROTEIN | -0.60564 | 0.001326 | NUP42/NUP155/NUP43/NUP160/NUP153/NUP58/NUP88/NUP210/NUP98/RAE1/POM121/AAAS/NUP54/NUP35/NUP62/SEC13/POM121C/NDC1/NUP85/NUP188/NUP205/NUP37/NUP107 | | 0.03069 | | | | REACTOME |
| REACTOME_REGULATION_OF_HMOX1_EXPRESSION_AND_ACTIVITY | -0.44834 | 0.002355 | PSMD8/PSME3/PSMC3/KEAP1/PSMD6/PSMB9/PSMF1/PSMB8/CUL1/PSME1/PSMD2/PSMA3/PSMC1/PSMA1/UBC/PSMB1/PSME4/MAFK/PSMB2/CSNK2A1/PSMA5/PSMD9/PSMB4/PSMB7/PSMD11/PSMD14/PSMD3/PSMB6/PSMA6/PSMD13/PSMA4/PSMD12/PSMC4/PSMD5/CSNK2B/PSMC2/PSMB3/SKP2/UBA52/BACH1/PSMA7/PSME2/SEM1/PSMA2/HM13/HMOX1 | | 0.01704 | | | | REACTOME |
| REACTOME_REGULATION_OF_MRNA_STABILITY_BY_PROTEINS_THAT_BIND_AU_RICH_ELEMENTS | -0.42867 | 0.001168 | PSMD6/PSMB9/PSMF1/PABPC1/PSMB8/PSME1/EXOSC5/NUP214/DCP1A/PSMD2/TNPO1/PSMA3/PSMC1/PSMA1/EIF4G1/UBC/PSMB1/PSME4/DCP2/PSMB2/EXOSC7/PSMA5/PSMD9/PSMB4/EXOSC3/PSMB7/ELAVL1/PSMD11/PSMD14/PSMD3/KHSRP/AKT1/PSMB6/PSMA6/PSMD13/PSMA4/PSMD12/PSMC4/EXOSC2/ZFP36L1/PSMD5/PSMC2/PSMB3/HSPB1/UBA52/SET/EXOSC9/PSMA7/HNRNPD/PSME2/SEM1/DIS3/EXOSC8/MAPKAPK2/PSMA2/ZFP36/EXOSC4 | | 0.003331 | | | | REACTOME |
| REACTOME_REGULATION_OF_RUNX3_EXPRESSION_AND_ACTIVITY | -0.50266 | 0.000856 | PSMD8/PSME3/EP300/PSMC3/PSMD6/PSMB9/PSMF1/PSMB8/SMURF2/PSME1/PSMD2/PSMA3/PSMC1/PSMA1/UBC/PSMB1/MDM2/PSMB2/PSMA5/PSMD9/PSMB4/PSMB7/PSMD11/PSMD14/PSMD3/CBFB/PSMB6/PSMA6/PSMD13/SMURF1/PSMA4/PSMD12/PSMC4/TGFB1/PSMD5/PSMC2/PSMB3/UBA52/PSMA7/PSME2/SEM1/RUNX3/PSMA2 | | 0.015171 | | | | REACTOME |
| REACTOME_REGULATION_OF_TP53_ACTIVITY | -0.31077 | 0.011378 | MBD3/UBA52/TOPBP1/NOC2L/MTA2/HDAC1/WRN/BARD1/TP53RK/PRMT5/RHNO1/PRDM1/DAXX/RAD9A/SSRP1/TOP3A/TP53/RBBP8/RPA3/BRCA1/BLM/RFC4/CDK2/RFC2/CHEK1/RFC3/CCNA2/CHEK2/CDK1/AURKA/MDM4/BRIP1/TP73/TPX2/EXO1/PLK3/AURKB/POU4F1 | | 0.015475 | | | | REACTOME |
| REACTOME_REGULATION_OF_TP53_ACTIVITY_THROUGH_PHOSPHORYLATION | -0.42326 | 0.000995 | STK11/ATRIP/TAF11/TAF6/HUS1/RMI1/RMI2/TAF7/DNA2/CSNK2B/RPA1/UBA52/TOPBP1/NOC2L/WRN/BARD1/TP53RK/RHNO1/RAD9A/SSRP1/TOP3A/TP53/RBBP8/RPA3/BRCA1/BLM/RFC4/CDK2/RFC2/CHEK1/RFC3/CCNA2/CHEK2/AURKA/MDM4/BRIP1/TPX2/EXO1/PLK3/AURKB | | 0.000353 | | | | REACTOME |
| REACTOME_REPRODUCTION | -0.47465 | 0.000164 | SUN1/H2BC8/H4C11/H2AZ1/LMNA/H2BC4/TOP3A/H2BC15/H2AX/RBBP8/SYCE2/RPA3/H3C6/BRCA1/BLM/H2BC5/CDK2/MSH5/PSMC3IP/BRCA2/H2AC20/H2AC8/CDK4/H2BC11/H4C5/H3C10/RAD51/LMNB1/MND1 | | 0.027151 | | | | REACTOME |
| REACTOME_RESOLUTION_OF_D_LOOP_STRUCTURES | -0.66515 | 3.61E-05 | RMI1/MUS81/RMI2/DNA2/WRN/BARD1/RTEL1/SLX1A/GEN1/TOP3A/RBBP8/BRCA1/BLM/BRCA2/RAD51AP1/XRCC2/BRIP1/RAD51/EXO1/EME1 | | 0.034505 | | | | REACTOME |
| REACTOME_RESPONSE_OF_EIF2AK1_HRI_TO_HEME_DEFICIENCY | -0.73355 | 0.004043 | EIF2S1/CEBPG/ATF5/EIF2AK1/ASNS/ATF4/DDIT3/PPP1R15A/GRB10/ATF3/CEBPB/TRIB3 | | 0.019371 | | | | REACTOME |
| REACTOME_RHO_GTPASES_ACTIVATE_FORMINS | -0.4925 | 7.96E-08 | RCC2/CENPL/SEC13/DSN1/INCENP/CENPQ/B9D2/ACTB/CENPP/SPDL1/NUP85/CENPN/MIS12/ITGB1/ITGB3BP/MAD1L1/NDE1/DVL2/NUP37/ACTG1/NUP107/CENPO/ZWILCH/PFN1/SPC25/CENPF/KNTC1/CENPH/SGO2/MAD2L1/KIF18A/KNL1/ZWINT/CENPI/PLK1/TUBA1C/BUB1B/CENPE/CENPM/NDC80/ERCC6L/TUBB6/CENPU/DIAPH3/BIRC5/NUF2/BUB1/CENPK/CDCA8/KIF2C/CDC20/SGO1/SKA1/CENPA/SPC24/AURKB | | 0.031519 | | | | REACTOME |
| REACTOME_RNA_POLYMERASE_I_PROMOTER_ESCAPE | -0.48986 | 0.001445 | H4C15/TBP/H2AZ2/TAF1D/RRN3/POLR2F/TAF1B/GTF2H3/TAF1C/POLR1A/GTF2H4/GTF2H2/H3-3A/CDK7/H2BC12/H4C8/TAF1A/H2AC6/H3-3B/POLR1B/POLR1E/H2BC8/POLR2H/H4C11/H2AZ1/POLR1C/H2BC4/H2BC15/H2AX/CBX3/H3C6/H2BC5/H2AC20/H2AC8/H2BC11/H4C5/H3C10 | | 0.004228 | | | | REACTOME |
| REACTOME_RNA_POLYMERASE_I_TRANSCRIPTION | -0.39122 | 0.008894 | GTF2H2/H3-3A/CDK7/RBBP4/H2BC12/H4C8/TAF1A/MBD2/GATAD2A/TTF1/KAT2A/H2AC6/H3-3B/MBD3/POLR1B/MTA2/HDAC1/POLR1E/MTA1/H2BC8/POLR2H/CAVIN1/H4C11/H2AZ1/POLR1C/H2BC4/H2BC15/H2AX/CBX3/H3C6/H2BC5/H2AC20/H2AC8/H2BC11/H4C5/H3C10 | | 0.014877 | | | | REACTOME |
| REACTOME_RNA_POLYMERASE_II_TRANSCRIBES_SNRNA_GENES | -0.38393 | 0.01925 | NABP2/GTF2B/SUPT5H/INTS8/GTF2E1/NCBP2/SSU72/INTS9/INTS3/INTS6/ICE2/INTS12/SP1/TBP/TAF13/GTF2A2/TAF5/INTS2/CCNT1/PCF11/POLR2F/GTF2F1/RPRD1B/ELL2/INTS10/INTS7/ZNF143/INTS13/SNAPC2/INTS5/GTF2F2/CDK7/POLR2G/TAF11/TAF6/GTF2E2/INTS11/SNAPC4/POLR2J/INTS4/INTS1/CCNK/INTS14/POLR2D/SRRT/NABP1/POLR2H/SNAPC1/ELL | | 0.002839 | | | | REACTOME |
| REACTOME_RNA_POLYMERASE_II_TRANSCRIPTION_TERMINATION | -0.47827 | 0.000808 | FIP1L1/PABPN1/PCF11/CSTF3/CPSF2/CSTF2/WDR33/CHTOP/CSTF1/SRSF7/SRRM1/SARNP/PAPOLA/CPSF7/SNRPE/SYMPK/MAGOHB/THOC5/THOC2/SNRPF/ZC3H11A/SRSF11/LSM10/SRSF3/SRSF4/CPSF1/SRSF6/MAGOH/SRSF2/THOC1/U2AF1/DDX39B/EIF4A3/CLP1/CPSF3/U2AF1L4/SRSF9/U2AF2/SNRPG/THOC6/ZNF473/DDX39A/CPSF4/ALYREF/SNRPB | | 0.003186 | | | | REACTOME |
| REACTOME_RRNA_MODIFICATION_IN_THE_NUCLEUS_AND_CYTOSOL | -0.53981 | 6.94E-05 | NHP2/TBL3/UTP4/MPHOSPH10/TSR3/PDCD11/UTP14A/RPS14/UTP18/WDR36/WDR3/FCF1/HEATR1/NOP14/WDR75/RPS9/EMG1/RPS7/TRMT112/RRP36/NOP10/UTP6/DDX47/WDR43/NOC4L/DHX37/GAR1/PNO1/DKC1/DDX52/UTP11/NOP58/UTP20/RPS2/UTP15/FBL/WDR46/NOL11/NOP56/DDX49/DCAF13/BUD23/RRP9/NOP2 | | 0.000956 | | | | REACTOME |
| REACTOME_RUNX1_REGULATES_TRANSCRIPTION_OF_GENES_INVOLVED_IN_DIFFERENTIATION_OF_HSCS | -0.47947 | 2.52E-05 | PSMD2/PSMA3/PSMC1/PSMA1/UBC/PSMB1/PSME4/PSMB2/PSMA5/PSMD9/PSMB4/H3-3A/PSMB7/CDK7/PSMD11/PSMD14/PSMD3/H2BC12/CBFB/H4C8/PSMB6/PSMA6/PSMD13/PSMA4/PSMD12/PSMC4/PSMD5/PSMC2/H2AC6/PSMB3/H3-3B/SPI1/UBA52/PSMA7/PSME2/SEM1/H2BC8/MYB/TCF12/PSMA2/H4C11/H2AZ1/ABL1/H2BC4/H2BC15/H2AX/RUNX1/H3C6/H2BC5/TCF3/H2AC20/H2AC8/H2BC11/TP73/H4C5/H3C10 | | 0.003131 | | | | REACTOME |
| REACTOME_S_PHASE | -0.5309 | 6.59E-09 | UBE2E1/PSMD2/RFC5/PSMA3/PSMC1/FZR1/PSMA1/TFDP2/UBC/PSMB1/PSME4/STAG2/MCM4/POLD4/PSMB2/PSMA5/PSMD9/CDC26/PSMB4/CDC23/PSMB7/CDK7/RBBP4/PSMD11/PSMD14/PSMD3/ANAPC7/AKT1/ANAPC2/PSMB6/PSMA6/E2F4/PSMD13/PRIM1/PSMA4/PSMD12/PSMC4/CDC16/DNA2/PSMD5/PSMC2/RPA1/STAG1/PSMB3/POLE3/CCNE2/ORC5/RB1/WEE1/SKP2/UBA52/LIN37/ANAPC11/CDKN1A/PSMA7/PSME2/POLA1/SEM1/ESCO1/POLD3/PSMA2/CDC25B/MCM6/POLD2/MYC/GINS3/E2F1/LIN9/PRIM2/CCNE1/CKS1B/MCM3/POLD1/RPA3/FEN1/POLA2/RFC4/PTK6/ORC6/UBE2S/MCM5/CDK2/MCM8/PCNA/RFC2/MCM2/LIG1/POLE2/POLE/GINS4/RFC3/GINS2/CDT1/CDC25A/CCNA2/CDCA5/CDK4/GINS1/MCM7/CDC6/ORC1/ESCO2/UBE2C/CDC45 | | 0.000636 | | | | REACTOME |
| REACTOME_SCF_SKP2_MEDIATED_DEGRADATION_OF_P27_P21 | -0.48481 | 0.000855 | PSMD6/PSMB9/PSMF1/PSMB8/CUL1/PSME1/PSMD2/PSMA3/PSMC1/PSMA1/UBC/PSMB1/PSMB2/PSMA5/PSMD9/PSMB4/PSMB7/PSMD11/PSMD14/PSMD3/PSMB6/PSMA6/PSMD13/PSMA4/PSMD12/PSMC4/PSMD5/PSMC2/PSMB3/CCNE2/SKP2/UBA52/CDKN1A/PSMA7/PSME2/SEM1/PSMA2/CCNE1/CKS1B/PTK6/CDK2/CCNA2/CDK4 | | 0.002639 | | | | REACTOME |
| REACTOME_SENESCENCE_ASSOCIATED_SECRETORY_PHENOTYPE_SASP | -0.54901 | 2.64E-06 | H2BC8/NFKB1/H4C11/H2AZ1/RELA/MAPK7/VENTX/H2BC4/H2BC15/H2AX/JUN/H3C6/UBE2S/H2BC5/CDK2/H2AC20/H2AC8/CDKN2C/CCNA2/CDK4/H2BC11/H4C5/H3C10/CEBPB/UBE2C/CDK6/IL1A/IL6/CXCL8 | | 0.005845 | | | | REACTOME |
| REACTOME_SENSORY_PERCEPTION | 0.588373 | 2.62E-06 | GRM1/SNAP25/SYN1/SCN2B/RAB3A/CABP1/GNAL/RBP4/PCLO/SCN9A/SYP/BSN/AKR1C1/SCN2A/AKR1C3/ATP2B2/GPC5/OTOF/CACNA2D2/CLIC5/SCN1B/STX1A/GPC3/SCN4B/KCNMA1/STRC/MYO3B/RIPOR2/CACNB2/HSD17B6/MYO15A/LRAT/EPB41L1/VAMP2/CACNA1D/GNB5/USH1C/EPB41L3/RDH12/CALM1/GPIHBP1/LRP2/PRKCQ/SCNN1B/ANO2/ABCA4/OTOGL/BCO2/CDH23/ATP2B1/SLC17A8/CYP4V2/RLBP1/NMT2/RGS9BP/SPTBN1/PRKCA/CAMKMT/RGS9/PLS1 | | 0.006401 | | | | REACTOME |
| REACTOME_SENSORY_PERCEPTION_OF_TASTE | 0.773472 | 0.001489 | GRM1/SCN2B/SCN9A/SCN2A/SCN1B/SCN4B | | 0.049139 | | | | REACTOME |
| REACTOME_SEPARATION_OF_SISTER_CHROMATIDS | -0.45108 | 1.31E-08 | STAG2/PSMB2/PSMA5/PSMD9/CDC26/PSMB4/NUP43/CDC23/PSMB7/CENPS/NUP160/PSMD11/PSMD14/PSMD3/ANAPC7/RPS27/TUBB2B/ANAPC2/ZW10/PSMB6/PSMA6/PPP1CC/SKA2/MAPRE1/PSMD13/TUBB1/PSMA4/PSMD12/PSMC4/CDC16/NUP98/PMF1/PSMD5/PSMC2/STAG1/PSMB3/TUBB4B/UBA52/ANAPC11/PSMA7/PSME2/SEM1/RCC2/CENPL/SEC13/DSN1/INCENP/CENPQ/B9D2/PSMA2/CENPP/SPDL1/NUP85/CENPN/MIS12/ITGB3BP/MAD1L1/NDE1/NUP37/NUP107/CENPO/ZWILCH/SPC25/CENPF/KNTC1/UBE2S/PTTG1/CENPH/SGO2/MAD2L1/KIF18A/KNL1/ZWINT/CENPI/PLK1/TUBA1C/BUB1B/CENPE/CDCA5/CENPM/NDC80/ERCC6L/TUBB6/CENPU/BIRC5/NUF2/BUB1/CENPK/CDCA8/KIF2C/CDC20/ESPL1/SGO1/SKA1/CENPA/UBE2C/SPC24/AURKB | | 0.002526 | | | | REACTOME |
| REACTOME_SNRNP_ASSEMBLY | -0.59709 | 1.93E-05 | GEMIN2/TGS1/NUP214/WDR77/NUP42/NUP155/NUP43/GEMIN4/SNRPE/NUP160/NUP153/GEMIN6/SNRPF/NUP58/NUP88/NUP210/NUP98/RAE1/GEMIN5/SMN1/POM121/AAAS/SMN2/SNRPD1/GEMIN7/NUP54/NUP35/NUP62/PRMT5/SEC13/POM121C/NDC1/SNRPD2/SNRPG/NUP85/NUP188/NUP205/DDX20/NUP37/NUP107/SNRPB | | 0.00042 | | | | REACTOME |
| REACTOME_STABILIZATION_OF_P53 | -0.50563 | 0.000801 | PSMD6/PSMB9/PSMF1/PSMB8/PSME1/PSMD2/PSMA3/PSMC1/PSMA1/UBC/PSMB1/MDM2/PSME4/PSMB2/PSMA5/PSMD9/PSMB4/PSMB7/PSMD11/PSMD14/PSMD3/PSMB6/PSMA6/PSMD13/PSMA4/PSMD12/PSMC4/PSMD5/PSMC2/PSMB3/UBA52/PSMA7/PSME2/SEM1/PSMA2/TP53/CHEK2/MDM4 | | 0.006405 | | | | REACTOME |
| REACTOME_STIMULI_SENSING_CHANNELS | 0.572832 | 0.001623 | RYR2/ASIC2/ANO3/TRPV6/ANO5/CASQ2/CLCA4/WNK2/ANO4/UNC80/CLCN4/NALCN/UNC79/TRPC3/TRPM3/RYR3/RYR1/CALM1/FKBP1B/SCNN1B/TRPM6/TRPV3/ANO2/SGK2/SLC9B2 | | 0.000138 | | | | REACTOME |
| REACTOME_SUMOYLATION_OF_DNA_DAMAGE_RESPONSE_AND_REPAIR_PROTEINS | -0.49691 | 8.68E-05 | NUP214/UBE2I/NSMCE1/PCGF2/NUP42/STAG2/NUP155/NUP43/RAD52/PIAS4/NUP160/NUP153/NUP58/MDC1/CETN2/NUP88/SMC6/HDAC7/NSMCE2/XRCC4/PHC1/NUP210/NUP98/RAE1/RPA1/STAG1/POM121/AAAS/SMC5/NUP54/NUP35/WRN/NUP62/SEC13/POM121C/NDC1/NUP85/NUP188/NUP205/TDG/NUP37/NUP107/CBX8/PHC2/BRCA1/BLM/CBX2 | | 0.042067 | | | | REACTOME |
| REACTOME_SUMOYLATION_OF_DNA_REPLICATION_PROTEINS | -0.60225 | 7.82E-05 | NUP214/UBE2I/PIAS3/NUP42/NUP155/NUP43/PIAS4/NUP160/NUP153/NUP58/TOP1/NUP88/NUP210/NUP98/RAE1/POM121/AAAS/NUP54/NUP35/NUP62/SEC13/POM121C/NDC1/INCENP/NUP85/NUP188/NUP205/NUP37/NUP107/PCNA/TOP2A/AURKA/BIRC5/CDCA8/AURKB | | 0.006965 | | | | REACTOME |
| REACTOME_SUMOYLATION_OF_RNA_BINDING_PROTEINS | -0.56856 | 0.000365 | NUP214/UBE2I/HNRNPC/PCGF2/NUP42/NUP155/NUP43/NUP160/NUP153/NUP58/NUP88/PHC1/NUP210/NUP98/RAE1/POM121/AAAS/NUP54/NUP35/NUP62/SEC13/NOP58/POM121C/NDC1/NUP85/NUP188/NUP205/NUP37/NUP107/CBX8/PHC2/CBX2 | | 0.026442 | | | | REACTOME |
| REACTOME_SUMOYLATION_OF_SUMOYLATION_PROTEINS | -0.59599 | 0.00092 | NUP214/UBE2I/NUP42/NUP155/NUP43/PIAS4/NUP160/NUP153/NUP58/NUP88/NUP210/NUP98/RAE1/POM121/AAAS/NUP54/NUP35/NUP62/SEC13/POM121C/NDC1/NUP85/NUP188/NUP205/NUP37/NUP107 | | 0.012523 | | | | REACTOME |
| REACTOME_SUMOYLATION_OF_UBIQUITINYLATION_PROTEINS | -0.57084 | 0.001056 | NUP214/UBE2I/MDM2/NUP42/VHL/TRIM27/NUP155/NUP43/PIAS4/NUP160/NUP153/NUP58/NUP88/NUP210/NUP98/RAE1/POM121/AAAS/NUP54/NUP35/NUP62/SEC13/POM121C/NDC1/NUP85/NUP188/NUP205/NUP37/NUP107 | | 0.011865 | | | | REACTOME |
| REACTOME_SWITCHING_OF_ORIGINS_TO_A_POST_REPLICATIVE_STATE | -0.52661 | 1.88E-06 | UBE2E1/PSMD2/PSMA3/PSMC1/FZR1/PSMA1/UBC/PSMB1/PSME4/MCM4/PSMB2/PSMA5/PSMD9/CDC26/PSMB4/CDC23/PSMB7/PSMD11/PSMD14/PSMD3/ANAPC7/ANAPC2/PSMB6/PSMA6/PSMD13/PSMA4/PSMD12/PSMC4/CDC16/PSMD5/PSMC2/PSMB3/CCNE2/ORC5/SKP2/UBA52/ANAPC11/PSMA7/PSME2/SEM1/PSMA2/MCM6/CCNE1/MCM3/ORC6/UBE2S/MCM5/CDK2/MCM8/MCM2/CDT1/CCNA2/MCM7/CDC6/ORC1/UBE2C | | 0.000237 | | | | REACTOME |
| REACTOME_SYNTHESIS_OF_BILE_ACIDS_AND_BILE_SALTS | 0.723635 | 0.001596 | AKR1C2/SLC27A2/AKR1C1/CYP39A1/AKR1C3/CYP46A1/CYP7B1/OSBPL1A/BAAT | | 0.00019 | | | | REACTOME |
| REACTOME_SYNTHESIS_OF_BILE_ACIDS_AND_BILE_SALTS_VIA_24_HYDROXYCHOLESTEROL | 0.901591 | 0.000287 | AKR1C2/SLC27A2/AKR1C1/CYP39A1/AKR1C3/CYP46A1 | | 0.000191 | | | | REACTOME |
| REACTOME_SYNTHESIS_OF_BILE_ACIDS_AND_BILE_SALTS_VIA_27_HYDROXYCHOLESTEROL | 0.79065 | 0.028579 | AKR1C2/AKR1C1/AKR1C3/CYP7B1 | | 3.61E-05 | | | | REACTOME |
| REACTOME_SYNTHESIS_OF_BILE_ACIDS_AND_BILE_SALTS_VIA_7ALPHA_HYDROXYCHOLESTEROL | 0.705242 | 0.041275 | AKR1C2/SLC27A2/AKR1C1/AKR1C3/CYP7B1/BAAT | | 3.36E-05 | | | | REACTOME |
| REACTOME_SYNTHESIS_OF_DNA | -0.57872 | 6.59E-09 | UBE2E1/PSMD2/RFC5/PSMA3/PSMC1/FZR1/PSMA1/UBC/PSMB1/PSME4/MCM4/POLD4/PSMB2/PSMA5/PSMD9/CDC26/PSMB4/CDC23/PSMB7/PSMD11/PSMD14/PSMD3/ANAPC7/ANAPC2/PSMB6/PSMA6/PSMD13/PRIM1/PSMA4/PSMD12/PSMC4/CDC16/DNA2/PSMD5/PSMC2/RPA1/PSMB3/POLE3/CCNE2/ORC5/SKP2/UBA52/ANAPC11/PSMA7/PSME2/POLA1/SEM1/POLD3/PSMA2/MCM6/POLD2/GINS3/PRIM2/CCNE1/MCM3/POLD1/RPA3/FEN1/POLA2/RFC4/ORC6/UBE2S/MCM5/CDK2/MCM8/PCNA/RFC2/MCM2/LIG1/POLE2/POLE/GINS4/RFC3/GINS2/CDT1/CCNA2/GINS1/MCM7/CDC6/ORC1/UBE2C/CDC45 | | 0.000582 | | | | REACTOME |
| REACTOME_TELOMERE_EXTENSION_BY_TELOMERASE | -0.54938 | 0.044297 | POT1/SHQ1/NOP10/GAR1/DKC1/RTEL1/RUVBL2/RUVBL1/WRAP53/CDK2/CCNA2/PIF1/TERT | | 0.003417 | | | | REACTOME |
| REACTOME_TELOMERE_MAINTENANCE | -0.58821 | 9.15E-09 | POLR2J/POT1/PRIM1/DNA2/SHQ1/RPA1/H2AC6/TEN1/H3-3B/POLR2D/NOP10/GAR1/DKC1/WRN/POLA1/RTEL1/H2BC8/DSCC1/POLR2H/POLD3/H4C11/POLD2/H2AZ1/DAXX/RUVBL2/RUVBL1/H2BC4/H2BC15/H2AX/PRIM2/POLD1/RPA3/FEN1/POLA2/WRAP53/BLM/RFC4/CHTF18/H2BC5/CDK2/PCNA/RFC2/LIG1/H2AC20/H2AC8/RFC3/CCNA2/H2BC11/PIF1/H4C5/TERT | | 0.001504 | | | | REACTOME |
| REACTOME_TERMINATION_OF_TRANSLESION_DNA_SYNTHESIS | -0.58193 | 0.002866 | POLI/RPA1/POLE3/UBA52/POLH/POLD3/POLD2/POLD1/RPA3/RFC4/PCNA/RFC2/POLE2/POLE/RFC3/PCLAF | | 0.01084 | | | | REACTOME |
| REACTOME_TP53_REGULATES_TRANSCRIPTION_OF_CELL_CYCLE_GENES | -0.53449 | 0.000606 | CDKN1A/CNOT3/RBL1/CNOT10/BAX/PRMT1/E2F1/CCNE1/TP53/CDK2/PCNA/CCNA2/E2F7/CCNB1/E2F8/CDK1/AURKA/CDC25C/PLK3 | | 0.001058 | | | | REACTOME |
| REACTOME_TP53_REGULATES_TRANSCRIPTION_OF_DNA_REPAIR_GENES | -0.5373 | 0.000112 | CCNT1/POLR2F/GTF2F1/SUPT16H/GTF2H3/NELFB/PMS2/GTF2H4/MLH1/GTF2F2/TCEA1/GTF2H2/CDK7/CDK12/POLR2G/MDC1/POLR2J/NELFCD/CCNK/ELOA/CDK13/CTDP1/POLR2D/FOS/NELFE/POLR2H/SSRP1/DDB2/TP53/JUN/BRCA1/ELL/FANCC/FANCI/CHEK1/FANCD2 | | 0.000415 | | | | REACTOME |
| REACTOME_TP53_REGULATES_TRANSCRIPTION_OF_GENES_INVOLVED_IN_G2_CELL_CYCLE_ARREST | -0.64945 | 0.019127 | BAX/PRMT1/TP53/PCNA/CCNB1/CDK1/AURKA/CDC25C | | 0.013896 | | | | REACTOME |
| REACTOME_TRANSCRIPTION_COUPLED_NUCLEOTIDE_EXCISION_REPAIR_TC_NER | -0.44557 | 0.000735 | GTF2H3/ZNF830/RFC5/PPIE/UBC/ERCC1/CUL4A/GTF2H4/POLD4/COPS3/COPS6/TCEA1/GTF2H2/CDK7/POLR2G/HMGN1/POLR2J/LIG3/RPA1/XRCC1/POLE3/POLR2D/UBA52/XAB2/ISY1/POLR2H/POLD3/POLD2/POLD1/RPA3/RFC4/ELL/PCNA/RFC2/LIG1/POLE2/POLE/RFC3 | | 0.004702 | | | | REACTOME |
| REACTOME_TRANSCRIPTION_OF_E2F_TARGETS_UNDER_NEGATIVE_CONTROL_BY_DREAM_COMPLEX | -0.69402 | 0.003099 | LIN37/HDAC1/RBL1/MYC/E2F1/LIN9/PCNA/CDC25A/TOP2A/CDC6 | | 0.035882 | | | | REACTOME |
| REACTOME_TRANSCRIPTION_OF_THE_HIV_GENOME | -0.3845 | 0.02758 | NELFA/CCNT2/ERCC3/TAF1/GTF2B/SUPT5H/GTF2E1/NCBP2/ERCC2/MNAT1/TAF12/TAF15/TBP/TAF13/GTF2A2/TAF5/CCNT1/POLR2F/GTF2F1/TAF10/SUPT16H/GTF2H3/TAF2/NELFB/GTF2H4/GTF2F2/TCEA1/GTF2H2/TAF4/CDK7/POLR2G/TAF11/TAF6/GTF2E2/POLR2J/TAF7/NELFCD/CCNK/ELOA/CTDP1/POLR2D/NELFE/POLR2H/SSRP1/ELL | | 0.003852 | | | | REACTOME |
| REACTOME_TRANSCRIPTIONAL_REGULATION_BY_RUNX3 | -0.4441 | 0.000233 | PSMD2/PSMA3/PSMC1/PSMA1/UBC/PSMB1/MDM2/TCF7/PSMB2/PSMA5/PSMD9/PSMB4/PSMB7/PSMD11/PSMD14/PSMD3/CBFB/PSMB6/PSMA6/SMAD4/SNW1/PSMD13/SMURF1/PSMA4/PSMD12/PSMC4/TGFB1/PSMD5/TEAD1/PSMC2/KAT2A/PSMB3/UBA52/HES1/WWTR1/CDKN1A/MAML1/PSMA7/PSME2/MAML2/SEM1/ITGA4/RUNX3/PSMA2/MYC/NOTCH1/TCF7L1/BCL2L11/TP53/RUNX1/CCN2/TEAD3/TEAD2/TEAD4/JAG1/SPP1 | | 0.001822 | | | | REACTOME |
| REACTOME_TRANSCRIPTIONAL_REGULATION_BY_SMALL_RNAS | -0.56366 | 1.88E-06 | NUP42/AGO2/NUP155/NUP43/H3-3A/NUP160/NUP153/H2BC12/POLR2G/H4C8/NUP58/RAN/NUP88/POLR2J/NUP210/NUP98/RAE1/H2AC6/POM121/H3-3B/POLR2D/AAAS/NUP54/NUP35/NUP62/H2BC8/SEC13/POM121C/POLR2H/NDC1/H4C11/NUP85/H2AZ1/NUP188/NUP205/NUP37/H2BC4/H2BC15/H2AX/NUP107/H3C6/H2BC5/H2AC20/H2AC8/H2BC11/H4C5/H3C10 | | 0.002513 | | | | REACTOME |
| REACTOME_TRANSCRIPTIONAL_REGULATION_BY_VENTX | -0.50935 | 0.008175 | CDC26/CDC23/ANAPC7/ANAPC2/MOV10/CDC16/EHMT1/ANAPC11/NFKB1/RELA/VENTX/TP53/UBE2S/CEBPB/UBE2C/IL6 | | 0.000539 | | | | REACTOME |
| REACTOME_TRANSLESION_SYNTHESIS_BY_POLH | -0.60492 | 0.035771 | RFC5/UBC/NPLOC4/RPA1/UBA52/POLH/UFD1/RPA3/RFC4/PCNA/RFC2/RFC3 | | 0.008813 | | | | REACTOME |
| REACTOME_TRANSLESION_SYNTHESIS_BY_POLK | -0.6572 | 0.011436 | RPA1/UBA52/RPA3/RFC4/PCNA/RFC2/MAD2L2/RFC3 | | 0.015258 | | | | REACTOME |
| REACTOME_TRANSLESION_SYNTHESIS_BY_Y_FAMILY_DNA_POLYMERASES_BYPASSES_LESIONS_ON_DNA_TEMPLATE | -0.57586 | 0.000884 | POLI/RPA1/POLE3/UBA52/POLH/UFD1/POLD3/POLD2/POLD1/RPA3/RFC4/PCNA/RFC2/MAD2L2/POLE2/POLE/RFC3/PCLAF | | 0.010283 | | | | REACTOME |
| REACTOME_TRANSPORT_OF_MATURE_MRNAS_DERIVED_FROM_INTRONLESS_TRANSCRIPTS | -0.59991 | 7.07E-05 | CPSF2/NUP214/WDR33/NUP42/NUP155/NUP43/SYMPK/NUP160/NUP153/NUP58/NUP88/NUP210/NUP98/CPSF1/RAE1/POM121/AAAS/NUP54/NUP35/CPSF3/NXF1/NUP62/SEC13/POM121C/NDC1/NUP85/NUP188/NUP205/NUP37/NUP107/CPSF4/ALYREF | | 0.013425 | | | | REACTOME |
| REACTOME_TRANSPORT_OF_MATURE_TRANSCRIPT_TO_CYTOPLASM | -0.57108 | 1.53E-07 | WDR33/CHTOP/SRSF7/NUP42/SRRM1/SARNP/NUP155/NUP43/GLE1/SYMPK/NUP160/MAGOHB/NUP153/THOC5/THOC2/NUP58/ZC3H11A/NUP88/SRSF11/SRSF3/SRSF4/NUP210/NUP98/CPSF1/SRSF6/MAGOH/SRSF2/THOC1/RAE1/U2AF1/POM121/DDX39B/AAAS/NUP54/NUP35/EIF4A3/CPSF3/NXF1/NUP62/U2AF1L4/SRSF9/SEC13/POM121C/NDC1/U2AF2/NUP85/NUP188/NUP205/THOC6/NUP37/NUP107/DDX39A/CPSF4/ALYREF/NXT1 | | 0.002035 | | | | REACTOME |
| REACTOME_TRANSPORT_OF_THE_SLBP_DEPENDANT_MATURE_MRNA | -0.61064 | 0.000507 | NUP42/NUP155/NUP43/NUP160/NUP153/NUP58/NUP88/NUP210/NUP98/RAE1/POM121/AAAS/NUP54/NUP35/NXF1/NUP62/SEC13/POM121C/NDC1/NUP85/NUP188/NUP205/NUP37/NUP107/ALYREF | | 0.030628 | | | | REACTOME |
| REACTOME_TRNA_MODIFICATION_IN_THE_NUCLEUS_AND_CYTOSOL | -0.54738 | 0.000808 | TRIT1/FTSJ1/ADAT2/TRMT12/ADAT3/PUS3/WDR4/URM1/CTU2/NSUN2/TRMT112/THADA/TRMT61A/TPRKB/PUS1/TP53RK/QTRT2/THG1L/TRMT6/TRMT1/QTRT1/TYW1/CTU1/PUS7/METTL1 | | 0.00088 | | | | REACTOME |
| REACTOME_TRNA_PROCESSING | -0.45564 | 2.2E-05 | OSGEP/POP5/NUP50/RPP38/TRMT5/ELAC2/NUP214/TSEN54/TRMT13/CSTF2/PRORP/NUP42/TRMT10C/TRIT1/FTSJ1/NUP155/NUP43/ADAT2/NUP160/NUP153/HSD17B10/TSEN15/TRMT12/NUP58/RAN/ADAT3/PUS3/WDR4/NUP88/POP7/URM1/CTU2/ZBTB8OS/NUP210/NUP98/CPSF1/NSUN2/TRMT112/RAE1/TRMU/THADA/TRMT61A/GTPBP3/TPRKB/POM121/AAAS/PUS1/NUP54/NUP35/CLP1/TP53RK/NUP62/QTRT2/THG1L/SEC13/POM121C/NDC1/TRMT6/TRMT1/NUP85/NUP188/NUP205/QTRT1/TSEN34/TYW1/CTU1/RPP40/NUP37/NUP107/CPSF4/PUS7/RPPH1/METTL1 | | 0.00073 | | | | REACTOME |
| REACTOME_TRNA_PROCESSING_IN_THE_NUCLEUS | -0.52663 | 0.000175 | POP5/NUP50/RPP38/ELAC2/NUP214/TSEN54/CSTF2/NUP42/NUP155/NUP43/NUP160/NUP153/TSEN15/NUP58/RAN/NUP88/POP7/ZBTB8OS/NUP210/NUP98/CPSF1/RAE1/POM121/AAAS/NUP54/NUP35/CLP1/NUP62/SEC13/POM121C/NDC1/NUP85/NUP188/NUP205/TSEN34/RPP40/NUP37/NUP107/CPSF4/RPPH1 | | 0.002105 | | | | REACTOME |
| REACTOME_UCH_PROTEINASES | -0.39992 | 0.002623 | PSMD6/PSMB9/PSMF1/PSMB8/INO80E/HCFC1/PSME1/OGT/PSMD2/PSMA3/PSMC1/PSMA1/UBC/PSMB1/MCRS1/PSME4/PSMB2/PSMA5/PSMD9/PSMB4/NFRKB/PSMB7/ACTR5/PSMD11/PSMD14/PSMD3/PSMB6/PSMA6/PSMD13/PSMA4/PSMD12/PSMC4/TGFB1/PSMD5/PSMC2/H2AC6/PSMB3/UCHL3/UBA52/PSMA7/ASXL1/ADRM1/PSME2/BARD1/SEM1/PSMA2/ACTB/ACTL6A/H2AC11/RUVBL1/H2AC20/H2AC8 | | 0.001699 | | | | REACTOME |
| REACTOME_UNFOLDED_PROTEIN_RESPONSE_UPR | -0.51687 | 1.88E-06 | DNAJC3/CREB3L4/EXOSC2/EIF2S1/SRPRB/CEBPG/SRPRA/ACADVL/EXOSC9/SYVN1/CUL7/ASNS/CREB3L1/YIF1A/SHC1/ATF4/DIS3/DNAJB11/EXOSC8/KDELR3/ZBTB17/HYOU1/EXOSC4/EDEM1/DNAJB9/ERN1/PDIA6/DDIT3/LMNA/CALR/XBP1/ARFGAP1/FKBP14/WIPI1/MYDGF/HSP90B1/EXTL3/PDIA5/HSPA5/DDX11/ATF3/CEBPB/CCL2/IGFBP1/CXCL8 | | 0.008939 | | | | REACTOME |
| REACTOME_VIRAL_MESSENGER_RNA_SYNTHESIS | -0.58902 | 7.82E-05 | NUP50/POLR2F/GTF2F1/NUP214/NUP42/NUP155/NUP43/GTF2F2/NUP160/NUP153/POLR2G/NUP58/NUP88/POLR2J/NUP210/NUP98/RAE1/POM121/POLR2D/AAAS/NUP54/NUP35/NUP62/SEC13/POM121C/POLR2H/NDC1/NUP85/NUP188/NUP205/NUP37/NUP107 | | 0.00483 | | | | REACTOME |
| REACTOME_VITAMIN_D_CALCIFEROL_METABOLISM | -0.74804 | 0.018243 | CUBN/CYP24A1/CYP27B1/VDR | | 0.028146 | | | | REACTOME |
| REACTOME_VOLTAGE_GATED_POTASSIUM_CHANNELS | 0.810723 | 9.12E-07 | KCNC2/KCNS1/KCNA1/KCNQ5/KCNB1/KCNH5/KCNH7/KCNH3/KCNA5/KCNAB1/KCNH1/KCNC1/KCNAB2/KCNQ3/KCNA3/KCNA6/KCNC3/KCNC4/KCNA2/KCNQ2/KCNS3/KCNH8 | | 0.03632 | | | | REACTOME |
| WP_ATM_SIGNALING_PATHWAY | -0.66616 | 3.88E-06 | RAD9A/PIDD1/ABL1/H2AX/CCNE1/TP53/CASP2/JUN/BRCA1/CDK2/CHEK1/CDC25A/CHEK2/FANCD2/CCNB1/CDK1/MDM4/TP73/RAD51/NFKBIA/CDC25C | | 0.000635 | | | | WP |
| WP_BENZOAPYRENE_METABOLISM | 0.871416 | 0.023872 | AKR1C2/AKR1C1/AKR1C3/EPHX1 | | 0.001765 | | | | WP |
| WP_CELL_CYCLE | -0.54321 | 6.59E-09 | STAG1/CCNE2/ORC5/RB1/WEE1/SKP2/ANAPC11/TGFB3/CDKN1A/HDAC1/RBL1/CCND2/GADD45B/CDC25B/MCM6/ZBTB17/CDC7/MYC/MAD1L1/ABL1/E2F1/CCNE1/TP53/MCM3/ORC6/GADD45G/MCM5/CDK2/PCNA/MCM2/PTTG1/PKMYT1/MAD2L2/CHEK1/TTK/CDKN2C/CDC25A/PLK1/CCNA2/CHEK2/CCNB1/CDK4/CDK1/MCM7/DBF4/CCNB2/BUB1/CDC6/ORC1/E2F2/CDC20/ESPL1/CDC25C/CDC45/CDK6 | | 0.012359 | | | | WP |
| WP_CELLTYPE_DEPENDENT_SELECTIVITY_OF_CCK2R_SIGNALING | 0.816722 | 0.004562 | CCK/CCKBR/RYR2/ITPR1/PLCB1/RYR3/RYR1/GNAI1 | | 0.020662 | | | | WP |
| WP_DNA_DAMAGE_RESPONSE | -0.593 | 8.37E-07 | BID/CDKN1A/BBC3/FAS/CCND2/GADD45B/RAD9A/MYC/PMAIP1/PIDD1/ABL1/DDB2/H2AX/BAX/E2F1/CCNE1/TP53/CASP3/BRCA1/TNFRSF10B/GADD45G/CDK2/CHEK1/CDC25A/CHEK2/FANCD2/CCNB1/CDK4/CDK1/CCNB2/RAD51/CDC25C/CDK6 | | 0.003248 | | | | WP |
| WP_DNA_IRDAMAGE_AND_CELLULAR_RESPONSE_VIA_ATR | -0.61648 | 6.59E-09 | MDC1/HUS1/RMI1/RFWD3/USP1/UIMC1/RPA1/PPM1D/TOPBP1/CLK2/CEP164/WRN/BARD1/SEM1/RECQL/TDP1/RAD9A/TRIM28/TOP3A/H2AX/E2F1/TP53/RBBP8/BCL6/FEN1/BRCA1/CDK2/PCNA/MCM2/FANCA/BRCA2/FANCI/CHEK1/PLK1/CHEK2/FANCD2/CDK1/CLSPN/BRIP1/RAD51/EXO1/FOXM1/CDC25C/CDC45 | | 0.03632 | | | | WP |
| WP_DNA_IRDOUBLE_STRAND_BREAKS_AND_CELLULAR_RESPONSE_VIA_ATM | -0.61571 | 1.94E-06 | ACTL6A/RAD9A/TRIM28/ABL1/H2AX/BAX/E2F1/TP53/CASP3/BRCA1/BLM/PCNA/BRCA2/CHEK1/CHEK2/FANCD2/TP73/RAD51/EXO1/CDC25C | | 0.013215 | | | | WP |
| WP_DNA_REPAIR_PATHWAYS_FULL_NETWORK | -0.52024 | 8.35E-08 | MBD4/FANCF/GTF2H3/RFC5/PMS2/FANCM/ERCC1/MRE11/UNG/CUL4A/GTF2H4/POLD4/XRCC6/MLH1/PALB2/GTF2H2/RAD52/CDK7/CENPS/NTHL1/MSH6/HMGB1/CETN2/XRCC4/LIG3/USP1/POLI/APEX2/RPA1/XRCC1/APEX1/POLE3/POLH/GTF2H2C/FANCG/WRN/POLD3/MUTYH/POLD2/PNKP/TDG/DDB2/H2AX/POLM/FAAP100/FANCE/FANCB/POLD1/RAD54B/RPA3/FEN1/CENPX/BRCA1/RFC4/NEIL3/PCNA/RFC2/LIG1/FANCA/FANCC/BRCA2/FANCI/CHEK1/POLE2/POLE/FAAP24/RFC3/FANCD2/BRIP1/RAD51/EXO1 | | 0.010656 | | | | WP |
| WP_EUKARYOTIC_TRANSCRIPTION_INITIATION | -0.48862 | 0.007495 | GTF2B/GTF2E1/ERCC2/MNAT1/TAF12/TBP/TAF13/GTF2A2/TAF5/POLR3E/POLR2F/GTF2H3/POLR1A/GTF2H4/GTF2F2/GTF2H2/CDK7/POLR2G/TAF6/GTF2E2/POLR2J/TAF7/ILK/POLR1B/GTF2H2C/POLR3D/POLR1E/POLR2H | | 0.001477 | | | | WP |
| WP_GABA_RECEPTOR_SIGNALING | 0.874455 | 6.59E-09 | GABRB2/GABRA1/GABRG2/GABRA4/GABRA5/GABRG1/SLC32A1/GAD2/GABRA2/GABRD/GABRB3/GABRB1/GABBR1/GABBR2/GABRA3/SLC6A1/GAD1 | | 0.03632 | | | | WP |
| WP_GASTRIC_CANCER_NETWORK_2 | -0.70523 | 1.28E-05 | LBR/DSCC1/MYC/BRIX1/S100A6/TP53/PLAC8/UBE2T/ATAD2/COL9A3/RFC4/CHTF18/LMNB2/FANCI/RFC3/TOP2A/UBE2C/EGFR | | 0.005066 | | | | WP |
| WP_GPCRS_CLASS_C_METABOTROPIC_GLUTAMATE_PHEROMONE | 0.836498 | 0.004243 | GRM1/GRM5/GRM7/GRM2/GRM3/GABBR1/GABBR2 | | 0.01583 | | | | WP |
| WP_H19_ACTION_RBE2F1_SIGNALING_AND_CDKBETACATENIN_ACTIVITY | -0.71136 | 0.008505 | MED1/SOX4/TULP3/RB1/MACROH2A1/PMAIP1/E2F1/CSRP2/CDK4/JAG1/H19 | | 0.000442 | | | | WP |
| WP_MBDNF_AND_PROBDNF_REGULATION_OF_GABA_NEUROTRANSMISSION | 0.809362 | 5.01E-07 | GABRB2/GABRA1/GABRG2/GABRA4/GABRA5/SLC12A5/GABRG1/GABRA2/GABRD/GABRB3/GABRB1/GABRA3 | | 0.037458 | | | | WP |
| WP_METAPATHWAY_BIOTRANSFORMATION_PHASE_I_AND_II | 0.570509 | 0.000306 | | NDST3/SULT4A1/HS6ST3/AKR1C2/CYP4X1/AKR1C1/GSTO2/CYP39A1/AKR1C3/KCNAB1/GSTM5/HS3ST4/CYP46A1/CYP26B1/HS3ST2/KCNAB2/SULT1A1/NAT8L/INMT/CYP4F11/CYP7B1/CYP2E1/FMO2/CYP2J2/CYP11A1/EPHX1/CHST1/CYP2C8/AKR7A3/HS3ST5 | | 0.000336 | WP |  |  |
| WP_MIRNA_REGULATION_OF_DNA_DAMAGE_RESPONSE | -0.60303 | 8.17E-08 | BID/CDKN1A/BBC3/FAS/CCND2/GADD45B/RAD9A/MYC/PMAIP1/PIDD1/ABL1/DDB2/H2AX/BAX/E2F1/CCNE1/TP53/CASP3/MIR17HG/BRCA1/TNFRSF10B/GADD45G/CDK2/CHEK1/CDC25A/CHEK2/FANCD2/CCNB1/CDK4/CDK1/MCM7/CCNB2/RAD51/CDC25C/CDK6 | | 0.001303 | | | | WP |
| WP_MONOAMINE_GPCRS | 0.826861 | 2.08E-05 | CHRM1/HTR2A/HTR6/ADRA1A/ADRB1/ADRA1B/DRD1/CHRM4/ADRA2C/HTR1D/CHRM3/ADRB2/HTR1B/HRH2/ADRA2A/HTR2B/HTR7 | | 0.01358 | | | | WP |
| WP_MRNA_PROCESSING | -0.40439 | 0.000233 | HNRNPK/PABPN1/HNRNPU/HNRNPA1/SUGP2/SF3B1/SUGP1/CSTF3/DHX9/CPSF2/SF3A1/TMED10/CSTF2/SFSWAP/PRPF6/SRSF10/SRP54/PRPF4B/CSTF1/HNRNPC/SRSF7/SRRM1/PAPOLA/DHX15/SREK1/SNRPE/DHX8/SF3B2/HNRNPR/FUS/SNRPF/PCBP2/SF3B5/RBM39/CLK1/PRPF40A/CLK3/SRSF3/RBM5/SRSF4/CPSF1/SRSF6/SRSF2/DHX16/U2AF1L5/U2AF1/LSM2/PRPF3/NONO/HNRNPM/TRA2B/HNRNPL/SFPQ/CLK2/RBMX/SF3A3/PHF5A/SNRPD1/SF3B4/HNRNPD/CLP1/CPSF3/NXF1/CLASRP/SNRPA1/PPM1G/SRSF9/SNRPB2/HNRNPA2B1/XRN2/SNRNP40/SNRPD2/U2AF2/SNRPG/EFTUD2/PRPF4/SF3A2/DDX20/METTL3/SNRPA/SNRNP70/HNRNPAB/HNRNPH1/YBX1/PRMT1/PTBP1/CPSF4/SNRPB/LSM7 | | 0.007253 | | | | WP |
| WP_NUCLEOTIDE_EXCISION_REPAIR | -0.49052 | 0.003756 | GTF2H3/RFC5/ERCC1/CUL4A/GTF2H4/POLD4/GTF2H2/CDK7/CETN2/RPA1/POLE3/GTF2H2C/POLD3/POLD2/DDB2/POLD1/RPA3/RFC4/PCNA/RFC2/LIG1/POLE2/POLE/RFC3 | | 0.016397 | | | | WP |
| WP_NUCLEOTIDE_EXCISION_REPAIR_IN_XERODERMA_PIGMENTOSUM | -0.49854 | 0.000339 | GTF2H3/RFC5/ERCC1/CUL4A/GTF2H4/POLD4/SLX4/GTF2H2/H3-3A/CDK7/H4C8/CETN2/HMGN1/LIG3/RPA1/XRCC1/H2AC6/POLE3/H3-3B/XAB2/POLH/GTF2H2C/CHD1L/POLD3/H4C11/POLD2/DDB2/POLD1/RPA3/BRCA1/RFC4/RAD18/PCNA/RFC2/LIG1/POLE2/POLE/RFC3/H4C5 | | 0.000929 | | | | WP |
| WP_PHOSPHODIESTERASES_IN_NEURONAL_FUNCTION | 0.725767 | 1.01E-05 | GRIN1/GRIN2B/GRIN2A/PDE2A/PDE1A/DRD1/PPP1R1B/PDE1B/PDE11A/ADCY2/ADCY5/ADCY1/NOS1/GUCY1B1/GRIN2C/PDE10A/GUCY1A1/CHRFAM7A/CHRNA7/GUCY1A2/GRIA1/PDE8B/PDE3B/PDE5A/ADCY9 | | 0.02166 | | | | WP |
| WP_PHOTODYNAMIC_THERAPYINDUCED_UNFOLDED_PROTEIN_RESPONSE | -0.75599 | 5.46E-06 | ASNS/BBC3/ATF4/ERP27/DNAJB11/EDEM1/DNAJB9/ERN1/PDIA6/DDIT3/CALR/BCL2L11/XBP1/HSP90B1/PPP1R15A/HSPA5/ATF3/TRIB3 | | 0.030712 | | | | WP |
| WP_PKCGAMMA_CALCIUM_SIGNALING_PATHWAY_IN_ATAXIA | 0.770467 | 0.001351 | PRKCG/GRM1/ATP2B2/ITPR1/PLCB1/PLCB4/TRPC3/RYR3/GRIA2/GNA14/CACNA1A/GRIA1/CA8/GRIA4 | | 0.040013 | | | | WP |
| WP_PROTEASOME_DEGRADATION | -0.40605 | 0.02309 | PSMD6/PSMB9/PSMB8/PSME1/PSMD2/PSMA3/PSMC1/PSMA1/UBC/PSMB1/PSMB2/PSMA5/PSMD9/PSMB4/PSMB7/NEDD4/PSMD11/PSMD3/PSMB6/PSMA6/PSMD13/PSMA4/PSMD12/PSMC4/HLA-B/PSMD5/PSMC2/PSMB3/UCHL3/PSMA7/PSME2/PSMA2/RPN1/H2AZ1/HLA-A/RPN2/H2AX | | 0.045744 | | | | WP |
| WP_PYRIMIDINE_METABOLISM | -0.44585 | 0.000731 | POLR2J3/UMPS/POLR2J/PRIM1/CDA/POLE3/POLR2D/NME1-NME2/RRM1/POLR1B/NME2/POLR3D/CAD/POLR1E/POLA1/NME4/POLR2H/POLD3/UCKL1/POLD2/CTPS1/NT5C/NME1/POLR1C/PRIM2/POLD1/POLA2/DPYD/DTYMK/POLE2/POLE/POLR2J2/TYMP/TYMS/RRM2/UPP1/TK1 | | 0.040987 | | | | WP |
| WP_RAC1PAK1P38MMP2_PATHWAY | -0.46502 | 0.001237 | PXN/MMP2/NFKB1/TNIP2/RELA/ANGPTL1/MYC/ITGB1/BAX/GRB14/TP53/TNIP1/MAPK13/ANGPT2/DOK2/BIRC5/EIF4EBP1/FN1/RAD51/NFKBIA/NOS2/EGFR | | 0.00592 | | | | WP |

**Table S4.** A summary of the genes in the three Radscore-related modules generated by WGCNA

| **Module** | **Genes within the module** | |
| --- | --- | --- |
| Blue  (ME2) | A2ML1, AAMDC, ABCB8, ABCB9, ABCC8, ABCD2, ABCG4, ABHD3, ABHD8, ABLIM1, ABLIM2, ABRACL, AC000093.1, AC000123.2, AC002070.1, AC002310.4, AC002456.1, AC004233.2, AC004816.1, AC004947.2, AC005225.2, AC005330.1, AC005696.4, AC006001.3, AC006115.2, AC008771.1, AC008875.3, AC008966.1, AC009005.1, AC009102.2, AC009119.1, AC010501.2, AC010618.3, AC010624.2, AC010973.2, AC011008.2, AC012085.1, AC012510.1, AC012645.3, AC016590.3, AC016876.3, AC016924.1, AC017083.2, AC018638.5, AC018645.3, AC020915.2, AC021078.1, AC022167.2, AC026150.2, AC026273.1, AC026367.2, AC026403.1, AC027097.2, AC027307.2, AC048341.2, AC053503.4, AC066612.2, AC068205.2, AC068308.1, AC068631.2, AC074117.1, AC074212.1, AC079140.6, AC079848.1, AC080013.1, AC080038.1, AC083799.1, AC087289.5, AC091390.4, AC091729.3, AC092376.2, AC093673.1, AC093673.2, AC097382.3, AC098595.1, AC103691.1, AC104794.5, AC107398.3, AC113935.1, AC116565.1, AC120036.4, AC123768.3, AC129492.3, AC129507.1, AC132872.3, AC133644.2, AC135050.6, AC138393.1, AC138430.2, AC138649.1, AC139795.1, AC233723.2, AC244669.2, AC245297.1, ACAD11, ACADSB, ACAP2-IT1, ACBD7, ACKR3, ACO2, ACOT7, ACP6, ACSBG1, ACSL6, ACSS1, ACTB, ACTC1, ACTL6B, ACTR3B, ACTR3C, ACY1, ACYP2, ADAM11, ADAM19, ADAMTS8, ADAMTS9, ADARB1, ADARB2, ADAT3, ADCY1, ADCY2, ADCY5, ADD3, ADGRA1-AS1, ADGRD1, ADGRE5, ADGRV1, ADHFE1, ADIRF, ADPRHL1, ADRA1A, ADRA1B, ADRA2A, ADRA2C, ADRB1, AEN, AF106564.1, AF117829.1, AF131216.3, AFAP1L1, AFF3, AGAP2, AGAP3, AGTPBP1, AHCY, AHCYL1, AHCYL2, AIFM3, AJAP1, AJM1, AJUBA, AK2, AKAP11, AKAP13, AKAP5, AKAP6, AKNA, AKR1C1, AKR1C2, AKR1C3, AL022322.1, AL031056.1, AL034548.2, AL035413.1, AL049796.1, AL049840.6, AL078581.2, AL109809.4, AL117332.1, AL121658.1, AL121820.2, AL132656.4, AL132780.4, AL133520.1, AL158211.5, AL161772.1, AL162171.1, AL353660.1, AL353807.5, AL355297.3, AL355974.2, AL356056.2, AL359764.2, AL365361.1, AL390755.1, AL513327.1, AL589987.1, AL590714.1, AL606834.1, AL627309.5, AL662844.4, AL713852.1, AL731533.2, ALDH1A1, ALDH1A2, ALDH1L1-AS2, ALDH2, ALDH6A1, ALDOC, ALG2, ALG3, ALG5, ALG9, AMER2, AMIGO1, AMIGO3, AMN1, AMOT, AMPH, AMZ1, ANK1, ANK2, ANK3, ANKHD1-EIF4EBP3, ANKRD24, ANKRD33B, ANKRD34A, ANKRD39, ANKRD46, ANKS1B, ANO5, ANTXR2, ANXA5, AP000223.1, AP000280.1, AP000350.5, AP000356.5, AP001972.5, AP1S1, AP3B2, AP4M1, AP4S1, AP5Z1, APBA1, APBA3, APBB1, APOBEC3B, APOD, APRT, AQP11, AQP3, AQP4, ARF4, ARFGAP1, ARFGEF3, ARHGAP23, ARHGAP36, ARHGAP44, ARHGDIG, ARHGEF1, ARHGEF19, ARHGEF25, ARHGEF26-AS1, ARHGEF4, ARHGEF9, ARL4D, ARPC1A, ARPC2, ARPC4, ARPC5, ARPP19, ARPP21, ARRB1, ARSD, ART3, ARX, ASB2, ASIC2, ASPDH, ASPHD2, ATE1, ATF4, ATF7IP2, ATL1, ATP13A2, ATP1A2, ATP1A3, ATP1B1, ATP1B3, ATP2B1, ATP2B2, ATP5F1E, ATP5IF1, ATP5MPL, ATP6V0A1, ATP6V0C, ATP6V0E2, ATP6V1B2, ATP6V1C1, ATP6V1D, ATP6V1E1, ATP6V1FNB, ATP6V1G2, ATP7B, ATP9A, ATPAF1, ATRNL1, ATXN10, ATXN7L3B, AUXG01000058.1, B3GAT1, B3GNT9, B4GALNT3, B4GALT4, B4GALT6, B4GALT7, B4GAT1, BACH1, BAG4, BAIAP2-DT, BAIAP3, BAK1, BASP1, BATF3, BAX, BAZ1A, BCAS3, BCAS4, BCKDK, BCL10, BCL11A, BCL2L12, BCL2L2, BCL7C, BCR, BDH1, BEAN1, BEGAIN, BEND3P3, BEND6, BEX1, BEX2, BEX4, BEX5, BFSP1, BICDL1, BID, BIRC2, BLCAP, BLOC1S4, BLVRA, BMP1, BOC, BOLA2B, BOLA3-AS1, BORCS8-MEF2B, BRINP1, BRIX1, BRMS1, BRMS1L, BRSK1, BRSK2, BRWD1, BSN, BTBD6, BTBD8, BTBD9, BTG3, BTN2A3P, BTRC, BUD31, BX255925.3, C11orf24, C12orf45, C12orf76, C14orf119, C14orf132, C15orf39, C16orf74, C16orf89, C17orf49, C19orf44, C19orf54, C1orf115, C1orf52, C1QL2, C1QTNF4, C1QTNF6, C20orf96, C2CD4C, C2CD4D-AS1, C2orf15, C3orf14, C3orf80, C4orf47, C5orf15, C6orf47, C7orf31, C9orf129, C9orf16, C9orf24, CA10, CA11, CA4, CA8, CAB39L, CABLES1, CABP1, CACNA1A, CACNA1C, CACNA1G, CACNA2D1, CACNA2D2, CACNA2D3, CACNB1, CACNB2, CACNB3, CACNB4, CACNG8, CACYBP, CADM3, CADM3-AS1, CADPS, CADPS2, CALB1, CALB2, CALD1, CALM1, CALM3, CALN1, CALR, CALU, CALY, CAMK1D, CAMK1G, CAMK2A, CAMK2B, CAMK2G, CAMK2N2, CAMK4, CAMKK1, CAMKK2, CAMKV, CAMSAP3, CAP2, CAPN2, CAPS2, CARD8, CARMIL2, CARS1, CARS2, CASD1, CASKIN1, CASP6, CAVIN2, CBFA2T3, CBLB, CBLN1, CBLN2, CBX6, CBX7, CC2D1B, CCDC102A, CCDC107, CCDC130, CCDC136, CCDC144A, CCDC171, CCDC186, CCDC3, CCDC58, CCDC6, CCDC80, CCDC85A, CCDC92B, CCK, CCM2L, CCN4, CCNA1, CCNL2, CCNY, CCSAP, CCSER2, CCT6P3, CCZ1, CCZ1B, CD151, CD200, CD276, CD9, CD99, CDH13, CDH22, CDH8, CDHR1, CDIP1, CDK11A, CDK2AP2, CDK5R1, CDK5R2, CDK5RAP3, CDKL1, CDKL2, CDKN2D, CDS1, CDYL2, CEBPG, CELF2, CELF3, CELF4, CELF5, CELF6, CEND1, CEP170B, CFAP44, CFAP58-DT, CGAS, CGNL1, CGREF1, CHCHD5, CHCHD6, CHD1, CHD3, CHD5, CHEK2, CHGA, CHGB, CHIC1, CHID1, CHN1, CHPF, CHPF2, CHRD, CHRM1, CHRM3, CHRM4, CHRNA7, CHRNB2, CHST1, CHST14, CIPC, CISD1, CIT, CKAP4, CKMT1A, CKMT1B, CLCN4, CLDN10, CLDN15, CLEC18B, CLEC2D, CLEC2L, CLEC9A, CLGN, CLIC4, CLIP3, CLSTN1, CLSTN2, CLSTN3, CLVS1, CLVS2, CMTM3, CMTM4, CMTM6, CNIH2, CNIH3, CNIH4, CNKSR2, CNPY4, CNRIP1, CNTN4, CNTNAP1, CNTNAP2, COG1, COG7, COL22A1, COL26A1, COMMD6, COQ2, CORO1B, CORO6, CORT, COTL1, COX7A1, CPE, CPEB1, CPLX1, CPLX2, CPNE1, CPNE6, CPNE7, CRABP1, CRACDL, CREB3L2, CREG2, CRELD2, CRHBP, CRHR1, CRIM1-DT, CRLS1, CRNDE, CRPPA, CRTC1, CRY2, CRYM, CSDC2, CSRP2, CT75, CTDSP1, CTHRC1, CTNNA2, CTNS, CTSK, CTU1, CTXN1, CX3CL1, CXCR5, CXorf38, CYFIP2, CYGB, CYP26B1, CYP2E1, CYP2R1, CYP46A1, CYP7B1, CYRIA, CYS1, CYTH2, DACH1, DACT3, DAD1, DAGLA, DALRD3, DAPK1, DAPK3, DAXX, DAZAP1, DBNL, DCHS2, DCLK1, DCTN1, DCUN1D5, DDHD2, DDN, DDN-AS1, DDOST, DDX25, DDX41, DDX56, DDX60L, DEAF1, DEGS2, DENND10, DENND11, DERL2, DGCR5, DGCR6, DGKB, DGKE, DGKG, DHCR24, DHX32, DHX34, DIRAS1, DIRAS2, DISP1, DISP2, DISP3, DIXDC1, DKK3, DLEU7, DLG2, DLG3, DLG4, DLGAP1, DLGAP1-AS2, DLGAP3, DLK2, DLX1, DLX5, DMGDH, DMRTC1B, DMTN, DNAJA4, DNAJC12, DNAJC3, DNM1, DNM3, DNPEP, DNTTIP1, DOC2A, DOC2B, DOCK3, DOCK9, DOCK9-DT, DOK6, DPM1, DPM3, DPP10, DPP10-AS1, DPP3, DPY19L2P1, DPYSL2, DSTN, DTNA, DTX2, DUSP26, DUSP4, DUSP6, DUSP8, DUSP9, DYNC1I1, DYNLL2, DYNLT1, DZIP1L, DZIP3, E2F4, ECE2, EDEM2, EEF1A2, EEF1AKMT4, EEPD1, EFCAB1, EFEMP2, EFNA3, EFNB3, EFR3B, EGFL7, EHD1, EHD3, EIF2AK4, EIF2S2, EIF3B, EIF3J-DT, EIF3M, EIF4A1, EIF4E3, EIF4EBP1, EIF5A, ELAPOR1, ELAVL2, ELAVL4, ELFN2, ELK1, ELMO1, ELMOD1, ELOVL4, ELOVL7, EMC1-AS1, EMG1, EMILIN1, EMILIN2, EML4, EMP1, EMX2, EMX2OS, ENC1, ENHO, ENO2, ENO3, EPB41L1, EPB41L4B, EPHA10, EPHA4, EPHA5, EPHB4, EPHB6, EPHX1, EPHX4, EPS15, ERBB4, ERC2, ERCC5, ERF, ERGIC3, ERICH3, ERN1, ETNPPL, ETV6, EVL, EXT2, EZH1, FAAP20, FABP3, FABP6, FAHD2B, FAIM2, FAM106A, FAM107A, FAM111A, FAM114A1, FAM126B, FAM131A, FAM131C, FAM135B, FAM13A, FAM13C, FAM149A, FAM155A, FAM162B, FAM163B, FAM167A, FAM169A, FAM171A1, FAM171B, FAM174B, FAM174C, FAM184A, FAM189A1, FAM217B, FAM219A, FAM234B, FAM241B, FAM243A, FAM47E, FAM50B, FAM66A, FAM81A, FAM86DP, FAM86EP, FAR2P3, FASTK, FAXDC2, FBL, FBLIM1, FBLL1, FBXL15, FBXL16, FBXL17, FBXL2, FBXL6, FBXO2, FBXO27, FBXO30-DT, FBXO4, FBXO41, FBXO44, FBXW7, FEM1C, FGD6, FGF12, FGF13, FGF17, FGF22, FGF7P6, FGF9, FGFR3, FHL3, FIRRE, FJX1, FKBP10, FKBP11, FKBP1B, FKBP7, FKBP9, FLII, FLRT2, FLT3LG, FNDC5, FO681492.1, FOCAD, FOXG1-AS1, FOXP4-AS1, FRMPD2, FRMPD2B, FRRS1L, FRS3, FSD1L, FUT9, FXYD1, FXYD7, FZD5, GABARAPL1, GABARAPL2, GABBR1, GABBR2, GABRA2, GABRA3, GABRA5, GABRB1, GABRB2, GABRB3, GABRD, GABRG2, GAD2, GAL3ST4, GALK1, GALNS, GALNT16, GALNT17, GALNT2, GALNT8, GALNT9, GANAB, GAR1, GARNL3, GAS5, GAS7, GDA, GDAP1, GDAP1L1, GET1, GFAP, GFOD1, GFRA2, GGA1, GGN, GLA, GLB1L, GLB1L3, GLRB, GLS2, GLT1D1, GLTPD2, GLUD1, GLYCTK, GMPPA, GMPPB, GNAI1, GNAI2, GNAL, GNAO1, GNAQ, GNAZ, GNB2, GNB5, GNG2, GNG3, GNG5, GNL2, GNL3, GNPTAB, GOLGA5, GOLGA7B, GOT1, GOT2, GPAM, GPC4, GPC5, GPN1, GPR108, GPR143, GPR158, GPR162, GPR176, GPR27, GPR37L1, GPR61, GPR85, GPRASP1, GPRASP2, GPRC5B, GPRIN1, GPT, GPX7, GRAMD1B, GRAMD1C, GRIA1, GRIK2, GRIN1, GRIN2A, GRIN2C, GRIP1, GRIP2, GRK3, GRM2, GRM3, GRM5, GRM8, GRWD1, GS1-124K5.4, GSDME, GSKIP, GSS, GSTK1, GSTM3, GSTM5, GSTO2, GTF2E2, GUCD1, GUCY1A1, GUCY1B1, GULP1, GUSB, H2AC20, H2AC8, H2BC11, H2BC15, H3-2, H3-3B, H3C10, H6PD, HABP4, HACE1, HAGH, HAPLN3, HAPLN4, HAS2, HBQ1, HDAC1, HDAC7, HECW1, HECW2, HEMK1, HENMT1, HERC1, HERC2P3, HERC3, HERC5, HESX1, HID1, HIF1A, HIF3A, HIGD1A, HINT3, HIVEP2, HK1, HLA-A, HLF, HM13, HMG20B, HMGA1, HMGCLL1, HNRNPC, HOPX, HOTAIRM1, HPCA, HPCAL1, HPCAL4, HPRT1, HRH2, HRH3, HRK, HS3ST2, HS3ST4, HS6ST2, HS6ST3, HSD17B6, HSP90B1, HSPA12A, HSPA5, HSPBAP1, HTATSF1P2, HULC, HYOU1, ICA1, ICAM5, ID2-AS1, ID3, ID4, IDI2-AS1, IDS, IFI44, IFNAR2, IFRD2, IFT43, IGFBP2, IGFL4, IGIP, IGSF21, IKBIP, IL10RB-DT, IL17D, IL34, IL7, IMPDH1, INA, INPP4A, INPP4B, INPP5F, INPP5J, INPPL1, INTS4, IPCEF1, IPO4, IQCA1, IQGAP2, IQSEC1, IQSEC2, IQSEC3, IRAG1, IRAK1, IRAK4, ISLR2, ISY1, ITGA7, ITGB3BP, ITGB5, ITIH2, ITIH5, ITPK1, ITPKA, ITPR1, IVNS1ABP, JAG1, JAG2, JAKMIP1, JCAD, JMJD6, JPH3, JPH4, KALRN, KAT2B, KATNA1, KBTBD11, KCNA1, KCNA3, KCNAB1, KCNAB2, KCNB1, KCNC1, KCNC2, KCNC3, KCNC4, KCNH1, KCNH3, KCNIP2, KCNIP4, KCNJ11, KCNJ4, KCNJ9, KCNK1, KCNK3, KCNK4, KCNMA1, KCNN1, KCNQ3, KCNQ5, KCNS3, KCTD1, KCTD17, KCTD2, KCTD4, KCTD8, KDELR2, KIAA0319, KIAA0513, KIAA1522, KIAA1549L, KIAA1671, KIF17, KIF1A, KIF26A, KIF3A, KIF3B, KIF3C, KIF5A, KIF5C, KIF9, KIFAP3, KIFBP, KIFC2, KIRREL1, KIRREL3, KIT, KLC1, KLF8, KLHDC1, KLHDC2, KLHDC9, KLHL2, KLHL26, KLHL3, KLHL35, KMT2B, KMT5C, KNDC1, KRCC1, KRT10, KRT8P12, KRTAP5-AS1, L1CAM, LAG3, LAMA4, LAMA5, LAMB2, LAMC1, LAMP5, LAPTM4A, LARGE1, LBX2-AS1, LCA5, LCN12, LDB2, LDHD, LEMD2, LFNG, LGI1, LGI4, LHFPL1, LHFPL5, LHX6, LIFR-AS1, LIMCH1, LIN37, LIN7B, LINC00174, LINC00205, LINC00294, LINC00339, LINC00595, LINC00632, LINC00634, LINC00641, LINC00672, LINC00844, LINC00943, LINC00963, LINC01232, LINC01411, LINC01485, LINC01535, LINC01625, LINC01750, LINC02245, LINC02352, LINC02449, LINC02604, LINC02716, LINC02875, LINGO1, LINGO3, LIPE-AS1, LIPT1, LITAF, LIX1, LMAN2, LMBRD2, LMCD1, LMF2, LMNA, LMO3, LMO7, LMTK2, LMTK3, LONP1, LONRF2, LOXL1, LOXL1-AS1, LOXL3, LPAR2, LPCAT1, LPCAT3, LPCAT4, LPGAT1, LPIN1, LRBA, LRFN5, LRP1, LRP11, LRRC10B, LRRC3B, LRRC46, LRRC59, LRRC7, LRRC73, LRRC8B, LRRTM4, LSM11, LSM5, LSM6, LTBP3, LY6E-DT, LY6H, LYAR, LYNX1, LYPD1, LYPD5, LYRM9, LYSMD2, LZTS3, MACIR, MACROD2, MADD, MAFG-DT, MAGEE1, MALSU1, MAMDC4, MAMSTR, MAN1B1, MANF, MAOA, MAP11, MAP1A, MAP2K1, MAP2K4, MAP3K10, MAP3K14, MAP3K9, MAP4, MAP6, MAP7D2, MAPK1, MAPK10, MAPK8IP1, MAPK8IP2, MAPT, MAPT-AS1, MARCHF4, MARVELD1, MAST3, MATK, MBLAC2, MCF2, MCF2L, MCF2L2, MCOLN1, MCTP1, MDK, MDM2, MDM4, ME1, MEF2A, MEF2C, MEG3, MEGF10, MEGF9, METTL1, METTL7B, MFSD10, MFSD13A, MFSD4A, MFSD6, MGAT3, MGAT4B, MGAT4C, MGAT5B, MGC16275, MGLL, MGST3, MIAT, MICAL2, MICU3, MIDN, MIEF2, MIPOL1, MIR124-1HG, MIR124-2HG, MIR137HG, MIR1915HG, MIR193BHG, MIR3936HG, MIR4500HG, MIR600HG, MIR7-3HG, MIRLET7A1HG, MLLT11, MLX, MMD, MMD2, MMP14, MMP17, MMP24, MN1, MOAP1, MOCS2, MOGS, MORC2, MORN4, MOSMO, MOSPD3, MPP2, MPPED1, MRAP2, MRAS, MRC2, MRGBP, MRO, MRPL17, MRPL32, MRPL36, MRPS30-DT, MRPS33, MRTFB, MSANTD3, MSMP, MSN, MSRB2, MTERF3, MTFP1, MTHFD2, MTMR10, MTMR7, MTSS2, MUC1, MXI1, MYBPC1, MYCBP, MYCBP2, MYD88, MYDGF, MYL3, MYL6, MYO5A, MYO9B, MYOM1, MYOM2, MYORG, MYRIP, MYT1L, N4BP2L1, N4BP3, NAALAD2, NADK, NAGLU, NALCN, NANS, NAP1L2, NAP1L3, NAP1L5, NAPB, NAPRT, NAT16, NAT8L, NAV3, NBEA, NBL1, NBPF8, NCAM2, NCDN, NCOA7, NCS1, NDFIP2, NDN, NDRG2, NDRG3, NDRG4, NDUFA4, NDUFA5, NDUFB8, NEBL, NEBL-AS1, NECAB1, NECAB2, NECAB3, NECAP1, NECTIN1, NEDD9, NEFH, NEFL, NEFM, NEGR1, NEK10, NEK6, NELL1, NELL2, NEU1, NEU3, NEURL1, NEUROD1, NEXMIF, NFATC1, NFE2L2, NFE2L3, NFKBIE, NGEF, NGRN, NHLRC1, NID1, NID2, NIPSNAP3B, NISCH, NKIRAS1, NMB, NME2, NME9, NMNAT2, NMT2, NNAT, NOP16, NOP53, NOP58, NOS1AP, NOTCH3, NPDC1, NPEPL1, NPIPA7, NPM2, NPM3, NPPC, NPTN, NPTX1, NPTX2, NPTXR, NPY, NPY1R, NR1D2, NR3C2, NRG3, NRGN, NRIP3, NRSN1, NRSN2, NRXN3, NSF, NSG1, NSG2, NSMAF, NSMF, NSUN7, NT5DC3, NT5E, NTM, NTMT1, NTN4, NTNG2, NTRK2, NTSR2, NUAK1, NUBP1, NUCB2, NUDT22, NUDT7, NUP205, NUP62, NWD1, NXT1, NYAP1, OAT, OBI1-AS1, OFD1, OGA, OGDHL, OGFOD2, OIP5-AS1, OLFM1, OMA1, OMG, OPCML, OPHN1, OPRL1, OS9, OSBP2, OSBPL1A, OST4, OSTC, OTUD7A, OXA1L, OXCT1, OXSM, P2RX5, P2RX6P, P2RY11, P2RY14, P3H1, P3H3, P3H4, P4HB, PABPC1L, PACRG, PACSIN1, PAH, PAK1, PAK3, PAK4, PAK6, PALM, PANX2, PAQR8, PARM1, PARP12, PART1, PBDC1, PC, PCBP3, PCCA-DT, PCDH20, PCDH7, PCDH8, PCDHA11, PCDHAC2, PCDHGA2, PCDHGA3, PCDHGA6, PCDHGB5, PCDHGC5, PCED1A, PCGF1, PCLO, PCMT1, PCOLCE, PCOLCE2, PCP4, PCP4L1, PCSK1N, PCSK2, PDAP1, PDE10A, PDE1A, PDE1B, PDE2A, PDE3B, PDE4A, PDE4DIP, PDE8B, PDGFC, PDIA3, PDIA4, PDIA6, PDLIM3, PDLIM7, PDP1, PDRG1, PDXP, PDYN, PDZD4, PDZD7, PEBP1, PEBP4, PEG13, PEG3, PELI3, PENK, PFKFB2, PFKM, PFN1, PFN2, PGAM1, PGAP4, PGBD5, PGLS, PGP, PGPEP1, PGRMC1, PHACTR3, PHC2, PHF24, PHKA2, PHLDA2, PHLDB3, PHYHD1, PHYHIP, PHYHIPL, PI4KA, PI4KAP2, PIDD1, PIEZO1, PIGCP1, PIGG, PIGT, PIK3CB, PIK3R1, PINK1, PINLYP, PIP5K1B, PIP5K1C, PITHD1, PITPNM2, PITPNM3, PKP2, PLAAT5, PLCB1, PLCB4, PLCH1, PLCL2, PLCXD3, PLD6, PLEKHA1, PLEKHA4, PLEKHA5, PLEKHA6, PLEKHA8P1, PLEKHF2, PLEKHG2, PLEKHG5, PLOD3, PLPP3, PLPPR2, PLPPR3, PLPPR4, PLSCR3, PLXNB2, PMM2, PNCK, PNKP, PNMA1, PNMA2, PNMA3, PNMA6A, PNMA8A, PNMA8B, PNMA8C, PNOC, POFUT2, POGLUT3, POLD1, POLD2, POLI, POLM, POLR2H, POLR2J, POLR2J4, POP4, POPDC2, POR, POSTN, PPAN, PPARGC1A, PPCDC, PPFIA2, PPFIA3, PPFIA4, PPIB, PPIL3, PPIP5K1, PPL, PPM1H, PPM1K, PPM1L, PPP1R12B, PPP1R13B, PPP1R14B, PPP1R14C, PPP1R1A, PPP1R1B, PPP1R26, PPP1R26-AS1, PPP1R35, PPP1R3E, PPP1R3F, PPP1R9A, PPP2R2C, PPP3CA, PPP3CB, PPP3R1, PPP4C, PPP4R1L, PPP4R4, PPRC1, PRDM11, PRDM16, PRDM16-DT, PRDM8, PRDX4, PRELP, PREPL, PRKACB, PRKAG2, PRKAG2-AS1, PRKAR1B, PRKAR1B-AS1, PRKAR2B, PRKCB, PRKCE, PRKCZ, PRKD2, PRKN, PRNP, PRODH, PRPF31, PRRT1, PRRT2, PRRX1, PRSS3, PRXL2A, PRXL2C, PSD, PSD2, PSD3, PSMA2, PSMA3, PSMA6, PSMC2, PSME1, PSME2, PSME4, PSTK, PTBP1, PTER, PTGES3L, PTGFRN, PTK2B, PTK7, PTOV1-AS2, PTP4A3, PTPN12, PTPN20, PTPN5, PTPRB, PTPRM, PTPRN, PTPRN2, PTPRO, PTPRR, PTPRT, PUM3, PVALB, PVT1, PXDN, PYGL, PYGM, QPCT, QPCTL, QSOX1, R3HDM1, R3HDM4, RAB11FIP4, RAB13, RAB15, RAB26, RAB27B, RAB33A, RAB34, RAB37, RAB38, RAB3A, RAB3C, RAB5IF, RAB6B, RABEP1, RABGAP1L, RACK1, RAET1E-AS1, RAI2, RALYL, RANBP3L, RANGAP1, RAP1GAP, RAP1GAP2, RAPGEF2, RAPGEF4, RAPGEFL1, RARA, RARA-AS1, RASAL1, RASD2, RASGEF1A, RASGRF2, RASGRP1, RASL10A, RBCK1, RBFOX1, RBFOX3, RBM11, RBM24, RBM28, RBM38, RBM39, RBM42, RBM6, RBMS1, RBP1, RBP4, RCAN1, RCAN2, RCC1L, RCN1, RDH5, RECQL5, REEP1, REEP2, REEP4, RELA, RELL2, RELN, REPS2, RER1, RERG, RERGL, REST, RFLNA, RFPL1S, RFT1, RFXANK, RGPD6, RGR, RGS20, RGS4, RGS7, RGS7BP, RGS8, RHBDF1, RHBDL1, RHOC, RHOF, RIDA, RIIAD1, RIMBP2, RIMS1, RIMS2, RIMS3, RIOK2, RIOK3, RIPOR2, RIPPLY2, RIT1, RIT2, RND1, RNF11, RNF114, RNF122, RNF150, RNF170, RNF175, RNF208, RNF212, RNF32, RNF6, RNPEP, RNPEPL1, ROBO3, ROPN1B, RORB, RPGR, RPH3A, RPL10, RPL11, RPL12, RPL13A, RPL17, RPL18, RPL18A, RPL18AP3, RPL22L1, RPL23, RPL27A, RPL28, RPL29, RPL36, RPL36A, RPL39, RPL6P27, RPLP0, RPLP0P6, RPLP1, RPN1, RPN2, RPRML, RPS10, RPS11, RPS14, RPS16, RPS19, RPS2, RPS20, RPS23, RPS27, RPS27L, RPS28, RPS3A, RPS5, RPS6KA5, RPS7, RPS8, RPS9, RRAGA, RRBP1, RRP8, RRS1, RSKR, RSPO2, RTL10, RTN1, RTN2, RTN3, RTN4, RTN4R, RTN4RL2, RTP5, RTRAF, RUBCNL, RUNDC3A, RUNDC3B, RUNX1T1, RUSC2, RXRG, RYR1, S100A2, S100A3, S1PR1, S1PR2, S1PR3, SAMD12, SAMD9, SAP30, SASH1, SBF1, SCAMP5, SCAPER, SCG3, SCG5, SCN2A, SCN2B, SCN3B, SCN4B, SCN8A, SCOC, SCRT1, SDC1, SDF2L1, SEC11A, SEC16A, SEC16B, SEC24D, SEC61A1, SEC61B, SELENBP1, SELENON, SELENOS, SEM1, SEMA3G, SEMA4F, SEMA6B, SEPTIN3, SEPTIN5, SERINC1, SERINC2, SERP2, SERPINH1, SERPINI1, SERTAD4, SESN1, SESN2, SF3B5, SGCD, SGIP1, SGPP2, SGSH, SGSM1, SGTB, SH2B2, SH2D5, SH3BGRL2, SH3BP2, SH3BP5, SH3GL2, SH3GLB1, SHANK1, SHANK2, SHANK3, SHC1, SHE, SHISA5, SHISA9, SHISAL1, SHKBP1, SHMT2, SIDT1, SIL1, SINHCAF, SIRT7, SIVA1, SKIV2L, SLC12A4, SLC12A5, SLC12A8, SLC12A9, SLC13A3, SLC14A1, SLC15A2, SLC17A7, SLC18B1, SLC1A1, SLC1A2, SLC1A3, SLC1A4, SLC24A4, SLC25A18, SLC25A22, SLC25A23, SLC25A24, SLC25A27, SLC25A4, SLC25A42, SLC25A48, SLC25A5-AS1, SLC26A4-AS1, SLC26A6, SLC2A11, SLC2A12, SLC2A13, SLC2A1-AS1, SLC30A5, SLC35C1, SLC35C2, SLC35D2, SLC35F2, SLC38A10, SLC38A6, SLC39A1, SLC39A12, SLC39A7, SLC3A2, SLC41A3, SLC43A2, SLC43A3, SLC45A1, SLC4A10, SLC4A2, SLC6A1, SLC6A11, SLC6A13, SLC6A15, SLC6A17, SLC7A11, SLC7A14, SLC8A2, SLC8A3, SLC9A3R1, SLC9A6, SLC9A8, SLC9B2, SLCO1C1, SLIT2, SLITRK1, SLX1A, SMAP2, SMARCA2, SMG9, SMIM10L2A, SMIM10L2B, SMIM17, SMIM24, SMIM8, SMO, SMPD3, SNAI3-AS1, SNAP25, SNAP25-AS1, SNAP91, SNAPC1, SNAPC5, SNCA, SNCB, SNCG, SNHG11, SNHG12, SNHG14, SNHG15, SNHG16, SNHG28, SNHG3, SNHG5, SNHG9, SNORC, SNORD3B-1, SNPH, SNRPB, SNRPB2, SNRPD2, SNRPN, SNTA1, SNX32, SNX33, SNX5, SNX7, SOCS2, SOCS2-AS1, SOD1, SORBS1, SORBS2, SORCS1, SORCS2, SOWAHA, SPARCL1, SPATA17, SPEF2, SPIN1, SPIRE2, SPPL2A, SPRN, SPRY1, SPRY4, SPRY4-AS1, SPSB2, SPTB, SPTBN1, SPTBN2, SPTBN4, SPX, SRPK2, SRPX, SRRM3, SRRT, SRSF4, SRSF9, SSBP3, SSR4, SST, SSTR2, SSX2IP, ST20, ST6GALNAC5, ST8SIA3, STAC2, STAM, STAMBPL1, STAP2, STAT4, STAU2, STIM1, STK10, STK17A, STK19, STK32C, STK36, STK40, STMN1, STMN2, STMN3, STMN4, STOML1, STON2, STOX1, STOX2, STT3A, STUM, STX1A, STX1B, STX4, STXBP1, STXBP5, STXBP5L, STXBP6, STYXL1, SUCLA2, SULT4A1, SUMF2, SURF4, SV2A, SYBU, SYDE1, SYMPK, SYN1, SYN2, SYN3, SYNE1, SYNGR1, SYNGR3, SYNPR, SYP, SYS1, SYT1, SYT12, SYT13, SYT14, SYT16, SYT3, SYT4, SYT5, SYT7, SYTL5, SYVN1, SZRD1, TAC1, TAC3, TAFA2, TAGLN3, TAMALIN, TAPT1, TARBP2, TAX1BP3, TBC1D1, TBC1D24, TBC1D30, TBC1D9, TBCA, TBL2, TBL3, TBPL1, TBX15, TBX2-AS1, TCEAL2, TCEAL3, TCEAL5, TCEAL6, TCEAL7, TCEAL9, TCF7, TCTN1, TDRD9, TEAD2, TEAD3, TEF, TEKT2, TEN1, TENM2, TENM3, TENT5A, TEP1, TESPA1, TFAP2A-AS1, TGDS, TGFB1I1, TGFBR3, TGFBR3L, TGIF1, THADA, THBS3, THG1L, THRA, THRB, THSD4, TIMM9, TINF2, TLCD3B, TLCD4, TLE3, TLN2, TM9SF1, TMCO6, TMED1, TMED2, TMED3, TMED4, TMED9, TMEFF1, TMEFF2, TMEM102, TMEM115, TMEM121B, TMEM130, TMEM138, TMEM145, TMEM147, TMEM14A, TMEM150C, TMEM151B, TMEM155, TMEM158, TMEM161A, TMEM165, TMEM170B, TMEM179, TMEM179B, TMEM189, TMEM196, TMEM200A, TMEM208, TMEM214, TMEM216, TMEM240, TMEM25, TMEM251, TMEM258, TMEM271, TMEM38A, TMEM39A, TMEM43, TMEM45A, TMEM47, TMEM59L, TMEM60, TMEM63C, TMEM74B, TMEM99, TMOD2, TMSB10, TMTC1, TMUB1, TNC, TNIP2, TNKS1BP1, TNNI3K, TNNT1, TOM1L2, TOMM40, TOX2, TP53, TP53BP2, TP53I13, TP53RK, TPD52L1, TPM3P9, TPM4, TPRA1, TPRG1L, TPTE2P1, TRA2A, TRABD, TRABD2A, TRAF2, TRAF5, TRAM2, TRAPPC2B, TRAPPC6B, TRDC, TRIAP1, TRIB3, TRIL, TRIM14, TRIM17, TRIM2, TRIM23, TRIM4, TRIM47, TRIM5, TRIP10, TRMT112, TRNAU1AP, TRNP1, TRPM3, TRPT1, TRUB1, TSC22D1-AS1, TSC22D3, TSEN34, TSKU, TSNARE1, TSPAN10, TSPAN5, TSPAN7, TSPOAP1, TSPOAP1-AS1, TSPYL1, TSPYL2, TSPYL4, TSPYL5, TTBK2, TTC7B, TTC9, TTC9B, TTLL4, TUBA4A, TUBA8, TUBB2A, TUBB6, TUBG2, TUSC3, TVP23A, TVP23B, TXNDC11, TXNDC17, TXNDC5, TXNRD3, TYK2, TYRO3, U91328.1, UBA52, UBA6-AS1, UBALD2, UBE2E2, UBE2J2, UBE2QL1, UBR3, UCHL1, UCK2, UCKL1, UFD1, UFSP1, UIMC1, ULK1, UNC13A, UNC5A, UNC5B-AS1, UNC5D, UNC79, UNC80, UROS, USP12, USP2, USP32P1, USP36, USP46, VAMP1, VAMP2, VAT1, VAT1L, VCAN, VIPR1, VKORC1, VLDLR-AS1, VPS53, VSNL1, VSTM2A, VSTM2B, VSTM2L, VSX1, VWA5B2, VWC2, WAC-AS1, WASF1, WASF3, WASH6P, WDFY3-AS2, WDR11-AS1, WDR47, WDR60, WDR90, WFDC1, WFS1, WIPF1, WIPF3, WNK2, WNT7A, WSB2, WWP1, XKR4, YJEFN3, YKT6, YPEL2, YPEL4, YWHAG, YWHAH, Z70719.1, Z83844.2, Z99129.4, ZBTB16, ZBTB42, ZBTB47, ZC3H12B, ZC3HAV1, ZCCHC10, ZCCHC12, ZCCHC9, ZDHHC1, ZDHHC4, ZDHHC5, ZDHHC8P1, ZER1, ZFAND6, ZFHX2-AS1, ZFPM2, ZFR2, ZFYVE21, ZFYVE9, ZMAT4, ZNF143, ZNF204P, ZNF25, ZNF317, ZNF337, ZNF365, ZNF385B, ZNF391, ZNF433-AS1, ZNF480, ZNF483, ZNF503-AS2, ZNF529-AS1, ZNF540, ZNF559, ZNF575, ZNF587B, ZNF593, ZNF702P, ZNF768, ZNF783, ZNF837, ZNF846, ZNRF3, ZPR1, ZSCAN31, ZSWIM4, ZYX | |
| Yellow  (ME4) | AAAS, ABCA5, ABCA8, ABCD3, ABCF1, ABHD4, ABL1, ABLIM3, ABTB1, AC002451.1, AC002550.2, AC004943.2, AC005562.1, AC005833.1, AC006538.2, AC007066.2, AC007620.2, AC008124.1, AC008735.4, AC009118.2, AC009403.1, AC009902.3, AC010913.1, AC011447.3, AC011447.7, AC011468.2, AC011477.2, AC012073.1, AC012306.2, AC012447.1, AC012615.1, AC012640.2, AC015813.5, AC015813.6, AC015982.2, AC016582.2, AC016629.3, AC017104.1, AC018630.2, AC019069.1, AC021242.3, AC021739.2, AC022075.1, AC022150.2, AC022413.1, AC024293.1, AC025171.1, AC026401.3, AC026471.6, AC046185.3, AC064875.1, AC068473.5, AC068831.6, AC073046.1, AC073332.1, AC073343.2, AC073389.1, AC073508.3, AC073896.1, AC074135.1, AC074143.1, AC087632.2, AC090114.2, AC090692.1, AC091057.1, AC091057.4, AC092279.1, AC092718.4, AC092821.3, AC092919.2, AC092958.1, AC093620.1, AC093675.1, AC098484.4, AC099568.2, AC100810.1, AC104794.3, AC105020.4, AC106820.5, AC107294.2, AC108519.1, AC109460.2, AC110285.6, AC112777.1, AC114271.1, AC138207.8, AC234917.3, AC245060.4, ACAD8, ACIN1, ACKR1, ACLY, ACOX1, ACVR2A, ADAMTS6, ADAMTS7, ADAT1, ADCY6, ADNP, ADNP2, AGBL3, AGBL5, AGPAT1, AGRN, AIMP2, AK3P3, AKAP8, AKT1, AKT2, AL008707.1, AL031430.1, AL031985.3, AL078587.2, AL109918.1, AL117335.1, AL118558.3, AL121603.2, AL121832.2, AL133517.1, AL133523.1, AL135925.1, AL137060.1, AL355001.2, AL358472.6, AL359762.1, AL365181.3, AL512625.1, AL512785.2, AL513165.1, AL590666.2, AL627230.1, AL662797.1, AL669830.2, AL807752.3, ALDH1B1, ALDOA, ALG14, ALG6, ALKBH2, ALKBH4, ALS2CL, ALYREF, ANAPC1P2, ANAPC7, ANGPTL2, ANKFN1, ANKRD29, ANP32A, ANP32B, ANP32E, ANXA2R, ANXA3, AP000487.1, AP000880.1, AP1G2, APEX1, APOL4, ARAP3, ARC, ARHGAP11A, ARHGAP26, ARHGAP42, ARHGEF2, ARHGEF37, ARHGEF39, ARHGEF40, ARL10, ARL2BP, ARL4A, ARL6IP6, ARMC6, ARNTL2, ASF1B, ASIC4, ASPM, ASTE1, ASXL1, ATAD2, ATAD3A, ATAD5, ATP2A1-AS1, ATP6V0A2, ATRIP, AURKA, AURKB, AVPI1, B3GALNT2, B3GALT6, B3GNT10, B3GNT5, B3GNT7, B4GALT1-AS1, B4GALT2, B4GALT3, BARD1, BBOF1, BCHE, BCS1L, BEND7, BEST3, BHMT2, BICD1, BICRA, BIRC5, BIVM, BLM, BLMH, BMP7, BMP8B, BOD1, BOLA2-SMG1P6, BOP1, BORA, BPGM, BPHL, BPNT1, BRCA1, BRCA2, BRD3OS, BRPF1, BTF3L4P2, BTG3-AS1, BUB1, BUB1B, BX664615.1, BYSL, BZW2, C11orf98, C14orf93, C17orf107, C17orf58, C18orf21, C18orf54, C19orf48, C19orf57, C1GALT1C1L, C1orf112, C1orf226, C1orf35, C1orf50, C1orf94, C1QL1, C1QL4, C20orf194, C21orf58, C2orf27A, C2orf68, C2orf74, C4orf46, C5, C5orf34, C6orf118, C6orf52, C7orf26, C8orf33, C8orf82, C8orf88, CACFD1, CACNG4, CACTIN, CAD, CADM4, CAMK2N1, CAPN5, CARHSP1, CASK, CASP2, CASP3, CBS, CBX2, CBX3, CBX8, CBY1, CCAR1, CCAR2, CCDC120, CCDC134, CCDC137, CCDC138, CCDC14, CCDC142, CCDC15, CCDC150, CCDC167, CCDC169, CCDC18, CCDC28A, CCDC77, CCDC82, CCER2, CCHCR1, CCL3, CCL3L3, CCL4, CCL4L2, CCNA2, CCNB1, CCNB1IP1, CCNB2, CCND1, CCND2, CCNDBP1, CCNE1, CCNE2, CCNF, CCNJL, CCT2, CCT3, CCT5, CD24, CD2BP2, CD320, CDC123, CDC20, CDC25A, CDC25B, CDC25C, CDC34, CDC45, CDC6, CDC7, CDCA2, CDCA3, CDCA4, CDCA5, CDCA7, CDCA7L, CDCA8, CDH24, CDK1, CDK2, CDK4, CDK5RAP2, CDK6, CDKAL1, CDKN2C, CDKN3, CDR2L, CDT1, CENPA, CENPE, CENPF, CENPH, CENPI, CENPJ, CENPK, CENPL, CENPM, CENPN, CENPO, CENPP, CENPQ, CENPU, CENPW, CENPX, CEP126, CEP152, CEP295, CEP41, CEP55, CEP57, CEP57L1, CEP78, CEP83, CEP89, CFDP1, CHAC2, CHAF1A, CHAF1B, CHAMP1, CHCHD10, CHCHD3, CHD7, CHD8, CHEK1, CHERP, CHIC2, CHST11, CHST12, CHST3, CHST9, CHTF18, CIP2A, CITED1, CKAP2, CKAP2L, CKLF-CMTM1, CKMT2, CKS1B, CKS2, CLIP2, CLIP4, CLN5, CLSPN, CLYBL, CNKSR3, CNOT10, CNOT6, CNTRL, COG8, COL23A1, COL27A1, COL4A5, COL5A3, COPG2, COPZ1, CORO1C, COX10-AS1, CPNE2, CPNE8, CPSF3, CPSF4, CPXM1, CREB3L1, CREB3L4, CREB5, CREBL2, CRY1, CSE1L, CSMD2, CSNK1E, CSPG4, CSTF3, CTNNAL1, CTPS1, CTPS2, CU633906.2, CU634019.5, CU638689.5, CUEDC1, CUL4A, CYB5RL, DAG1, DBF4, DBF4B, DBI, DBN1, DBP, DCAF15, DCHS1, DCLK2, DCLRE1C, DCPS, DCTPP1, DCX, DDIAS, DDR1, DDX11, DDX11-AS1, DDX19A, DDX20, DDX23, DDX39A, DDX42, DDX49, DDX52, DDX55, DEK, DENND2B, DEPDC1, DEPDC1B, DEPTOR, DERPC, DESI2, DGCR8, DGKD, DGLUCY, DHFR, DHODH, DHPS, DHRS13, DHRS7B, DHRSX, DHX33, DHX57, DHX8, DIAPH1, DIAPH3, DIDO1, DIO2, DIO3OS, DKC1, DLEU1, DLEU2, DLGAP5, DLL3, DMKN, DNA2, DNAAF4, DNAAF5, DNAJB5, DNAJC17, DNMT1, DNMT3A, DOK4, DOLK, DOLPP1, DONSON, DOT1L, DPEP1, DPF2, DPH2, DPH7, DPYSL3, DPYSL5, DRAXIN, DSCC1, DSEL, DSN1, DTL, DTYMK, DUS2, DUS3L, DUSP12, DUSP2, DVL2, DYRK2, E2F1, E2F2, E2F3, E2F5, E2F6, E2F7, E2F8, ECHS1, ECT2, EDC3, EFCAB2, EFHD2, EFL1, EFNA2, EFNA5, EFNB1, EFR3A, EFS, EFTUD2, EGFEM1P, EGFR, EHMT1, EIF2B2, EIF3I, ELAC1, ELAVL1, ELP4, EMC10, EMC3-AS1, EME1, EML2, ENPP5, ENTR1, EPB41L4A-DT, EPCAM-DT, EPHB2, EPHB3, EPHX2, ERCC8, ERFE, ERGIC1, ERH, ESCO2, ESPL1, ESS2, ETV1, ETV4, EXO1, EXO5, EXOC4, EXOSC10, EXOSC2, EXOSC3, EXOSC8, EXTL2, EXTL3, EZH2, F10, FAAP100, FAAP24, FABP7, FADS1, FAF1, FAM110A, FAM110B, FAM111B, FAM120C, FAM122C, FAM133B, FAM182A, FAM182B, FAM200A, FAM27C, FAM27E3, FAM72B, FAM72D, FAM83D, FAM83H, FANCA, FANCC, FANCD2, FANCE, FANCG, FANCI, FARP1, FARP2, FBN3, FBXO46, FBXO5, FBXW8, FBXW9, FEN1, FERMT1, FGD1, FGFBP3, FHDC1, FHL1, FIBIN, FIGNL1, FLRT3, FLVCR1-DT, FMN1, FOSB, FOXD1, FOXD2-AS1, FOXD3-AS1, FOXM1, FOXO3B, FP565260.6, FREM2, FRMD8, FRMPD3, FSCN1, FUS, FYN, GABPB1, GABPB1-AS1, GALE, GAS1, GAS2L3, GASK1A, GBX2, GCDH, GCN1, GEMIN4, GEMIN6, GFER, GGH, GINS1, GINS2, GINS3, GINS4, GJC1, GLCCI1, GLDC, GLIDR, GLIPR2, GLIS2, GLT8D2, GLUL, GLYCTK-AS1, GMEB1, GMEB2, GMNN, GNA11, GNA12, GOLGA2P10, GOLGA8N, GOLM1, GPC1, GPC2, GPR161, GPR89A, GRIK3, GRINA, GSPT2, GSX1, GTF2F1, GTF2F2, GTF2H2C, GTF2H4, GTF2IRD1, GTF2IRD2B, GTF3C2, GTF3C5, GTF3C6, GTPBP4, GTSE1, H1-0, H19, H2AC11, H2AX, H2AZ1, H2AZ2, H3P6, HAS2-AS1, HASPIN, HAT1, HAUS1, HAUS5, HAUS6, HAUS8, HDAC2, HDGF, HDHD5, HEATR6, HELLS, HEY1, HGH1, HHAT, HHIPL1, HIP1, HIRA, HIRIP3, HJURP, HMBS, HMGB1, HMGB1P6, HMGB2, HMGB3, HMGN2, HMGN2P5, HMMR, HNF4G, HNRNPA1, HNRNPA1P16, HNRNPA1P48, HNRNPA2B1, HNRNPA3, HNRNPAB, HNRNPCP7, HNRNPD, HNRNPH1, HNRNPR, HNRNPUL1, HOXC4, HOXD3, HOXD-AS2, HPN, HROB, HSBP1, HSD17B10, HSD17B14, HSF4, HYAL2, HYKK, IARS1, IDH1, IDH2, IFT81, IGDCC3, IGF2BP2, IGF2BP3, IGSF3, IGSF9B, IL17RD, IL1RAP, ILF2, ILF3, ILF3-DT, IMPACT, INAVA, INCENP, ING1, ING3, ING4, INKA2, INKA2-AS1, INSM1, INSYN2A, INTS14, INTS5, INTS7, IPO5, IPO9, IQGAP3, IRAG1-AS1, IRAK1BP1, IRX1, ITPRIPL1, JADE2, JADE3, JAM3, JMJD1C-AS1, JMJD8, JPT1, JRK, KANSL2, KCNF1, KCTD15, KCTD21-AS1, KCTD3, KDM1A, KDM4B, KDM5B, KHSRP, KIAA1217, KIAA1549, KIF11, KIF14, KIF15, KIF18A, KIF18B, KIF20A, KIF22, KIF23, KIF26B, KIF2C, KIF4A, KIF7, KIFC1, KLF4, KLHDC8A, KLHL13, KLHL25, KLHL4, KLHL7, KNL1, KNOP1, KNSTRN, KNTC1, KPNA2, KRBA2, KRI1, KRT17, KTI12, LAMA1, LAPTM4B, LBH, LBR, LDAH, LDLRAD3, LENG8-AS1, LEO1, LHFPL3, LIG3, LIMA1, LIMD1, LIMD1-AS1, LIMD2, LIN9, LINC00461, LINC00511, LINC00526, LINC00654, LINC00665, LINC00882, LINC00928, LINC00957, LINC01003, LINC01159, LINC01410, LINC01550, LINC01560, LINC01578, LINC01637, LINC01670, LINC01778, LINC01816, LINC02361, LINC02588, LINC02802, LINC-PINT, LIX1L, LLGL2, LMAN2L, LMNB1, LMNB2, LPIN3, LRCH3, LRFN3, LRFN4, LRP5, LRP6, LRR1, LRRC36, LRRC37BP1, LRRC45, LRRC6, LRRN1, LRWD1, LSG1, LSM2, LSM7, LSM8, LTV1, LUC7L2, LY6E, LZIC, M1AP, MACROH2A1, MAD2L1, MAD2L2, MAGED1, MAGED2, MAGED4, MAGEF1, MANEAL, MAP3K1, MAP3K5, MAPK7, MAPKAP1, MARCKS, MARCKSL1, MASP1, MATN2, MAVS, MAZ, MBD3, MBTD1, MCAM, MCM10, MCM2, MCM3, MCM4, MCM5, MCM6, MCM7, MCM8, MDC1, MDFI, MDH2, MED1, MED19, MED22, MED26, MED27, MED30, MEF2C-AS2, MEGF11, MELK, MEN1, MEST, METTL2A, METTL3, MEX3A, MEX3B, MEX3D, MFAP3L, MFGE8, MFSD14C, MFSD9, MGME1, MICAL1, MICALL1, MINK1, MIR4453HG, MIR503HG, MIRLET7BHG, MIS12, MIS18A, MKI67, MKRN3, MKS1, MLXIPL, MMP15, MMP16, MMP2, MND1, MNS1, MPDU1, MPHOSPH9, MPPED2-AS1, MPST, MPV17L2, MRM2, MROH1, MRPL11, MRPL12, MRPL20-AS1, MRPS18A, MRTO4, MSH2, MSH6, MSI1, MSI2, MSTO2P, MT1G, MTBP, MTFR2, MTG2, MTHFD1, MTSS1, MUS81, MUTYH, MXD3, MYBL1, MYBL2, MYCL, MYCN, MYEF2, MYH7B, NAA40, NAE1, NASP, NBPF11, NBPF15, NBPF9, NCAPD2, NCAPD3, NCAPG, NCAPG2, NCAPH, NCAPH2, NCBP2, NCBP3, NCEH1, NCLN, NDC1, NDC80, NDE1, NEIL3, NEK2, NELFA, NELFCD, NEMP1, NES, NETO2, NEURL1B, NFIA, NFIB, NGDN, NIFK, NIP7, NIPAL2, NKIRAS2, NKX2-2, NLE1, NME1, NME1-NME2, NME5, NMRK1, NMU, NOC2L, NODAL, NOL11, NOL12, NOL9, NONO, NOP2, NOP56, NOTCH1, NOTCH4, NOVA1, NOXA1, NPAS3, NR1D1, NR2C2AP, NR4A2, NRARP, NRAV, NRF1, NRM, NSD2, NT5C3A, NT5DC2, NTHL1, NTN1, NUDCD2, NUDT1, NUDT14, NUDT5, NUF2, NUP107, NUP188, NUP210, NUP42, NUP43, NUP88, NUSAP1, NVL, ODC1, ODF2, OGFOD3, OGG1, OIP5, OPTN, OR7E7P, ORAI1, ORC1, ORC5, ORC6, OTUD1, PA2G4, PAFAH1B3, PAICS, PALM3, PANTR1, PAPLN, PARD3, PARD6G, PARP1, PARPBP, PASK, PATZ1, PAXBP1, PAXIP1, PBK, PBX3, PCCB, PCDH17, PCDHB10, PCDHB16, PCDHB9, PCDHGC3, PCDHGC4, PCGF2, PCIF1, PCLAF, PCNA, PDCD2, PDE7A, PDF, PDSS1, PES1, PFAS, PGAM5, PGBD1, PGD, PHACTR4, PHB, PHC1, PHETA1, PHF12, PHF13, PHF14, PHF19, PHF2, PHF5A, PHLDA1, PHOSPHO2, PHRF1, PHTF2, PIAS4, PIBF1, PIF1, PIGU, PIGW, PIK3R2, PIMREG, PINX1, PKMYT1, PKN3, PLA2G4C, PLA2G5, PLAAT3, PLAT, PLCE1, PLCG1-AS1, PLCXD1, PLEKHH3, PLEKHJ1, PLGRKT, PLK1, PLK4, PLPP5, PLXNA3, PMF1, PMPCA, PMS2CL, PMS2P1, PMS2P3, PNO1, PNPLA7, POC1A, POGLUT1, POGLUT2, POLA1, POLA2, POLD3, POLDIP3, POLE, POLE2, POLG2, POLR1C, POLR1E, POLR2F, POLR3D, POMGNT1, POMGNT2, POMK, POMT2, POU3F1, POU3F2, POU3F3, PPIL1, PPIL2, PPM1D, PPP1CC, PPP1R3C, PPP1R3G, PPP4R1, PPT2-EGFL8, PRC1, PRELID3B, PRIM1, PRIM2, PRKD1, PRKDC, PRMT1, PRMT5, PRMT6, PROM1, PROSER1, PROSER3, PRPF38A, PRPF4, PRPF6, PRR11, PRR19, PRRC2A, PRRT3-AS1, PRTG, PSKH1, PSMC3IP, PSMD11, PSMD3, PSMG3, PSRC1, PTCD1, PTGER4P2, PTMS, PTN, PTPRZ1, PTTG1, PUF60, PUS3, PUSL1, PXDC1, PXMP2, PYCR1, PYGO1, QRICH1, QSOX2, RAB11FIP5, RAB3IP, RAB7B, RACGAP1, RAD18, RAD51, RAD51AP1, RAD51D, RAD54B, RAD54L, RAF1, RAI1, RALY, RAN, RANBP1, RARS2, RASA4CP, RASSF7, RAVER1, RBBP8, RBBP9, RBFA, RBL1, RBM10, RBM15B, RBM3, RBM8A, RBMX, RBMX2, RCC1, RCL1, RCN2, RECQL4, REEP6, REPIN1, REV3L, REXO5, RFC2, RFC3, RFC4, RFC5, RFWD3, RFX5-AS1, RGL3, RGS14, RHBDD3, RILP, RIOK1, RMI1, RMI2, RN7SL1, RN7SL2, RNASEH1-AS1, RNASEH2A, RNASEH2C, RNF216, RNF216P1, RNF40, RNFT1, ROBO1, ROCK1P1, RP9, RPA1, RPA3, RPAIN, RPARP-AS1, RPL23AP42, RPL7AP6, RPP40, RPS2P46, RPS2P5, RPUSD1, RRM1, RRM2, RRP15, RRP9, RSU1, RTKN2, RUVBL1, RUVBL2, RYK, S100A13, S100PBP, SAAL1, SAC3D1, SALL1, SALL3, SAMD1, SAPCD2, SARM1, SCARF2, SCLT1, SCN1B, SDC3, SEC14L6, SELENOM, SEMA3B, SEMA4A, SEMA5A, SEMA5B, SEPHS1, SEPTIN9, SET, SETD1A, SETDB1, SF1, SF3A2, SFTA1P, SGO1, SGO2, SH3BP4, SH3D19, SH3PXD2B, SHCBP1, SIAH1, SIGMAR1, SIM2, SIRPA, SIRT6, SIX1, SKA1, SKA2, SKA3, SKP2, SLC16A2, SLC25A10, SLC25A15, SLC25A17, SLC25A25, SLC25A51, SLC25A53, SLC29A2, SLC29A4, SLC35A4, SLC35B2, SLC35F1, SLC37A3, SLC39A6, SLC52A2, SLC6A9, SLC7A8, SLIT3, SMARCA4, SMARCB1, SMARCC1, SMARCD1, SMC2, SMC4, SMC6, SMIM1, SMIM4, SMU1, SNAP29, SNCAIP, SNHG1, SNHG17, SNHG29, SNHG30, SNHG4, SNHG7, SNRNP200, SNRPA, SNRPD3, SNRPE, SORD2P, SOX11, SOX12, SOX13, SOX2, SOX3, SOX4, SOX6, SOX7, SOX9, SPAG5, SPATA33, SPATA6, SPATS2, SPC24, SPC25, SPDL1, SPECC1, SPIN4, SPINDOC, SPINK8, SPOCK1, SPOUT1, SPSB4, SQLE, SRC, SRI, SRPRB, SRR, SRSF10, SRSF3, SSPOP, SSRP1, ST7, ST7-AS1, ST7L, ST8SIA1, STEAP1B, STEAP2, STIL, STIMATE, STK32B, STMP1, STOML2, STON1, STX2, STXBP5-AS1, SULF2, SULT1A1, SUMO2, SUPT16H, SUPT20H, SUSD1, SUV39H1, SUV39H2, SUZ12P1, SYCE2, SYNE2, SYNPO2, TACC3, TAF15, TANC1, TANGO6, TBC1D7, TCF19, TCF3, TCF7L1, TCTN3, TDP1, TEDC1, TEDC2, TENM1, TEX10, TEX30, TEX9, TFAP2A, TFB2M, TFIP11, TGIF2, THAP9-AS1, THOC2, THOP1, THUMPD3-AS1, TICRR, TIGD5, TIGD7, TIMELESS, TIMM13, TIMM22, TIMM44, TIMM50, TIMM8B, TIMP4, TIPIN, TK1, TLCD3A, TLE2, TLE6, TMEM104, TMEM106C, TMEM131L, TMEM191A, TMEM209, TMEM218, TMEM229A, TMEM237, TMEM255A, TMEM51, TMEM79, TMEM97, TMOD4, TMPO, TMPO-AS1, TMSB15A, TMSB15B, TMSB15B-AS1, TNFRSF19, TNFRSF21, TNFRSF25, TNFSF12, TNFSF9, TNRC18, TOMM22, TOMM40P4, TONSL, TOP1MT, TOP2A, TOP3A, TOR1B, TOR3A, TP53I3, TP53INP1, TPCN1, TPRKB, TPX2, TRA2B, TRAF3IP2, TRAF4, TRAIP, TRDMT1, TRGC2, TRIB2, TRIM16, TRIM24, TRIM26, TRIM28, TRIM36, TRIO, TRIOBP, TRIP13, TRMT6, TRMT61A, TRMU, TROAP, TRRAP, TSEN15, TSPAN11, TSPAN12, TSPAN18, TSPAN6, TSR1, TTC30B, TTC36, TTC38, TTI1, TTK, TTLL1, TTLL12, TUBA1A, TUBA1B, TUBB, TUBB2B, TUBB4B, TUBD1, TUBG1, TUBGCP3, TUFM, TUG1, TULP3, TWNK, TXLNG, TYMS, U2AF2, UBA2, UBE2C, UBE2E3, UBE2I, UBE2N, UBE2S, UBE2SP1, UBE2T, UBIAD1, UBL7-AS1, UBTD2, UBTF, UBXN10-AS1, UBXN2A, UGDH, UGGT1, UHRF1, UHRF2, UMPS, UNG, UQCC3, URB2, USF1, USP1, USP18, USP19, USP21, USP42, UST, UTP11, UTP15, UTP4, VANGL2, VASH1, VASH2, VAV2, VAX2, VBP1, VEZF1, VHL, VMA21, VPS25, VPS37B, VPS72, VRK1, VSIG10, WAKMAR2, WASH5P, WDCP, WDHD1, WDR12, WDR34, WDR4, WDR46, WDR5, WDR54, WDR62, WDR74, WDR75, WDR76, WDR77, WDR92, WEE1, WIZ, WRAP53, WSCD1, WWC3, XPO5, XRCC1, XRCC2, XXYLT1, YAE1, YBX1, YBX1P1, YEATS4, YES1, YJU2, YPEL3, ZBED1, ZBED4, ZBED8, ZBTB12, ZBTB2, ZBTB26, ZBTB5, ZBTB7A, ZBTB8A, ZC2HC1C, ZC3H10, ZC3H4, ZC3HAV1L, ZC3HC1, ZCCHC3, ZDBF2, ZEB1-AS1, ZFP30, ZFP37, ZFP41, ZFP69B, ZFYVE28, ZGRF1, ZKSCAN5, ZKSCAN7, ZMYM1, ZMYND12, ZMYND19, ZNF114, ZNF121, ZNF124, ZNF137P, ZNF140, ZNF177, ZNF195, ZNF20, ZNF202, ZNF211, ZNF212, ZNF219, ZNF22, ZNF235, ZNF239, ZNF250, ZNF260, ZNF28, ZNF285, ZNF286A, ZNF3, ZNF300, ZNF311, ZNF320, ZNF324, ZNF324B, ZNF326, ZNF333, ZNF343, ZNF345, ZNF346, ZNF347, ZNF35, ZNF362, ZNF367, ZNF385A, ZNF398, ZNF410, ZNF419, ZNF428, ZNF432, ZNF433, ZNF439, ZNF446, ZNF45, ZNF462, ZNF467, ZNF470, ZNF473, ZNF48, ZNF486, ZNF491, ZNF496, ZNF501, ZNF502, ZNF512B, ZNF516, ZNF521, ZNF527, ZNF528-AS1, ZNF530, ZNF532, ZNF548, ZNF549, ZNF551, ZNF559-ZNF177, ZNF566, ZNF568, ZNF574, ZNF584, ZNF586, ZNF589, ZNF598, ZNF606, ZNF608, ZNF620, ZNF625, ZNF625-ZNF20, ZNF627, ZNF628, ZNF629, ZNF649, ZNF664, ZNF669, ZNF682, ZNF687, ZNF687-AS1, ZNF69, ZNF696, ZNF709, ZNF724, ZNF726, ZNF730, ZNF736, ZNF738, ZNF74, ZNF749, ZNF75A, ZNF775, ZNF788P, ZNF81, ZNF829, ZNF835, ZNF85, ZNF853, ZNF875, ZNF880, ZNF883, ZNF891, ZNF90, ZNF92, ZNF93, ZNRD2-AS1, ZSCAN12P1, ZSCAN16, ZSCAN21, ZSCAN9, ZWILCH, ZWINT, ZXDC | |
| Green (ME5) | | A1BG-AS1, AATK, ABCA2, ABHD12B, ABHD17B, ABHD6, ABTB2, AC004241.1, AC006059.1, AC007036.3, AC007114.1, AC009063.2, AC009090.3, AC009955.4, AC010359.2, AC010378.1, AC016597.1, AC018647.1, AC022007.1, AC023024.1, AC023024.2, AC024075.3, AC026691.1, AC027130.1, AC068580.4, AC068888.1, AC069209.2, AC092809.2, AC093330.1, AC102953.2, AC104083.1, AC105383.1, AC110285.1, AC117415.1, AC127459.3, AC131160.1, AC135050.3, AC142381.3, ACTN2, AD001527.2, ADAMTSL2, ADAP1, ADIPOR2, AFMID, AGBL2, AGPAT4, AK5, AL023284.4, AL031121.2, AL031651.2, AL033523.1, AL078590.2, AL118558.4, AL121655.1, AL133304.3, AL139353.1, AL160412.1, AL353751.1, AL354920.1, AL359091.1, AL365259.1, AL513477.1, AL591686.2, AL596244.1, AL662884.4, ALAD, ALCAM, AMOTL2, ANKRD65, ANLN, ANO4, AOPEP, AP000547.3, APLP1, APOLD1, ARHGAP21, ARHGAP22, ARHGEF26, ASPA, ASPHD1, ATP10B, ATP8A1, AZIN2, BACE1, BCAS1, BDNF-AS, BEST1, BIN1, BLOC1S2, BMP8A, BOK, BVES-AS1, BX284668.4, C10orf90, C1orf198, C21orf91, CABCOCO1, CAPN3, CARNS1, CBR1, CBR3, CCDC152, CCDC189, CCP110, CCT6A, CD22, CD27-AS1, CDC42EP2, CDK18, CDK19, CDKN1C, CERCAM, CES4A, CETN2, CFAP54, CFAP69, CFAP91, CFL2, CHADL, CHCHD2, CHN2, CLBA1, CLDN11, CLDND1, CLMN, CLN8, CMTM5, CNDP1, CNP, CNTF, CNTN2, CNTNAP4, COL21A1, CPB2-AS1, CPOX, CSRP1, CTNNA3, CYB5R2, CYP2J2, CYP2U1-AS1, CYTH1, DAAM2, DBNDD2, DESI1, DHRS9, DIPK1A, DLG1, DNAAF3, DNAH17, DNAH9, DNAJC2, DNAJC6, DOCK1, DOCK10, DOCK5, DOHH, DRC7, DSCAML1, DST, DTNB, DTX4, DYSF, DZANK1, EDIL3, EFHC1, EFHC2, EFHD1, EIF4HP2, ELAPOR2, ELOVL1, EMCN, ENDOD1, ENKD1, ENOX1, ENPP2, ENPP4, ENPP6, EPB41L2, EPB41L3, ERBB3, ERMN, ERMP1, EVI2A, EYA2, FA2H, FAAH, FAM102A, FAM107B, FAM124A, FAM178B, FAM181A-AS1, FAM201A, FAM222A, FAM53B, FAM86HP, FCHO1, FGFR2, FHIT, FMNL2, FN3K, FNTB, FOLH1, FOXJ1, FOXO4, FRA10AC1, FRMD4B, FRMD5, FUT8, FUT8-AS1, FZD3, GAL3ST1, GALNT13, GALNT7, GCK, GGCT, GJB1, GJC2, GLB1L2, GLCE, GLDN, GLTP, GOLGB1, GPD1, GPIHBP1, GPR37, GPR39, GPR62, GREM1, GRIK5, GSC, HAGLR, HAPLN2, HCN2, HDAC11, HDAC2-AS2, HES1, HHATL, HHIP, HMOX2, HMSD, HORMAD2-AS1, HOXB2, HOXD1, HSD17B3, HSD17B4, HSPA2, HYDIN, ICAM2, IFITM10, IFT140, IFT22, IGSF8, INF2, INHBB, INPP1, INSYN2B, IP6K3, IPO13, IQCG, IQCJ-SCHIP1, ISYNA1, JAKMIP3, KANK3, KAZN, KCNH8, KCNK10, KCNMB4, KDM2B-DT, KDM3A, KIAA0930, KIAA1755, KIF13B, KIF1C, KIF6, KLF13, KLHL32, KLK6, KRT17P1, KRT17P2, L3MBTL4, LACC1, LANCL1, LARP6, LCA5L, LCNL1, LDB3, LDLRAD4, LDLRAP1, LGI3, LGR5, LHPP, LILRA4, LINC00320, LINC00639, LINC00863, LINC00987, LINC01315, LINC01630, LINC02610, LINC02712, LIPE, LRP2, LRRC39, LRRC8C-DT, LURAP1, MAG, MAL, MAN2A2, MAP6D1, MAP7, MAPRE2, MATN1-AS1, MBNL2, MBP, MID1IP1, MINDY1, MINDY3, MINDY4, MOB3B, MOBP, MOG, MOK, MRPS6, MTURN, MTUS1, MVB12B, MYO18A, MYO1D, MYOT, MYOZ1, MYRF, NACAD, NANOS1, NEK11, NINJ2, NIPA1, NIPAL3, NIPAL4, NIPSNAP2, NKAIN2, NKX6-2, NPC1, NPHP1, NRBP2, NRIP2, NT5DC1, NUTM2A, NUTM2A-AS1, NUTM2D, NXPE3, OBSL1, OLMALINC, OPALIN, PACC1, PACS2, PADI2, PAIP2B, PAQR5, PAQR6, PBX4, PCSK6, PDE6B, PDIA2, PDK4, PDZRN3, PEX5L, PHACTR3-AS1, PHLDB1, PIEZO2, PIK3IP1, PIP4K2A, PKP4, PLA2G7, PLCH2, PLCL1, PLD1, PLEKHB1, PLEKHG3, PLEKHH1, PLLP, PLP1, PLPP2, PLXNB3, PMPCB, POLR2J3, POPDC3, PPA1, PPFIA1, PPFIBP2, PPP1R14A, PPP1R16B, PRCD, PREX1, PRIMA1, PRKCQ, PRKCQ-AS1, PRR18, PRRG1, PTGDS, PTPRD, PTPRH, PTPRK, PXK, QDPR, RAB36, RAB40B, RAP1GDS1, RAPGEF3, RAPGEF5, RASGEF1C, RASGRF1, RASGRP3, RASIP1, RBP7, RBX1, RFX2, RHBDL2, RHEB, RHOU, RINT1, RIPOR3, RNF125, ROGDI, RPS6KA2, RTKN, RUFY2, S100A1, S1PR5, SAMD15, SCD, SCOC-AS1, SDK1, SEC14L5, SEC61G, SECISBP2L, SELENOP, SEMA4D, SEMA7A, SEPTIN4, SEPTIN8, SERTAD4-AS1, SFTPC, SGK2, SGK3, SGMS1, SH2D3C, SH2D4A, SH3GL3, SH3GLB2, SH3PXD2A, SH3TC2, SHROOM3, SHROOM4, SHTN1, SIK3, SILC1, SIPA1L1, SIRT2, SLAIN1, SLC12A2, SLC22A15, SLC24A2, SLC2A10, SLC31A2, SLC44A1, SLC45A3, SLC48A1, SLC5A11, SLC9A3R2, SLCO1A2, SLCO3A1, SNX22, SNX30, SORT1, SOX10, SOX17, SOX2-OT, SPATA5L1, SPEG, SPNS2, SPOCK3, SRARP, SRCIN1, SSBP2, ST18, ST3GAL5, ST6GALNAC3, STAG3L4, STK39, SUN2, SVIP, SYNDIG1, SYNJ2, SYT9, TAF6, TBC1D12, TBC1D2, TCFL5, TCTN2, TEX2, TF, TGFA, TIAF1, TJP2, TLE4, TM6SF2, TMC7, TMCC2, TMCC3, TMEM125, TMEM139, TMEM144, TMEM151A, TMEM178A, TMEM235, TMEM248, TMEM63A, TMEM67, TMEM88B, TMEM98, TMIGD2, TMTC2, TMTC4, TNRC6C-AS1, TNXB, TP53INP2, TPPP, TPRN, TPTEP1, TRAM1L1, TRIM59, TRPV3, TSPAN15, TTC26, TTLL11, TTLL7, TTYH2, TUBB4A, TULP4, TXLNB, TYMSOS, UGT8, ULK2, UNC5C, USH1C, USP31, USP54, VWA1, VWA3B, Z83843.1, ZCCHC24, ZDHHC11, ZDHHC11B, ZDHHC14, ZDHHC2, ZDHHC20, ZEB2-AS1, ZFYVE16, ZNF334, ZNF488, ZNF536, ZNF701 |

**Table S5.** A summary of the pathways enriched by three Radscore-related modules

Enrichment analysis was performed using the R package clusterProfiler, querying the following annotated gene set databases: Kyoto Encyclopedia of Genes and Genomes (KEGG), Hallmark, Reactome, BioCarta, Pathway Interaction Database (PID), WikiPathways.

| **Pathway** | ***P* value** | **FDR** | **Genes** | **Count** | **Database** |
| --- | --- | --- | --- | --- | --- |
| BIOCARTA_AT1R_PATHWAY | 0.000128369 | 0.003383917 | CALM1/CALM3/ELK1/GNAQ/MAP2K1/MAP2K4/MAPK1/PAK1/PRKCB/PTK2B/SHC1 | 11 | BIOCARTA |
| BIOCARTA_ATRBRCA_PATHWAY | 3.62983E-05 | 0.000543493 | BRCA1/BRCA2/CHEK1/FANCA/FANCC/FANCD2/FANCE/FANCG/RAD51 | 9 | BIOCARTA |
| BIOCARTA_BAD_PATHWAY | 0.001516116 | 0.024850701 | ADCY1/BAX/KIT/MAPK1/PIK3R1/PRKACB/PRKAR1B/PRKAR2B/YWHAH | 9 | BIOCARTA |
| BIOCARTA_BARD1_PATHWAY | 2.13242E-07 | 4.72545E-06 | BARD1/BRCA1/FANCA/FANCC/FANCD2/FANCE/FANCG | 7 | BIOCARTA |
| BIOCARTA_BARR_MAPK_PATHWAY | 5.75768E-05 | 0.001863594 | ADCY1/ARRB1/DNM1/KCNA1/KCNA3/MAP2K1/MAPK1/PLCB1 | 8 | BIOCARTA |
| BIOCARTA_BARRESTIN_PATHWAY | 0.00131422 | 0.022860276 | ADCY1/ARRB1/DNM1/KCNA1/KCNA3/PLCB1 | 6 | BIOCARTA |
| BIOCARTA_BARRESTIN_SRC_PATHWAY | 0.000335986 | 0.008029119 | ADCY1/ARRB1/DNM1/KCNA1/KCNA3/MAP2K1/MAPK1/PLCB1 | 8 | BIOCARTA |
| BIOCARTA_BOTULIN_PATHWAY | 0.000875884 | 0.016839355 | CHRM1/SNAP25/STX1A/VAMP2 | 4 | BIOCARTA |
| BIOCARTA_CACAM_PATHWAY | 0.000163823 | 0.004231279 | CALM1/CALM3/CAMK1G/CAMK4/CAMKK1/CAMKK2 | 6 | BIOCARTA |
| BIOCARTA_CALCINEURIN_PATHWAY | 0.003076292 | 0.040969165 | CALM1/CALM3/GNAQ/NFATC1/PPP3CA/PPP3CB/PRKCB | 7 | BIOCARTA |
| BIOCARTA_CDC25_PATHWAY | 2.31621E-05 | 0.000356439 | CDC25A/CDC25B/CDC25C/CDK1/CHEK1/WEE1 | 6 | BIOCARTA |
| BIOCARTA_CELLCYCLE_PATHWAY | 6.48949E-08 | 1.65295E-06 | CCNB1/CCND1/CCND2/CCNE1/CDC25A/CDK1/CDK2/CDK4/CDK6/CDKN2C/E2F1/RBL1 | 12 | BIOCARTA |
| BIOCARTA_CK1_PATHWAY | 0.001368297 | 0.022867547 | CDK5R1/PLCB1/PPP1R1B/PPP3CA/PRKACB/PRKAR1B/PRKAR2B | 7 | BIOCARTA |
| BIOCARTA_CREB_PATHWAY | 0.002571059 | 0.035156134 | ADCY1/MAPK1/PIK3R1/PRKACB/PRKAR1B/PRKAR2B/PRKCB/RPS6KA5 | 8 | BIOCARTA |
| BIOCARTA_EFP_PATHWAY | 0.001319956 | 0.013862664 | CCNB1/CCNB2/CDK1/CDK2/CDK4/CDK6 | 6 | BIOCARTA |
| BIOCARTA_FBW7_PATHWAY | 0.00440283 | 0.040640443 | CCNE1/CDC34/CDK2/E2F1 | 4 | BIOCARTA |
| BIOCARTA_FCER1_PATHWAY | 0.001291108 | 0.022612072 | CALM1/CALM3/ELK1/MAP2K1/MAP2K4/MAPK1/NFATC1/PIK3R1/PPP3CA/PPP3CB/PRKCB/SHC1 | 12 | BIOCARTA |
| BIOCARTA_FLUMAZENIL_PATHWAY | 0.001902723 | 0.028321368 | GABRA2/GABRA3/GABRA5/PRKCE/SOD1 | 5 | BIOCARTA |
| BIOCARTA_FMLP_PATHWAY | 0.000317274 | 0.007653488 | CALM1/CALM3/CAMK1G/ELK1/MAP2K1/MAPK1/NFATC1/PAK1/PLCB1/PPP3CA/PPP3CB/RELA | 12 | BIOCARTA |
| BIOCARTA_G1_PATHWAY | 7.77824E-06 | 0.000131577 | ABL1/CCND1/CCNE1/CDC25A/CDK1/CDK2/CDK4/CDK6/DHFR/E2F1/SKP2 | 11 | BIOCARTA |
| BIOCARTA_G2_PATHWAY | 1.23825E-06 | 2.38604E-05 | BRCA1/CCNB1/CDC25A/CDC25B/CDC25C/CDC34/CDK1/CHEK1/PLK1/PRKDC/WEE1 | 11 | BIOCARTA |
| BIOCARTA_GPCR_PATHWAY | 1.40961E-05 | 0.000572124 | ADCY1/CALM1/CALM3/ELK1/GNAQ/MAP2K1/NFATC1/PPP3CA/PPP3CB/PRKACB/PRKAR1B/PRKAR2B/PRKCB | 13 | BIOCARTA |
| BIOCARTA_IGF1R_PATHWAY | 0.000754944 | 0.014964282 | ADCY1/MAP2K1/MAPK1/PIK3R1/PRKACB/PRKAR1B/PRKAR2B/SHC1/YWHAH | 9 | BIOCARTA |
| BIOCARTA_MCM_PATHWAY | 5.49333E-11 | 2.21331E-09 | CCNE1/CDC6/CDK2/CDT1/MCM2/MCM3/MCM4/MCM5/MCM6/MCM7/ORC1/ORC5/ORC6 | 13 | BIOCARTA |
| BIOCARTA_MEF2D_PATHWAY | 0.000542463 | 0.011277061 | CALM1/CALM3/CAPN2/HDAC1/NFATC1/PPP3CA/PPP3CB/PRKCB | 8 | BIOCARTA |
| BIOCARTA_NDKDYNAMIN_PATHWAY | 0.002090914 | 0.030206034 | CALM1/CALM3/DNM1/EPS15/NME2/PPP3CA/PPP3CB | 7 | BIOCARTA |
| BIOCARTA_NFAT_PATHWAY | 0.000572094 | 0.011797129 | CALM1/CALM3/CALR/CAMK1G/CAMK4/MAP2K1/MAPK1/MEF2C/NFATC1/PIK3R1/PPP3CA/PPP3CB/PRKACB/PRKAR1B/PRKAR2B | 15 | BIOCARTA |
| BIOCARTA_NOS1_PATHWAY | 7.40729E-07 | 3.94593E-05 | CALM1/CALM3/DLG4/GRIN1/GRIN2A/GRIN2C/PPP3CA/PPP3CB/PRKACB/PRKAR1B/PRKAR2B/PRKCB | 12 | BIOCARTA |
| BIOCARTA_P27_PATHWAY | 0.002991489 | 0.029203263 | CCNE1/CDK2/CKS1B/E2F1/SKP2 | 5 | BIOCARTA |
| BIOCARTA_P53_PATHWAY | 0.001319956 | 0.013862664 | CCND1/CCNE1/CDK2/CDK4/E2F1/PCNA | 6 | BIOCARTA |
| BIOCARTA_PAR1_PATHWAY | 0.000840605 | 0.016283534 | ADCY1/ARHGEF1/GNAQ/PIK3R1/PLCB1/PPP1R12B/PRKCB/PTK2B | 8 | BIOCARTA |
| BIOCARTA_PDZS_PATHWAY | 0.000542463 | 0.011277061 | ANK1/DLG2/DLG3/DLG4/GRIN1/NRXN3/PCLO/SPTBN1 | 8 | BIOCARTA |
| BIOCARTA_PGC1A_PATHWAY | 0.000110605 | 0.003107886 | CALM1/CALM3/CAMK1G/CAMK4/PPARGC1A/PPP3CA/PPP3CB/YWHAH | 8 | BIOCARTA |
| BIOCARTA_PTC1_PATHWAY | 0.001238008 | 0.013126441 | CCNB1/CDC25A/CDC25B/CDC25C/CDK1 | 5 | BIOCARTA |
| BIOCARTA_PYK2_PATHWAY | 0.000644433 | 0.012974925 | CALM1/CALM3/GNAQ/MAP2K1/MAP2K4/MAPK1/PAK1/PRKCB/PTK2B/SHC1 | 10 | BIOCARTA |
| BIOCARTA_RACCYCD_PATHWAY | 0.0009491 | 0.010309829 | AKT1/CCND1/CCNE1/CDK2/CDK4/CDK6/E2F1/RAF1 | 8 | BIOCARTA |
| BIOCARTA_RAN_PATHWAY | 0.005175362 | 0.045874411 | RAN/RANBP1/RCC1 | 3 | BIOCARTA |
| BIOCARTA_RANMS_PATHWAY | 2.7495E-06 | 4.99418E-05 | AURKA/KIF15/KPNA2/RAN/RANBP1/RCC1/TPX2 | 7 | BIOCARTA |
| BIOCARTA_RB_PATHWAY | 2.10241E-06 | 3.91508E-05 | CDC25A/CDC25B/CDC25C/CDK1/CDK2/CDK4/CHEK1/WEE1 | 8 | BIOCARTA |
| BIOCARTA_RNA_PATHWAY | 0.003436895 | 0.045299691 | DNAJC3/EIF2S2/MAP3K14/RELA/TP53 | 5 | BIOCARTA |
| BIOCARTA_SKP2E2F_PATHWAY | 0.000725234 | 0.008075971 | CCNE1/CDC34/CDK2/E2F1/SKP2 | 5 | BIOCARTA |
| BIOCARTA_SRCRPTP_PATHWAY | 0.001238008 | 0.013126441 | CCNB1/CDC25A/CDC25B/CDC25C/CDK1 | 5 | BIOCARTA |
| BIOCARTA_VDR_PATHWAY | 0.00278132 | 0.027763084 | CHAF1A/MED1/PRMT1/SMARCA4/SMARCC1/SMARCD1/SUPT16H | 7 | BIOCARTA |
| BIOCARTA_VIP_PATHWAY | 0.000453298 | 0.0101269 | CALM1/CALM3/GNAQ/NFATC1/PPP3CA/PPP3CB/PRKACB/PRKAR1B/PRKAR2B/RELA | 10 | BIOCARTA |
| HALLMARK_DNA_REPAIR | 2.11417E-07 | 4.72545E-06 | AAAS/ADCY6/ALYREF/CSTF3/DGCR8/ERCC8/FEN1/GTF2F1/GTF3C5/NCBP2/NELFCD/NME1/NT5C3A/PCNA/POLA1/POLA2/POLD3/POLR1C/POLR2F/PRIM1/RAD51/REV3L/RFC2/RFC3/RFC4/RFC5/RPA3/SAC3D1/SSRP1/TYMS/UMPS/VPS37B/ZWINT | 33 | HALLMARK |
| HALLMARK_E2F_TARGETS | 1.0467E-85 | 2.31949E-82 | ANP32E/ASF1B/ATAD2/AURKA/AURKB/BARD1/BIRC5/BRCA1/BRCA2/BUB1B/CCNB2/CCNE1/CDC20/CDC25A/CDC25B/CDCA3/CDCA8/CDK1/CDK4/CDKN2C/CDKN3/CENPE/CENPM/CHEK1/CKS1B/CKS2/CSE1L/CTPS1/DCTPP1/DDX39A/DEK/DEPDC1/DIAPH3/DLGAP5/DNMT1/DONSON/DSCC1/E2F8/ESPL1/EXOSC8/EZH2/GINS1/GINS3/GINS4/H2AX/H2AZ1/HELLS/HMGB2/HMGB3/HMMR/HNRNPD/ILF3/ING3/JPT1/KIF18B/KIF22/KIF2C/KIF4A/KPNA2/LBR/LMNB1/MAD2L1/MCM2/MCM3/MCM4/MCM5/MCM6/MCM7/MELK/MKI67/MSH2/MXD3/MYBL2/NASP/NCAPD2/NME1/NOP56/NUP107/ORC6/PA2G4/PAICS/PCNA/PHF5A/PLK1/PLK4/POLA2/POLD3/POLE/PPM1D/PRIM2/PRKDC/PSMC3IP/PTTG1/RACGAP1/RAD51AP1/RAN/RANBP1/RFC2/RFC3/RNASEH2A/RPA1/RPA3/RRM2/SMC4/SMC6/SPAG5/SPC24/SPC25/SSRP1/SUV39H1/TACC3/TCF19/TIMELESS/TIPIN/TK1/TMPO/TOP2A/TRA2B/TRIP13/TUBB/TUBG1/UBE2S/UBE2T/UNG/USP1/WEE1 | 126 | HALLMARK |
| HALLMARK_EPITHELIAL_MESENCHYMAL_TRANSITION | 0.001949608 | 0.028650274 | BASP1/BMP1/CALD1/CALU/CAP2/CTHRC1/EFEMP2/ENO2/GPX7/IGFBP2/ITGB5/LAMC1/LOXL1/LRP1/MAGEE1/MMP14/NID2/NT5E/NTM/P3H1/PCOLCE/PCOLCE2/PFN2/PLOD3/POSTN/PPIB/PRRX1/QSOX1/RGS4/SDC1/SERPINH1/SGCD/SLIT2/TGFBR3/TNC/TPM4/VCAN/WIPF1 | 38 | HALLMARK |
| HALLMARK_G2M_CHECKPOINT | 5.04652E-67 | 3.7277E-64 | ABL1/AURKA/AURKB/BARD1/BIRC5/BRCA2/BUB1/CCNA2/CCNB2/CCND1/CCNF/CDC20/CDC25A/CDC25B/CDC45/CDC6/CDC7/CDK1/CDK4/CDKN2C/CDKN3/CENPA/CENPE/CENPF/CHAF1A/CHEK1/CKS1B/CKS2/CUL4A/DBF4/DDX39A/DKC1/DTYMK/E2F1/E2F2/E2F3/EFNA5/ESPL1/EXO1/EZH2/FANCC/FBXO5/GINS2/H2AX/H2AZ1/H2AZ2/HIRA/HMGB3/HMGN2/HMMR/HNRNPD/ILF3/INCENP/JPT1/KIF11/KIF15/KIF22/KIF23/KIF2C/KIF4A/KNL1/KPNA2/LBR/LIG3/LMNB1/MAD2L1/MARCKS/MCM2/MCM3/MCM5/MCM6/MKI67/MYBL2/NASP/NDC80/NEK2/NSD2/NUSAP1/ODC1/ODF2/ORC5/ORC6/PBK/PLK1/PLK4/POLA2/POLE/PRC1/PRIM2/PRMT5/PTTG1/RACGAP1/RAD54L/RBL1/SMARCC1/SMC2/SMC4/SQLE/SRSF10/STIL/SUV39H1/TACC3/TMPO/TOP2A/TPX2/TRA2B/TRAIP/TROAP/TTK/UBE2C/UBE2S | 111 | HALLMARK |
| HALLMARK_GLYCOLYSIS | 0.001949608 | 0.028650274 | ARPP19/B3GAT1/B4GALT4/B4GALT7/CASP6/CHPF/CHPF2/CHST1/EFNA3/ENO2/EXT2/GALK1/GMPPA/GMPPB/GOT1/GOT2/GPC4/GUSB/HS6ST2/HSPA5/ME1/MXI1/NT5E/PC/PGAM1/PGLS/PHKA2/PKP2/PMM2/PPFIA4/PYGL/QSOX1/RBCK1/SAP30/SDC1/SOD1/STMN1/VCAN | 38 | HALLMARK |
| HALLMARK_MITOTIC_SPINDLE | 3.37691E-12 | 1.59218E-10 | ABL1/ARAP3/ARHGEF2/AURKA/BIRC5/BRCA2/BUB1/CCNB2/CDK1/CDK5RAP2/CENPE/CENPF/CENPJ/CEP57/CLIP2/CNTRL/DLGAP5/ECT2/ESPL1/FARP1/FBXO5/FSCN1/GEMIN4/INCENP/KIF11/KIF15/KIF22/KIF23/KIF2C/KIF4A/KNTC1/LMNB1/MARCKS/NDC80/NEK2/NUSAP1/PIF1/PLK1/PRC1/RACGAP1/SAC3D1/SEPTIN9/SMC4/TOP2A/TPX2/TRIO/TTK/TUBD1/TUBGCP3 | 49 | HALLMARK |
| HALLMARK_MYC_TARGETS_V1 | 4.48556E-19 | 4.14166E-17 | AIMP2/APEX1/CAD/CBX3/CCNA2/CCT2/CCT3/CCT5/CDC20/CDC45/CDK2/CDK4/CTPS1/DEK/ERH/H2AZ1/HDAC2/HDGF/HNRNPA1/HNRNPA2B1/HNRNPA3/HNRNPD/HNRNPR/IARS1/ILF2/KPNA2/LSM2/LSM7/MAD2L1/MCM2/MCM4/MCM5/MCM6/MCM7/NCBP2/NME1/NOP56/ODC1/PA2G4/PCNA/PHB/PSMD3/RAN/RANBP1/RFC4/RRM1/RRP9/RUVBL2/SET/SMARCC1/SNRPA/SNRPD3/SRSF3/TRA2B/TRIM28/TUFM/TYMS/UBA2/USP1/VBP1 | 60 | HALLMARK |
| HALLMARK_MYC_TARGETS_V2 | 9.27266E-12 | 4.10964E-10 | AIMP2/BYSL/CBX3/CDK4/DCTPP1/DUSP2/MCM4/MCM5/MRTO4/NIP7/NOP2/NOP56/PA2G4/PES1/PHB/PLK1/PLK4/RCL1/RRP9/SLC29A2/TFB2M/TMEM97/UNG/WDR74 | 24 | HALLMARK |
| HALLMARK_MYOGENESIS | 6.19578E-05 | 0.001932026 | ABLIM1/ACTC1/APOD/CAMK2B/CDH13/COX7A1/DTNA/ENO3/FABP3/FKBP1B/FLII/FXYD1/GABARAPL2/GNAO1/ITGA7/ITGB5/KCNH1/LARGE1/LPIN1/MEF2A/MEF2C/MRAS/MYL3/MYOM1/MYOM2/PC/PDE4DIP/PDLIM7/PFKM/PLXNB2/PPFIA4/PRNP/PTP4A3/PVALB/PYGM/REEP1/RIT1/RYR1/SGCD/SORBS1/TNNT1/TPD52L1/VIPR1 | 43 | HALLMARK |
| HALLMARK_P53_PATHWAY | 0.003553946 | 0.046364484 | AEN/BAK1/BAX/BLCAP/CDH13/CDK5R1/CYFIP2/EPHX1/ERCC5/FBXW7/FGF13/GLS2/IRAK1/JAG2/MDM2/PIDD1/PLXNB2/PVT1/RACK1/RPL18/RPL36/RPS27L/RRP8/SDC1/SEC61A1/SESN1/SLC3A2/SLC7A11/TAX1BP3/TP53/TPD52L1/TRIAP1/TRIB3/TSPYL2/WWP1/ZBTB16/ZNF365 | 37 | HALLMARK |
| HALLMARK_UNFOLDED_PROTEIN_RESPONSE | 0.002885183 | 0.038828483 | ARFGAP1/ATF4/CALR/CEBPG/DCTN1/DNAJA4/DNAJC3/EIF4A1/EIF4EBP1/ERN1/HSP90B1/HSPA5/HYOU1/MTHFD2/PDIA6/POP4/RPS14/SEC11A/SHC1/SLC1A4/SLC30A5/TSPYL2/TUBB2A/WFS1 | 24 | HALLMARK |
| KEGG_AMYOTROPHIC_LATERAL_SCLEROSIS_ALS | 7.64734E-05 | 0.002247614 | BAX/BID/DAXX/GRIA1/GRIN1/GRIN2A/GRIN2C/NEFH/NEFL/NEFM/PPP3CA/PPP3CB/PPP3R1/SLC1A2/SOD1/TOMM40/TP53 | 17 | KEGG |
| KEGG_APOPTOSIS | 0.002378817 | 0.033477736 | BAX/BID/BIRC2/CAPN2/CASP6/IRAK1/IRAK4/MAP3K14/MYD88/PIK3CB/PIK3R1/PPP3CA/PPP3CB/PPP3R1/PRKACB/PRKAR1B/PRKAR2B/RELA/TP53/TRAF2 | 20 | KEGG |
| KEGG_AXON_GUIDANCE | 2.97468E-05 | 0.001086607 | ABLIM1/ABLIM2/DPYSL2/EFNA3/EFNB3/EPHA4/EPHA5/EPHB4/EPHB6/GNAI1/GNAI2/L1CAM/MAPK1/NFATC1/NGEF/NTN4/PAK1/PAK3/PAK4/PAK6/PLXNB2/PPP3CA/PPP3CB/PPP3R1/RND1/ROBO3/SEMA3G/SEMA4F/SEMA6B/SLIT2/UNC5A/UNC5D | 32 | KEGG |
| KEGG_BASE_EXCISION_REPAIR | 4.26553E-08 | 1.11205E-06 | APEX1/FEN1/HMGB1/LIG3/MUTYH/NEIL3/NTHL1/OGG1/PARP1/PCNA/POLD3/POLE/POLE2/UNG/XRCC1 | 15 | KEGG |
| KEGG_CALCIUM_SIGNALING_PATHWAY | 4.44725E-07 | 2.47209E-05 | ADCY1/ADCY2/ADRA1A/ADRA1B/ADRB1/ATP2B1/ATP2B2/CACNA1A/CACNA1C/CACNA1G/CALM1/CALM3/CAMK2A/CAMK2B/CAMK2G/CAMK4/CHRM1/CHRM3/CHRNA7/ERBB4/GNAL/GNAQ/GRIN1/GRIN2A/GRIN2C/GRM5/HRH2/ITPKA/ITPR1/P2RX5/PDE1A/PDE1B/PHKA2/PLCB1/PLCB4/PPP3CA/PPP3CB/PPP3R1/PRKACB/PRKCB/PTK2B/RYR1/SLC25A4/SLC8A2/SLC8A3 | 45 | KEGG |
| KEGG_CELL_CYCLE | 3.7726E-23 | 6.96673E-21 | ABL1/ANAPC7/BUB1/BUB1B/CCNA2/CCNB1/CCNB2/CCND1/CCND2/CCNE1/CCNE2/CDC20/CDC25A/CDC25B/CDC25C/CDC45/CDC6/CDC7/CDK1/CDK2/CDK4/CDK6/CDKN2C/CHEK1/DBF4/E2F1/E2F2/E2F3/E2F5/ESPL1/HDAC2/MAD2L1/MAD2L2/MCM2/MCM3/MCM4/MCM5/MCM6/MCM7/ORC1/ORC5/ORC6/PCNA/PKMYT1/PLK1/PRKDC/PTTG1/RBL1/SKP2/TTK/WEE1 | 51 | KEGG |
| KEGG_DILATED_CARDIOMYOPATHY | 0.000618627 | 0.012554203 | ACTB/ACTC1/ADCY1/ADCY2/ADCY5/ADRB1/CACNA1C/CACNA2D1/CACNA2D2/CACNA2D3/CACNB1/CACNB2/CACNB3/CACNB4/CACNG8/ITGA7/ITGB5/LMNA/MYL3/PRKACB/SGCD/TPM4 | 22 | KEGG |
| KEGG_DNA_REPLICATION | 5.50836E-18 | 4.88261E-16 | DNA2/FEN1/MCM2/MCM3/MCM4/MCM5/MCM6/MCM7/PCNA/POLA1/POLA2/POLD3/POLE/POLE2/PRIM1/PRIM2/RFC2/RFC3/RFC4/RFC5/RNASEH2A/RNASEH2C/RPA1/RPA3 | 24 | KEGG |
| KEGG_ERBB_SIGNALING_PATHWAY | 0.002378817 | 0.033477736 | CAMK2A/CAMK2B/CAMK2G/CBLB/EIF4EBP1/ELK1/ERBB4/MAP2K1/MAP2K4/MAPK1/MAPK10/NRG3/PAK1/PAK3/PAK4/PAK6/PIK3CB/PIK3R1/PRKCB/SHC1 | 20 | KEGG |
| KEGG_GAP_JUNCTION | 0.000618627 | 0.012554203 | ADCY1/ADCY2/ADCY5/ADRB1/GNAI1/GNAI2/GNAQ/GRM5/GUCY1A1/GUCY1B1/ITPR1/MAP2K1/MAPK1/PDGFC/PLCB1/PLCB4/PRKACB/PRKCB/TUBA4A/TUBA8/TUBB2A/TUBB6 | 22 | KEGG |
| KEGG_GNRH_SIGNALING_PATHWAY | 0.001338816 | 0.022867547 | ADCY1/ADCY2/ADCY5/ATF4/CACNA1C/CALM1/CALM3/CAMK2A/CAMK2B/CAMK2G/ELK1/GNAQ/ITPR1/MAP2K1/MAP2K4/MAPK1/MAPK10/MMP14/PLCB1/PLCB4/PRKACB/PRKCB/PTK2B | 23 | KEGG |
| KEGG_HOMOLOGOUS_RECOMBINATION | 1.07957E-07 | 2.6795E-06 | BLM/BRCA2/EME1/MUS81/POLD3/RAD51/RAD51D/RAD54B/RAD54L/RPA1/RPA3/TOP3A/XRCC2 | 13 | KEGG |
| KEGG_LONG_TERM_DEPRESSION | 0.001000396 | 0.018808915 | CACNA1A/CRHR1/GNAI1/GNAI2/GNAO1/GNAQ/GNAZ/GRIA1/GRM5/GUCY1A1/GUCY1B1/ITPR1/MAP2K1/MAPK1/PLCB1/PLCB4/PRKCB/RYR1 | 18 | KEGG |
| KEGG_LONG_TERM_POTENTIATION | 3.77983E-08 | 2.92879E-06 | ADCY1/ATF4/CACNA1C/CALM1/CALM3/CAMK2A/CAMK2B/CAMK2G/CAMK4/GNAQ/GRIA1/GRIN1/GRIN2A/GRIN2C/GRM5/ITPR1/MAP2K1/MAPK1/PLCB1/PLCB4/PPP1R1A/PPP3CA/PPP3CB/PPP3R1/PRKACB/PRKCB | 26 | KEGG |
| KEGG_LYSINE_DEGRADATION | 0.00294211 | 0.029203263 | ALDH1B1/DOT1L/ECHS1/EHMT1/GCDH/NSD2/SETD1A/SETDB1/SUV39H1/SUV39H2 | 10 | KEGG |
| KEGG_MAPK_SIGNALING_PATHWAY | 0.00017306 | 0.004381325 | ARRB1/ATF4/CACNA1A/CACNA1C/CACNA1G/CACNA2D1/CACNA2D2/CACNA2D3/CACNB1/CACNB2/CACNB3/CACNB4/CACNG8/DAXX/DUSP4/DUSP6/DUSP8/DUSP9/ELK1/FGF12/FGF13/FGF17/FGF22/FGF9/FGFR3/MAP2K1/MAP2K4/MAP3K14/MAPK1/MAPK10/MAPK8IP1/MAPK8IP2/MAPT/MEF2C/MRAS/NTRK2/PAK1/PPP3CA/PPP3CB/PPP3R1/PRKACB/PRKCB/PTPN5/PTPRR/RAPGEF2/RASGRF2/RASGRP1/RELA/RPS6KA5/STMN1/TP53/TRAF2 | 52 | KEGG |
| KEGG_MELANOGENESIS | 0.001338816 | 0.022867547 | ADCY1/ADCY2/ADCY5/CALM1/CALM3/CAMK2A/CAMK2B/CAMK2G/CREB3L2/FZD5/GNAI1/GNAI2/GNAO1/GNAQ/KIT/MAP2K1/MAPK1/PLCB1/PLCB4/PRKACB/PRKCB/TCF7/WNT7A | 23 | KEGG |
| KEGG_MISMATCH_REPAIR | 7.25664E-07 | 1.47529E-05 | EXO1/MSH2/MSH6/PCNA/POLD3/RFC2/RFC3/RFC4/RFC5/RPA1/RPA3 | 11 | KEGG |
| KEGG_N_GLYCAN_BIOSYNTHESIS | 3.72493E-07 | 2.16469E-05 | ALG2/ALG3/ALG5/ALG9/DAD1/DDOST/DPM1/DPM3/GANAB/MAN1B1/MGAT3/MGAT4B/MGAT5B/MOGS/RFT1/RPN1/RPN2/STT3A/TUSC3 | 19 | KEGG |
| KEGG_NEUROACTIVE_LIGAND_RECEPTOR_INTERACTION | 0.002724061 | 0.037050129 | ADRA1A/ADRA1B/ADRA2A/ADRA2C/ADRB1/CHRM1/CHRM3/CHRM4/CHRNA7/CHRNB2/CRHR1/GABBR1/GABBR2/GABRA2/GABRA3/GABRA5/GABRB1/GABRB2/GABRB3/GABRD/GABRG2/GLRB/GRIA1/GRIK2/GRIN1/GRIN2A/GRIN2C/GRM2/GRM3/GRM5/GRM8/HRH2/HRH3/LPAR2/NPY1R/NTSR2/OPRL1/P2RX5/P2RY11/P2RY14/PRSS3/S1PR1/S1PR2/S1PR3/SSTR2/THRA/THRB/VIPR1 | 48 | KEGG |
| KEGG_NON_SMALL_CELL_LUNG_CANCER | 0.004589919 | 0.04168549 | AKT1/AKT2/CCND1/CDK4/CDK6/E2F1/E2F2/E2F3/EGFR/PIK3R2/RAF1 | 11 | KEGG |
| KEGG_NUCLEOTIDE_EXCISION_REPAIR | 4.19918E-05 | 0.000620359 | CUL4A/ERCC8/GTF2H4/PCNA/POLD3/POLE/POLE2/RFC2/RFC3/RFC4/RFC5/RPA1/RPA3 | 13 | KEGG |
| KEGG_OOCYTE_MEIOSIS | 0.000414511 | 0.004938474 | ADCY6/ANAPC7/AURKA/BUB1/CCNB1/CCNB2/CCNE1/CCNE2/CDC20/CDC25C/CDK1/CDK2/ESPL1/FBXO5/MAD2L1/MAD2L2/PKMYT1/PLK1/PPP1CC/PTTG1/SGO1 | 21 | KEGG |
| KEGG_P53_SIGNALING_PATHWAY | 3.28823E-05 | 0.000499091 | CASP3/CCNB1/CCNB2/CCND1/CCND2/CCNE1/CCNE2/CDK1/CDK2/CDK4/CDK6/CHEK1/GTSE1/PPM1D/RRM2/SIAH1/TP53I3 | 17 | KEGG |
| KEGG_PANCREATIC_CANCER | 0.00498704 | 0.044561616 | AKT1/AKT2/BRCA2/CCND1/CDK4/CDK6/E2F1/E2F2/E2F3/EGFR/PIK3R2/RAD51/RAF1 | 13 | KEGG |
| KEGG_PHOSPHATIDYLINOSITOL_SIGNALING_SYSTEM | 0.000133134 | 0.003473702 | CALM1/CALM3/CDS1/DGKB/DGKE/DGKG/INPP4A/INPP4B/INPP5J/INPPL1/ITPK1/ITPKA/ITPR1/PI4KA/PIK3CB/PIK3R1/PIP5K1B/PIP5K1C/PLCB1/PLCB4/PRKCB | 21 | KEGG |
| KEGG_PROGESTERONE_MEDIATED_OOCYTE_MATURATION | 6.18712E-05 | 0.000884559 | ADCY6/AKT1/AKT2/ANAPC7/BUB1/CCNA2/CCNB1/CCNB2/CDC25A/CDC25B/CDC25C/CDK1/CDK2/MAD2L1/MAD2L2/PIK3R2/PKMYT1/PLK1/RAF1 | 19 | KEGG |
| KEGG_PROSTATE_CANCER | 0.002756589 | 0.027640727 | AKT1/AKT2/CCND1/CCNE1/CCNE2/CDK2/CREB3L1/CREB3L4/CREB5/E2F1/E2F2/E2F3/EGFR/PIK3R2/RAF1/TCF7L1 | 16 | KEGG |
| KEGG_PYRIMIDINE_METABOLISM | 3.29619E-07 | 6.95654E-06 | CAD/CTPS1/CTPS2/DHODH/DTYMK/NME1/NME1-NME2/NME5/NT5C3A/POLA1/POLA2/POLD3/POLE/POLE2/POLR1C/POLR1E/POLR2F/POLR3D/PRIM1/PRIM2/RRM1/RRM2/TK1/TYMS/UMPS | 25 | KEGG |
| KEGG_RIBOSOME | 4.24933E-10 | 6.03641E-08 | RPL10/RPL11/RPL12/RPL13A/RPL17/RPL18/RPL18A/RPL22L1/RPL23/RPL27A/RPL28/RPL29/RPL36/RPL36A/RPL39/RPLP0/RPLP1/RPS10/RPS11/RPS16/RPS19/RPS2/RPS20/RPS23/RPS27/RPS27L/RPS28/RPS3A/RPS5/RPS7/RPS8/RPS9/UBA52 | 33 | KEGG |
| KEGG_SMALL_CELL_LUNG_CANCER | 0.000519434 | 0.006122686 | AKT1/AKT2/CCND1/CCNE1/CCNE2/CDK2/CDK4/CDK6/CKS1B/E2F1/E2F2/E2F3/LAMA1/PIAS4/PIK3R2/SKP2/TRAF4 | 17 | KEGG |
| KEGG_SPLICEOSOME | 3.99858E-08 | 1.06757E-06 | ACIN1/ALYREF/CHERP/DDX23/DDX42/DHX8/EFTUD2/HNRNPA1/HNRNPA3/LSM2/LSM7/LSM8/NCBP2/PHF5A/PPIL1/PRPF38A/PRPF4/PRPF6/PUF60/RBM8A/RBMX/SF3A2/SNRNP200/SNRPA/SNRPD3/SNRPE/SRSF10/SRSF3/THOC2/TRA2B/U2AF2 | 31 | KEGG |
| KEGG_TYPE_II_DIABETES_MELLITUS | 0.002421119 | 0.033829514 | ABCC8/CACNA1A/CACNA1C/CACNA1G/HK1/KCNJ11/MAPK1/MAPK10/PIK3CB/PIK3R1/PRKCE/PRKCZ/SOCS2 | 13 | KEGG |
| KEGG_VASCULAR_SMOOTH_MUSCLE_CONTRACTION | 0.001703395 | 0.026776573 | ADCY1/ADCY2/ADCY5/ADRA1A/ADRA1B/ARHGEF1/CACNA1C/CALD1/CALM1/CALM3/GNAQ/GUCY1A1/GUCY1B1/IRAG1/ITPR1/KCNMA1/MAP2K1/MAPK1/MYL6/PLCB1/PLCB4/PPP1R12B/PRKACB/PRKCB/PRKCE | 25 | KEGG |
| KEGG_VIBRIO_CHOLERAE_INFECTION | 0.001103926 | 0.020267014 | ACTB/ATP6V0A1/ATP6V0C/ATP6V0E2/ATP6V1B2/ATP6V1C1/ATP6V1D/ATP6V1E1/ATP6V1G2/KDELR2/PDIA4/PRKACB/PRKCB/SEC61A1/SEC61B | 15 | KEGG |
| PID_AR_PATHWAY | 0.001392894 | 0.014559684 | AKT1/BRCA1/CCND1/CDK6/HIP1/HNRNPA1/KDM1A/MED1/PA2G4/PATZ1/PIAS4/PRKDC/UBE2I | 13 | PID |
| PID_ATM_PATHWAY | 1.65927E-06 | 3.14269E-05 | ABL1/BLM/BRCA1/CDC25A/CDC25C/DCLRE1C/FANCD2/H2AX/MDC1/RBBP8/TOP3A/TRIM28/UBE2N | 13 | PID |
| PID_ATR_PATHWAY | 2.20122E-14 | 1.31835E-12 | ATRIP/BRCA2/CCNA2/CDC25A/CDC25C/CDC6/CDK2/CHEK1/CLSPN/FANCD2/MCM2/MCM7/PLK1/RAD51/RFC2/RFC3/RFC4/RFC5/RPA1/SSPOP/TIMELESS/TIPIN | 22 | PID |
| PID_AURORA_A_PATHWAY | 2.42387E-05 | 0.000370434 | AKT1/AURKA/AURKB/BIRC5/BRCA1/CDC25B/CENPA/DLGAP5/RAN/TACC3/TPX2 | 11 | PID |
| PID_AURORA_B_PATHWAY | 3.72601E-12 | 1.72018E-10 | AURKA/AURKB/BIRC5/BUB1/CDCA8/CENPA/INCENP/KIF20A/KIF23/KIF2C/KLHL13/NCAPD2/NCAPG/NCAPH/NDC80/PPP1CC/RACGAP1/SGO1/SMC2/SMC4 | 20 | PID |
| PID_BARD1_PATHWAY | 1.80677E-07 | 4.25937E-06 | BARD1/BRCA1/CCNE1/CDK2/FANCA/FANCC/FANCD2/FANCE/FANCG/PCNA/PRKDC/RAD51/RBBP8 | 13 | PID |
| PID_CIRCADIAN_PATHWAY | 0.000166873 | 0.002201135 | CHEK1/CRY1/CSNK1E/NONO/NR1D1/TIMELESS/WDR5 | 7 | PID |
| PID_E2F_PATHWAY | 3.57808E-15 | 2.20251E-13 | BRCA1/CCNA2/CCNE1/CCNE2/CDC25A/CDC6/CDK1/CDK2/CDKN2C/DHFR/E2F1/E2F2/E2F3/E2F5/E2F6/E2F7/MCM3/MYBL2/ORC1/POLA1/PRMT5/RANBP1/RBBP8/RBL1/RRM1/RRM2/TK1/TRIM28/TRRAP/TYMS/XRCC1 | 31 | PID |
| PID_FANCONI_PATHWAY | 2.87399E-14 | 1.63302E-12 | ATRIP/BLM/BRCA1/BRCA2/CHEK1/FAAP100/FAAP24/FANCA/FANCC/FANCD2/FANCE/FANCG/FANCI/H2AX/RFC2/RFC3/RFC4/RFC5/RMI1/RPA1/SSPOP/TOP3A/UBE2T/USP1 | 24 | PID |
| PID_FOXM1_PATHWAY | 4.5067E-14 | 2.49671E-12 | AURKB/BIRC5/BRCA2/CCNA2/CCNB1/CCNB2/CCND1/CCNE1/CDC25B/CDK1/CDK2/CDK4/CENPA/CENPF/CKS1B/FOXM1/GAS1/MMP2/NEK2/PLK1/SKP2/XRCC1 | 22 | PID |
| PID_LYSOPHOSPHOLIPID_PATHWAY | 0.000381555 | 0.008789512 | ADCY1/ADCY2/ADCY5/ADRA1B/ARHGEF1/GNAI1/GNAI2/GNAO1/GNAQ/GNAZ/GNG2/LPAR2/MAPT/PIK3CB/PIK3R1/PRKCE/PTK2B/RELA | 18 | PID |
| PID_MYC_ACTIV_PATHWAY | 0.000242229 | 0.003049885 | BIRC5/CAD/CCNB1/CCND2/CDC25A/CDCA7/CDK4/E2F3/NME1/ODC1/POLR3D/RCC1/RUVBL1/RUVBL2/TK1/TRRAP/UBTF | 17 | PID |
| PID_P53_REGULATION_PATHWAY | 0.003176622 | 0.030739712 | ABL1/AKT1/CCNA2/CDK2/CHEK1/CSE1L/CSNK1E/DYRK2/PPM1D/PRMT5/SKP2/TRIM28 | 12 | PID |
| PID_P73PATHWAY | 0.000743912 | 0.008242546 | ABL1/BRCA2/BUB1/CASP2/CCNA2/CCNB1/CCNE2/CDK1/CDK2/CDK6/CHEK1/IL1RAP/PLK1/RAD51/TP53I3/TUBA1A | 16 | PID |
| PID_PLK1_PATHWAY | 8.62487E-17 | 5.97272E-15 | AURKA/BORA/BUB1/BUB1B/CCNB1/CDC20/CDC25B/CDC25C/CDK1/CENPE/CENPU/CLSPN/ECT2/FBXO5/INCENP/KIF20A/NDC80/ODF2/PLK1/PRC1/SGO1/SPC24/SSPOP/TPX2/TUBG1/WEE1 | 26 | PID |
| PID_RB_1PATHWAY | 4.10791E-06 | 7.30754E-05 | ABL1/CCNA2/CCND1/CCND2/CCNE1/CDK2/CDK4/CDK6/DNMT1/E2F1/E2F2/E2F3/RAF1/SKP2/SMARCA4/SMARCB1/SUV39H1/UBTF | 18 | PID |
| PID_S1P_META_PATHWAY | 0.001822618 | 0.027740677 | GNAI1/GNAI2/GNAO1/GNAQ/GNAZ/S1PR1/S1PR2/S1PR3 | 8 | PID |
| PID_S1P_S1P2_PATHWAY | 0.001082521 | 0.020058011 | ELK1/GNAI1/GNAI2/GNAO1/GNAQ/GNAZ/MAPK1/PAK1/S1PR2 | 9 | PID |
| PID_SYNDECAN_3_PATHWAY | 0.001896362 | 0.01943825 | CASK/EGFR/FYN/PTN/SDC3/SRC | 6 | PID |
| PID_THROMBIN_PAR1_PATHWAY | 6.77739E-05 | 0.00201509 | AKAP13/ARHGEF1/ARRB1/DNM1/GNAI1/GNAI2/GNAO1/GNAQ/GNAZ/GNG2/GRK3/PIK3R1/PLCB1/PRKCB/ZYX | 15 | PID |
| REACTOME_ABERRANT_REGULATION_OF_MITOTIC_G1_S_TRANSITION_IN_CANCER_DUE_TO_RB1_DEFECTS | 1.86913E-07 | 4.35998E-06 | CCND1/CCND2/CCNE1/CCNE2/CDK2/CDK4/CDK6/E2F1/E2F2/E2F3 | 10 | REACTOME |
| REACTOME_ACETYLCHOLINE_NEUROTRANSMITTER_RELEASE_CYCLE | 2.46039E-08 | 2.02789E-06 | CPLX1/PPFIA2/PPFIA3/PPFIA4/RAB3A/RIMS1/SNAP25/STX1A/STXBP1/SYT1/TSPOAP1/VAMP2 | 12 | REACTOME |
| REACTOME_ACTIVATION_OF_ATR_IN_RESPONSE_TO_REPLICATION_STRESS | 1.00177E-21 | 1.30584E-19 | ATRIP/CDC25A/CDC25C/CDC45/CDC6/CDC7/CDK2/CHEK1/CLSPN/DBF4/MCM10/MCM2/MCM3/MCM4/MCM5/MCM6/MCM7/MCM8/ORC1/ORC5/ORC6/RFC2/RFC3/RFC4/RFC5/RPA1/RPA3 | 27 | REACTOME |
| REACTOME_ACTIVATION_OF_KAINATE_RECEPTORS_UPON_GLUTAMATE_BINDING | 0.001647496 | 0.026494639 | CALM1/DLG3/DLG4/GNB2/GNB5/GNG2/GNG3/GNG5/GRIK2/PLCB1 | 10 | REACTOME |
| REACTOME_ACTIVATION_OF_NIMA_KINASES_NEK9_NEK6_NEK7 | 0.001402557 | 0.014591857 | CCNB1/CCNB2/CDK1/PLK1 | 4 | REACTOME |
| REACTOME_ACTIVATION_OF_NMDA_RECEPTORS_AND_POSTSYNAPTIC_EVENTS | 7.12208E-10 | 9.58482E-08 | ADCY1/APBA1/CALM1/CAMK2A/CAMK2B/CAMK2G/CAMK4/CAMKK1/CAMKK2/DLG2/DLG3/DLG4/ERBB4/GRIA1/GRIN1/GRIN2A/GRIN2C/KIF17/LIN7B/LRRC7/MAPK1/MAPT/NBEA/NEFL/NRGN/PRKACB/PRKAG2/PRKAR1B/PRKAR2B/RASGRF2/TUBA4A/TUBA8/TUBB2A/TUBB6 | 34 | REACTOME |
| REACTOME_ACTIVATION_OF_THE_MRNA_UPON_BINDING_OF_THE_CAP_BINDING_COMPLEX_AND_EIFS_AND_SUBSEQUENT_BINDING_TO_43S | 2.35895E-06 | 0.000107712 | EIF2S2/EIF3B/EIF3M/EIF4A1/EIF4EBP1/RPS10/RPS11/RPS14/RPS16/RPS19/RPS2/RPS20/RPS23/RPS27/RPS27L/RPS28/RPS3A/RPS5/RPS7/RPS8/RPS9 | 21 | REACTOME |
| REACTOME_ACTIVATION_OF_THE_PRE_REPLICATIVE_COMPLEX | 1.91611E-22 | 3.03292E-20 | CDC45/CDC6/CDC7/CDK2/CDT1/DBF4/GMNN/MCM10/MCM2/MCM3/MCM4/MCM5/MCM6/MCM7/MCM8/ORC1/ORC5/ORC6/POLA1/POLA2/POLE/POLE2/PRIM1/PRIM2/RPA1/RPA3 | 26 | REACTOME |
| REACTOME_ADENYLATE_CYCLASE_INHIBITORY_PATHWAY | 0.003465455 | 0.045441886 | ADCY1/ADCY2/ADCY5/GNAI1/GNAI2/GNAL | 6 | REACTOME |
| REACTOME_ADRENALINE_NORADRENALINE_INHIBITS_INSULIN_SECRETION | 7.7226E-07 | 4.02994E-05 | ADCY5/ADRA2A/ADRA2C/CACNA1C/CACNA2D2/CACNB2/CACNB3/GNAI1/GNAI2/GNB2/GNB5/GNG2/GNG3/GNG5 | 14 | REACTOME |
| REACTOME_ADRENOCEPTORS | 0.001902723 | 0.028321368 | ADRA1A/ADRA1B/ADRA2A/ADRA2C/ADRB1 | 5 | REACTOME |
| REACTOME_ANCHORING_OF_THE_BASAL_BODY_TO_THE_PLASMA_MEMBRANE | 3.38077E-09 | 1.10173E-07 | CDK1/CDK5RAP2/CENPJ/CEP152/CEP41/CEP57/CEP78/CEP83/CEP89/CNTRL/CSNK1E/HAUS1/HAUS5/HAUS6/HAUS8/MKS1/NDE1/NEK2/ODF2/PLK1/PLK4/RAB3IP/SCLT1/TCTN3/TUBA1A/TUBB/TUBB4B/TUBG1 | 28 | REACTOME |
| REACTOME_APC_C_MEDIATED_DEGRADATION_OF_CELL_CYCLE_PROTEINS | 0.000101777 | 0.001400854 | ANAPC7/AURKA/AURKB/BUB1B/CCNA2/CCNB1/CDC20/CDK1/CDK2/FBXO5/MAD2L1/NEK2/PLK1/PSMD11/PSMD3/PTTG1/SKP2/UBE2C/UBE2S | 19 | REACTOME |
| REACTOME_APC_CDC20_MEDIATED_DEGRADATION_OF_NEK2A | 0.004559354 | 0.041578303 | ANAPC7/BUB1B/CDC20/MAD2L1/NEK2/UBE2C/UBE2S | 7 | REACTOME |
| REACTOME_AQUAPORIN_MEDIATED_TRANSPORT | 0.002213358 | 0.031617629 | ADCY1/ADCY2/ADCY5/AQP11/AQP3/AQP4/GNB2/GNB5/GNG2/GNG3/GNG5/PRKACB/PRKAR1B/PRKAR2B | 14 | REACTOME |
| REACTOME_ASPARAGINE_N_LINKED_GLYCOSYLATION | 1.01223E-08 | 8.92509E-07 | ALG2/ALG3/ALG5/ALG9/ANK1/ANK2/ANK3/ARF4/ARFGAP1/B4GALT4/B4GALT6/CALR/CNIH2/CNIH3/COG1/COG7/DAD1/DCTN1/DDOST/DERL2/DPM1/DPM3/DYNC1I1/DYNLL2/EDEM2/GANAB/GMPPA/GMPPB/GRIA1/KDELR2/LMAN2/MAN1B1/MGAT3/MGAT4B/MGAT4C/MOGS/NANS/NAPB/NEU1/NEU3/NSF/OS9/PDIA3/PMM2/RFT1/RPN1/RPN2/SEC16A/SEC16B/SEC24D/SLC35C1/SPTB/SPTBN1/SPTBN2/SPTBN4/ST6GALNAC5/ST8SIA3/STT3A/SYVN1/TMED2/TMED3/TMED9/TMEM115/TRAPPC6B/TUBA4A/TUBA8/TUBB2A/TUBB6/TUSC3/UBA52/YKT6 | 71 | REACTOME |
| REACTOME_ASSEMBLY_AND_CELL_SURFACE_PRESENTATION_OF_NMDA_RECEPTORS | 1.59524E-07 | 9.712E-06 | APBA1/CAMK2A/CAMK2B/CAMK2G/DLG2/DLG3/DLG4/GRIN1/GRIN2A/GRIN2C/KIF17/LIN7B/LRRC7/NBEA/NEFL/TUBA4A/TUBA8/TUBB2A/TUBB6 | 19 | REACTOME |
| REACTOME_AURKA_ACTIVATION_BY_TPX2 | 3.11286E-10 | 1.16917E-08 | AURKA/CDK1/CDK5RAP2/CENPJ/CEP152/CEP41/CEP57/CEP78/CNTRL/CSNK1E/HAUS1/HAUS5/HAUS6/HAUS8/HMMR/NDE1/NEK2/ODF2/PLK1/PLK4/TPX2/TUBA1A/TUBB/TUBB4B/TUBG1 | 25 | REACTOME |
| REACTOME_BASE_EXCISION_REPAIR | 7.55313E-07 | 1.5079E-05 | APEX1/FEN1/H2AX/H2AZ1/H2AZ2/LIG3/MUTYH/NEIL3/NTHL1/OGG1/PARP1/PCNA/POLD3/POLE/POLE2/RFC2/RFC3/RFC4/RFC5/RPA1/RPA3/UNG/XRCC1 | 23 | REACTOME |
| REACTOME_CA_DEPENDENT_EVENTS | 2.39664E-07 | 1.42516E-05 | ADCY1/ADCY2/ADCY5/CALM1/CAMK2A/CAMK2B/CAMK2G/CAMK4/CAMKK1/CAMKK2/MAPK1/NBEA/PDE1A/PDE1B/PRKACB/PRKAR1B/PRKAR2B | 17 | REACTOME |
| REACTOME_CA2_PATHWAY | 0.001796334 | 0.02767003 | CALM1/CAMK2A/FZD5/GNAO1/GNB2/GNB5/GNG2/GNG3/GNG5/ITPR1/NFATC1/PLCB1/PPP3CA/PPP3CB/PPP3R1/TCF7 | 16 | REACTOME |
| REACTOME_CALCINEURIN_ACTIVATES_NFAT | 0.001902723 | 0.028321368 | CALM1/NFATC1/PPP3CA/PPP3CB/PPP3R1 | 5 | REACTOME |
| REACTOME_CALNEXIN_CALRETICULIN_CYCLE | 0.002078785 | 0.030201434 | CALR/DERL2/EDEM2/GANAB/MAN1B1/OS9/PDIA3/SYVN1/UBA52 | 9 | REACTOME |
| REACTOME_CAMK_IV_MEDIATED_PHOSPHORYLATION_OF_CREB | 2.73386E-05 | 0.001028012 | CALM1/CAMK2A/CAMK2B/CAMK2G/CAMK4/CAMKK1/CAMKK2 | 7 | REACTOME |
| REACTOME_CARBOXYTERMINAL_POST_TRANSLATIONAL_MODIFICATIONS_OF_TUBULIN | 0.004147764 | 0.038946802 | AGBL3/AGBL5/TTLL1/TTLL12/TUBA1A/TUBA1B/TUBB2B/TUBB4B/VASH1/VASH2 | 10 | REACTOME |
| REACTOME_CARDIAC_CONDUCTION | 2.65669E-09 | 3.08779E-07 | AHCYL1/ATP1A2/ATP1A3/ATP1B1/ATP1B3/ATP2B1/ATP2B2/CACNA1C/CACNA2D2/CACNB1/CACNB2/CACNG8/CALM1/CAMK2A/CAMK2B/CAMK2G/FGF12/FGF13/FKBP1B/FXYD1/FXYD7/ITPR1/KAT2B/KCNIP2/KCNIP4/KCNJ11/KCNJ4/KCNK1/KCNK3/KCNK4/NPPC/RYR1/SCN2A/SCN2B/SCN3B/SCN4B/SCN8A/SLC8A2/SLC8A3/STIM1 | 40 | REACTOME |
| REACTOME_CDC6_ASSOCIATION_WITH_THE_ORC_ORIGIN_COMPLEX | 3.14235E-07 | 6.69562E-06 | CDC6/E2F1/E2F2/E2F3/MCM8/ORC1/ORC5/ORC6 | 8 | REACTOME |
| REACTOME_CELL_CYCLE | 7.09378E-76 | 7.85991E-73 | AAAS/ABL1/AKT1/AKT2/ANAPC7/ATRIP/AURKA/AURKB/BARD1/BIRC5/BLM/BORA/BRCA1/BRCA2/BUB1/BUB1B/CCNA2/CCNB1/CCNB2/CCND1/CCND2/CCNE1/CCNE2/CDC20/CDC25A/CDC25B/CDC25C/CDC45/CDC6/CDC7/CDCA5/CDCA8/CDK1/CDK2/CDK4/CDK5RAP2/CDK6/CDKN2C/CDT1/CENPA/CENPE/CENPF/CENPH/CENPI/CENPJ/CENPK/CENPL/CENPM/CENPN/CENPO/CENPP/CENPQ/CENPU/CENPW/CENPX/CEP152/CEP41/CEP57/CEP78/CHEK1/CHTF18/CKS1B/CLSPN/CNTRL/CSNK1E/DBF4/DHFR/DIDO1/DKC1/DNA2/DSCC1/DSN1/E2F1/E2F2/E2F3/E2F5/E2F6/ESCO2/ESPL1/EXO1/FBXO5/FEN1/FOXM1/GINS1/GINS2/GINS3/GINS4/GMNN/GTSE1/H2AX/H2AZ1/H2AZ2/HAUS1/HAUS5/HAUS6/HAUS8/HJURP/HMMR/INCENP/KIF18A/KIF20A/KIF23/KIF2C/KNL1/KNTC1/LBR/LIN9/LMNB1/LPIN3/MAD2L1/MCM10/MCM2/MCM3/MCM4/MCM5/MCM6/MCM7/MCM8/MDC1/MIS12/MIS18A/MND1/MYBL2/NCAPD2/NCAPD3/NCAPG/NCAPG2/NCAPH/NCAPH2/NDC1/NDC80/NDE1/NEK2/NSD2/NUF2/NUP107/NUP188/NUP210/NUP42/NUP43/NUP88/ODF2/OIP5/OPTN/ORC1/ORC5/ORC6/PCNA/PHLDA1/PIAS4/PIF1/PKMYT1/PLK1/PLK4/PMF1/POLA1/POLA2/POLD3/POLE/POLE2/POLR2F/PPP1CC/PRIM1/PRIM2/PSMC3IP/PSMD11/PSMD3/PTTG1/RAD51/RAN/RBBP8/RBL1/RCC1/RFC2/RFC3/RFC4/RFC5/RMI1/RMI2/RPA1/RPA3/RRM2/RUVBL1/RUVBL2/SET/SGO1/SGO2/SKA1/SKA2/SKP2/SMC2/SMC4/SPC24/SPC25/SPDL1/SRC/SYCE2/SYNE2/TK1/TMPO/TOP2A/TOP3A/TPX2/TUBA1A/TUBA1B/TUBB/TUBB2B/TUBB4B/TUBG1/TUBGCP3/TYMS/UBE2C/UBE2I/UBE2N/UBE2S/VRK1/WEE1/WRAP53/ZNF385A/ZWILCH/ZWINT | 221 | REACTOME |
| REACTOME_CELL_CYCLE_CHECKPOINTS | 1.29083E-36 | 4.76746E-34 | ANAPC7/ATRIP/AURKB/BARD1/BIRC5/BLM/BRCA1/BUB1/BUB1B/CCNA2/CCNB1/CCNB2/CCNE1/CCNE2/CDC20/CDC25A/CDC25C/CDC45/CDC6/CDC7/CDCA8/CDK1/CDK2/CENPA/CENPE/CENPF/CENPH/CENPI/CENPK/CENPL/CENPM/CENPN/CENPO/CENPP/CENPQ/CENPU/CHEK1/CLSPN/DBF4/DNA2/DSN1/EXO1/GTSE1/H2AX/INCENP/KIF18A/KIF2C/KNL1/KNTC1/MAD2L1/MCM10/MCM2/MCM3/MCM4/MCM5/MCM6/MCM7/MCM8/MDC1/MIS12/NDC80/NDE1/NSD2/NUF2/NUP107/NUP43/ORC1/ORC5/ORC6/PIAS4/PKMYT1/PLK1/PMF1/PPP1CC/PSMD11/PSMD3/RBBP8/RFC2/RFC3/RFC4/RFC5/RMI1/RMI2/RPA1/RPA3/SGO1/SGO2/SKA1/SKA2/SPC24/SPC25/SPDL1/TOP3A/UBE2C/UBE2N/UBE2S/WEE1/ZNF385A/ZWILCH/ZWINT | 100 | REACTOME |
| REACTOME_CELL_CYCLE_MITOTIC | 1.35319E-65 | 7.49665E-63 | AAAS/ABL1/AKT1/AKT2/ANAPC7/AURKA/AURKB/BIRC5/BORA/BUB1/BUB1B/CCNA2/CCNB1/CCNB2/CCND1/CCND2/CCNE1/CCNE2/CDC20/CDC25A/CDC25B/CDC25C/CDC45/CDC6/CDC7/CDCA5/CDCA8/CDK1/CDK2/CDK4/CDK5RAP2/CDK6/CDKN2C/CDT1/CENPA/CENPE/CENPF/CENPH/CENPI/CENPJ/CENPK/CENPL/CENPM/CENPN/CENPO/CENPP/CENPQ/CENPU/CEP152/CEP41/CEP57/CEP78/CKS1B/CNTRL/CSNK1E/DBF4/DHFR/DNA2/DSN1/E2F1/E2F2/E2F3/E2F5/E2F6/ESCO2/ESPL1/FBXO5/FEN1/FOXM1/GINS1/GINS2/GINS3/GINS4/GMNN/GTSE1/H2AX/H2AZ1/H2AZ2/HAUS1/HAUS5/HAUS6/HAUS8/HMMR/INCENP/KIF18A/KIF20A/KIF23/KIF2C/KNL1/KNTC1/LBR/LIN9/LMNB1/LPIN3/MAD2L1/MCM10/MCM2/MCM3/MCM4/MCM5/MCM6/MCM7/MCM8/MIS12/MYBL2/NCAPD2/NCAPD3/NCAPG/NCAPG2/NCAPH/NCAPH2/NDC1/NDC80/NDE1/NEK2/NUF2/NUP107/NUP188/NUP210/NUP42/NUP43/NUP88/ODF2/OPTN/ORC1/ORC5/ORC6/PCNA/PHLDA1/PKMYT1/PLK1/PLK4/PMF1/POLA1/POLA2/POLD3/POLE/POLE2/PPP1CC/PRIM1/PRIM2/PSMD11/PSMD3/PTTG1/RAN/RBL1/RCC1/RFC2/RFC3/RFC4/RFC5/RPA1/RPA3/RRM2/SET/SGO1/SGO2/SKA1/SKA2/SKP2/SMC2/SMC4/SPC24/SPC25/SPDL1/SRC/TK1/TMPO/TOP2A/TPX2/TUBA1A/TUBA1B/TUBB/TUBB2B/TUBB4B/TUBG1/TUBGCP3/TYMS/UBE2C/UBE2I/UBE2S/VRK1/WEE1/ZWILCH/ZWINT | 185 | REACTOME |
| REACTOME_CELLULAR_RESPONSE_TO_STARVATION | 6.34384E-11 | 1.08141E-08 | ATF4/ATP6V0C/ATP6V0E2/ATP6V1B2/ATP6V1C1/ATP6V1D/ATP6V1E1/ATP6V1G2/CEBPG/EIF2AK4/EIF2S2/RPL10/RPL11/RPL12/RPL13A/RPL17/RPL18/RPL18A/RPL22L1/RPL23/RPL27A/RPL28/RPL29/RPL36/RPL36A/RPL39/RPLP0/RPLP1/RPS10/RPS11/RPS14/RPS16/RPS19/RPS2/RPS20/RPS23/RPS27/RPS27L/RPS28/RPS3A/RPS5/RPS7/RPS8/RPS9/RRAGA/SESN1/SESN2/TRIB3/UBA52 | 49 | REACTOME |
| REACTOME_CELLULAR_RESPONSES_TO_STIMULI | 6.1919E-05 | 0.001932026 | AJUBA/ARFGAP1/ATF4/ATP6V0C/ATP6V0E2/ATP6V1B2/ATP6V1C1/ATP6V1D/ATP6V1E1/ATP6V1G2/BACH1/BAG4/BLVRA/CALR/CAMK2A/CAMK2B/CAMK2G/CBX6/CCNA1/CDKN2D/CEBPG/CREB3L2/CRTC1/DCTN1/DNAJA4/DNAJC3/DYNC1I1/DYNLL2/EIF2AK4/EIF2S2/ERF/ERN1/FBXL17/GPX7/H2AC20/H2AC8/H2BC11/H2BC15/H3-3B/H3C10/HIF1A/HIF3A/HIGD1A/HM13/HMGA1/HSP90B1/HSPA12A/HSPA5/HYOU1/LMNA/MAP2K4/MAPK1/MAPK10/MDM2/MDM4/MEF2C/MYDGF/NDUFA4/NFE2L2/NR3C2/NUP205/NUP62/P4HB/PDIA6/PHC2/PPARGC1A/PSMA2/PSMA3/PSMA6/PSMC2/PSME1/PSME2/PSME4/RELA/RPL10/RPL11/RPL12/RPL13A/RPL17/RPL18/RPL18A/RPL22L1/RPL23/RPL27A/RPL28/RPL29/RPL36/RPL36A/RPL39/RPLP0/RPLP1/RPS10/RPS11/RPS14/RPS16/RPS19/RPS2/RPS20/RPS23/RPS27/RPS27L/RPS28/RPS3A/RPS5/RPS7/RPS8/RPS9/RRAGA/SEM1/SERPINH1/SESN1/SESN2/SHC1/SNCB/SOD1/STAP2/SYVN1/TINF2/TP53/TRIB3/TSPYL2/TUBA4A/TUBA8/TUBB2A/TUBB6/UBA52/USP46/WFS1 | 128 | REACTOME |
| REACTOME_CGMP_EFFECTS | 0.001368297 | 0.022867547 | IRAG1/ITPR1/KCNMA1/PDE10A/PDE1A/PDE1B/PDE2A | 7 | REACTOME |
| REACTOME_CHK1_CHK2_CDS1_MEDIATED_INACTIVATION_OF_CYCLIN_B_CDK1_COMPLEX | 0.002991489 | 0.029203263 | CCNB1/CDC25C/CDK1/CHEK1/WEE1 | 5 | REACTOME |
| REACTOME_CHONDROITIN_SULFATE_DERMATAN_SULFATE_METABOLISM | 0.000694221 | 0.00780911 | AGRN/B3GALT6/CHST11/CHST12/CHST3/CHST9/CSPG4/DSEL/GPC1/GPC2/SDC3/UST | 12 | REACTOME |
| REACTOME_CHROMATIN_MODIFYING_ENZYMES | 1.7139E-05 | 0.000273238 | BRPF1/CCND1/CDK4/DNMT3A/DOT1L/EHMT1/ELP4/EZH2/H2AC11/H2AX/H2AZ1/H2AZ2/HAT1/HDAC2/ING3/ING4/JADE2/JADE3/KANSL2/KDM1A/KDM4B/KDM5B/MBD3/NSD2/PHF2/PRMT1/PRMT5/PRMT6/RUVBL1/RUVBL2/SETD1A/SETDB1/SMARCA4/SMARCB1/SMARCC1/SMARCD1/SUPT20H/SUV39H1/SUV39H2/TRRAP/VPS72/WDR5/WDR77/YEATS4 | 44 | REACTOME |
| REACTOME_CHROMOSOME_MAINTENANCE | 3.83243E-16 | 2.57354E-14 | BLM/CCNA2/CDK2/CENPA/CENPH/CENPI/CENPK/CENPL/CENPM/CENPN/CENPO/CENPP/CENPQ/CENPU/CENPW/CENPX/CHTF18/DKC1/DNA2/DSCC1/FEN1/H2AX/H2AZ1/H2AZ2/HJURP/KNL1/MIS18A/OIP5/PCNA/PIF1/POLA1/POLA2/POLD3/POLR2F/PRIM1/PRIM2/RFC2/RFC3/RFC4/RFC5/RPA1/RPA3/RUVBL1/RUVBL2/WRAP53 | 45 | REACTOME |
| REACTOME_CILIUM_ASSEMBLY | 1.16234E-05 | 0.000189393 | CCT2/CCT3/CCT5/CDK1/CDK5RAP2/CENPJ/CEP152/CEP41/CEP57/CEP78/CEP83/CEP89/CNTRL/CSNK1E/EXOC4/HAUS1/HAUS5/HAUS6/HAUS8/IFT81/MKS1/NDE1/NEK2/ODF2/PLK1/PLK4/RAB3IP/SCLT1/TCTN3/TTC30B/TUBA1A/TUBA1B/TUBB/TUBB2B/TUBB4B/TUBG1 | 36 | REACTOME |
| REACTOME_CLEC7A_DECTIN_1_INDUCES_NFAT_ACTIVATION | 6.74886E-05 | 0.00201509 | AHCYL1/CALM1/ITPR1/NFATC1/PPP3CA/PPP3CB/PPP3R1 | 7 | REACTOME |
| REACTOME_CONDENSATION_OF_PROMETAPHASE_CHROMOSOMES | 3.14235E-07 | 6.69562E-06 | CCNB1/CCNB2/CDK1/NCAPD2/NCAPG/NCAPH/SMC2/SMC4 | 8 | REACTOME |
| REACTOME_COOPERATION_OF_PREFOLDIN_AND_TRIC_CCT_IN_ACTIN_AND_TUBULIN_FOLDING | 0.00498372 | 0.044561616 | CCT2/CCT3/CCT5/TUBA1A/TUBA1B/TUBB2B/TUBB4B/VBP1 | 8 | REACTOME |
| REACTOME_COPI_DEPENDENT_GOLGI_TO_ER_RETROGRADE_TRAFFIC | 0.00116096 | 0.021053729 | ARF4/ARFGAP1/KDELR2/KIF1A/KIF26A/KIF3A/KIF3B/KIF3C/KIF5A/KIF9/KIFAP3/KIFC2/KLC1/NAPB/NSF/SURF4/TMED2/TMED3/TMED9/TUBA4A/TUBA8/TUBB2A/TUBB6 | 23 | REACTOME |
| REACTOME_COPI_MEDIATED_ANTEROGRADE_TRANSPORT | 9.92758E-05 | 0.002852226 | ANK1/ANK2/ANK3/ARF4/ARFGAP1/COG1/COG7/DCTN1/DYNC1I1/DYNLL2/KDELR2/NAPB/NSF/SPTB/SPTBN1/SPTBN2/SPTBN4/TMED2/TMED3/TMED9/TMEM115/TUBA4A/TUBA8/TUBB2A/TUBB6/YKT6 | 26 | REACTOME |
| REACTOME_CREB1_PHOSPHORYLATION_THROUGH_NMDA_RECEPTOR_MEDIATED_ACTIVATION_OF_RAS_SIGNALING | 3.46398E-05 | 0.001247522 | CALM1/CAMK2A/CAMK2B/CAMK2G/DLG2/DLG3/DLG4/GRIN1/LRRC7/MAPK1/NEFL/RASGRF2 | 12 | REACTOME |
| REACTOME_CREB1_PHOSPHORYLATION_THROUGH_THE_ACTIVATION_OF_CAMKII_CAMKK_CAMKIV_CASCASDE | 6.07E-05 | 0.001932026 | CALM1/CAMK2B/CAMK2G/CAMK4/CAMKK1/CAMKK2 | 6 | REACTOME |
| REACTOME_CYCLIN_A_B1_B2_ASSOCIATED_EVENTS_DURING_G2_M_TRANSITION | 2.12965E-07 | 4.72545E-06 | CCNA2/CCNB1/CCNB2/CDC25A/CDC25B/CDC25C/CDK1/CDK2/FOXM1/PKMYT1/PLK1/WEE1 | 12 | REACTOME |
| REACTOME_CYCLIN_A_CDK2_ASSOCIATED_EVENTS_AT_S_PHASE_ENTRY | 0.000199076 | 0.002582783 | AKT1/AKT2/CCNA2/CCND1/CCNE1/CCNE2/CDC25A/CDC25B/CDK2/CDK4/CKS1B/E2F1/E2F5/LIN9/PSMD11/PSMD3/SKP2/WEE1 | 18 | REACTOME |
| REACTOME_CYCLIN_D_ASSOCIATED_EVENTS_IN_G1 | 1.08824E-07 | 2.6795E-06 | ABL1/CCND1/CCND2/CCNE1/CCNE2/CDK2/CDK4/CDK6/CDKN2C/CKS1B/E2F1/E2F2/E2F3/E2F5/RBL1/SKP2/SRC | 17 | REACTOME |
| REACTOME_DAG_AND_IP3_SIGNALING | 3.93267E-08 | 2.9576E-06 | ADCY1/ADCY2/ADCY5/AHCYL1/CALM1/CAMK2A/CAMK2B/CAMK2G/CAMK4/CAMKK1/CAMKK2/ITPR1/NBEA/PDE1A/PDE1B/PRKACB/PRKAR1B/PRKAR2B/PRKCE | 19 | REACTOME |
| REACTOME_DARPP_32_EVENTS | 0.001082521 | 0.020058011 | CALM1/PDE4A/PPP1R1B/PPP3CA/PPP3CB/PPP3R1/PRKACB/PRKAR1B/PRKAR2B | 9 | REACTOME |
| REACTOME_DCC_MEDIATED_ATTRACTIVE_SIGNALING | 0.004335094 | 0.040363734 | ABLIM3/FYN/NTN1/SRC/TRIO | 5 | REACTOME |
| REACTOME_DEACTIVATION_OF_THE_BETA_CATENIN_TRANSACTIVATING_COMPLEX | 7.38623E-07 | 1.48799E-05 | AKT1/AKT2/CBY1/CHD8/MEN1/PYGO1/SOX13/SOX2/SOX3/SOX4/SOX6/SOX7/SOX9/TCF7L1/TLE2 | 15 | REACTOME |
| REACTOME_DEATH_RECEPTOR_SIGNALLING | 7.82254E-05 | 0.002272982 | AKAP13/ARHGEF1/ARHGEF19/ARHGEF4/ARHGEF9/BAG4/BIRC2/CLIP3/HDAC1/IRAK1/ITGB3BP/KALRN/LINGO1/MADD/MCF2/MCF2L/MYD88/NGEF/NSMAF/OMG/PLEKHG2/PLEKHG5/RACK1/RASGRF2/RBCK1/RELA/RTN4/RTN4R/SMPD3/SPPL2A/TRAF2/UBA52/USP2 | 33 | REACTOME |
| REACTOME_DEPOSITION_OF_NEW_CENPA_CONTAINING_NUCLEOSOMES_AT_THE_CENTROMERE | 1.94042E-07 | 4.47913E-06 | CENPA/CENPH/CENPI/CENPK/CENPL/CENPM/CENPN/CENPO/CENPP/CENPQ/CENPU/CENPW/CENPX/H2AX/H2AZ1/H2AZ2/HJURP/KNL1/MIS18A/OIP5/RUVBL1 | 21 | REACTOME |
| REACTOME_DISEASES_OF_BASE_EXCISION_REPAIR | 0.000229979 | 0.002912197 | MUTYH/NEIL3/NTHL1/OGG1 | 4 | REACTOME |
| REACTOME_DISEASES_OF_DNA_REPAIR | 1.13211E-13 | 6.07898E-12 | BARD1/BLM/BRCA1/BRCA2/DNA2/EXO1/MSH2/MSH6/MUTYH/NEIL3/NTHL1/OGG1/RAD51/RAD51AP1/RAD51D/RBBP8/RMI1/RMI2/TOP3A/XRCC2 | 20 | REACTOME |
| REACTOME_DISEASES_OF_GLYCOSYLATION | 0.003927033 | 0.037348953 | ADAMTS6/ADAMTS7/AGRN/ALG14/ALG6/B3GALT6/CHST3/CSPG4/DAG1/DOLK/GALE/GPC1/GPC2/MPDU1/NOTCH1/NOTCH4/POMGNT1/POMT2/SDC3/SEMA5A/SEMA5B/SSPOP | 22 | REACTOME |
| REACTOME_DISEASES_OF_MITOTIC_CELL_CYCLE | 1.1196E-06 | 2.17635E-05 | ANAPC7/CCND1/CCND2/CCNE1/CCNE2/CDK2/CDK4/CDK6/E2F1/E2F2/E2F3/SKP2/UBE2C/UBE2S | 14 | REACTOME |
| REACTOME_DISPLACEMENT_OF_DNA_GLYCOSYLASE_BY_APEX1 | 0.000389507 | 0.004665667 | APEX1/MUTYH/NTHL1/OGG1/UNG | 5 | REACTOME |
| REACTOME_DNA_DAMAGE_BYPASS | 1.55425E-07 | 3.7437E-06 | CUL4A/DTL/MAD2L2/PCLAF/PCNA/POLD3/POLE/POLE2/RAD18/REV3L/RFC2/RFC3/RFC4/RFC5/RPA1/RPA3/USP1 | 17 | REACTOME |
| REACTOME_DNA_DOUBLE_STRAND_BREAK_REPAIR | 1.80016E-17 | 1.53429E-15 | ABL1/ATRIP/BARD1/BLM/BRCA1/BRCA2/CCNA2/CDK2/CHEK1/CLSPN/DCLRE1C/DNA2/EME1/EXO1/FEN1/H2AX/KDM4B/KPNA2/LIG3/MDC1/MUS81/NSD2/PARP1/PAXIP1/PCNA/PIAS4/POLD3/POLE/POLE2/PRKDC/RAD51/RAD51AP1/RAD51D/RBBP8/RFC2/RFC3/RFC4/RFC5/RMI1/RMI2/RPA1/RPA3/SIRT6/SUMO2/TDP1/TIMELESS/TIPIN/TOP3A/UBE2I/UBE2N/XRCC1/XRCC2 | 52 | REACTOME |
| REACTOME_DNA_REPAIR | 4.8532E-21 | 4.88849E-19 | ABL1/ALKBH2/APEX1/ATRIP/BARD1/BLM/BRCA1/BRCA2/CCNA2/CDK2/CENPX/CHEK1/CLSPN/CUL4A/DCLRE1C/DNA2/DTL/EME1/ERCC8/EXO1/FAAP100/FAAP24/FANCA/FANCC/FANCD2/FANCE/FANCG/FANCI/FEN1/GTF2H4/H2AX/H2AZ1/H2AZ2/KDM4B/KPNA2/LIG3/MAD2L2/MDC1/MSH2/MSH6/MUS81/MUTYH/NEIL3/NSD2/NTHL1/OGG1/PARP1/PAXIP1/PCLAF/PCNA/PIAS4/POLD3/POLE/POLE2/POLR2F/PRKDC/RAD18/RAD51/RAD51AP1/RAD51D/RBBP8/REV3L/RFC2/RFC3/RFC4/RFC5/RMI1/RMI2/RPA1/RPA3/RUVBL1/SIRT6/SUMO2/TDP1/TIMELESS/TIPIN/TOP3A/UBE2I/UBE2N/UBE2T/UNG/USP1/XRCC1/XRCC2 | 84 | REACTOME |
| REACTOME_DNA_REPLICATION | 1.3135E-15 | 8.56096E-14 | ANAPC7/CCNA2/CCNE1/CCNE2/CDC45/CDC6/CDC7/CDK2/CDT1/DBF4/DNA2/E2F1/E2F2/E2F3/FEN1/GINS1/GINS2/GINS3/GINS4/GMNN/H2AX/H2AZ1/H2AZ2/MCM10/MCM2/MCM3/MCM4/MCM5/MCM6/MCM7/MCM8/ORC1/ORC5/ORC6/PCNA/POLA1/POLA2/POLD3/POLE/POLE2/PRIM1/PRIM2/PSMD11/PSMD3/RFC2/RFC3/RFC4/RFC5/RPA1/RPA3/SKP2/UBE2C/UBE2S | 53 | REACTOME |
| REACTOME_DNA_REPLICATION_INITIATION | 8.31378E-06 | 0.000138521 | POLA1/POLA2/POLE/POLE2/PRIM1/PRIM2 | 6 | REACTOME |
| REACTOME_DNA_REPLICATION_PRE_INITIATION | 3.15734E-08 | 8.87307E-07 | CDC45/CDC6/CDC7/CDK2/CDT1/DBF4/E2F1/E2F2/E2F3/GMNN/H2AX/H2AZ1/H2AZ2/MCM10/MCM2/MCM3/MCM4/MCM5/MCM6/MCM7/MCM8/ORC1/ORC5/ORC6/POLA1/POLA2/POLE/POLE2/PRIM1/PRIM2/PSMD11/PSMD3/RPA1/RPA3 | 34 | REACTOME |
| REACTOME_DNA_STRAND_ELONGATION | 4.41343E-23 | 7.5232E-21 | CDC45/DNA2/FEN1/GINS1/GINS2/GINS3/GINS4/MCM2/MCM3/MCM4/MCM5/MCM6/MCM7/MCM8/PCNA/POLA1/POLA2/POLD3/PRIM1/PRIM2/RFC2/RFC3/RFC4/RFC5/RPA1/RPA3 | 26 | REACTOME |
| REACTOME_DOPAMINE_NEUROTRANSMITTER_RELEASE_CYCLE | 7.768E-12 | 1.80571E-09 | APBA1/CPLX1/LIN7B/PPFIA2/PPFIA3/PPFIA4/RAB3A/RIMS1/SNAP25/STX1A/STXBP1/SYN1/SYN2/SYN3/SYT1/TSPOAP1/VAMP2 | 17 | REACTOME |
| REACTOME_DOWNSTREAM_SIGNALING_EVENTS_OF_B_CELL_RECEPTOR_BCR | 0.00093787 | 0.017896511 | BCL10/BTRC/CALM1/NFATC1/NFKBIE/PPP3CA/PPP3CB/PPP3R1/PRKCB/PSMA2/PSMA3/PSMA6/PSMC2/PSME1/PSME2/PSME4/RASGRP1/RELA/SEM1/UBA52 | 20 | REACTOME |
| REACTOME_DUAL_INCISION_IN_GG_NER | 1.80735E-05 | 0.000286078 | CUL4A/GTF2H4/PARP1/PCNA/POLD3/POLE/POLE2/RFC2/RFC3/RFC4/RFC5/RPA1/RPA3 | 13 | REACTOME |
| REACTOME_DUAL_INCISION_IN_TC_NER | 0.000828081 | 0.00908429 | CUL4A/ERCC8/GTF2H4/PCNA/POLD3/POLE/POLE2/POLR2F/RFC2/RFC3/RFC4/RFC5/RPA1/RPA3 | 14 | REACTOME |
| REACTOME_E2F_ENABLED_INHIBITION_OF_PRE_REPLICATION_COMPLEX_FORMATION | 2.31621E-05 | 0.000356439 | CCNB1/CDK1/MCM8/ORC1/ORC5/ORC6 | 6 | REACTOME |
| REACTOME_E2F_MEDIATED_REGULATION_OF_DNA_REPLICATION | 4.09644E-07 | 8.40529E-06 | CCNB1/CDK1/E2F1/MCM8/ORC1/ORC5/ORC6/POLA1/POLA2/PRIM1/PRIM2 | 11 | REACTOME |
| REACTOME_ER_TO_GOLGI_ANTEROGRADE_TRANSPORT | 0.000234869 | 0.005887848 | ANK1/ANK2/ANK3/ARF4/ARFGAP1/CNIH2/CNIH3/COG1/COG7/DCTN1/DYNC1I1/DYNLL2/GRIA1/KDELR2/LMAN2/NAPB/NSF/SEC16A/SEC16B/SEC24D/SPTB/SPTBN1/SPTBN2/SPTBN4/TMED2/TMED3/TMED9/TMEM115/TRAPPC6B/TUBA4A/TUBA8/TUBB2A/TUBB6/YKT6 | 34 | REACTOME |
| REACTOME_EUKARYOTIC_TRANSLATION_ELONGATION | 1.58707E-10 | 2.53634E-08 | EEF1A2/RPL10/RPL11/RPL12/RPL13A/RPL17/RPL18/RPL18A/RPL22L1/RPL23/RPL27A/RPL28/RPL29/RPL36/RPL36A/RPL39/RPLP0/RPLP1/RPS10/RPS11/RPS14/RPS16/RPS19/RPS2/RPS20/RPS23/RPS27/RPS27L/RPS28/RPS3A/RPS5/RPS7/RPS8/RPS9/UBA52 | 35 | REACTOME |
| REACTOME_EUKARYOTIC_TRANSLATION_INITIATION | 1.52268E-09 | 1.94675E-07 | EIF2S2/EIF3B/EIF3M/EIF4A1/EIF4EBP1/RPL10/RPL11/RPL12/RPL13A/RPL17/RPL18/RPL18A/RPL22L1/RPL23/RPL27A/RPL28/RPL29/RPL36/RPL36A/RPL39/RPLP0/RPLP1/RPS10/RPS11/RPS14/RPS16/RPS19/RPS2/RPS20/RPS23/RPS27/RPS27L/RPS28/RPS3A/RPS5/RPS7/RPS8/RPS9/UBA52 | 39 | REACTOME |
| REACTOME_EXPORT_OF_VIRAL_RIBONUCLEOPROTEINS_FROM_NUCLEUS | 0.001210786 | 0.012961845 | AAAS/NDC1/NUP107/NUP188/NUP210/NUP42/NUP43/NUP88/RAN | 9 | REACTOME |
| REACTOME_EXTENSION_OF_TELOMERES | 2.94672E-13 | 1.41955E-11 | BLM/CCNA2/CDK2/CHTF18/DKC1/DNA2/DSCC1/FEN1/PCNA/PIF1/POLA1/POLA2/POLD3/PRIM1/PRIM2/RFC2/RFC3/RFC4/RFC5/RPA1/RPA3/RUVBL1/RUVBL2/WRAP53 | 24 | REACTOME |
| REACTOME_FANCONI_ANEMIA_PATHWAY | 3.20327E-08 | 8.87307E-07 | ATRIP/CENPX/EME1/FAAP100/FAAP24/FANCA/FANCC/FANCD2/FANCE/FANCG/FANCI/MUS81/RPA1/RPA3/UBE2T/USP1 | 16 | REACTOME |
| REACTOME_FORMATION_OF_TUBULIN_FOLDING_INTERMEDIATES_BY_CCT_TRIC | 0.004559354 | 0.041578303 | CCT2/CCT3/CCT5/TUBA1A/TUBA1B/TUBB2B/TUBB4B | 7 | REACTOME |
| REACTOME_G_ALPHA_12_13_SIGNALLING_EVENTS | 0.000792639 | 0.015590607 | ADRA1A/ADRA1B/AKAP13/ARHGEF1/ARHGEF19/ARHGEF4/ARHGEF9/GNB2/GNB5/GNG2/GNG3/GNG5/KALRN/MCF2/MCF2L/NGEF/PLEKHG2/PLEKHG5/RASGRF2/RHOC | 20 | REACTOME |
| REACTOME_G_ALPHA_I_SIGNALLING_EVENTS | 1.75598E-06 | 8.16371E-05 | ACKR3/ADCY1/ADCY2/ADCY5/ADRA2A/ADRA2C/AHCYL1/CALM1/CAMK2A/CAMK2B/CAMK2G/CAMK4/CAMKK1/CAMKK2/CHRM4/CORT/CX3CL1/CXCR5/GABBR1/GABBR2/GNAI1/GNAI2/GNAL/GNAQ/GNAZ/GNB2/GNB5/GNG2/GNG3/GNG5/GPR37L1/GRM2/GRM3/GRM8/ITPR1/LPAR2/MAPK1/NBEA/NPY/NPY1R/OPRL1/P2RY14/PDE1A/PDE1B/PDE4A/PDYN/PENK/PLCB1/PLCB4/PNOC/PPP1R1B/PPP3CA/PPP3CB/PPP3R1/PRKACB/PRKAR1B/PRKAR2B/RGR/RGS20/RGS4/RGS7/RGS8/S1PR2/S1PR3/SST/SSTR2 | 66 | REACTOME |
| REACTOME_G_ALPHA_Z_SIGNALLING_EVENTS | 1.7886E-05 | 0.0007146 | ADCY1/ADCY2/ADCY5/ADRA2A/ADRA2C/GNAI1/GNAI2/GNAZ/GNB2/GNB5/GNG2/GNG3/GNG5/PRKCB/PRKCE/RGS20/RGS4 | 17 | REACTOME |
| REACTOME_G_PROTEIN_MEDIATED_EVENTS | 2.89266E-10 | 4.35091E-08 | ADCY1/ADCY2/ADCY5/AHCYL1/CALM1/CAMK2A/CAMK2B/CAMK2G/CAMK4/CAMKK1/CAMKK2/GNAI1/GNAI2/GNAL/GNAQ/ITPR1/MAPK1/NBEA/PDE1A/PDE1B/PLCB1/PLCB4/PRKACB/PRKAR1B/PRKAR2B | 25 | REACTOME |
| REACTOME_G0_AND_EARLY_G1 | 5.60325E-09 | 1.72456E-07 | CCNA2/CCNE1/CCNE2/CDC25A/CDC6/CDK1/CDK2/E2F1/E2F5/LIN9/MYBL2/PCNA/RBL1/TOP2A | 14 | REACTOME |
| REACTOME_G1_S_SPECIFIC_TRANSCRIPTION | 2.73891E-14 | 1.59722E-12 | CCNE1/CDC25A/CDC45/CDC6/CDK1/CDT1/DHFR/E2F1/E2F5/E2F6/FBXO5/LIN9/ORC1/PCNA/POLA1/RBL1/RRM2/TK1/TYMS | 19 | REACTOME |
| REACTOME_G2_M_CHECKPOINTS | 2.86745E-15 | 1.81551E-13 | ATRIP/BARD1/BLM/BRCA1/CCNB1/CCNB2/CDC25A/CDC25C/CDC45/CDC6/CDC7/CDK1/CDK2/CHEK1/CLSPN/DBF4/DNA2/EXO1/GTSE1/H2AX/MCM10/MCM2/MCM3/MCM4/MCM5/MCM6/MCM7/MCM8/MDC1/NSD2/ORC1/ORC5/ORC6/PIAS4/PKMYT1/PSMD11/PSMD3/RBBP8/RFC2/RFC3/RFC4/RFC5/RMI1/RMI2/RPA1/RPA3/TOP3A/UBE2N/WEE1 | 49 | REACTOME |
| REACTOME_G2_M_DNA_DAMAGE_CHECKPOINT | 4.11213E-08 | 1.08482E-06 | ATRIP/BARD1/BLM/BRCA1/CCNB1/CDC25C/CDK1/CHEK1/DNA2/EXO1/H2AX/MDC1/NSD2/PIAS4/RBBP8/RFC2/RFC3/RFC4/RFC5/RMI1/RMI2/RPA1/RPA3/TOP3A/UBE2N/WEE1 | 26 | REACTOME |
| REACTOME_G2_M_DNA_REPLICATION_CHECKPOINT | 4.12203E-06 | 7.30754E-05 | CCNB1/CCNB2/CDK1/PKMYT1/WEE1 | 5 | REACTOME |
| REACTOME_G2_PHASE | 0.000229979 | 0.002912197 | CCNA2/CDK2/E2F1/E2F3 | 4 | REACTOME |
| REACTOME_GABA_B_RECEPTOR_ACTIVATION | 6.77739E-05 | 0.00201509 | ADCY1/ADCY2/ADCY5/GABBR1/GABBR2/GNAI1/GNAI2/GNAL/GNB2/GNB5/GNG2/GNG3/GNG5/KCNJ4/KCNJ9 | 15 | REACTOME |
| REACTOME_GABA_RECEPTOR_ACTIVATION | 2.34165E-08 | 1.99587E-06 | ADCY1/ADCY2/ADCY5/ARHGEF9/GABBR1/GABBR2/GABRA2/GABRA3/GABRA5/GABRB1/GABRB2/GABRB3/GABRG2/GNAI1/GNAI2/GNAL/GNB2/GNB5/GNG2/GNG3/GNG5/KCNJ4/KCNJ9/NPTN | 24 | REACTOME |
| REACTOME_GABA_SYNTHESIS_RELEASE_REUPTAKE_AND_DEGRADATION | 1.59472E-07 | 9.712E-06 | CPLX1/GAD2/RAB3A/RIMS1/SLC6A1/SLC6A11/SLC6A13/SNAP25/STX1A/STXBP1/SYT1/VAMP2 | 12 | REACTOME |
| REACTOME_GAP_FILLING_DNA_REPAIR_SYNTHESIS_AND_LIGATION_IN_GG_NER | 2.12965E-07 | 4.72545E-06 | LIG3/PCNA/POLD3/POLE/POLE2/RFC2/RFC3/RFC4/RFC5/RPA1/RPA3/XRCC1 | 12 | REACTOME |
| REACTOME_GLOBAL_GENOME_NUCLEOTIDE_EXCISION_REPAIR_GG_NER | 5.2072E-05 | 0.000754194 | CUL4A/GTF2H4/LIG3/PARP1/PCNA/POLD3/POLE/POLE2/RFC2/RFC3/RFC4/RFC5/RPA1/RPA3/RUVBL1/SUMO2/UBE2I/UBE2N/XRCC1 | 19 | REACTOME |
| REACTOME_GLUCAGON_LIKE_PEPTIDE_1_GLP1_REGULATES_INSULIN_SECRETION | 4.94875E-05 | 0.001704557 | ADCY5/AKAP5/GNB2/GNB5/GNG2/GNG3/GNG5/ITPR1/KCNB1/KCNC2/KCNS3/PRKACB/PRKAR1B/PRKAR2B/RAPGEF4 | 15 | REACTOME |
| REACTOME_GLUCAGON_SIGNALING_IN_METABOLIC_REGULATION | 0.003648492 | 0.046880369 | ADCY1/ADCY2/ADCY5/GNB2/GNG2/GNG3/GNG5/PRKACB/PRKAR1B/PRKAR2B | 10 | REACTOME |
| REACTOME_GLUTAMATE_NEUROTRANSMITTER_RELEASE_CYCLE | 2.3698E-11 | 4.66122E-09 | CPLX1/GLS2/PPFIA2/PPFIA3/PPFIA4/RAB3A/RIMS1/SLC17A7/SLC1A1/SLC1A2/SLC1A3/SNAP25/STX1A/STXBP1/SYT1/TSPOAP1/VAMP2 | 17 | REACTOME |
| REACTOME_GLYCOSAMINOGLYCAN_METABOLISM | 0.001109653 | 0.020267014 | B3GAT1/B4GALT4/B4GALT6/B4GALT7/B4GAT1/CHPF/CHPF2/CHST1/CHST14/EXT2/GALNS/GLB1L/GPC4/GPC5/GUSB/HAS2/HS3ST2/HS3ST4/HS6ST2/HS6ST3/IDS/NAGLU/PRELP/SDC1/SGSH/SLC35D2/VCAN | 27 | REACTOME |
| REACTOME_GOLGI_TO_ER_RETROGRADE_TRANSPORT | 0.001755652 | 0.027373191 | ARF4/ARFGAP1/DCTN1/DYNC1I1/DYNLL2/GALNT2/KDELR2/KIF1A/KIF26A/KIF3A/KIF3B/KIF3C/KIF5A/KIF9/KIFAP3/KIFC2/KLC1/NAPB/NSF/RAB6B/SURF4/TMED2/TMED3/TMED9/TUBA4A/TUBA8/TUBB2A/TUBB6 | 28 | REACTOME |
| REACTOME_HDR_THROUGH_HOMOLOGOUS_RECOMBINATION_HRR | 1.26825E-13 | 6.38736E-12 | ATRIP/BARD1/BLM/BRCA1/BRCA2/CHEK1/DNA2/EME1/EXO1/MUS81/PCNA/POLD3/POLE/POLE2/RAD51/RAD51AP1/RAD51D/RBBP8/RFC2/RFC3/RFC4/RFC5/RMI1/RMI2/RPA1/RPA3/TOP3A/XRCC2 | 28 | REACTOME |
| REACTOME_HDR_THROUGH_MMEJ_ALT_NHEJ | 0.000725234 | 0.008075971 | FEN1/LIG3/PARP1/RBBP8/XRCC1 | 5 | REACTOME |
| REACTOME_HDR_THROUGH_SINGLE_STRAND_ANNEALING_SSA | 1.40807E-10 | 5.57193E-09 | ABL1/ATRIP/BARD1/BLM/BRCA1/DNA2/EXO1/RAD51/RBBP8/RFC2/RFC3/RFC4/RFC5/RMI1/RMI2/RPA1/RPA3/TOP3A | 18 | REACTOME |
| REACTOME_HEPARAN_SULFATE_HEPARIN_HS_GAG_METABOLISM | 0.000128314 | 0.003383917 | B3GAT1/B4GALT7/EXT2/GLB1L/GPC4/GPC5/GUSB/HS3ST2/HS3ST4/HS6ST2/HS6ST3/IDS/NAGLU/SDC1/SGSH/SLC35D2/VCAN | 17 | REACTOME |
| REACTOME_HIV_LIFE_CYCLE | 0.003999282 | 0.037873546 | AAAS/FEN1/GTF2F1/GTF2F2/GTF2H4/NCBP2/NDC1/NELFA/NELFCD/NUP107/NUP188/NUP210/NUP42/NUP43/NUP88/POLR2F/RAN/RANBP1/RCC1/SSRP1/SUPT16H/TAF15/VPS37B | 23 | REACTOME |
| REACTOME_HOMOLOGOUS_DNA_PAIRING_AND_STRAND_EXCHANGE | 1.73355E-13 | 8.53677E-12 | ATRIP/BARD1/BLM/BRCA1/BRCA2/CHEK1/DNA2/EXO1/RAD51/RAD51AP1/RAD51D/RBBP8/RFC2/RFC3/RFC4/RFC5/RMI1/RMI2/RPA1/RPA3/TOP3A/XRCC2 | 22 | REACTOME |
| REACTOME_HOMOLOGY_DIRECTED_REPAIR | 6.81778E-17 | 4.87361E-15 | ABL1/ATRIP/BARD1/BLM/BRCA1/BRCA2/CCNA2/CDK2/CHEK1/CLSPN/DNA2/EME1/EXO1/FEN1/H2AX/LIG3/MDC1/MUS81/NSD2/PARP1/PCNA/PIAS4/POLD3/POLE/POLE2/RAD51/RAD51AP1/RAD51D/RBBP8/RFC2/RFC3/RFC4/RFC5/RMI1/RMI2/RPA1/RPA3/SIRT6/SUMO2/TIMELESS/TIPIN/TOP3A/UBE2I/UBE2N/XRCC1/XRCC2 | 46 | REACTOME |
| REACTOME_HS_GAG_DEGRADATION | 0.002571059 | 0.035156134 | GLB1L/GPC4/GPC5/GUSB/IDS/NAGLU/SDC1/SGSH | 8 | REACTOME |
| REACTOME_INFECTIOUS_DISEASE | 1.03452E-05 | 0.000440877 | ACTB/ADCY1/ADCY2/ADCY5/ADRB1/AHCYL1/ANTXR2/AP1S1/ARPC1A/ARPC2/ARPC4/ARPC5/ATP1A2/ATP1A3/ATP1B1/ATP1B3/BRMS1/BTRC/CALM1/CALR/CD9/CHD3/CRHR1/CYFIP2/DAD1/DAXX/DDOST/DNAJC3/DYNC1I1/DYNLL2/EDEM2/ELK1/ELMO1/EPS15/FXYD1/FXYD7/GANAB/GNAI1/GNAI2/GNAZ/GNB2/GNB5/GNG2/GNG3/GNG5/GPR176/GPR27/GTF2E2/H2AC20/H2AC8/H2BC11/H2BC15/H3C10/HDAC1/HLA-A/HMG20B/HMGA1/HRH2/IFNAR2/IMPDH1/ITPR1/MAN1B1/MAP2K1/MAP2K4/MAPK1/MGAT4B/MGAT4C/MOGS/MYO5A/MYO9B/NFE2L2/NMT2/NT5E/NUP205/NUP62/P2RY11/POLR2H/POLR2J/PRKACB/PRKAR1B/PRKAR2B/PSMA2/PSMA3/PSMA6/PSMC2/PSME1/PSME2/PSME4/RANGAP1/RELA/REST/RPL10/RPL11/RPL12/RPL13A/RPL17/RPL18/RPL18A/RPL22L1/RPL23/RPL27A/RPL28/RPL29/RPL36/RPL36A/RPL39/RPLP0/RPLP1/RPN1/RPN2/RPS10/RPS11/RPS14/RPS16/RPS19/RPS2/RPS20/RPS23/RPS27/RPS27L/RPS28/RPS3A/RPS5/RPS7/RPS8/RPS9/S1PR1/SAP30/SEM1/SH3GL2/SLC25A4/SNAP25/SRPK2/STAM/STT3A/STX1A/STX1B/SV2A/SYT1/TUBA4A/TUBA8/TUBB2A/TUBB6/TUSC3/TYK2/UBA52/VAMP1/VAMP2/VIPR1/WASF1/WASF3/WIPF1/WIPF3 | 153 | REACTOME |
| REACTOME_INFLUENZA_INFECTION | 1.54627E-06 | 7.46E-05 | CALR/DNAJC3/NUP205/NUP62/POLR2H/POLR2J/RPL10/RPL11/RPL12/RPL13A/RPL17/RPL18/RPL18A/RPL22L1/RPL23/RPL27A/RPL28/RPL29/RPL36/RPL36A/RPL39/RPLP0/RPLP1/RPS10/RPS11/RPS14/RPS16/RPS19/RPS2/RPS20/RPS23/RPS27/RPS27L/RPS28/RPS3A/RPS5/RPS7/RPS8/RPS9/UBA52 | 40 | REACTOME |
| REACTOME_INHIBITION_OF_REPLICATION_INITIATION_OF_DAMAGED_DNA_BY_RB1_E2F1 | 0.002991489 | 0.029203263 | E2F1/POLA1/POLA2/PRIM1/PRIM2 | 5 | REACTOME |
| REACTOME_INITIATION_OF_NUCLEAR_ENVELOPE_NE_REFORMATION | 0.000587267 | 0.006708158 | CCNB1/CCNB2/CDK1/LBR/LMNB1/TMPO/VRK1 | 7 | REACTOME |
| REACTOME_INSULIN_PROCESSING | 0.00279596 | 0.037826829 | CPE/KIF5A/KIF5C/MYO5A/MYRIP/PCSK2/SLC30A5/STX1A/VAMP2 | 9 | REACTOME |
| REACTOME_INTEGRATION_OF_ENERGY_METABOLISM | 4.26834E-11 | 7.79582E-09 | ABCC8/ADCY1/ADCY2/ADCY5/ADRA2A/ADRA2C/AHCYL1/AKAP5/CACNA1A/CACNA1C/CACNA2D2/CACNB2/CACNB3/CHRM3/GNAI1/GNAI2/GNAQ/GNB2/GNB5/GNG2/GNG3/GNG5/ITPR1/KCNB1/KCNC2/KCNJ11/KCNS3/MLX/PLCB1/PRKACB/PRKAG2/PRKAR1B/PRKAR2B/RAPGEF4/SNAP25/STX1A/STXBP1/SYT5/VAMP2 | 39 | REACTOME |
| REACTOME_INTERACTION_BETWEEN_L1_AND_ANKYRINS | 5.33899E-07 | 2.90464E-05 | ACTB/ANK1/ANK2/ANK3/KCNQ3/L1CAM/SCN2A/SCN2B/SCN3B/SCN4B/SCN8A/SPTB/SPTBN1/SPTBN2/SPTBN4 | 15 | REACTOME |
| REACTOME_INTERACTIONS_OF_REV_WITH_HOST_CELLULAR_PROTEINS | 0.000153098 | 0.002068689 | AAAS/NDC1/NUP107/NUP188/NUP210/NUP42/NUP43/NUP88/RAN/RANBP1/RCC1 | 11 | REACTOME |
| REACTOME_INTERCONVERSION_OF_NUCLEOTIDE_DI_AND_TRIPHOSPHATES | 0.002079861 | 0.021142073 | CTPS1/CTPS2/DCTPP1/DTYMK/NME1/RRM1/RRM2/TYMS | 8 | REACTOME |
| REACTOME_INTRACELLULAR_SIGNALING_BY_SECOND_MESSENGERS | 0.00166019 | 0.026531914 | ADCY1/ADCY2/ADCY5/AHCYL1/CALM1/CAMK2A/CAMK2B/CAMK2G/CAMK4/CAMKK1/CAMKK2/CBX6/CHD3/ERBB4/FGF17/FGF22/FGF9/FGFR3/FLT3LG/HDAC1/HDAC7/IRAK1/IRAK4/ITPR1/KIT/MAPK1/MDM2/MYD88/NBEA/NRG3/PDE1A/PDE1B/PHC2/PIK3CB/PIK3R1/PIP5K1B/PIP5K1C/PRKACB/PRKAR1B/PRKAR2B/PRKCE/PSMA2/PSMA3/PSMA6/PSMC2/PSME1/PSME2/PSME4/REST/RRAGA/SEM1/TP53/TRIB3/UBA52 | 54 | REACTOME |
| REACTOME_INWARDLY_RECTIFYING_K_CHANNELS | 0.001682018 | 0.026713788 | ABCC8/GABBR1/GABBR2/GNB2/GNB5/GNG2/GNG3/GNG5/KCNJ11/KCNJ4/KCNJ9 | 11 | REACTOME |
| REACTOME_ION_CHANNEL_TRANSPORT | 0.000339205 | 0.00803099 | ANO5/ASIC2/ATP13A2/ATP1A2/ATP1A3/ATP1B1/ATP1B3/ATP2B1/ATP2B2/ATP6V0A1/ATP6V0C/ATP6V0E2/ATP6V1B2/ATP6V1C1/ATP6V1D/ATP6V1E1/ATP6V1G2/ATP7B/ATP9A/CALM1/CAMK2A/CAMK2B/CAMK2G/CLCN4/FKBP1B/FXYD1/FXYD7/MCOLN1/NALCN/RYR1/SLC9B2/TRPM3/TSC22D3/UBA52/UNC79/UNC80/WNK2/WWP1 | 38 | REACTOME |
| REACTOME_ION_HOMEOSTASIS | 1.47354E-06 | 7.24584E-05 | AHCYL1/ATP1A2/ATP1A3/ATP1B1/ATP1B3/ATP2B1/ATP2B2/CALM1/CAMK2A/CAMK2B/CAMK2G/FKBP1B/FXYD1/FXYD7/ITPR1/KCNJ11/RYR1/SLC8A2/SLC8A3/STIM1 | 20 | REACTOME |
| REACTOME_ION_TRANSPORT_BY_P_TYPE_ATPASES | 0.001354995 | 0.022867547 | ATP13A2/ATP1A2/ATP1A3/ATP1B1/ATP1B3/ATP2B1/ATP2B2/ATP7B/ATP9A/CALM1/CAMK2A/CAMK2B/CAMK2G/FXYD1/FXYD7 | 15 | REACTOME |
| REACTOME_KINESINS | 6.89228E-06 | 0.000118398 | CENPE/KIF11/KIF15/KIF18A/KIF18B/KIF20A/KIF22/KIF23/KIF26B/KIF2C/KIF4A/KIFC1/RACGAP1/TUBA1A/TUBA1B/TUBB2B/TUBB4B | 17 | REACTOME |
| REACTOME_L1CAM_INTERACTIONS | 7.25417E-06 | 0.000314388 | ACTB/ANK1/ANK2/ANK3/CNTNAP1/DLG3/DLG4/DNM1/DNM3/DPYSL2/KCNQ3/L1CAM/LAMC1/MAP2K1/MAPK1/MSN/PAK1/RPS6KA5/SCN2A/SCN2B/SCN3B/SCN4B/SCN8A/SH3GL2/SPTB/SPTBN1/SPTBN2/SPTBN4/TUBA4A/TUBA8/TUBB2A/TUBB6 | 32 | REACTOME |
| REACTOME_LAGGING_STRAND_SYNTHESIS | 1.89999E-11 | 8.25564E-10 | DNA2/FEN1/PCNA/POLA1/POLA2/POLD3/PRIM1/PRIM2/RFC2/RFC3/RFC4/RFC5/RPA1/RPA3 | 14 | REACTOME |
| REACTOME_LGI_ADAM_INTERACTIONS | 0.000508385 | 0.010923873 | ADAM11/CACNG8/DLG4/LGI1/LGI4/STX1A/STX1B | 7 | REACTOME |
| REACTOME_LONG_TERM_POTENTIATION | 2.19607E-09 | 2.67397E-07 | CALM1/CAMK2A/CAMK2B/CAMK2G/DLG2/DLG3/DLG4/ERBB4/GRIA1/GRIN1/GRIN2A/GRIN2C/LRRC7/NEFL/NRGN | 15 | REACTOME |
| REACTOME_LYSOSPHINGOLIPID_AND_LPA_RECEPTORS | 0.000508385 | 0.010923873 | LPAR2/PLPPR2/PLPPR3/PLPPR4/S1PR1/S1PR2/S1PR3 | 7 | REACTOME |
| REACTOME_M_PHASE | 1.77628E-26 | 4.37358E-24 | AAAS/ANAPC7/AURKB/BIRC5/BUB1/BUB1B/CCNB1/CCNB2/CDC20/CDCA5/CDCA8/CDK1/CDK5RAP2/CENPA/CENPE/CENPF/CENPH/CENPI/CENPJ/CENPK/CENPL/CENPM/CENPN/CENPO/CENPP/CENPQ/CENPU/CEP152/CEP41/CEP57/CEP78/CNTRL/CSNK1E/DSN1/ESPL1/FBXO5/H2AX/H2AZ1/H2AZ2/HAUS1/HAUS5/HAUS6/HAUS8/INCENP/KIF18A/KIF20A/KIF23/KIF2C/KNL1/KNTC1/LBR/LMNB1/LPIN3/MAD2L1/MIS12/NCAPD2/NCAPD3/NCAPG/NCAPG2/NCAPH/NCAPH2/NDC1/NDC80/NDE1/NEK2/NUF2/NUP107/NUP188/NUP210/NUP42/NUP43/NUP88/ODF2/PLK1/PLK4/PMF1/PPP1CC/PSMD11/PSMD3/PTTG1/RAN/RCC1/SET/SGO1/SGO2/SKA1/SKA2/SMC2/SMC4/SPC24/SPC25/SPDL1/TMPO/TUBA1A/TUBA1B/TUBB/TUBB2B/TUBB4B/TUBG1/TUBGCP3/UBE2C/UBE2I/UBE2S/VRK1/ZWILCH/ZWINT | 106 | REACTOME |
| REACTOME_MAPK_FAMILY_SIGNALING_CASCADES | 0.001557212 | 0.025361722 | ACTB/ARRB1/CALM1/CAMK2A/CAMK2B/CAMK2G/CNKSR2/DLG2/DLG3/DLG4/DUSP4/DUSP6/DUSP8/DUSP9/ERBB4/FGF17/FGF22/FGF9/FGFR3/FLT3LG/FRS3/GFRA2/GRIN1/KALRN/KIT/LRRC7/MAP2K1/MAPK1/MRAS/NEFL/NRG3/PAK1/PAK3/PEBP1/PIK3CB/PIK3R1/PRKACB/PSMA2/PSMA3/PSMA6/PSMC2/PSME1/PSME2/PSME4/RAPGEF2/RASAL1/RASGEF1A/RASGRF2/RASGRP1/SEM1/SHC1/SPTB/SPTBN1/SPTBN2/SPTBN4/TYK2/UBA52 | 57 | REACTOME |
| REACTOME_MATURATION_OF_SARS_COV_2_SPIKE_PROTEIN | 5.27724E-05 | 0.001750307 | DAD1/DDOST/EDEM2/GANAB/MAN1B1/MGAT4B/MGAT4C/MOGS/RPN1/RPN2/STT3A/TUSC3 | 12 | REACTOME |
| REACTOME_MEIOSIS | 0.001658024 | 0.017169074 | BLM/BRCA1/BRCA2/CDK2/CDK4/DIDO1/H2AX/H2AZ1/H2AZ2/LMNB1/MND1/PSMC3IP/RAD51/RBBP8/RPA1/RPA3/SYCE2/SYNE2/TOP3A/UBE2I | 20 | REACTOME |
| REACTOME_MEIOTIC_RECOMBINATION | 0.004419832 | 0.040640443 | BLM/BRCA1/BRCA2/CDK2/CDK4/H2AX/H2AZ1/H2AZ2/MND1/PSMC3IP/RAD51/RBBP8/RPA1/RPA3/TOP3A | 15 | REACTOME |
| REACTOME_MEMBRANE_TRAFFICKING | 4.99968E-05 | 0.001704557 | ACTB/AMPH/ANK1/ANK2/ANK3/AP1S1/AP4M1/AP4S1/ARF4/ARFGAP1/ARPC1A/ARPC2/ARPC4/ARPC5/ARRB1/BLOC1S4/CALM1/CCZ1/CCZ1B/CLVS1/CLVS2/CNIH2/CNIH3/COG1/COG7/CYTH2/DCTN1/DNM1/DNM3/DYNC1I1/DYNLL2/EPS15/GABARAPL2/GALNT2/GGA1/GOLGA5/GRIA1/GRK3/KDELR2/KIAA0319/KIF1A/KIF26A/KIF3A/KIF3B/KIF3C/KIF5A/KIF9/KIFAP3/KIFC2/KLC1/LMAN2/MADD/MYO5A/NAPB/NECAP1/NSF/PACSIN1/PIP5K1C/PRKAG2/RAB13/RAB27B/RAB33A/RAB38/RAB3A/RAB6B/RABEP1/REPS2/SBF1/SCOC/SEC16A/SEC16B/SEC24D/SGIP1/SH3GL2/SNAP91/SNX5/SPTB/SPTBN1/SPTBN2/SPTBN4/STAM/STON2/STX4/SURF4/SYS1/SYT1/TBC1D1/TBC1D24/TMED2/TMED3/TMED9/TMEM115/TPD52L1/TRAPPC6B/TRIP10/TUBA4A/TUBA8/TUBB2A/TUBB6/TXNDC5/UBA52/ULK1/VAMP2/VPS53/YKT6/YWHAG/YWHAH | 107 | REACTOME |
| REACTOME_METABOLISM_OF_AMINO_ACIDS_AND_DERIVATIVES | 0.000169535 | 0.004335019 | ACADSB/AHCY/ALDH6A1/BCKDK/CKMT1A/CKMT1B/CRYM/DMGDH/GLS2/GLUD1/GOT1/GOT2/GPT/LIPT1/NAALAD2/NAT8L/OAT/PAH/PPM1K/PRODH/PSMA2/PSMA3/PSMA6/PSMC2/PSME1/PSME2/PSME4/PSTK/RIDA/RPL10/RPL11/RPL12/RPL13A/RPL17/RPL18/RPL18A/RPL22L1/RPL23/RPL27A/RPL28/RPL29/RPL36/RPL36A/RPL39/RPLP0/RPLP1/RPS10/RPS11/RPS14/RPS16/RPS19/RPS2/RPS20/RPS23/RPS27/RPS27L/RPS28/RPS3A/RPS5/RPS7/RPS8/RPS9/SEM1/SERINC1/SERINC2/SLC3A2/SLC6A11/UBA52 | 68 | REACTOME |
| REACTOME_METABOLISM_OF_CARBOHYDRATES | 0.001641015 | 0.026494639 | ALDH1A1/ALDOC/B3GAT1/B4GALT4/B4GALT6/B4GALT7/B4GAT1/CALM1/CHPF/CHPF2/CHST1/CHST14/ENO2/ENO3/EXT2/FUT9/GALK1/GALNS/GLB1L/GLYCTK/GOT1/GOT2/GPC4/GPC5/GUSB/HAS2/HK1/HS3ST2/HS3ST4/HS6ST2/HS6ST3/IDS/NAGLU/NHLRC1/NUP205/NUP62/PC/PFKFB2/PFKM/PGAM1/PGLS/PGP/PHKA2/PRELP/PRKACB/PYGL/PYGM/SDC1/SGSH/SLC35D2/UBA52/VCAN | 52 | REACTOME |
| REACTOME_METABOLISM_OF_NUCLEOTIDES | 0.002732018 | 0.027518874 | CAD/CTPS1/CTPS2/DCTPP1/DHODH/DTYMK/NME1/NT5C3A/NUDT1/NUDT5/PAICS/PFAS/RRM1/RRM2/TK1/TYMS/UMPS | 17 | REACTOME |
| REACTOME_METABOLISM_OF_RNA | 2.96153E-17 | 2.30073E-15 | AAAS/ADAT1/AKT1/ALYREF/ANP32A/BOP1/BYSL/CCAR1/CD2BP2/CDKAL1/CHERP/CNOT10/CNOT6/CPSF3/CPSF4/CSNK1E/CSTF3/DCPS/DDX20/DDX23/DDX39A/DDX42/DDX49/DDX52/DKC1/DUS2/EDC3/EFTUD2/ELAVL1/EXOSC10/EXOSC2/EXOSC3/EXOSC8/FUS/GEMIN4/GEMIN6/GSPT2/GTF2F1/GTF2F2/GTF2H4/HNRNPA1/HNRNPA2B1/HNRNPA3/HNRNPD/HNRNPH1/HNRNPR/HNRNPUL1/HSD17B10/IGF2BP2/IGF2BP3/KHSRP/LSM2/LSM7/LSM8/LTV1/METTL3/MRM2/NCBP2/NDC1/NIP7/NOL11/NOL12/NOL9/NOP2/NOP56/NUP107/NUP188/NUP210/NUP42/NUP43/NUP88/PES1/PHF5A/PNO1/POLDIP3/POLR2F/PPIL1/PRMT5/PRPF38A/PRPF4/PRPF6/PSMD11/PSMD3/PUF60/PUS3/RAN/RBM8A/RBMX/RCL1/RIOK1/RPP40/RRP9/SET/SF1/SF3A2/SNRNP200/SNRPA/SNRPD3/SNRPE/SRSF10/SRSF3/TEX10/TFIP11/THOC2/TPRKB/TRA2B/TRDMT1/TRMT6/TRMT61A/TRMU/TSEN15/TSR1/U2AF2/UTP11/UTP15/UTP4/WDR12/WDR4/WDR46/WDR75/WDR77/YBX1/ZNF473 | 123 | REACTOME |
| REACTOME_MISMATCH_REPAIR | 0.000101178 | 0.001400854 | EXO1/MSH2/MSH6/PCNA/POLD3/RPA1/RPA3 | 7 | REACTOME |
| REACTOME_MITOTIC_G1_PHASE_AND_G1_S_TRANSITION | 1.48477E-27 | 4.11282E-25 | ABL1/AKT1/AKT2/CCNA2/CCNB1/CCND1/CCND2/CCNE1/CCNE2/CDC25A/CDC45/CDC6/CDC7/CDK1/CDK2/CDK4/CDK6/CDKN2C/CDT1/CKS1B/DBF4/DHFR/E2F1/E2F2/E2F3/E2F5/E2F6/FBXO5/GMNN/LIN9/MCM10/MCM2/MCM3/MCM4/MCM5/MCM6/MCM7/MCM8/MYBL2/ORC1/ORC5/ORC6/PCNA/POLA1/POLA2/POLE/POLE2/PRIM1/PRIM2/PSMD11/PSMD3/RBL1/RPA1/RPA3/RRM2/SKP2/SRC/TK1/TOP2A/TYMS/WEE1 | 61 | REACTOME |
| REACTOME_MITOTIC_G2_G2_M_PHASES | 4.11815E-12 | 1.86241E-10 | AURKA/BORA/CCNA2/CCNB1/CCNB2/CDC25A/CDC25B/CDC25C/CDK1/CDK2/CDK5RAP2/CENPF/CENPJ/CEP152/CEP41/CEP57/CEP78/CNTRL/CSNK1E/E2F1/E2F3/FOXM1/GTSE1/HAUS1/HAUS5/HAUS6/HAUS8/HMMR/LIN9/MYBL2/NDE1/NEK2/ODF2/OPTN/PHLDA1/PKMYT1/PLK1/PLK4/PSMD11/PSMD3/TPX2/TUBA1A/TUBA1B/TUBB/TUBB2B/TUBB4B/TUBG1/TUBGCP3/WEE1 | 49 | REACTOME |
| REACTOME_MITOTIC_METAPHASE_AND_ANAPHASE | 4.61459E-21 | 4.86949E-19 | ANAPC7/AURKB/BIRC5/BUB1/BUB1B/CCNB1/CCNB2/CDC20/CDCA5/CDCA8/CDK1/CENPA/CENPE/CENPF/CENPH/CENPI/CENPK/CENPL/CENPM/CENPN/CENPO/CENPP/CENPQ/CENPU/DSN1/ESPL1/FBXO5/INCENP/KIF18A/KIF2C/KNL1/KNTC1/LBR/LMNB1/MAD2L1/MIS12/NDC1/NDC80/NDE1/NUF2/NUP107/NUP188/NUP43/PLK1/PMF1/PPP1CC/PSMD11/PSMD3/PTTG1/RAN/RCC1/SGO1/SGO2/SKA1/SKA2/SPC24/SPC25/SPDL1/TMPO/TUBA1A/TUBA1B/TUBB2B/TUBB4B/UBE2C/UBE2I/UBE2S/VRK1/ZWILCH/ZWINT | 69 | REACTOME |
| REACTOME_MITOTIC_PROMETAPHASE | 3.98385E-30 | 1.26117E-27 | AURKB/BIRC5/BUB1/BUB1B/CCNB1/CCNB2/CDC20/CDCA5/CDCA8/CDK1/CDK5RAP2/CENPA/CENPE/CENPF/CENPH/CENPI/CENPJ/CENPK/CENPL/CENPM/CENPN/CENPO/CENPP/CENPQ/CENPU/CEP152/CEP41/CEP57/CEP78/CNTRL/CSNK1E/DSN1/HAUS1/HAUS5/HAUS6/HAUS8/INCENP/KIF18A/KIF2C/KNL1/KNTC1/MAD2L1/MIS12/NCAPD2/NCAPG/NCAPH/NDC80/NDE1/NEK2/NUF2/NUP107/NUP43/ODF2/PLK1/PLK4/PMF1/PPP1CC/SGO1/SGO2/SKA1/SKA2/SMC2/SMC4/SPC24/SPC25/SPDL1/TUBA1A/TUBA1B/TUBB/TUBB2B/TUBB4B/TUBG1/TUBGCP3/ZWILCH/ZWINT | 75 | REACTOME |
| REACTOME_MITOTIC_PROPHASE | 0.000263109 | 0.003258529 | AAAS/CCNB1/CCNB2/CDK1/H2AX/H2AZ1/H2AZ2/LMNB1/LPIN3/NCAPD3/NCAPG2/NCAPH2/NDC1/NUP107/NUP188/NUP210/NUP42/NUP43/NUP88/PLK1/SET/SMC2/SMC4/TMPO/VRK1 | 25 | REACTOME |
| REACTOME_MITOTIC_SPINDLE_CHECKPOINT | 2.95738E-22 | 4.36903E-20 | ANAPC7/AURKB/BIRC5/BUB1/BUB1B/CDC20/CDCA8/CENPA/CENPE/CENPF/CENPH/CENPI/CENPK/CENPL/CENPM/CENPN/CENPO/CENPP/CENPQ/CENPU/DSN1/INCENP/KIF18A/KIF2C/KNL1/KNTC1/MAD2L1/MIS12/NDC80/NDE1/NUF2/NUP107/NUP43/PLK1/PMF1/PPP1CC/SGO1/SGO2/SKA1/SKA2/SPC24/SPC25/SPDL1/UBE2C/UBE2S/ZWILCH/ZWINT | 47 | REACTOME |
| REACTOME_MRNA_SPLICING | 1.97311E-11 | 8.40849E-10 | ALYREF/CCAR1/CD2BP2/CHERP/CPSF3/CPSF4/CSTF3/DDX23/DDX42/EFTUD2/ELAVL1/FUS/GTF2F1/GTF2F2/HNRNPA1/HNRNPA2B1/HNRNPA3/HNRNPD/HNRNPH1/HNRNPR/HNRNPUL1/LSM2/LSM7/LSM8/NCBP2/PHF5A/POLR2F/PPIL1/PRPF38A/PRPF4/PRPF6/PUF60/RBM8A/RBMX/SF1/SF3A2/SNRNP200/SNRPA/SNRPD3/SNRPE/SRSF10/SRSF3/TFIP11/TRA2B/U2AF2/YBX1 | 46 | REACTOME |
| REACTOME_MRNA_SPLICING_MINOR_PATHWAY | 0.000273133 | 0.003362566 | DDX23/DDX42/EFTUD2/GTF2F1/GTF2F2/LSM2/NCBP2/POLR2F/PRPF6/SNRNP200/SNRPD3/SNRPE/YBX1 | 13 | REACTOME |
| REACTOME_MUSCLE_CONTRACTION | 2.93415E-09 | 3.26201E-07 | ACTC1/AHCYL1/ATP1A2/ATP1A3/ATP1B1/ATP1B3/ATP2B1/ATP2B2/CACNA1C/CACNA2D2/CACNB1/CACNB2/CACNG8/CALD1/CALM1/CAMK2A/CAMK2B/CAMK2G/FGF12/FGF13/FKBP1B/FXYD1/FXYD7/GUCY1A1/GUCY1B1/ITGB5/ITPR1/KAT2B/KCNIP2/KCNIP4/KCNJ11/KCNJ4/KCNK1/KCNK3/KCNK4/MYBPC1/MYL3/MYL6/NPPC/PAK1/RYR1/SCN2A/SCN2B/SCN3B/SCN4B/SCN8A/SLC8A2/SLC8A3/SORBS1/STIM1/TMOD2/TNNT1/TPM4 | 53 | REACTOME |
| REACTOME_NEGATIVE_REGULATION_OF_NMDA_RECEPTOR_MEDIATED_NEURONAL_TRANSMISSION | 6.7001E-08 | 4.63031E-06 | CALM1/CAMK2A/CAMK2B/CAMK2G/CAMK4/DLG2/DLG3/DLG4/GRIN1/GRIN2A/GRIN2C/LRRC7/NEFL | 13 | REACTOME |
| REACTOME_NERVOUS_SYSTEM_DEVELOPMENT | 2.79781E-13 | 1.4308E-10 | ABLIM1/ABLIM2/ACTB/ADGRV1/AGAP2/AKAP5/ANK1/ANK2/ANK3/ARPC1A/ARPC2/ARPC4/ARPC5/CACNA1C/CACNA1G/CACNB1/CACNB2/CACNB3/CACNB4/CAP2/CDK5R1/CNTNAP1/DLG3/DLG4/DNM1/DNM3/DOK6/DPYSL2/EFNA3/EFNB3/EPHA10/EPHA4/EPHA5/EPHB4/EPHB6/EVL/GFRA2/GRIN1/KALRN/KCNQ3/L1CAM/LAMC1/MAP2K1/MAPK1/MSN/MYL6/MYO9B/NELL2/NGEF/NTN4/PAK1/PAK3/PAK4/PAK6/PDLIM7/PFN1/PFN2/PIK3CB/PIK3R1/PIP5K1C/PPP3CB/PRKACB/PRNP/PSMA2/PSMA3/PSMA6/PSMC2/PSME1/PSME2/PSME4/RAP1GAP/RELN/RHOC/RND1/ROBO3/RPL10/RPL11/RPL12/RPL13A/RPL17/RPL18/RPL18A/RPL22L1/RPL23/RPL27A/RPL28/RPL29/RPL36/RPL36A/RPL39/RPLP0/RPLP1/RPS10/RPS11/RPS14/RPS16/RPS19/RPS2/RPS20/RPS23/RPS27/RPS27L/RPS28/RPS3A/RPS5/RPS6KA5/RPS7/RPS8/RPS9/SCN2A/SCN2B/SCN3B/SCN4B/SCN8A/SEM1/SH3GL2/SHC1/SLIT2/SPTB/SPTBN1/SPTBN2/SPTBN4/TUBA4A/TUBA8/TUBB2A/TUBB6/UBA52/UNC5A/UNC5D | 129 | REACTOME |
| REACTOME_NEUREXINS_AND_NEUROLIGINS | 4.53237E-09 | 4.13903E-07 | APBA1/APBA3/BEGAIN/DBNL/DLG2/DLG3/DLG4/DLGAP1/DLGAP3/EPB41L1/GRIN1/GRIN2A/GRIN2C/GRM5/LIN7B/LRRTM4/NRXN3/SHANK1/SHANK2/STX1A/STXBP1/SYT1/SYT12/SYT7 | 24 | REACTOME |
| REACTOME_NEURONAL_SYSTEM | 1.56768E-33 | 4.00856E-30 | ABCC8/ADCY1/ADCY2/ADCY5/AKAP5/ALDH2/APBA1/APBA3/ARHGEF9/BEGAIN/CACNA1A/CACNA2D2/CACNA2D3/CACNB1/CACNB2/CACNB3/CACNB4/CACNG8/CALM1/CAMK2A/CAMK2B/CAMK2G/CAMK4/CAMKK1/CAMKK2/CHRNA7/CHRNB2/CPLX1/DBNL/DLG2/DLG3/DLG4/DLGAP1/DLGAP3/EPB41L1/ERBB4/GABBR1/GABBR2/GABRA2/GABRA3/GABRA5/GABRB1/GABRB2/GABRB3/GABRG2/GAD2/GLRB/GLS2/GNAI1/GNAI2/GNAL/GNB2/GNB5/GNG2/GNG3/GNG5/GRIA1/GRIK2/GRIN1/GRIN2A/GRIN2C/GRIP1/GRIP2/GRM5/KCNA1/KCNA3/KCNAB1/KCNAB2/KCNB1/KCNC1/KCNC2/KCNC3/KCNC4/KCNH1/KCNH3/KCNJ11/KCNJ4/KCNJ9/KCNK1/KCNK3/KCNK4/KCNMA1/KCNN1/KCNQ3/KCNQ5/KCNS3/KIF17/LIN7B/LRRC7/LRRTM4/MAOA/MAPK1/MAPT/MDM2/NBEA/NEFL/NPTN/NRGN/NRXN3/NSF/PANX2/PLCB1/PPFIA2/PPFIA3/PPFIA4/PRKACB/PRKAG2/PRKAR1B/PRKAR2B/PRKCB/RAB3A/RASGRF2/RIMS1/RTN3/SHANK1/SHANK2/SLC17A7/SLC1A1/SLC1A2/SLC1A3/SLC6A1/SLC6A11/SLC6A13/SLITRK1/SNAP25/STX1A/STXBP1/SYN1/SYN2/SYN3/SYT1/SYT12/SYT7/TSPAN7/TSPOAP1/TUBA4A/TUBA8/TUBB2A/TUBB6/VAMP2 | 140 | REACTOME |
| REACTOME_NEUROTOXICITY_OF_CLOSTRIDIUM_TOXINS | 2.73386E-05 | 0.001028012 | SNAP25/STX1A/STX1B/SV2A/SYT1/VAMP1/VAMP2 | 7 | REACTOME |
| REACTOME_NEUROTRANSMITTER_RECEPTORS_AND_POSTSYNAPTIC_SIGNAL_TRANSMISSION | 6.15067E-18 | 5.24242E-15 | ADCY1/ADCY2/ADCY5/AKAP5/APBA1/ARHGEF9/CACNG8/CALM1/CAMK2A/CAMK2B/CAMK2G/CAMK4/CAMKK1/CAMKK2/CHRNA7/CHRNB2/DLG2/DLG3/DLG4/EPB41L1/ERBB4/GABBR1/GABBR2/GABRA2/GABRA3/GABRA5/GABRB1/GABRB2/GABRB3/GABRG2/GLRB/GNAI1/GNAI2/GNAL/GNB2/GNB5/GNG2/GNG3/GNG5/GRIA1/GRIK2/GRIN1/GRIN2A/GRIN2C/GRIP1/GRIP2/KCNJ4/KCNJ9/KIF17/LIN7B/LRRC7/MAPK1/MAPT/MDM2/NBEA/NEFL/NPTN/NRGN/NSF/PLCB1/PRKACB/PRKAG2/PRKAR1B/PRKAR2B/PRKCB/RASGRF2/TSPAN7/TUBA4A/TUBA8/TUBB2A/TUBB6 | 71 | REACTOME |
| REACTOME_NEUROTRANSMITTER_RELEASE_CYCLE | 9.58962E-13 | 4.08677E-10 | APBA1/CPLX1/GAD2/GLS2/LIN7B/MAOA/PPFIA2/PPFIA3/PPFIA4/RAB3A/RIMS1/SLC17A7/SLC1A1/SLC1A2/SLC1A3/SLC6A1/SLC6A11/SLC6A13/SNAP25/STX1A/STXBP1/SYN1/SYN2/SYN3/SYT1/TSPOAP1/VAMP2 | 27 | REACTOME |
| REACTOME_NITRIC_OXIDE_STIMULATES_GUANYLATE_CYCLASE | 0.000512846 | 0.010927899 | GUCY1A1/GUCY1B1/IRAG1/ITPR1/KCNMA1/PDE10A/PDE1A/PDE1B/PDE2A | 9 | REACTOME |
| REACTOME_NONSENSE_MEDIATED_DECAY_NMD | 8.66377E-08 | 5.68032E-06 | RPL10/RPL11/RPL12/RPL13A/RPL17/RPL18/RPL18A/RPL22L1/RPL23/RPL27A/RPL28/RPL29/RPL36/RPL36A/RPL39/RPLP0/RPLP1/RPS10/RPS11/RPS14/RPS16/RPS19/RPS2/RPS20/RPS23/RPS27/RPS27L/RPS28/RPS3A/RPS5/RPS7/RPS8/RPS9/SMG9/UBA52 | 35 | REACTOME |
| REACTOME_NOREPINEPHRINE_NEUROTRANSMITTER_RELEASE_CYCLE | 3.98061E-09 | 3.91478E-07 | CPLX1/MAOA/PPFIA2/PPFIA3/PPFIA4/RAB3A/RIMS1/SNAP25/STX1A/STXBP1/SYT1/TSPOAP1/VAMP2 | 13 | REACTOME |
| REACTOME_NS1_MEDIATED_EFFECTS_ON_HOST_PATHWAYS | 0.001673198 | 0.017245611 | AAAS/CPSF4/KPNA2/NDC1/NUP107/NUP188/NUP210/NUP42/NUP43/NUP88 | 10 | REACTOME |
| REACTOME_NUCLEAR_ENVELOPE_BREAKDOWN | 4.08646E-06 | 7.30754E-05 | AAAS/CCNB1/CCNB2/CDK1/LMNB1/LPIN3/NDC1/NUP107/NUP188/NUP210/NUP42/NUP43/NUP88/PLK1/TMPO/VRK1 | 16 | REACTOME |
| REACTOME_NUCLEAR_ENVELOPE_NE_REASSEMBLY | 4.259E-05 | 0.00062503 | CCNB1/CCNB2/CDK1/LBR/LMNB1/NDC1/NUP107/NUP188/NUP43/RAN/RCC1/TMPO/TUBA1A/TUBA1B/TUBB2B/TUBB4B/UBE2I/VRK1 | 18 | REACTOME |
| REACTOME_NUCLEAR_IMPORT_OF_REV_PROTEIN | 0.000335615 | 0.004064055 | AAAS/NDC1/NUP107/NUP188/NUP210/NUP42/NUP43/NUP88/RAN/RCC1 | 10 | REACTOME |
| REACTOME_NUCLEAR_PORE_COMPLEX_NPC_DISASSEMBLY | 0.000116316 | 0.001591092 | AAAS/CCNB1/CCNB2/CDK1/NDC1/NUP107/NUP188/NUP210/NUP42/NUP43/NUP88 | 11 | REACTOME |
| REACTOME_NUCLEOTIDE_BIOSYNTHESIS | 0.004335094 | 0.040363734 | CAD/DHODH/PAICS/PFAS/UMPS | 5 | REACTOME |
| REACTOME_NUCLEOTIDE_EXCISION_REPAIR | 0.00028313 | 0.003466392 | CUL4A/ERCC8/GTF2H4/LIG3/PARP1/PCNA/POLD3/POLE/POLE2/POLR2F/RFC2/RFC3/RFC4/RFC5/RPA1/RPA3/RUVBL1/SUMO2/UBE2I/UBE2N/XRCC1 | 21 | REACTOME |
| REACTOME_OPIOID_SIGNALLING | 7.57571E-12 | 1.80571E-09 | ADCY1/ADCY2/ADCY5/AHCYL1/CALM1/CAMK2A/CAMK2B/CAMK2G/CAMK4/CAMKK1/CAMKK2/GNAI1/GNAI2/GNAL/GNAQ/GNB2/GNB5/GNG2/GNG3/GNG5/ITPR1/MAPK1/NBEA/PDE1A/PDE1B/PDE4A/PDYN/PLCB1/PLCB4/PPP1R1B/PPP3CA/PPP3CB/PPP3R1/PRKACB/PRKAR1B/PRKAR2B | 36 | REACTOME |
| REACTOME_ORC1_REMOVAL_FROM_CHROMATIN | 5.95628E-05 | 0.000857086 | CCNA2/CDC6/CDK2/CDT1/MCM2/MCM3/MCM4/MCM5/MCM6/MCM7/MCM8/ORC1/ORC5/ORC6/PSMD11/PSMD3/SKP2 | 17 | REACTOME |
| REACTOME_ORGANELLE_BIOGENESIS_AND_MAINTENANCE | 6.59755E-05 | 0.000937191 | CCT2/CCT3/CCT5/CDK1/CDK5RAP2/CENPJ/CEP152/CEP41/CEP57/CEP78/CEP83/CEP89/CHCHD3/CNTRL/CSNK1E/EXOC4/GABPB1/HAUS1/HAUS5/HAUS6/HAUS8/IDH2/IFT81/MED1/MKS1/NDE1/NEK2/NR1D1/NRF1/ODF2/PLK1/PLK4/POLG2/RAB3IP/SCLT1/TCTN3/TFB2M/TTC30B/TUBA1A/TUBA1B/TUBB/TUBB2B/TUBB4B/TUBG1/TWNK | 45 | REACTOME |
| REACTOME_P75_NTR_RECEPTOR_MEDIATED_SIGNALLING | 0.001781082 | 0.02760137 | AKAP13/ARHGEF1/ARHGEF19/ARHGEF4/ARHGEF9/HDAC1/IRAK1/ITGB3BP/KALRN/LINGO1/MCF2/MCF2L/MYD88/NGEF/OMG/PLEKHG2/PLEKHG5/RASGRF2/RELA/RTN4/RTN4R/UBA52 | 22 | REACTOME |
| REACTOME_P75NTR_REGULATES_AXONOGENESIS | 0.003436895 | 0.045299691 | LINGO1/MCF2/OMG/RTN4/RTN4R | 5 | REACTOME |
| REACTOME_PCNA_DEPENDENT_LONG_PATCH_BASE_EXCISION_REPAIR | 1.65367E-08 | 4.88606E-07 | APEX1/FEN1/PCNA/POLD3/POLE/POLE2/RFC2/RFC3/RFC4/RFC5/RPA1/RPA3 | 12 | REACTOME |
| REACTOME_PHASE_0_RAPID_DEPOLARISATION | 1.24052E-07 | 7.93004E-06 | CACNA1C/CACNA2D2/CACNB1/CACNB2/CACNG8/CALM1/CAMK2A/CAMK2B/CAMK2G/FGF12/FGF13/SCN2A/SCN2B/SCN3B/SCN4B/SCN8A | 16 | REACTOME |
| REACTOME_PHOSPHORYLATION_OF_EMI1 | 2.30101E-05 | 0.000356439 | CCNB1/CDC20/CDK1/FBXO5/PLK1 | 5 | REACTOME |
| REACTOME_PHOSPHORYLATION_OF_THE_APC_C | 0.004775329 | 0.04319236 | ANAPC7/CCNB1/CDK1/PLK1/UBE2C/UBE2S | 6 | REACTOME |
| REACTOME_PKA_MEDIATED_PHOSPHORYLATION_OF_CREB | 0.001257149 | 0.022479224 | ADCY1/ADCY2/ADCY5/CALM1/NBEA/PRKACB/PRKAR1B/PRKAR2B | 8 | REACTOME |
| REACTOME_PLATELET_HOMEOSTASIS | 0.000826609 | 0.016134644 | ATP2B1/ATP2B2/CALM1/GNB2/GNB5/GNG2/GNG3/GNG5/GUCY1A1/GUCY1B1/IRAG1/ITPR1/KCNMA1/P2RX5/PDE10A/PDE1A/PDE1B/PDE2A/SLC8A2/SLC8A3/STIM1 | 21 | REACTOME |
| REACTOME_POLO_LIKE_KINASE_MEDIATED_EVENTS | 4.07347E-09 | 1.30823E-07 | CCNB1/CCNB2/CDC25A/CDC25C/CENPF/FOXM1/LIN9/MYBL2/PKMYT1/PLK1/WEE1 | 11 | REACTOME |
| REACTOME_POLYMERASE_SWITCHING | 1.21718E-08 | 3.64495E-07 | PCNA/POLA1/POLA2/POLD3/PRIM1/PRIM2/RFC2/RFC3/RFC4/RFC5 | 10 | REACTOME |
| REACTOME_POLYMERASE_SWITCHING_ON_THE_C_STRAND_OF_THE_TELOMERE | 3.65356E-07 | 7.63802E-06 | CHTF18/DSCC1/PCNA/POLA1/POLA2/POLD3/PRIM1/PRIM2/RFC2/RFC3/RFC4/RFC5 | 12 | REACTOME |
| REACTOME_POTASSIUM_CHANNELS | 1.62813E-06 | 7.70951E-05 | ABCC8/GABBR1/GABBR2/GNB2/GNB5/GNG2/GNG3/GNG5/KCNA1/KCNA3/KCNAB1/KCNAB2/KCNB1/KCNC1/KCNC2/KCNC3/KCNC4/KCNH1/KCNH3/KCNJ11/KCNJ4/KCNJ9/KCNK1/KCNK3/KCNK4/KCNMA1/KCNN1/KCNQ3/KCNQ5/KCNS3 | 30 | REACTOME |
| REACTOME_PRESYNAPTIC_DEPOLARIZATION_AND_CALCIUM_CHANNEL_OPENING | 6.74886E-05 | 0.00201509 | CACNA1A/CACNA2D2/CACNA2D3/CACNB1/CACNB2/CACNB3/CACNB4 | 7 | REACTOME |
| REACTOME_PROCESSING_OF_CAPPED_INTRON_CONTAINING_PRE_MRNA | 1.1874E-13 | 6.11923E-12 | AAAS/ALYREF/CCAR1/CD2BP2/CHERP/CPSF3/CPSF4/CSTF3/DDX23/DDX39A/DDX42/EFTUD2/ELAVL1/FUS/GTF2F1/GTF2F2/HNRNPA1/HNRNPA2B1/HNRNPA3/HNRNPD/HNRNPH1/HNRNPR/HNRNPUL1/LSM2/LSM7/LSM8/METTL3/NCBP2/NDC1/NUP107/NUP188/NUP210/NUP42/NUP43/NUP88/PHF5A/POLDIP3/POLR2F/PPIL1/PRPF38A/PRPF4/PRPF6/PUF60/RBM8A/RBMX/SF1/SF3A2/SNRNP200/SNRPA/SNRPD3/SNRPE/SRSF10/SRSF3/TFIP11/THOC2/TRA2B/U2AF2/YBX1 | 58 | REACTOME |
| REACTOME_PROCESSING_OF_DNA_DOUBLE_STRAND_BREAK_ENDS | 1.90343E-10 | 7.39999E-09 | ATRIP/BARD1/BLM/BRCA1/CCNA2/CDK2/CHEK1/CLSPN/DNA2/EXO1/H2AX/MDC1/NSD2/PIAS4/RBBP8/RFC2/RFC3/RFC4/RFC5/RMI1/RMI2/RPA1/RPA3/SIRT6/SUMO2/TIMELESS/TIPIN/TOP3A/UBE2I/UBE2N | 30 | REACTOME |
| REACTOME_PROCESSIVE_SYNTHESIS_ON_THE_C_STRAND_OF_THE_TELOMERE | 0.000587267 | 0.006708158 | BLM/DNA2/FEN1/PCNA/POLD3/RPA1/RPA3 | 7 | REACTOME |
| REACTOME_PROCESSIVE_SYNTHESIS_ON_THE_LAGGING_STRAND | 3.37598E-08 | 9.23602E-07 | DNA2/FEN1/PCNA/POLA1/POLA2/POLD3/PRIM1/PRIM2/RPA1/RPA3 | 10 | REACTOME |
| REACTOME_PROTEIN_PROTEIN_INTERACTIONS_AT_SYNAPSES | 2.53783E-08 | 2.02789E-06 | APBA1/APBA3/BEGAIN/DBNL/DLG2/DLG3/DLG4/DLGAP1/DLGAP3/EPB41L1/GRIA1/GRIN1/GRIN2A/GRIN2C/GRM5/LIN7B/LRRTM4/NRXN3/PPFIA2/PPFIA3/PPFIA4/RTN3/SHANK1/SHANK2/SLITRK1/STX1A/STXBP1/SYT1/SYT12/SYT7 | 30 | REACTOME |
| REACTOME_PTK6_REGULATES_CELL_CYCLE | 0.000643918 | 0.007280218 | CCND1/CCNE1/CDK2/CDK4 | 4 | REACTOME |
| REACTOME_RAS_ACTIVATION_UPON_CA2_INFLUX_THROUGH_NMDA_RECEPTOR | 3.6354E-06 | 0.000160271 | CALM1/CAMK2A/CAMK2B/CAMK2G/DLG2/DLG3/DLG4/GRIN1/LRRC7/NEFL/RASGRF2 | 11 | REACTOME |
| REACTOME_RECOGNITION_OF_DNA_DAMAGE_BY_PCNA_CONTAINING_REPLICATION_COMPLEX | 3.19375E-08 | 8.87307E-07 | CUL4A/DTL/PCNA/POLD3/POLE/POLE2/RAD18/RFC2/RFC3/RFC4/RFC5/RPA1/RPA3/USP1 | 14 | REACTOME |
| REACTOME_RECRUITMENT_OF_MITOTIC_CENTROSOME_PROTEINS_AND_COMPLEXES | 1.17983E-07 | 2.87308E-06 | CDK1/CDK5RAP2/CENPJ/CEP152/CEP41/CEP57/CEP78/CNTRL/CSNK1E/HAUS1/HAUS5/HAUS6/HAUS8/NDE1/NEK2/ODF2/PLK1/PLK4/TUBA1A/TUBB/TUBB4B/TUBG1/TUBGCP3 | 23 | REACTOME |
| REACTOME_RECRUITMENT_OF_NUMA_TO_MITOTIC_CENTROSOMES | 1.72273E-07 | 4.10491E-06 | CDK1/CDK5RAP2/CENPJ/CEP152/CEP41/CEP57/CEP78/CNTRL/CSNK1E/HAUS1/HAUS5/HAUS6/HAUS8/NDE1/NEK2/ODF2/PLK1/PLK4/TUBA1A/TUBA1B/TUBB/TUBB2B/TUBB4B/TUBG1/TUBGCP3 | 25 | REACTOME |
| REACTOME_RECYCLING_PATHWAY_OF_L1 | 0.003611891 | 0.046880369 | ACTB/DNM1/DNM3/DPYSL2/L1CAM/MAPK1/MSN/RPS6KA5/SH3GL2/TUBA4A/TUBA8/TUBB2A/TUBB6 | 13 | REACTOME |
| REACTOME_REGULATION_OF_EXPRESSION_OF_SLITS_AND_ROBOS | 4.17545E-07 | 2.37258E-05 | PSMA2/PSMA3/PSMA6/PSMC2/PSME1/PSME2/PSME4/ROBO3/RPL10/RPL11/RPL12/RPL13A/RPL17/RPL18/RPL18A/RPL22L1/RPL23/RPL27A/RPL28/RPL29/RPL36/RPL36A/RPL39/RPLP0/RPLP1/RPS10/RPS11/RPS14/RPS16/RPS19/RPS2/RPS20/RPS23/RPS27/RPS27L/RPS28/RPS3A/RPS5/RPS7/RPS8/RPS9/SEM1/SLIT2/UBA52 | 44 | REACTOME |
| REACTOME_REGULATION_OF_GLUCOKINASE_BY_GLUCOKINASE_REGULATORY_PROTEIN | 0.004069069 | 0.038370458 | AAAS/NDC1/NUP107/NUP188/NUP210/NUP42/NUP43/NUP88 | 8 | REACTOME |
| REACTOME_REGULATION_OF_INSULIN_SECRETION | 2.55028E-13 | 1.4308E-10 | ABCC8/ADCY5/ADRA2A/ADRA2C/AHCYL1/AKAP5/CACNA1A/CACNA1C/CACNA2D2/CACNB2/CACNB3/CHRM3/GNAI1/GNAI2/GNAQ/GNB2/GNB5/GNG2/GNG3/GNG5/ITPR1/KCNB1/KCNC2/KCNJ11/KCNS3/PLCB1/PRKACB/PRKAR1B/PRKAR2B/RAPGEF4/SNAP25/STX1A/STXBP1/SYT5/VAMP2 | 35 | REACTOME |
| REACTOME_REGULATION_OF_PLK1_ACTIVITY_AT_G2_M_TRANSITION | 1.09311E-09 | 3.97102E-08 | AURKA/BORA/CCNB1/CCNB2/CDK1/CDK5RAP2/CENPJ/CEP152/CEP41/CEP57/CEP78/CNTRL/CSNK1E/HAUS1/HAUS5/HAUS6/HAUS8/NDE1/NEK2/ODF2/OPTN/PLK1/PLK4/TUBA1A/TUBB/TUBB4B/TUBG1 | 27 | REACTOME |
| REACTOME_REGULATION_OF_TP53_ACTIVITY | 2.74428E-09 | 9.35589E-08 | AKT1/AKT2/ATRIP/AURKA/AURKB/BARD1/BLM/BRCA1/BRPF1/CCNA2/CDK1/CDK2/CHEK1/DNA2/DYRK2/EHMT1/EXO1/HDAC2/MAPKAP1/MBD3/NOC2L/PRMT5/RBBP8/RFC2/RFC3/RFC4/RFC5/RMI1/RMI2/RPA1/RPA3/SSRP1/SUPT16H/TAF15/TOP3A/TP53INP1/TPX2/ZNF385A | 38 | REACTOME |
| REACTOME_REGULATION_OF_TP53_ACTIVITY_THROUGH_PHOSPHORYLATION | 8.89137E-10 | 3.28388E-08 | ATRIP/AURKA/AURKB/BARD1/BLM/BRCA1/CCNA2/CDK2/CHEK1/DNA2/DYRK2/EXO1/NOC2L/RBBP8/RFC2/RFC3/RFC4/RFC5/RMI1/RMI2/RPA1/RPA3/SSRP1/SUPT16H/TAF15/TOP3A/TP53INP1/TPX2 | 28 | REACTOME |
| REACTOME_RESOLUTION_OF_ABASIC_SITES_AP_SITES | 2.32009E-11 | 9.70061E-10 | APEX1/FEN1/LIG3/MUTYH/NTHL1/OGG1/PARP1/PCNA/POLD3/POLE/POLE2/RFC2/RFC3/RFC4/RFC5/RPA1/RPA3/UNG/XRCC1 | 19 | REACTOME |
| REACTOME_RESOLUTION_OF_AP_SITES_VIA_THE_MULTIPLE_NUCLEOTIDE_PATCH_REPLACEMENT_PATHWAY | 1.90495E-08 | 5.55444E-07 | APEX1/FEN1/PARP1/PCNA/POLD3/POLE/POLE2/RFC2/RFC3/RFC4/RFC5/RPA1/RPA3 | 13 | REACTOME |
| REACTOME_RESOLUTION_OF_D_LOOP_STRUCTURES | 2.80292E-09 | 9.41103E-08 | BARD1/BLM/BRCA1/BRCA2/DNA2/EME1/EXO1/MUS81/RAD51/RAD51AP1/RAD51D/RBBP8/RMI1/RMI2/TOP3A/XRCC2 | 16 | REACTOME |
| REACTOME_RESOLUTION_OF_D_LOOP_STRUCTURES_THROUGH_SYNTHESIS_DEPENDENT_STRAND_ANNEALING_SDSA | 2.92328E-09 | 9.66865E-08 | BARD1/BLM/BRCA1/BRCA2/DNA2/EXO1/RAD51/RAD51AP1/RAD51D/RBBP8/RMI1/RMI2/TOP3A/XRCC2 | 14 | REACTOME |
| REACTOME_RESOLUTION_OF_SISTER_CHROMATID_COHESION | 7.3727E-24 | 1.48526E-21 | AURKB/BIRC5/BUB1/BUB1B/CCNB1/CCNB2/CDC20/CDCA5/CDCA8/CDK1/CENPA/CENPE/CENPF/CENPH/CENPI/CENPK/CENPL/CENPM/CENPN/CENPO/CENPP/CENPQ/CENPU/DSN1/INCENP/KIF18A/KIF2C/KNL1/KNTC1/MAD2L1/MIS12/NDC80/NDE1/NUF2/NUP107/NUP43/PLK1/PMF1/PPP1CC/SGO1/SGO2/SKA1/SKA2/SPC24/SPC25/SPDL1/TUBA1A/TUBA1B/TUBB2B/TUBB4B/ZWILCH/ZWINT | 52 | REACTOME |
| REACTOME_RESPONSE_OF_EIF2AK4_GCN2_TO_AMINO_ACID_DEFICIENCY | 5.46816E-12 | 1.74776E-09 | ATF4/CEBPG/EIF2AK4/EIF2S2/RPL10/RPL11/RPL12/RPL13A/RPL17/RPL18/RPL18A/RPL22L1/RPL23/RPL27A/RPL28/RPL29/RPL36/RPL36A/RPL39/RPLP0/RPLP1/RPS10/RPS11/RPS14/RPS16/RPS19/RPS2/RPS20/RPS23/RPS27/RPS27L/RPS28/RPS3A/RPS5/RPS7/RPS8/RPS9/TRIB3/UBA52 | 39 | REACTOME |
| REACTOME_RHO_GTPASE_EFFECTORS | 4.82934E-11 | 1.98182E-09 | ABL1/AURKB/BIRC5/BUB1/BUB1B/CDC20/CDC25C/CDCA8/CENPA/CENPE/CENPF/CENPH/CENPI/CENPK/CENPL/CENPM/CENPN/CENPO/CENPP/CENPQ/CENPU/DIAPH1/DIAPH3/DSN1/DVL2/H2AX/H2AZ1/H2AZ2/INCENP/IQGAP3/KDM1A/KIF14/KIF18A/KIF2C/KNL1/KNTC1/MAD2L1/MEN1/MIS12/NDC80/NDE1/NOXA1/NUF2/NUP107/NUP43/PKN3/PLK1/PMF1/PPP1CC/PRC1/SGO1/SGO2/SKA1/SKA2/SPC24/SPC25/SPDL1/SRC/TUBA1A/TUBA1B/TUBB2B/TUBB4B/ZWILCH/ZWINT | 64 | REACTOME |
| REACTOME_RHO_GTPASES_ACTIVATE_FORMINS | 2.35441E-21 | 2.74598E-19 | AURKB/BIRC5/BUB1/BUB1B/CDC20/CDCA8/CENPA/CENPE/CENPF/CENPH/CENPI/CENPK/CENPL/CENPM/CENPN/CENPO/CENPP/CENPQ/CENPU/DIAPH1/DIAPH3/DSN1/DVL2/INCENP/KIF18A/KIF2C/KNL1/KNTC1/MAD2L1/MIS12/NDC80/NDE1/NUF2/NUP107/NUP43/PLK1/PMF1/PPP1CC/SGO1/SGO2/SKA1/SKA2/SPC24/SPC25/SPDL1/SRC/TUBA1A/TUBA1B/TUBB2B/TUBB4B/ZWILCH/ZWINT | 52 | REACTOME |
| REACTOME_RHO_GTPASES_ACTIVATE_WASPS_AND_WAVES | 0.002167601 | 0.031137958 | ACTB/ARPC1A/ARPC2/ARPC4/ARPC5/CYFIP2/MAPK1/WASF1/WASF3/WIPF1/WIPF3 | 11 | REACTOME |
| REACTOME_RHOD_GTPASE_CYCLE | 0.000334499 | 0.004064055 | ARHGAP26/CPNE8/DBN1/DEPDC1B/DIAPH1/DIAPH3/EFHD2/LBR/LMNB1/MCAM/PIK3R2/RACGAP1/TMPO | 13 | REACTOME |
| REACTOME_RMTS_METHYLATE_HISTONE_ARGININES | 0.000552792 | 0.00648141 | CCND1/CDK4/DNMT3A/H2AC11/H2AX/H2AZ1/H2AZ2/PRMT1/PRMT5/PRMT6/SMARCA4/SMARCB1/SMARCC1/SMARCD1/WDR5/WDR77 | 16 | REACTOME |
| REACTOME_RNA_POLYMERASE_II_TRANSCRIPTION_TERMINATION | 0.000971381 | 0.010500393 | ALYREF/CPSF3/CPSF4/CSTF3/DDX39A/NCBP2/POLDIP3/RBM8A/SNRPD3/SNRPE/SRSF3/THOC2/U2AF2/ZNF473 | 14 | REACTOME |
| REACTOME_RRNA_MODIFICATION_IN_THE_NUCLEUS_AND_CYTOSOL | 0.000349466 | 0.004208784 | DDX49/DDX52/DKC1/NOL11/NOP2/NOP56/PNO1/RCL1/RRP9/UTP11/UTP15/UTP4/WDR46/WDR75 | 14 | REACTOME |
| REACTOME_RRNA_PROCESSING | 5.33922E-05 | 0.001750307 | EMG1/FBL/GAR1/GNL3/NOP58/RBM28/RIOK2/RIOK3/RPL10/RPL11/RPL12/RPL13A/RPL17/RPL18/RPL18A/RPL22L1/RPL23/RPL27A/RPL28/RPL29/RPL36/RPL36A/RPL39/RPLP0/RPLP1/RPS10/RPS11/RPS14/RPS16/RPS19/RPS2/RPS20/RPS23/RPS27/RPS27L/RPS28/RPS3A/RPS5/RPS7/RPS8/RPS9/TBL3/TRMT112/UBA52 | 44 | REACTOME |
| REACTOME_S_PHASE | 3.77617E-21 | 4.184E-19 | AKT1/AKT2/ANAPC7/CCNA2/CCND1/CCNE1/CCNE2/CDC25A/CDC25B/CDC45/CDC6/CDCA5/CDK2/CDK4/CDT1/CKS1B/DNA2/E2F1/E2F5/ESCO2/FEN1/GINS1/GINS2/GINS3/GINS4/LIN9/MCM2/MCM3/MCM4/MCM5/MCM6/MCM7/MCM8/ORC1/ORC5/ORC6/PCNA/POLA1/POLA2/POLD3/POLE/POLE2/PRIM1/PRIM2/PSMD11/PSMD3/RFC2/RFC3/RFC4/RFC5/RPA1/RPA3/SKP2/UBE2C/UBE2S/WEE1 | 56 | REACTOME |
| REACTOME_SARS_COV_INFECTIONS | 0.00238285 | 0.033477736 | ATP1A2/ATP1A3/ATP1B1/ATP1B3/BRMS1/CHD3/DAD1/DDOST/EDEM2/FXYD1/FXYD7/GANAB/HDAC1/HMG20B/IFNAR2/IMPDH1/MAN1B1/MGAT4B/MGAT4C/MOGS/NFE2L2/REST/RPN1/RPN2/S1PR1/SAP30/SRPK2/STT3A/TUSC3/TYK2/UBA52 | 31 | REACTOME |
| REACTOME_SELENOAMINO_ACID_METABOLISM | 4.14348E-08 | 3.02711E-06 | AHCY/PSTK/RPL10/RPL11/RPL12/RPL13A/RPL17/RPL18/RPL18A/RPL22L1/RPL23/RPL27A/RPL28/RPL29/RPL36/RPL36A/RPL39/RPLP0/RPLP1/RPS10/RPS11/RPS14/RPS16/RPS19/RPS2/RPS20/RPS23/RPS27/RPS27L/RPS28/RPS3A/RPS5/RPS7/RPS8/RPS9/UBA52 | 36 | REACTOME |
| REACTOME_SENSORY_PROCESSING_OF_SOUND | 0.000466788 | 0.010289458 | ACTB/ATP2B1/ATP2B2/BSN/CABP1/CACNA2D2/CACNB2/EPB41L1/KCNMA1/LHFPL5/MSN/PCLO/RAB3A/RIPOR2/SNAP25/SPTBN1/STX1A/SYN1/SYP/VAMP2 | 20 | REACTOME |
| REACTOME_SEPARATION_OF_SISTER_CHROMATIDS | 2.21899E-17 | 1.82121E-15 | ANAPC7/AURKB/BIRC5/BUB1/BUB1B/CDC20/CDCA5/CDCA8/CENPA/CENPE/CENPF/CENPH/CENPI/CENPK/CENPL/CENPM/CENPN/CENPO/CENPP/CENPQ/CENPU/DSN1/ESPL1/INCENP/KIF18A/KIF2C/KNL1/KNTC1/MAD2L1/MIS12/NDC80/NDE1/NUF2/NUP107/NUP43/PLK1/PMF1/PPP1CC/PSMD11/PSMD3/PTTG1/SGO1/SGO2/SKA1/SKA2/SPC24/SPC25/SPDL1/TUBA1A/TUBA1B/TUBB2B/TUBB4B/UBE2C/UBE2S/ZWILCH/ZWINT | 56 | REACTOME |
| REACTOME_SEROTONIN_NEUROTRANSMITTER_RELEASE_CYCLE | 6.52658E-12 | 1.80571E-09 | CPLX1/PPFIA2/PPFIA3/PPFIA4/RAB3A/RIMS1/SNAP25/STX1A/STXBP1/SYN1/SYN2/SYN3/SYT1/TSPOAP1/VAMP2 | 15 | REACTOME |
| REACTOME_SIGNALING_BY_GPCR | 8.14141E-07 | 4.16352E-05 | ACKR3/ADCY1/ADCY2/ADCY5/ADGRE5/ADRA1A/ADRA1B/ADRA2A/ADRA2C/ADRB1/AHCYL1/AKAP13/ARHGEF1/ARHGEF19/ARHGEF25/ARHGEF4/ARHGEF9/ARRB1/CALM1/CAMK2A/CAMK2B/CAMK2G/CAMK4/CAMKK1/CAMKK2/CCK/CHRM1/CHRM3/CHRM4/CORT/CRHBP/CRHR1/CX3CL1/CXCR5/DAGLA/DGKB/DGKE/DGKG/ECE2/FZD5/GABBR1/GABBR2/GNAI1/GNAI2/GNAL/GNAQ/GNAZ/GNB2/GNB5/GNG2/GNG3/GNG5/GPR143/GPR176/GPR27/GPR37L1/GRK3/GRM2/GRM3/GRM5/GRM8/HRH2/HRH3/ITPR1/KALRN/LPAR2/MAPK1/MCF2/MCF2L/MGLL/NBEA/NGEF/NMB/NPY/NPY1R/NTSR2/OPRL1/P2RY11/P2RY14/PAK1/PDE10A/PDE1A/PDE1B/PDE2A/PDE3B/PDE4A/PDE8B/PDYN/PENK/PIK3R1/PLCB1/PLCB4/PLEKHG2/PLEKHG5/PLPPR2/PLPPR3/PLPPR4/PNOC/PPP1R1B/PPP3CA/PPP3CB/PPP3R1/PRKACB/PRKAR1B/PRKAR2B/PRKCB/PRKCE/RASGRF2/RASGRP1/RGR/RGS20/RGS4/RGS7/RGS8/RHOC/S1PR1/S1PR2/S1PR3/SMO/SST/SSTR2/TAC1/TAC3/VIPR1/WNT7A | 125 | REACTOME |
| REACTOME_SIGNALING_BY_HEDGEHOG | 0.002506671 | 0.034834552 | ADCY1/ADCY2/ADCY5/ARRB1/BOC/BTRC/DERL2/DISP2/GPC5/KIF3A/OFD1/OS9/P4HB/PRKACB/PRKAR1B/PRKAR2B/PSMA2/PSMA3/PSMA6/PSMC2/PSME1/PSME2/PSME4/SEM1/SMO/SYVN1/TUBA4A/TUBB2A/TUBB6/UBA52 | 30 | REACTOME |
| REACTOME_SIGNALING_BY_INSULIN_RECEPTOR | 0.00147938 | 0.024563474 | ATP6V0A1/ATP6V0C/ATP6V0E2/ATP6V1B2/ATP6V1C1/ATP6V1D/ATP6V1E1/ATP6V1G2/FGF17/FGF22/FGF9/FGFR3/FLT3LG/MAPK1/PDE3B/PIK3CB/PIK3R1/SHC1/TRIB3 | 19 | REACTOME |
| REACTOME_SIGNALING_BY_RECEPTOR_TYROSINE_KINASES | 0.000408401 | 0.009323943 | ACTB/AHCYL1/ATP6V0A1/ATP6V0C/ATP6V0E2/ATP6V1B2/ATP6V1C1/ATP6V1D/ATP6V1E1/ATP6V1G2/BAX/CALM1/CDK5R1/CDK5R2/CYFIP2/DLG4/DNM1/DNM3/DOCK3/DUSP4/DUSP6/ELK1/ELMO1/EPS15/ERBB4/FGF17/FGF22/FGF9/FGFR3/FLRT2/FLT3LG/FRS3/GABRB1/GABRB2/GABRB3/GABRG2/GFAP/HDAC1/HIF1A/ID3/ID4/ITPR1/KIT/LAMA4/LAMA5/LAMB2/LAMC1/MAP2K1/MAPK1/MATK/MDK/MEF2A/MEF2C/NRG3/NTRK2/PAK1/PAK3/PDE3B/PDGFC/PIK3CB/PIK3R1/POLR2H/POLR2J/PRKACB/PRKCB/PRKCE/PRKCZ/PTBP1/PTK2B/PTPN12/PTPRO/REST/RIT1/RIT2/RPS6KA5/SH2B2/SH3GL2/SHC1/SPRY1/STAM/STMN1/THBS3/TRIB3/UBA52/WASF1/WASF3/WWP1 | 87 | REACTOME |
| REACTOME_SIGNALING_BY_RHO_GTPASES_MIRO_GTPASES_AND_RHOBTB3 | 8.18737E-07 | 1.61993E-05 | AAAS/ABCD3/ABL1/ARAP3/ARHGAP11A/ARHGAP26/ARHGAP42/ARHGEF2/ARHGEF39/ARHGEF40/AURKB/BIRC5/BUB1/BUB1B/CCNE1/CCT2/CDC20/CDC25C/CDCA8/CENPA/CENPE/CENPF/CENPH/CENPI/CENPK/CENPL/CENPM/CENPN/CENPO/CENPP/CENPQ/CENPU/CPNE8/DBN1/DEPDC1B/DIAPH1/DIAPH3/DSN1/DVL2/ECT2/EFHD2/FARP1/FARP2/FGD1/H2AX/H2AZ1/H2AZ2/INCENP/IQGAP3/KCTD3/KDM1A/KIF14/KIF18A/KIF2C/KNL1/KNTC1/LBR/LMNB1/MAD2L1/MCAM/MEN1/MIS12/MSI2/NDC80/NDE1/NOXA1/NUF2/NUP107/NUP43/PIK3R2/PKN3/PLK1/PMF1/PPP1CC/PRC1/RACGAP1/RBMX/SGO1/SGO2/SKA1/SKA2/SPC24/SPC25/SPDL1/SRC/TMPO/TRA2B/TRIO/TUBA1A/TUBA1B/TUBB2B/TUBB4B/VANGL2/VAV2/VHL/ZNF512B/ZWILCH/ZWINT | 98 | REACTOME |
| REACTOME_SIGNALING_BY_ROBO_RECEPTORS | 3.58929E-09 | 3.7634E-07 | AKAP5/CAP2/EVL/MYO9B/NELL2/PAK1/PAK3/PAK4/PAK6/PFN1/PFN2/PPP3CB/PRKACB/PSMA2/PSMA3/PSMA6/PSMC2/PSME1/PSME2/PSME4/ROBO3/RPL10/RPL11/RPL12/RPL13A/RPL17/RPL18/RPL18A/RPL22L1/RPL23/RPL27A/RPL28/RPL29/RPL36/RPL36A/RPL39/RPLP0/RPLP1/RPS10/RPS11/RPS14/RPS16/RPS19/RPS2/RPS20/RPS23/RPS27/RPS27L/RPS28/RPS3A/RPS5/RPS7/RPS8/RPS9/SEM1/SLIT2/UBA52 | 57 | REACTOME |
| REACTOME_SNRNP_ASSEMBLY | 5.3591E-06 | 9.27795E-05 | AAAS/DDX20/GEMIN4/GEMIN6/NCBP2/NDC1/NUP107/NUP188/NUP210/NUP42/NUP43/NUP88/PRMT5/SNRPD3/SNRPE/WDR77 | 16 | REACTOME |
| REACTOME_SRP_DEPENDENT_COTRANSLATIONAL_PROTEIN_TARGETING_TO_MEMBRANE | 1.13757E-11 | 2.42397E-09 | DDOST/RPL10/RPL11/RPL12/RPL13A/RPL17/RPL18/RPL18A/RPL22L1/RPL23/RPL27A/RPL28/RPL29/RPL36/RPL36A/RPL39/RPLP0/RPLP1/RPN1/RPN2/RPS10/RPS11/RPS14/RPS16/RPS19/RPS2/RPS20/RPS23/RPS27/RPS27L/RPS28/RPS3A/RPS5/RPS7/RPS8/RPS9/SEC11A/SEC61A1/SEC61B/SSR4/UBA52 | 41 | REACTOME |
| REACTOME_SUMOYLATION | 2.33488E-07 | 5.12287E-06 | AAAS/AURKA/AURKB/BIRC5/BLM/BRCA1/CBX2/CBX8/CDCA8/DNMT1/DNMT3A/HDAC2/INCENP/MDC1/NDC1/NR4A2/NUP107/NUP188/NUP210/NUP42/NUP43/NUP88/PARP1/PCGF2/PCNA/PHC1/PIAS4/RPA1/SMC6/SUMO2/TFAP2A/TOP2A/TRIM28/UBA2/UBE2I/UHRF2/VHL/ZBED1 | 38 | REACTOME |
| REACTOME_SUMOYLATION_OF_CHROMATIN_ORGANIZATION_PROTEINS | 0.000208739 | 0.002673789 | AAAS/CBX2/CBX8/HDAC2/NDC1/NUP107/NUP188/NUP210/NUP42/NUP43/NUP88/PCGF2/PHC1/SUMO2/UBE2I/ZBED1 | 16 | REACTOME |
| REACTOME_SUMOYLATION_OF_DNA_DAMAGE_RESPONSE_AND_REPAIR_PROTEINS | 8.69741E-07 | 1.70562E-05 | AAAS/BLM/BRCA1/CBX2/CBX8/MDC1/NDC1/NUP107/NUP188/NUP210/NUP42/NUP43/NUP88/PARP1/PCGF2/PHC1/PIAS4/RPA1/SMC6/SUMO2/UBE2I | 21 | REACTOME |
| REACTOME_SUMOYLATION_OF_DNA_METHYLATION_PROTEINS | 0.000166873 | 0.002201135 | CBX2/CBX8/DNMT1/DNMT3A/PCGF2/PHC1/UBE2I | 7 | REACTOME |
| REACTOME_SUMOYLATION_OF_DNA_REPLICATION_PROTEINS | 1.08158E-08 | 3.28326E-07 | AAAS/AURKA/AURKB/BIRC5/CDCA8/INCENP/NDC1/NUP107/NUP188/NUP210/NUP42/NUP43/NUP88/PCNA/PIAS4/SUMO2/TOP2A/UBE2I | 18 | REACTOME |
| REACTOME_SUMOYLATION_OF_RNA_BINDING_PROTEINS | 1.93013E-05 | 0.000303345 | AAAS/CBX2/CBX8/NDC1/NUP107/NUP188/NUP210/NUP42/NUP43/NUP88/PCGF2/PHC1/SUMO2/UBE2I | 14 | REACTOME |
| REACTOME_SUMOYLATION_OF_SUMOYLATION_PROTEINS | 8.73378E-05 | 0.001217237 | AAAS/NDC1/NUP107/NUP188/NUP210/NUP42/NUP43/NUP88/PIAS4/SUMO2/UBE2I | 11 | REACTOME |
| REACTOME_SUMOYLATION_OF_UBIQUITINYLATION_PROTEINS | 0.000256786 | 0.003214902 | AAAS/NDC1/NUP107/NUP188/NUP210/NUP42/NUP43/NUP88/PIAS4/UBE2I/VHL | 11 | REACTOME |
| REACTOME_SWITCHING_OF_ORIGINS_TO_A_POST_REPLICATIVE_STATE | 4.34246E-06 | 7.63722E-05 | ANAPC7/CCNA2/CCNE1/CCNE2/CDC6/CDK2/CDT1/MCM2/MCM3/MCM4/MCM5/MCM6/MCM7/MCM8/ORC1/ORC5/ORC6/PSMD11/PSMD3/SKP2/UBE2C/UBE2S | 22 | REACTOME |
| REACTOME_SYNTHESIS_OF_DNA | 3.33273E-17 | 2.46178E-15 | ANAPC7/CCNA2/CCNE1/CCNE2/CDC45/CDC6/CDK2/CDT1/DNA2/FEN1/GINS1/GINS2/GINS3/GINS4/MCM2/MCM3/MCM4/MCM5/MCM6/MCM7/MCM8/ORC1/ORC5/ORC6/PCNA/POLA1/POLA2/POLD3/POLE/POLE2/PRIM1/PRIM2/PSMD11/PSMD3/RFC2/RFC3/RFC4/RFC5/RPA1/RPA3/SKP2/UBE2C/UBE2S | 43 | REACTOME |
| REACTOME_TELOMERE_C_STRAND_LAGGING_STRAND_SYNTHESIS | 2.65332E-10 | 1.01375E-08 | BLM/CHTF18/DNA2/DSCC1/FEN1/PCNA/POLA1/POLA2/POLD3/PRIM1/PRIM2/RFC2/RFC3/RFC4/RFC5/RPA1/RPA3 | 17 | REACTOME |
| REACTOME_TELOMERE_EXTENSION_BY_TELOMERASE | 0.002121693 | 0.021468823 | CCNA2/CDK2/DKC1/PIF1/RUVBL1/RUVBL2/WRAP53 | 7 | REACTOME |
| REACTOME_TELOMERE_MAINTENANCE | 8.51355E-08 | 2.14387E-06 | BLM/CCNA2/CDK2/CHTF18/DKC1/DNA2/DSCC1/FEN1/H2AX/H2AZ1/H2AZ2/PCNA/PIF1/POLA1/POLA2/POLD3/POLR2F/PRIM1/PRIM2/RFC2/RFC3/RFC4/RFC5/RPA1/RPA3/RUVBL1/RUVBL2/WRAP53 | 28 | REACTOME |
| REACTOME_TERMINATION_OF_TRANSLESION_DNA_SYNTHESIS | 3.41524E-05 | 0.000514841 | PCLAF/PCNA/POLD3/POLE/POLE2/RFC2/RFC3/RFC4/RFC5/RPA1/RPA3 | 11 | REACTOME |
| REACTOME_TFAP2A_ACTS_AS_A_TRANSCRIPTIONAL_REPRESSOR_DURING_RETINOIC_ACID_INDUCED_CELL_DIFFERENTIATION | 0.005175362 | 0.045874411 | MYBL2/NOP2/TFAP2A | 3 | REACTOME |
| REACTOME_TP53_REGULATES_TRANSCRIPTION_OF_CELL_CYCLE_GENES | 3.47596E-08 | 9.39358E-07 | AURKA/CCNA2/CCNB1/CCNE1/CCNE2/CDC25C/CDK1/CDK2/CENPJ/CNOT10/CNOT6/E2F1/E2F7/E2F8/PCNA/PRMT1/RBL1/ZNF385A | 18 | REACTOME |
| REACTOME_TP53_REGULATES_TRANSCRIPTION_OF_DNA_REPAIR_GENES | 6.82121E-05 | 0.00096279 | BRCA1/CHEK1/FANCC/FANCD2/FANCI/GTF2F1/GTF2F2/GTF2H4/MDC1/MSH2/NELFA/NELFCD/POLR2F/RAD51D/SSRP1/SUPT16H | 16 | REACTOME |
| REACTOME_TP53_REGULATES_TRANSCRIPTION_OF_GENES_INVOLVED_IN_G1_CELL_CYCLE_ARREST | 4.54391E-06 | 7.92859E-05 | CCNA2/CCNE1/CCNE2/CDK2/E2F1/E2F7/E2F8/ZNF385A | 8 | REACTOME |
| REACTOME_TP53_REGULATES_TRANSCRIPTION_OF_GENES_INVOLVED_IN_G2_CELL_CYCLE_ARREST | 4.87783E-05 | 0.000711136 | AURKA/CCNB1/CDC25C/CDK1/PCNA/PRMT1/RBL1/ZNF385A | 8 | REACTOME |
| REACTOME_TRAFFICKING_OF_AMPA_RECEPTORS | 3.61789E-06 | 0.000160271 | AKAP5/CACNG8/CAMK2A/CAMK2B/CAMK2G/DLG4/EPB41L1/GRIA1/GRIP1/GRIP2/MDM2/NSF/PRKCB/TSPAN7 | 14 | REACTOME |
| REACTOME_TRANSCRIPTION_COUPLED_NUCLEOTIDE_EXCISION_REPAIR_TC_NER | 0.000642305 | 0.007280218 | CUL4A/ERCC8/GTF2H4/LIG3/PCNA/POLD3/POLE/POLE2/POLR2F/RFC2/RFC3/RFC4/RFC5/RPA1/RPA3/XRCC1 | 16 | REACTOME |
| REACTOME_TRANSCRIPTION_OF_E2F_TARGETS_UNDER_NEGATIVE_CONTROL_BY_DREAM_COMPLEX | 7.80731E-05 | 0.001095 | CDC25A/CDC6/E2F1/E2F5/LIN9/PCNA/RBL1/TOP2A | 8 | REACTOME |
| REACTOME_TRANSCRIPTION_OF_E2F_TARGETS_UNDER_NEGATIVE_CONTROL_BY_P107_RBL1_AND_P130_RBL2_IN_COMPLEX_WITH_HDAC1 | 0.000166873 | 0.002201135 | CCNA2/CDK1/E2F1/E2F5/LIN9/MYBL2/RBL1 | 7 | REACTOME |
| REACTOME_TRANSCRIPTIONAL_REGULATION_BY_E2F6 | 1.65927E-06 | 3.14269E-05 | BRCA1/CBX3/CDC7/CHEK1/E2F1/E2F6/EHMT1/EZH2/PCGF2/PHC1/RAD51/RBBP8/RRM2 | 13 | REACTOME |
| REACTOME_TRANSCRIPTIONAL_REGULATION_BY_THE_AP_2_TFAP2_FAMILY_OF_TRANSCRIPTION_FACTORS | 0.000199303 | 0.002582783 | ATAD2/CITED1/DEK/EGFR/KCTD15/KDM5B/MYBL2/NOP2/TFAP2A/UBE2I/YEATS4 | 11 | REACTOME |
| REACTOME_TRANSCRIPTIONAL_REGULATION_BY_TP53 | 1.77705E-09 | 6.25071E-08 | AKT1/AKT2/ATRIP/AURKA/AURKB/BARD1/BIRC5/BLM/BRCA1/BRPF1/CASP2/CCNA2/CCNB1/CCNE1/CCNE2/CDC25C/CDK1/CDK2/CENPJ/CHEK1/CNOT10/CNOT6/DNA2/DYRK2/E2F1/E2F7/E2F8/EHMT1/EXO1/FANCC/FANCD2/FANCI/GTF2F1/GTF2F2/GTF2H4/HDAC2/MAPKAP1/MBD3/MDC1/MSH2/NELFA/NELFCD/NOC2L/PCNA/POLR2F/PRMT1/PRMT5/RAD51D/RBBP8/RBL1/RFC2/RFC3/RFC4/RFC5/RMI1/RMI2/RPA1/RPA3/SSRP1/SUPT16H/TAF15/TOP3A/TP53I3/TP53INP1/TPX2/ZNF385A | 66 | REACTOME |
| REACTOME_TRANSLATION | 0.000363328 | 0.008445732 | CARS1/CARS2/DDOST/EEF1A2/EIF2S2/EIF3B/EIF3M/EIF4A1/EIF4EBP1/MRPL17/MRPL32/MRPL36/MRPS33/OXA1L/RPL10/RPL11/RPL12/RPL13A/RPL17/RPL18/RPL18A/RPL22L1/RPL23/RPL27A/RPL28/RPL29/RPL36/RPL36A/RPL39/RPLP0/RPLP1/RPN1/RPN2/RPS10/RPS11/RPS14/RPS16/RPS19/RPS2/RPS20/RPS23/RPS27/RPS27L/RPS28/RPS3A/RPS5/RPS7/RPS8/RPS9/SEC11A/SEC61A1/SEC61B/SSR4/TRMT112/UBA52 | 55 | REACTOME |
| REACTOME_TRANSLATION_OF_SARS_COV_2_STRUCTURAL_PROTEINS | 0.001185577 | 0.02134873 | DAD1/DDOST/EDEM2/GANAB/MAN1B1/MGAT4B/MGAT4C/MOGS/RPN1/RPN2/SRPK2/STT3A/TUSC3/UBA52 | 14 | REACTOME |
| REACTOME_TRANSLESION_SYNTHESIS_BY_POLH | 0.000587267 | 0.006708158 | PCNA/RFC2/RFC3/RFC4/RFC5/RPA1/RPA3 | 7 | REACTOME |
| REACTOME_TRANSLESION_SYNTHESIS_BY_POLK | 2.61053E-06 | 4.78093E-05 | MAD2L2/PCNA/REV3L/RFC2/RFC3/RFC4/RFC5/RPA1/RPA3 | 9 | REACTOME |
| REACTOME_TRANSLESION_SYNTHESIS_BY_Y_FAMILY_DNA_POLYMERASES_BYPASSES_LESIONS_ON_DNA_TEMPLATE | 9.76312E-06 | 0.000161456 | MAD2L2/PCLAF/PCNA/POLD3/POLE/POLE2/REV3L/RFC2/RFC3/RFC4/RFC5/RPA1/RPA3 | 13 | REACTOME |
| REACTOME_TRANSLOCATION_OF_SLC2A4_GLUT4_TO_THE_PLASMA_MEMBRANE | 0.00364327 | 0.046880369 | ACTB/CALM1/KIF3A/KIF3B/KIFAP3/MYO5A/PRKAG2/RAB13/STX4/TBC1D1/TUBA4A/TUBA8/TUBB2A/TUBB6/VAMP2/YWHAG/YWHAH | 17 | REACTOME |
| REACTOME_TRANSMISSION_ACROSS_CHEMICAL_SYNAPSES | 3.71332E-30 | 4.74748E-27 | ADCY1/ADCY2/ADCY5/AKAP5/ALDH2/APBA1/ARHGEF9/CACNA1A/CACNA2D2/CACNA2D3/CACNB1/CACNB2/CACNB3/CACNB4/CACNG8/CALM1/CAMK2A/CAMK2B/CAMK2G/CAMK4/CAMKK1/CAMKK2/CHRNA7/CHRNB2/CPLX1/DLG2/DLG3/DLG4/EPB41L1/ERBB4/GABBR1/GABBR2/GABRA2/GABRA3/GABRA5/GABRB1/GABRB2/GABRB3/GABRG2/GAD2/GLRB/GLS2/GNAI1/GNAI2/GNAL/GNB2/GNB5/GNG2/GNG3/GNG5/GRIA1/GRIK2/GRIN1/GRIN2A/GRIN2C/GRIP1/GRIP2/KCNJ4/KCNJ9/KIF17/LIN7B/LRRC7/MAOA/MAPK1/MAPT/MDM2/NBEA/NEFL/NPTN/NRGN/NSF/PLCB1/PPFIA2/PPFIA3/PPFIA4/PRKACB/PRKAG2/PRKAR1B/PRKAR2B/PRKCB/RAB3A/RASGRF2/RIMS1/SLC17A7/SLC1A1/SLC1A2/SLC1A3/SLC6A1/SLC6A11/SLC6A13/SNAP25/STX1A/STXBP1/SYN1/SYN2/SYN3/SYT1/TSPAN7/TSPOAP1/TUBA4A/TUBA8/TUBB2A/TUBB6/VAMP2 | 104 | REACTOME |
| REACTOME_TRANSPORT_OF_INORGANIC_CATIONS_ANIONS_AND_AMINO_ACIDS_OLIGOPEPTIDES | 0.000487453 | 0.010653139 | AHCYL2/CALM1/CTNS/SLC12A4/SLC12A5/SLC15A2/SLC17A7/SLC1A1/SLC1A2/SLC1A3/SLC1A4/SLC24A4/SLC25A18/SLC25A22/SLC26A6/SLC3A2/SLC43A2/SLC4A10/SLC4A2/SLC6A15/SLC7A11/SLC8A2/SLC8A3/SLC9A6/SLC9A8 | 25 | REACTOME |
| REACTOME_TRANSPORT_OF_MATURE_MRNAS_DERIVED_FROM_INTRONLESS_TRANSCRIPTS | 0.000151752 | 0.002063078 | AAAS/ALYREF/CPSF3/CPSF4/NCBP2/NDC1/NUP107/NUP188/NUP210/NUP42/NUP43/NUP88 | 12 | REACTOME |
| REACTOME_TRANSPORT_OF_MATURE_TRANSCRIPT_TO_CYTOPLASM | 0.000170021 | 0.002229385 | AAAS/ALYREF/CPSF3/CPSF4/DDX39A/NCBP2/NDC1/NUP107/NUP188/NUP210/NUP42/NUP43/NUP88/POLDIP3/RBM8A/SRSF3/THOC2/U2AF2 | 18 | REACTOME |
| REACTOME_TRANSPORT_OF_SMALL_MOLECULES | 0.00011277 | 0.003134264 | ABCB8/ABCB9/ABCD2/ABCG4/ADCY1/ADCY2/ADCY5/ADD3/AHCYL2/ANO5/APOD/AQP11/AQP3/AQP4/ASIC2/ATP13A2/ATP1A2/ATP1A3/ATP1B1/ATP1B3/ATP2B1/ATP2B2/ATP6V0A1/ATP6V0C/ATP6V0E2/ATP6V1B2/ATP6V1C1/ATP6V1D/ATP6V1E1/ATP6V1G2/ATP7B/ATP9A/BMP1/CA4/CALM1/CAMK2A/CAMK2B/CAMK2G/CLCN4/CTNS/CYGB/DERL2/DMTN/EIF2S2/FKBP1B/FXYD1/FXYD7/GNB2/GNB5/GNG2/GNG3/GNG5/KCNJ11/LCN12/LMF2/LRRC8B/MCOLN1/MICU3/NALCN/OS9/P4HB/PRKACB/PRKAR1B/PRKAR2B/PSMA2/PSMA3/PSMA6/PSMC2/PSME1/PSME2/PSME4/RYR1/SEM1/SLC12A4/SLC12A5/SLC13A3/SLC14A1/SLC15A2/SLC17A7/SLC1A1/SLC1A2/SLC1A3/SLC1A4/SLC24A4/SLC25A18/SLC25A22/SLC25A4/SLC26A6/SLC2A11/SLC2A12/SLC2A13/SLC30A5/SLC35C1/SLC35D2/SLC39A1/SLC39A7/SLC3A2/SLC43A2/SLC4A10/SLC4A2/SLC6A1/SLC6A11/SLC6A13/SLC6A15/SLC7A11/SLC8A2/SLC8A3/SLC9A6/SLC9A8/SLC9B2/SLCO1C1/TRPM3/TSC22D3/TUSC3/UBA52/UNC79/UNC80/WNK2/WWP1 | 119 | REACTOME |
| REACTOME_TRANSPORT_OF_THE_SLBP_DEPENDANT_MATURE_MRNA | 0.000557467 | 0.006501829 | AAAS/ALYREF/NCBP2/NDC1/NUP107/NUP188/NUP210/NUP42/NUP43/NUP88 | 10 | REACTOME |
| REACTOME_TRANSPORT_TO_THE_GOLGI_AND_SUBSEQUENT_MODIFICATION | 0.000109486 | 0.003107886 | ANK1/ANK2/ANK3/ARF4/ARFGAP1/B4GALT4/B4GALT6/CNIH2/CNIH3/COG1/COG7/DCTN1/DYNC1I1/DYNLL2/GRIA1/KDELR2/LMAN2/MGAT3/MGAT4B/MGAT4C/NAPB/NSF/SEC16A/SEC16B/SEC24D/SPTB/SPTBN1/SPTBN2/SPTBN4/ST8SIA3/TMED2/TMED3/TMED9/TMEM115/TRAPPC6B/TUBA4A/TUBA8/TUBB2A/TUBB6/YKT6 | 40 | REACTOME |
| REACTOME_TRNA_PROCESSING | 3.95228E-05 | 0.000587802 | AAAS/ADAT1/CDKAL1/CPSF4/DUS2/HSD17B10/NDC1/NUP107/NUP188/NUP210/NUP42/NUP43/NUP88/PUS3/RAN/RPP40/TPRKB/TRDMT1/TRMT6/TRMT61A/TRMU/TSEN15/WDR4 | 23 | REACTOME |
| REACTOME_TRNA_PROCESSING_IN_THE_NUCLEUS | 0.003176622 | 0.030739712 | AAAS/CPSF4/NDC1/NUP107/NUP188/NUP210/NUP42/NUP43/NUP88/RAN/RPP40/TSEN15 | 12 | REACTOME |
| REACTOME_UNBLOCKING_OF_NMDA_RECEPTORS_GLUTAMATE_BINDING_AND_ACTIVATION | 6.7001E-08 | 4.63031E-06 | CALM1/CAMK2A/CAMK2B/CAMK2G/DLG2/DLG3/DLG4/GRIA1/GRIN1/GRIN2A/GRIN2C/LRRC7/NEFL | 13 | REACTOME |
| REACTOME_UNWINDING_OF_DNA | 1.15215E-13 | 6.07898E-12 | CDC45/GINS1/GINS2/GINS3/GINS4/MCM2/MCM3/MCM4/MCM5/MCM6/MCM7/MCM8 | 12 | REACTOME |
| REACTOME_UPTAKE_AND_ACTIONS_OF_BACTERIAL_TOXINS | 5.27724E-05 | 0.001750307 | ANTXR2/CALM1/CD9/MAP2K1/MAP2K4/SNAP25/STX1A/STX1B/SV2A/SYT1/VAMP1/VAMP2 | 12 | REACTOME |
| REACTOME_VASOPRESSIN_REGULATES_RENAL_WATER_HOMEOSTASIS_VIA_AQUAPORINS | 0.000986952 | 0.018693604 | ADCY1/ADCY2/ADCY5/AQP3/AQP4/GNB2/GNB5/GNG2/GNG3/GNG5/PRKACB/PRKAR1B/PRKAR2B | 13 | REACTOME |
| REACTOME_VESICLE_MEDIATED_TRANSPORT | 0.00203109 | 0.029677132 | ACTB/AMPH/ANK1/ANK2/ANK3/AP1S1/AP4M1/AP4S1/ARF4/ARFGAP1/ARPC1A/ARPC2/ARPC4/ARPC5/ARRB1/BLOC1S4/CALM1/CALR/CCZ1/CCZ1B/CLVS1/CLVS2/CNIH2/CNIH3/COG1/COG7/CYTH2/DCTN1/DNM1/DNM3/DYNC1I1/DYNLL2/EPS15/GABARAPL2/GALNT2/GGA1/GOLGA5/GRIA1/GRK3/HSP90B1/HYOU1/KDELR2/KIAA0319/KIF1A/KIF26A/KIF3A/KIF3B/KIF3C/KIF5A/KIF9/KIFAP3/KIFC2/KLC1/LMAN2/LRP1/MADD/MYO5A/NAPB/NECAP1/NSF/PACSIN1/PIP5K1C/PRKAG2/RAB13/RAB27B/RAB33A/RAB38/RAB3A/RAB6B/RABEP1/REPS2/SBF1/SCOC/SEC16A/SEC16B/SEC24D/SGIP1/SH3GL2/SNAP91/SNX5/SPTB/SPTBN1/SPTBN2/SPTBN4/STAM/STON2/STX4/SURF4/SYS1/SYT1/TBC1D1/TBC1D24/TMED2/TMED3/TMED9/TMEM115/TPD52L1/TRAPPC6B/TRIP10/TUBA4A/TUBA8/TUBB2A/TUBB6/TXNDC5/UBA52/ULK1/VAMP2/VPS53/YKT6/YWHAG/YWHAH | 111 | REACTOME |
| REACTOME_VIRAL_MESSENGER_RNA_SYNTHESIS | 0.000796493 | 0.008781238 | AAAS/GTF2F1/GTF2F2/NDC1/NUP107/NUP188/NUP210/NUP42/NUP43/NUP88/POLR2F | 11 | REACTOME |
| REACTOME_VOLTAGE_GATED_POTASSIUM_CHANNELS | 0.000271703 | 0.00668023 | KCNA1/KCNA3/KCNAB1/KCNAB2/KCNB1/KCNC1/KCNC2/KCNC3/KCNC4/KCNH1/KCNH3/KCNQ3/KCNQ5/KCNS3 | 14 | REACTOME |
| WP_ALZHEIMERS_DISEASE | 0.001706915 | 0.026776573 | APBB1/ATF4/BID/CACNA1C/CALM1/CALM3/CAPN2/CDK5R1/CHRNA7/ERN1/FZD5/GNAQ/GRIN1/GRIN2A/GRIN2C/ITPR1/KIF5A/KIF5C/KLC1/LRP1/MAP2K1/MAPK1/MAPK10/MAPT/PIK3CB/PIK3R1/PLCB1/PLCB4/PPP3CA/PPP3CB/PPP3R1/PSMA2/PSMA3/PSMA6/PSMC2/RELA/RTN3/RTN4/SEM1/SLC25A4/SNCA/TRAF2/TUBA4A/TUBA8/TUBB2A/TUBB6/ULK1/WNT7A | 48 | WP |
| WP_AMYOTROPHIC_LATERAL_SCLEROSIS_ALS | 0.000256959 | 0.006379069 | BAX/BID/DAXX/GRIA1/NEFH/NEFL/NEFM/PPP3CA/PPP3CB/SLC1A2/SOD1/TOMM40/TP53 | 13 | WP |
| WP_ATM_SIGNALING_IN_DEVELOPMENT_AND_DISEASE | 1.46746E-05 | 0.000237365 | AURKB/BUB1/CDK1/CHEK1/DCLRE1C/H2AX/LBR/MAP3K5/MDC1/PPM1D/PRKDC/RBBP8/RNF40/TRIM28 | 14 | WP |
| WP_ATM_SIGNALING_PATHWAY | 2.29042E-06 | 4.22964E-05 | ABL1/BRCA1/CASP2/CCNB1/CCNE1/CDC25A/CDC25C/CDK1/CDK2/CHEK1/FANCD2/H2AX/MDC1/RAD51 | 14 | WP |
| WP_BASE_EXCISION_REPAIR | 5.44551E-09 | 1.69961E-07 | APEX1/FEN1/HMGB1/LIG3/MUTYH/NEIL3/NTHL1/OGG1/PARP1/PCNA/POLD3/POLE/POLE2/UNG/XRCC1 | 15 | WP |
| WP_BIOMARKERS_FOR_PYRIMIDINE_METABOLISM_DISORDERS | 0.00088755 | 0.009688721 | DHODH/NT5C3A/RRM1/RRM2/TYMS/UMPS | 6 | WP |
| WP_BRAINDERIVED_NEUROTROPHIC_FACTOR_BDNF_SIGNALING_PATHWAY | 0.000120667 | 0.003247858 | CAMK2A/CAMK4/CDK5R1/CRTC1/DOCK3/DPYSL2/EIF2S2/EIF4EBP1/ELK1/FRS3/GABRB3/GRIA1/GRIN1/GRIP1/KCNA3/LINGO1/MAP2K1/MAPK1/MAPK10/MAPT/MEF2A/MEF2C/NSF/NTRK2/PIK3R1/PTK2B/RAB3A/RACK1/RELA/RPS6KA5/SH2B2/SHC1/SYN1 | 33 | WP |
| WP_BREAST_CANCER_PATHWAY | 0.005529484 | 0.048818071 | AKT1/AKT2/BRCA1/BRCA2/CCND1/CDK4/CDK6/DLL3/DVL2/E2F1/E2F2/E2F3/EGFR/HEY1/LRP5/LRP6/NOTCH1/NOTCH4/PARP1/PIK3R2/RAD51/RAF1/TCF7L1 | 23 | WP |
| WP_CALCIUM_REGULATION_IN_CARDIAC_CELLS | 4.59684E-12 | 1.67916E-09 | ADCY1/ADCY2/ADCY5/ADRA1A/ADRA1B/ADRB1/ARRB1/ATP1B1/ATP1B3/ATP2B1/ATP2B2/CACNA1A/CACNA1C/CACNB1/CACNB3/CALM1/CALM3/CALR/CAMK2A/CAMK2B/CAMK2G/CAMK4/CHRM1/CHRM3/CHRM4/GNAI1/GNAI2/GNAO1/GNAQ/GNAZ/GNB2/GNB5/GNG2/GNG3/GNG5/ITPR1/KCNB1/PRKACB/PRKAR1B/PRKAR2B/PRKCB/PRKCE/PRKCZ/RGS20/RGS4/RGS7/RYR1/SLC8A3/YWHAG/YWHAH | 50 | WP |
| WP_CELL_CYCLE | 6.2202E-22 | 8.61498E-20 | ABL1/ANAPC7/BUB1/CCNA2/CCNB1/CCNB2/CCND1/CCND2/CCNE1/CCNE2/CDC20/CDC25A/CDC25B/CDC25C/CDC45/CDC6/CDC7/CDK1/CDK2/CDK4/CDK6/CDKN2C/CHEK1/DBF4/E2F1/E2F2/E2F3/E2F5/ESPL1/HDAC2/MAD2L2/MCM2/MCM3/MCM4/MCM5/MCM6/MCM7/ORC1/ORC5/ORC6/PCNA/PKMYT1/PLK1/PRKDC/PTTG1/RBL1/SKP2/TTK/WEE1 | 49 | WP |
| WP_CELL_MIGRATION_AND_INVASION_THROUGH_P75NTR | 0.003289396 | 0.031692616 | AKT1/AKT2/EFNA2/EFNA5/EFNB1/MMP2/PARD3/TRIO | 8 | WP |
| WP_CELLTYPE_DEPENDENT_SELECTIVITY_OF_CCK2R_SIGNALING | 0.000282981 | 0.006891261 | CCK/DAGLA/GNAI1/GNAQ/ITPR1/PLCB1/RYR1 | 7 | WP |
| WP_COHESIN_COMPLEX_CORNELIA_DE_LANGE_SYNDROME | 0.001903475 | 0.01943825 | AURKB/CDCA5/CDK1/ESCO2/ESPL1/PLK1/PTTG1/SGO1/SGO2 | 9 | WP |
| WP_COMMON_PATHWAYS_UNDERLYING_DRUG_ADDICTION | 4.94875E-05 | 0.001704557 | ACTB/ADCY1/CALM1/CAMK2A/CAMK4/GNAI1/GRIA1/GRIN1/GRIN2A/GRM5/MAP2K1/MAPK1/PPP1R1A/PRKACB/PRKCB | 15 | WP |
| WP_CYTOPLASMIC_RIBOSOMAL_PROTEINS | 3.6795E-09 | 3.7634E-07 | RPL10/RPL11/RPL12/RPL13A/RPL17/RPL18/RPL18A/RPL23/RPL27A/RPL28/RPL29/RPL36/RPL36A/RPL39/RPLP0/RPLP1/RPS10/RPS11/RPS14/RPS16/RPS19/RPS2/RPS20/RPS23/RPS27/RPS28/RPS3A/RPS5/RPS7/RPS8/RPS9/UBA52 | 32 | WP |
| WP_CYTOSINE_METHYLATION | 0.00440283 | 0.040640443 | DNMT1/IDH1/IDH2/MBD3 | 4 | WP |
| WP_DISRUPTION_OF_POSTSYNAPTIC_SIGNALING_BY_CNV | 1.34682E-05 | 0.000555454 | CAMK2A/CAMK2B/CAMK2G/DLG2/DLGAP1/GRIN1/GRIN2A/GRIN2C/MAPK1/NRXN3/RPH3A/SHANK1/STX1A/YWHAG | 14 | WP |
| WP_DNA_DAMAGE_RESPONSE | 2.1829E-08 | 6.28221E-07 | ABL1/ATRIP/BRCA1/CASP3/CCNB1/CCNB2/CCND1/CCND2/CCNE1/CCNE2/CDC25A/CDC25C/CDK1/CDK2/CDK4/CDK6/CHEK1/E2F1/FANCD2/H2AX/PRKDC/RAD51 | 22 | WP |
| WP_DNA_IRDAMAGE_AND_CELLULAR_RESPONSE_VIA_ATR | 3.01088E-17 | 2.30073E-15 | ATRIP/BARD1/BRCA1/BRCA2/CDC25C/CDC45/CDK1/CDK2/CHEK1/CLSPN/E2F1/EXO1/FANCA/FANCD2/FANCI/FEN1/FOXM1/H2AX/MCM2/MDC1/MSH2/PARP1/PCNA/PLK1/PPM1D/PRKDC/RAD51/RBBP8/RFWD3/RMI1/RPA1/TDP1/TOP3A/TRIM28/USP1 | 35 | WP |
| WP_DNA_IRDOUBLE_STRAND_BREAKS_AND_CELLULAR_RESPONSE_VIA_ATM | 2.66621E-07 | 5.79247E-06 | ABL1/BLM/BRCA1/BRCA2/CASP3/CDC25C/CHEK1/DCLRE1C/E2F1/EXO1/FANCD2/H2AX/MDC1/PARP1/PCNA/PRKDC/RAD51/TRIM28 | 18 | WP |
| WP_DNA_MISMATCH_REPAIR | 4.91691E-09 | 1.55655E-07 | EXO1/MSH2/MSH6/PCNA/POLD3/POLE/POLE2/RFC2/RFC3/RFC4/RFC5/RPA1/RPA3 | 13 | WP |
| WP_DNA_REPAIR_PATHWAYS_FULL_NETWORK | 2.28254E-20 | 2.19918E-18 | APEX1/BRCA1/BRCA2/CENPX/CHEK1/CUL4A/DCLRE1C/ERCC8/EXO1/FAAP100/FAAP24/FANCA/FANCC/FANCD2/FANCE/FANCG/FANCI/FEN1/GTF2H2C/GTF2H4/H2AX/HMGB1/LIG3/MSH2/MSH6/MUTYH/NEIL3/NTHL1/OGG1/PARP1/PCNA/POLD3/POLE/POLE2/PRKDC/RAD51/RAD54B/REV3L/RFC2/RFC3/RFC4/RFC5/RPA1/RPA3/UNG/USP1/XRCC1 | 47 | WP |
| WP_DNA_REPLICATION | 5.10953E-25 | 1.13227E-22 | CDC45/CDC6/CDC7/CDK2/CDT1/DBF4/GMNN/MCM10/MCM2/MCM3/MCM4/MCM5/MCM6/MCM7/ORC1/ORC5/ORC6/PCNA/POLA1/POLA2/POLD3/POLE/POLE2/PRIM1/PRIM2/RFC2/RFC3/RFC4/RFC5/RPA1/RPA3 | 31 | WP |
| WP_ECTODERM_DIFFERENTIATION | 0.001291055 | 0.022612072 | ANKS1B/ARHGDIG/ARHGEF9/ARX/BAZ1A/BCAS3/BOC/CAP2/CDH8/CLVS1/CTNNA2/ELOVL4/FZD5/GRAMD1B/HESX1/JAKMIP1/LDB2/MYORG/NFATC1/PI4KA/PLCXD3/PRKAG2/PTPRB/RIT1/RRBP1/SNCA/SOCS2/SORCS1/TSKU/ZBTB16 | 30 | WP |
| WP_ENDOTHELIN_PATHWAYS | 0.001288985 | 0.022612072 | ADRA1A/ADRB1/CALM1/GNAI1/GNB5/MAP2K1/MAPK1/NPY/NPY1R/PLCB1/RIIAD1 | 11 | WP |
| WP_ERBB_SIGNALING_PATHWAY | 0.000726595 | 0.014514869 | CAMK2A/CAMK2B/CAMK2G/CBLB/EIF4EBP1/ELK1/ERBB4/MAP2K1/MAP2K4/MAPK1/MAPK10/MDM2/NRG3/PAK1/PAK3/PAK4/PAK6/PIK3CB/PIK3R1/PRKCB/SHC1/TP53 | 22 | WP |
| WP_FRAGILE_X_SYNDROME | 1.04447E-06 | 5.23669E-05 | AGAP2/AKAP5/CAMK2A/CAMK2B/CAMK4/CYFIP2/DLG4/DLGAP3/DNM1/EIF4A1/EPHA4/GABRB2/GABRD/GABRG2/GRIA1/GRIN1/GRIN2A/GRIP1/GRIP2/GRM5/ITPR1/KCNC1/MAP2K1/MAPK1/NTRK2/PIK3CB/PLCB1/PPP3CA/PTPN5/RAP1GAP/SHANK1/SHC1/SLC6A1/TARBP2 | 34 | WP |
| WP_G_PROTEIN_SIGNALING_PATHWAYS | 8.46331E-08 | 5.68032E-06 | ADCY1/ADCY2/ADCY5/AKAP11/AKAP13/AKAP5/AKAP6/ARHGEF1/CALM1/GNAI1/GNAI2/GNAL/GNAO1/GNAQ/GNAZ/GNB2/GNB5/GNG3/GNG5/ITPR1/PDE1A/PDE1B/PDE4A/PDE8B/PPP3CA/PRKACB/PRKAR1B/PRKAR2B/PRKCB/PRKCE/PRKCZ | 31 | WP |
| WP_G1_TO_S_CELL_CYCLE_CONTROL | 1.66941E-21 | 2.05523E-19 | CCNB1/CCND1/CCND2/CCNE1/CCNE2/CDC25A/CDC45/CDK1/CDK2/CDK4/CDK6/CDKN2C/CREB3L1/CREB3L4/E2F1/E2F2/E2F3/MCM2/MCM3/MCM4/MCM5/MCM6/MCM7/ORC1/ORC5/ORC6/PCNA/POLA2/POLE/POLE2/PRIM1/PRIM2/RPA1/RPA3/WEE1 | 35 | WP |
| WP_GABA_RECEPTOR_SIGNALING | 4.79535E-05 | 0.001703014 | GABBR1/GABBR2/GABRA2/GABRA3/GABRA5/GABRB1/GABRB2/GABRB3/GABRD/GABRG2/GAD2/SLC6A1/SLC6A11 | 13 | WP |
| WP_GASTRIC_CANCER_NETWORK_1 | 1.6509E-09 | 5.90064E-08 | AURKA/CENPF/E2F7/ECT2/KIF15/LIN9/MCM4/MYBL2/NOTCH1/NUP107/RNF216/RUVBL1/TOP2A/TPX2/UBE2C | 15 | WP |
| WP_GASTRIC_CANCER_NETWORK_2 | 7.71439E-06 | 0.000131501 | ATAD2/CHTF18/DSCC1/EGFR/FANCI/LBR/LMNB2/RFC3/RFC4/TOP2A/UBE2C/UBE2T | 12 | WP |
| WP_GLIAL_CELL_DIFFERENTIATION | 3.4056E-07 | 0.000394027 | CNP/MAG/MBP/PLP1/TPPP | 5 | WP |
| WP_GLIOBLASTOMA_SIGNALING_PATHWAYS | 0.000448719 | 0.005317442 | AKT1/AKT2/BRCA1/BRCA2/CCND1/CCND2/CCNE1/CDK2/CDK4/CDK6/CDKN2C/E2F1/EGFR/MSH6/PIK3R2/RAF1/SRC | 17 | WP |
| WP_GLYCOSYLATION_AND_RELATED_CONGENITAL_DEFECTS | 0.001516116 | 0.024850701 | ALG2/ALG3/ALG9/DPM1/DPM3/GMPPB/MOGS/PMM2/TUSC3 | 9 | WP |
| WP_GPCRS_CLASS_C_METABOTROPIC_GLUTAMATE_PHEROMONE | 0.001368297 | 0.022867547 | GABBR1/GABBR2/GPRC5B/GRM2/GRM3/GRM5/GRM8 | 7 | WP |
| WP_H19_ACTION_RBE2F1_SIGNALING_AND_CDKBETACATENIN_ACTIVITY | 1.6712E-05 | 0.000268361 | CCND1/CDK4/E2F1/H19/MACROH2A1/MED1/SOX4/TULP3 | 8 | WP |
| WP_HOMOLOGOUS_RECOMBINATION | 0.002991489 | 0.029203263 | BRCA2/POLD3/RAD51/RAD54B/RPA1 | 5 | WP |
| WP_INTEGRATED_CANCER_PATHWAY | 5.14744E-08 | 1.32636E-06 | AKT1/BARD1/BLM/BRCA1/CASP3/CDC25A/CDC25B/CDK1/CDK2/CDK4/CHEK1/E2F1/MAP3K5/MSH2/MSH6/NOXA1/PLK1 | 17 | WP |
| WP_MAPK_SIGNALING_PATHWAY | 2.58026E-05 | 0.001015034 | ARRB1/ATF4/CACNA1A/CACNA1C/CACNA1G/CACNA2D1/CACNA2D2/CACNA2D3/CACNB1/CACNB2/CACNB3/CACNB4/CACNG8/DAXX/DUSP4/DUSP6/DUSP8/DUSP9/ELK1/FGF12/FGF13/FGF17/FGF22/FGF9/FGFR3/MAP2K1/MAP2K4/MAP3K14/MAPK1/MAPK10/MAPK8IP1/MAPK8IP2/MAPT/MEF2C/MRAS/NFATC1/NTRK2/PAK1/PPP3CA/PPP3CB/PPP3R1/PRKACB/PTPN5/PTPRR/RAPGEF2/RASGRF2/RASGRP1/RELA/RPS6KA5/STMN1/TP53/TRAF2 | 52 | WP |
| WP_MBDNF_AND_PROBDNF_REGULATION_OF_GABA_NEUROTRANSMISSION | 0.000455453 | 0.0101269 | GABRA2/GABRA3/GABRA5/GABRB1/GABRB2/GABRB3/GABRD/GABRG2/NTRK2/PIK3CB/PIK3R1/SHC1/SLC12A5 | 13 | WP |
| WP_MELANOMA | 0.001905074 | 0.028321368 | BAK1/BAX/CALM1/CALM3/ELK1/ERBB4/GRIN2A/GRM3/KIT/MAP2K1/MAPK1/MDM2/PAK1/PIK3CB/PIK3R1/STK19/TP53 | 17 | WP |
| WP_MIRNA_REGULATION_OF_DNA_DAMAGE_RESPONSE | 1.73605E-06 | 3.26024E-05 | ABL1/ATRIP/BRCA1/CASP3/CCNB1/CCNB2/CCND1/CCND2/CCNE1/CCNE2/CDC25A/CDC25C/CDK1/CDK2/CDK4/CDK6/CHEK1/E2F1/FANCD2/H2AX/MCM7/PRKDC/RAD51 | 23 | WP |
| WP_MRNA_PROCESSING | 2.46747E-09 | 8.54361E-08 | CD2BP2/CPSF3/CPSF4/CSTF3/DDX20/DHX8/EFTUD2/FUS/HNRNPA1/HNRNPA2B1/HNRNPAB/HNRNPD/HNRNPH1/HNRNPR/LSM2/LSM7/METTL3/NCBP2/NONO/PHF5A/PRMT1/PRPF4/PRPF6/PSKH1/RBMX/SF3A2/SNRPA/SNRPD3/SNRPE/SRSF10/SRSF3/TRA2B/U2AF2/YBX1 | 34 | WP |
| WP_MYOMETRIAL_RELAXATION_AND_CONTRACTION_PATHWAYS | 2.92606E-05 | 0.001084338 | ACKR3/ACTB/ACTC1/ADCY1/ADCY2/ADCY5/ARRB1/ATF4/CACNB3/CALD1/CALM1/CALM3/CAMK2A/CAMK2B/CAMK2G/CRHR1/GNAQ/GNB2/GNB5/GNG2/GNG3/GNG5/GUCY1A1/IGFBP2/ITPR1/PRKACB/PRKAR1B/PRKAR2B/PRKCB/PRKCE/PRKCZ/RGS20/RGS4/RGS7/RYR1/YWHAG/YWHAH | 37 | WP |
| WP_NEUROINFLAMMATION_AND_GLUTAMATERGIC_SIGNALING | 0.000120667 | 0.003247858 | ADCY1/CALM1/CAMK2A/CAMK2B/CAMK2G/CAMK4/CAMKK1/CAMKK2/GFAP/GLS2/GOT1/GRIA1/GRIK2/GRIN1/GRIN2A/GRIN2C/GRM2/GRM5/GRM8/LRRC8B/MAPK1/NSMF/PLCB1/PLCB4/PRKCB/SHMT2/SLC17A7/SLC1A1/SLC1A2/SLC1A3/SLC1A4/TGFBR3/TRAF5 | 33 | WP |
| WP_NOCGMPPKG_MEDIATED_NEUROPROTECTION | 0.002968175 | 0.039736249 | CALM1/CAMK2A/CAMK2B/CAMK2G/DLG4/GRIN1/GRIN2A/GRIN2C/GUCY1A1/GUCY1B1/NEFL/PDE2A/RELA | 13 | WP |
| WP_NUCLEOTIDE_EXCISION_REPAIR | 8.24682E-06 | 0.000138447 | CUL4A/ERCC8/GTF2H2C/GTF2H4/PCNA/POLD3/POLE/POLE2/RFC2/RFC3/RFC4/RFC5/RPA1/RPA3 | 14 | WP |
| WP_NUCLEOTIDE_EXCISION_REPAIR_IN_XERODERMA_PIGMENTOSUM | 1.14958E-05 | 0.000188701 | BRCA1/CUL4A/ERCC8/GTF2H2C/GTF2H4/LIG3/PARP1/PCNA/POLD3/POLE/POLE2/RAD18/RFC2/RFC3/RFC4/RFC5/RPA1/RPA3/XRCC1 | 19 | WP |
| WP_OLIGODENDROCYTE_SPECIFICATION_AND_DIFFERENTIATION_LEADING_TO_MYELIN_COMPONENTS_FOR_CNS | 5.43168E-06 | 0.003142229 | CNP/CNTF/MAG/MBP/MOG/PLP1/SOX10 | 7 | WP |
| WP_PHOSPHODIESTERASES_IN_NEURONAL_FUNCTION | 2.64254E-05 | 0.001023785 | ADCY1/ADCY2/ADCY5/CHRNA7/GRIA1/GRIN1/GRIN2A/GRIN2C/GUCY1A1/GUCY1B1/PDE10A/PDE1A/PDE1B/PDE2A/PDE3B/PDE4A/PDE8B/PPP1R1B | 18 | WP |
| WP_PKCGAMMA_CALCIUM_SIGNALING_PATHWAY_IN_ATAXIA | 0.002571059 | 0.035156134 | ATP2B2/CA8/CACNA1A/GNAQ/GRIA1/ITPR1/PLCB1/PLCB4 | 8 | WP |
| WP_PREIMPLANTATION_EMBRYO | 0.003674436 | 0.035249139 | E2F5/ELAVL1/FOSB/FOXD1/HNRNPAB/KHSRP/KLF4/MYBL1/SMARCA4/SOX11/SOX2/TCF7L1 | 12 | WP |
| WP_PYRIMIDINE_METABOLISM | 3.88713E-07 | 8.05036E-06 | CAD/CTPS1/CTPS2/DCTPP1/DHODH/DTYMK/NME1/NME1-NME2/POLA1/POLA2/POLD3/POLE/POLE2/POLR1C/POLR1E/POLR3D/PRIM1/PRIM2/RRM1/RRM2/TK1/TYMS/UMPS | 23 | WP |
| WP_PYRIMIDINE_METABOLISM_AND_RELATED_DISEASES | 0.000263211 | 0.003258529 | CAD/DHODH/NT5C3A/RRM1/RRM2/TYMS/UMPS | 7 | WP |
| WP_REGULATION_OF_SISTER_CHROMATID_SEPARATION_AT_THE_METAPHASEANAPHASE_TRANSITION | 0.000166873 | 0.002201135 | BUB1/BUB1B/CDC20/CENPE/ESPL1/MAD2L1/PTTG1 | 7 | WP |
| WP_RETINOBLASTOMA_GENE_IN_CANCER | 2.47326E-41 | 1.09615E-38 | ABL1/BARD1/CCNA2/CCNB1/CCNB2/CCND1/CCNE1/CCNE2/CDC25A/CDC25B/CDC45/CDC7/CDK1/CDK2/CDK4/CDK6/CDT1/CHEK1/DHFR/DNMT1/E2F1/E2F2/E2F3/FAF1/FANCG/H2AZ1/HMGB1/HMGB2/KIF4A/MCM3/MCM4/MCM6/MCM7/MSH6/ORC1/PCNA/PLK4/POLA1/POLD3/POLE/POLE2/PRIM1/PRKDC/RAF1/RFC3/RFC4/RFC5/RPA1/RPA3/RRM1/RRM2/SKP2/SMC2/SUV39H1/TOP2A/TTK/TYMS/WEE1 | 58 | WP |
| WP_RETT_SYNDROME_CAUSING_GENES | 0.000355022 | 0.00832837 | ACTL6B/GABBR2/GABRA3/GABRD/GNAO1/GRIN2A/HDAC1/HIVEP2/MEF2C/SCN2A/SCN8A/SHANK3/SMARCA2/SRRM3/STXBP1 | 15 | WP |
| WP_SMALL_CELL_LUNG_CANCER | 0.00120022 | 0.012911102 | AKT1/AKT2/CASP3/CCND1/CCNE1/CCNE2/CDK2/CDK4/CDK6/CKS1B/CKS2/COL4A5/E2F1/E2F2/E2F3/LAMA1/PIK3R2/TRAF4 | 18 | WP |
| WP_SPLICING_FACTOR_NOVA_REGULATED_SYNAPTIC_PROTEINS | 1.08178E-05 | 0.000453462 | ANK3/ATP2B1/CADM3/CAMK2G/CLSTN1/EPB41L1/GABBR2/GABRG2/GRIK2/GRIN1/KCNMA1/MAP4/NCDN/PLCB4/PRKCZ/RAP1GAP | 16 | WP |
| WP_SUDDEN_INFANT_DEATH_SYNDROME_SIDS_SUSCEPTIBILITY_PATHWAYS | 0.000118486 | 0.003247858 | AQP4/ATP1A3/CHRNA7/CHRNB2/DEAF1/GRIN1/HDAC1/HIF1A/HSP90B1/MAOA/MEF2C/NEUROD1/NOS1AP/NTRK2/PAH/PPARGC1A/PRKACB/PRKAR1B/PRKAR2B/REST/SCN3B/SCN4B/SLC1A3/SLC25A4/SNAP25/SNTA1/SPTBN1/SST/SSTR2/TAC1/THRB/TSPYL1/VAMP2/VIPR1/YWHAG/YWHAH | 36 | WP |
| WP_SYNAPTIC_SIGNALING_PATHWAYS_ASSOCIATED_WITH_AUTISM_SPECTRUM_DISORDER | 0.001810638 | 0.027723357 | CACNA1C/CAMK2B/CAMK4/DLG4/EIF4EBP1/GRIN1/GRIN2A/GRIN2C/MAPK1/NTRK2/PIK3CB/PIK3R1/PRKAG2/SHANK3 | 14 | WP |
| WP_SYNAPTIC_VESICLE_PATHWAY | 4.51325E-09 | 4.13903E-07 | ATP1A2/CACNA1A/CPLX1/CPLX2/DNM1/DNM3/NSF/RAB3A/RIMS1/SLC17A7/SLC1A3/SLC25A4/SNAP25/STX1A/STX1B/STXBP1/SYN1/SYN2/SYN3/SYP/SYT1/UNC13A/VAMP2 | 23 | WP |
| WP_TUMOR_SUPPRESSOR_ACTIVITY_OF_SMARCB1 | 0.00498372 | 0.044561616 | CDK4/CDK6/DPF2/EZH2/SMARCA4/SMARCB1/SMARCC1/SMARCD1 | 8 | WP |
| WP_VEGFAVEGFR2_SIGNALING_PATHWAY | 0.000534694 | 0.011277061 | ACKR3/ADAMTS9/AMOT/ARF4/ATF4/ATP6V1E1/C15orf39/CACNA2D1/CALR/CALU/CAMKK2/CAPN2/CGNL1/CSRP2/DPM1/ELK1/EPS15/ERN1/FJX1/FLII/GLUD1/HDAC1/HDAC7/HYOU1/INPP4B/ITGB5/JAG1/KCNC3/LDB2/LRRC59/MAP2K1/MAP2K4/MAPK1/MDM2/MEF2C/MICAL2/MIR1915HG/MMP14/NFATC1/P4HB/PAK1/PDE4DIP/PDIA6/PFN1/PIK3R1/PLOD3/PPP3CA/PRKCB/PRKCE/PRKCZ/PRKD2/PTK2B/RAB37/RACK1/RBM39/RCAN1/RCAN2/RCN1/RELA/RHOC/RND1/RPL13A/RPL18A/RPS11/RPS6KA5/S1PR1/SDF2L1/SHC1/SMARCA2/SSR4/STAM/TBCA/TMSB10/TXNDC5/WASF1 | 75 | WP |
| WP_VITAMIN_DSENSITIVE_CALCIUM_SIGNALING_IN_DEPRESSION | 0.000455453 | 0.0101269 | ATP2B1/ATP2B2/CACNA1C/CALB1/CHRM1/GRIN1/GRIN2A/GRIN2C/GRM5/ITPR1/KCNQ3/NFE2L2/PVALB | 13 | WP |

**Table S6.** Classification of intersective pathways between GESA and WGCNA approaches

| **Pathway Category** | **Intersective Pathway** |
| --- | --- |
| DNA damage response | BIOCARTA_ATRBRCA_PATHWAY |
| DNA damage response | BIOCARTA_P53_PATHWAY |
| DNA damage response | HALLMARK_DNA_REPAIR |
| DNA damage response | KEGG_HOMOLOGOUS_RECOMBINATION |
| DNA damage response | KEGG_MISMATCH_REPAIR |
| DNA damage response | KEGG_NUCLEOTIDE_EXCISION_REPAIR |
| DNA damage response | KEGG_P53_SIGNALING_PATHWAY |
| DNA damage response | PID_ATM_PATHWAY |
| DNA damage response | PID_ATR_PATHWAY |
| DNA damage response | PID_BARD1_PATHWAY |
| DNA damage response | PID_P73PATHWAY |
| DNA damage response | REACTOME_BASE_EXCISION_REPAIR |
| DNA damage response | REACTOME_DNA_DAMAGE_BYPASS |
| DNA damage response | REACTOME_DNA_DOUBLE_STRAND_BREAK_REPAIR |
| DNA damage response | REACTOME_DUAL_INCISION_IN_GG_NER |
| DNA damage response | REACTOME_DUAL_INCISION_IN_TC_NER |
| DNA damage response | REACTOME_GAP_FILLING_DNA_REPAIR_SYNTHESIS_AND_LIGATION_IN_GG_NER |
| DNA damage response | REACTOME_GLOBAL_GENOME_NUCLEOTIDE_EXCISION_REPAIR_GG_NER |
| DNA damage response | REACTOME_HDR_THROUGH_HOMOLOGOUS_RECOMBINATION_HRR |
| DNA damage response | REACTOME_HDR_THROUGH_SINGLE_STRAND_ANNEALING_SSA |
| DNA damage response | REACTOME_HOMOLOGOUS_DNA_PAIRING_AND_STRAND_EXCHANGE |
| DNA damage response | REACTOME_HOMOLOGY_DIRECTED_REPAIR |
| DNA damage response | REACTOME_NUCLEOTIDE_EXCISION_REPAIR |
| DNA damage response | REACTOME_PROCESSING_OF_DNA_DOUBLE_STRAND_BREAK_ENDS |
| DNA damage response | REACTOME_RECOGNITION_OF_DNA_DAMAGE_BY_PCNA_CONTAINING_REPLICATION_COMPLEX |
| DNA damage response | REACTOME_REGULATION_OF_TP53_ACTIVITY_THROUGH_PHOSPHORYLATION |
| DNA damage response | REACTOME_RESOLUTION_OF_D_LOOP_STRUCTURES |
| DNA damage response | REACTOME_SUMOYLATION_OF_DNA_DAMAGE_RESPONSE_AND_REPAIR_PROTEINS |
| DNA damage response | REACTOME_SUMOYLATION_OF_DNA_REPLICATION_PROTEINS |
| DNA damage response | REACTOME_TERMINATION_OF_TRANSLESION_DNA_SYNTHESIS |
| DNA damage response | REACTOME_TP53_REGULATES_TRANSCRIPTION_OF_CELL_CYCLE_GENES |
| DNA damage response | REACTOME_TP53_REGULATES_TRANSCRIPTION_OF_DNA_REPAIR_GENES |
| DNA damage response | REACTOME_TP53_REGULATES_TRANSCRIPTION_OF_GENES_INVOLVED_IN_G2_CELL_CYCLE_ARREST |
| DNA damage response | REACTOME_TRANSCRIPTION_COUPLED_NUCLEOTIDE_EXCISION_REPAIR_TC_NER |
| DNA damage response | REACTOME_TRANSLESION_SYNTHESIS_BY_POLK |
| DNA damage response | REACTOME_TRANSLESION_SYNTHESIS_BY_Y_FAMILY_DNA_POLYMERASES_BYPASSES_LESIONS_ON_DNA_TEMPLATE |
| DNA damage response | WP_ATM_SIGNALING_PATHWAY |
| DNA damage response | WP_DNA_DAMAGE_RESPONSE |
| DNA damage response | WP_DNA_IRDAMAGE_AND_CELLULAR_RESPONSE_VIA_ATR |
| DNA damage response | WP_DNA_IRDOUBLE_STRAND_BREAKS_AND_CELLULAR_RESPONSE_VIA_ATM |
| DNA damage response | WP_DNA_REPAIR_PATHWAYS_FULL_NETWORK |
| DNA damage response | WP_MIRNA_REGULATION_OF_DNA_DAMAGE_RESPONSE |
| DNA damage response | WP_MRNA_PROCESSING |
| DNA damage response | WP_NUCLEOTIDE_EXCISION_REPAIR |
| DNA damage response | WP_NUCLEOTIDE_EXCISION_REPAIR_IN_XERODERMA_PIGMENTOSUM |
| DNA damage response | REACTOME_TRANSLESION_SYNTHESIS_BY_POLH |
| DNA damage response | BIOCARTA_RB_PATHWAY |
| DNA damage response | REACTOME_REGULATION_OF_TP53_ACTIVITY |
| DNA damage response | REACTOME_FANCONI_ANEMIA_PATHWAY |
| Synapse | KEGG_CALCIUM_SIGNALING_PATHWAY |
| Synapse | REACTOME_NITRIC_OXIDE_STIMULATES_GUANYLATE_CYCLASE |
| Synapse | KEGG_LONG_TERM_DEPRESSION |
| Synapse | REACTOME_GABA_B_RECEPTOR_ACTIVATION |
| Synapse | REACTOME_GABA_RECEPTOR_ACTIVATION |
| Synapse | REACTOME_GLUTAMATE_NEUROTRANSMITTER_RELEASE_CYCLE |
| Synapse | REACTOME_ION_CHANNEL_TRANSPORT |
| Synapse | REACTOME_ION_HOMEOSTASIS |
| Synapse | REACTOME_ION_TRANSPORT_BY_P_TYPE_ATPASES |
| Synapse | REACTOME_MUSCLE_CONTRACTION |
| Synapse | REACTOME_NEUROTRANSMITTER_RELEASE_CYCLE |
| Synapse | REACTOME_NOREPINEPHRINE_NEUROTRANSMITTER_RELEASE_CYCLE |
| Synapse | REACTOME_POTASSIUM_CHANNELS |
| Synapse | REACTOME_VOLTAGE_GATED_POTASSIUM_CHANNELS |
| Synapse | WP_CELLTYPE_DEPENDENT_SELECTIVITY_OF_CCK2R_SIGNALING |
| Synapse | WP_GABA_RECEPTOR_SIGNALING |
| Synapse | WP_GPCRS_CLASS_C_METABOTROPIC_GLUTAMATE_PHEROMONE |
| Synapse | WP_MBDNF_AND_PROBDNF_REGULATION_OF_GABA_NEUROTRANSMISSION |
| Synapse | WP_PHOSPHODIESTERASES_IN_NEURONAL_FUNCTION |
| Synapse | WP_PKCGAMMA_CALCIUM_SIGNALING_PATHWAY_IN_ATAXIA |
| Proliferation | REACTOME_RHO_GTPASES_ACTIVATE_FORMINS |
| Proliferation | PID_E2F_PATHWAY |
| Proliferation | KEGG_SPLICEOSOME |
| Proliferation | PID_FOXM1_PATHWAY |
| Proliferation | REACTOME_SNRNP_ASSEMBLY |
| Proliferation | WP_GASTRIC_CANCER_NETWORK_2 |
| Proliferation | KEGG_PHOSPHATIDYLINOSITOL_SIGNALING_SYSTEM |
| Proliferation | REACTOME_CGMP_EFFECTS |
| Proliferation | BIOCARTA_EFP_PATHWAY |
| Proliferation | BIOCARTA_G2_PATHWAY |
| Proliferation | BIOCARTA_PTC1_PATHWAY |
| Proliferation | BIOCARTA_RANMS_PATHWAY |
| Proliferation | HALLMARK_MYC_TARGETS_V2 |
| Proliferation | KEGG_CELL_CYCLE |
| Proliferation | KEGG_PYRIMIDINE_METABOLISM |
| Proliferation | PID_MYC_ACTIV_PATHWAY |
| Proliferation | REACTOME_APC_C_MEDIATED_DEGRADATION_OF_CELL_CYCLE_PROTEINS |
| Proliferation | REACTOME_APC_CDC20_MEDIATED_DEGRADATION_OF_NEK2A |
| Proliferation | REACTOME_CHROMOSOME_MAINTENANCE |
| Proliferation | REACTOME_CYCLIN_A_B1_B2_ASSOCIATED_EVENTS_DURING_G2_M_TRANSITION |
| Proliferation | REACTOME_CYCLIN_A_CDK2_ASSOCIATED_EVENTS_AT_S_PHASE_ENTRY |
| Proliferation | REACTOME_DEPOSITION_OF_NEW_CENPA_CONTAINING_NUCLEOSOMES_AT_THE_CENTROMERE |
| Proliferation | REACTOME_DISEASES_OF_MITOTIC_CELL_CYCLE |
| Proliferation | REACTOME_DNA_REPLICATION |
| Proliferation | REACTOME_DNA_REPLICATION_PRE_INITIATION |
| Proliferation | REACTOME_EXTENSION_OF_TELOMERES |
| Proliferation | REACTOME_G2_M_CHECKPOINTS |
| Proliferation | REACTOME_G2_M_DNA_DAMAGE_CHECKPOINT |
| Proliferation | REACTOME_INITIATION_OF_NUCLEAR_ENVELOPE_NE_REFORMATION |
| Proliferation | REACTOME_MEIOSIS |
| Proliferation | REACTOME_MEIOTIC_RECOMBINATION |
| Proliferation | REACTOME_MITOTIC_G1_PHASE_AND_G1_S_TRANSITION |
| Proliferation | REACTOME_MITOTIC_PROPHASE |
| Proliferation | REACTOME_MITOTIC_SPINDLE_CHECKPOINT |
| Proliferation | REACTOME_NUCLEAR_PORE_COMPLEX_NPC_DISASSEMBLY |
| Proliferation | REACTOME_ORC1_REMOVAL_FROM_CHROMATIN |
| Proliferation | REACTOME_PHOSPHORYLATION_OF_THE_APC_C |
| Proliferation | REACTOME_S_PHASE |
| Proliferation | REACTOME_SUMOYLATION_OF_RNA_BINDING_PROTEINS |
| Proliferation | REACTOME_SWITCHING_OF_ORIGINS_TO_A_POST_REPLICATIVE_STATE |
| Proliferation | REACTOME_SYNTHESIS_OF_DNA |
| Proliferation | REACTOME_TELOMERE_MAINTENANCE |
| Proliferation | REACTOME_TRANSCRIPTION_OF_E2F_TARGETS_UNDER_NEGATIVE_CONTROL_BY_DREAM_COMPLEX |
| Proliferation | WP_CELL_CYCLE |
| Proliferation | WP_H19_ACTION_RBE2F1_SIGNALING_AND_CDKBETACATENIN_ACTIVITY |
| Proliferation | WP_PYRIMIDINE_METABOLISM |
| Proliferation | REACTOME_TELOMERE_EXTENSION_BY_TELOMERASE |
| Proliferation | REACTOME_SEPARATION_OF_SISTER_CHROMATIDS |
| Proliferation | REACTOME_CYCLIN_D_ASSOCIATED_EVENTS_IN_G1 |
| Proliferation | REACTOME_NUCLEOTIDE_BIOSYNTHESIS |
| Proliferation | PID_AURORA_A_PATHWAY |
| Proliferation | PID_RB_1PATHWAY |
| Proliferation | REACTOME_ADRENOCEPTORS |
| Complex cellular functions | REACTOME_RRNA_MODIFICATION_IN_THE_NUCLEUS_AND_CYTOSOL |
| Complex cellular functions | REACTOME_TRANSPORT_OF_MATURE_MRNAS_DERIVED_FROM_INTRONLESS_TRANSCRIPTS |
| Complex cellular functions | REACTOME_TRNA_PROCESSING |
| Complex cellular functions | REACTOME_TRNA_PROCESSING_IN_THE_NUCLEUS |
| Complex cellular functions | REACTOME_TRANSPORT_OF_THE_SLBP_DEPENDANT_MATURE_MRNA |
| Complex cellular functions | REACTOME_EXPORT_OF_VIRAL_RIBONUCLEOPROTEINS_FROM_NUCLEUS |
| Complex cellular functions | REACTOME_HIV_LIFE_CYCLE |
| Complex cellular functions | REACTOME_VIRAL_MESSENGER_RNA_SYNTHESIS |
| Complex cellular functions | REACTOME_TRANSPORT_OF_MATURE_TRANSCRIPT_TO_CYTOPLASM |
| Complex cellular functions | REACTOME_REGULATION_OF_GLUCOKINASE_BY_GLUCOKINASE_REGULATORY_PROTEIN |
| Complex cellular functions | REACTOME_SUMOYLATION_OF_SUMOYLATION_PROTEINS |
| Complex cellular functions | REACTOME_SUMOYLATION_OF_UBIQUITINYLATION_PROTEINS |
| Complex cellular functions | HALLMARK_UNFOLDED_PROTEIN_RESPONSE |
| Complex cellular functions | REACTOME_NUCLEAR_IMPORT_OF_REV_PROTEIN |
| Complex cellular functions | REACTOME_INTERACTIONS_OF_REV_WITH_HOST_CELLULAR_PROTEINS |
| Complex cellular functions | REACTOME_RNA_POLYMERASE_II_TRANSCRIPTION_TERMINATION |
| Complex cellular functions | REACTOME_MRNA_SPLICING_MINOR_PATHWAY |
| Complex cellular functions | BIOCARTA_FLUMAZENIL_PATHWAY |
| Complex cellular functions | REACTOME_CARDIAC_CONDUCTION |
| Complex cellular functions | KEGG_VASCULAR_SMOOTH_MUSCLE_CONTRACTION |

**Table S7.** The number of relevant pathway species corresponding to each prognostic radiomic feature

|  | **Complex cellular functions** | **DNA damage response** | **Proliferation** | **Synapse** | **C-index (95%CI)** |
| --- | --- | --- | --- | --- | --- |
| **RF1** | 8 | 42 | 44 | 1 | 0.6024 (0.5417, 0.6631) |
| **RF2** | 20 | 49 | 53 | 20 | 0.3564 (0.2955, 0.4173) |
| **RF3** | 19 | 46 | 52 | 8 | 0.6278 (0.5735, 0.6821) |
| **RF4** | 0 | 0 | 0 | 0 | 0.4419 (0.3714, 0.5124) |
| **RF5** | 1 | 1 | 3 | 0 | 0.6073 (0.5428, 0.6718) |
| **RF6** | 0 | 0 | 0 | 0 | 0.5572 (0.4918, 0.6227) |
| **RF7** | 2 | 1 | 1 | 9 | 0.6307 (0.5700, 0.6914) |
| **RF8** | 12 | 16 | 15 | 0 | 0.3850 (0.3231, 0.4468) |
| **RF9** | 18 | 27 | 20 | 17 | 0.3812 (0.3179, 0.4444) |
| **RF10** | 0 | 0 | 0 | 0 | 0.4440 (0.3800, 0.5081) |
| **RF11** | 12 | 16 | 15 | 0 | 0.3840 (0.3222, 0.4458) |
| **RF12** | 2 | 12 | 22 | 2 | 0.6101 (0.5474, 0.6728) |
| **RF13** | 20 | 45 | 51 | 20 | 0.6422 (0.5794, 0.7050) |
| **RF14** | 9 | 9 | 17 | 14 | 0.3898 (0.3265, 0.4530) |

**Table S8.** Exact data points of Figure 3C

| **Pathway** | **p.adjust** | **-log10(p.adjust)** | **Database** |
| --- | --- | --- | --- |
| KEGG_SPLICEOSOME | 8.35E-08 | 7.078313525 | KEGG |
| KEGG_PHOSPHATIDYLINOSITOL_SIGNALING_SYSTEM | 0.000153 | 3.815308569 | KEGG |
| KEGG_HOMOLOGOUS_RECOMBINATION | 0.000537 | 3.270025714 | KEGG |
| KEGG_RNA_DEGRADATION | 0.002936 | 2.532243949 | KEGG |
| REACTOME_CELLULAR_SENESCENCE | 1.53E-07 | 6.815308569 | REACTOME |
| REACTOME_BILE_ACID_AND_BILE_SALT_METABOLISM | 0.001732 | 2.761452112 | REACTOME |
| REACTOME_SYNTHESIS_OF_BILE_ACIDS_AND_BILE_SALTS_VIA_27_HYDROXYCHOLESTEROL | 0.028579 | 1.543952972 | REACTOME |
| REACTOME_SYNTHESIS_OF_BILE_ACIDS_AND_BILE_SALTS_VIA_7ALPHA_HYDROXYCHOLESTEROL | 0.041275 | 1.384312918 | REACTOME |
| PID_FOXM1_PATHWAY | 1.58E-06 | 5.801342913 | PID |
| PID_MYC_ACTIV_PATHWAY | 3.84E-06 | 5.415668776 | PID |
| PID_ATR_PATHWAY | 9.26E-06 | 5.033389013 | PID |
| PID_AURORA_A_PATHWAY | 0.00606 | 2.217527376 | PID |
| HALLMARK_UNFOLDED_PROTEIN_RESPONSE | 3.01E-06 | 5.521433504 | HALLMARK |
| HALLMARK_KRAS_SIGNALING_DN | 8.16E-06 | 5.088309841 | HALLMARK |
| HALLMARK_DNA_REPAIR | 8.66E-06 | 5.062482108 | HALLMARK |
| HALLMARK_MYC_TARGETS_V2 | 7.95E-05 | 4.099632871 | HALLMARK |
| WP_ATM_SIGNALING_PATHWAY | 3.88E-06 | 5.411168274 | WP |
| WP_METAPATHWAY_BIOTRANSFORMATION_PHASE_I_AND_II | 0.000306 | 3.514278574 | WP |
| WP_NUCLEOTIDE_EXCISION_REPAIR_IN_XERODERMA_PIGMENTOSUM | 0.000339 | 3.469800302 | WP |
| WP_H19_ACTION_RBE2F1_SIGNALING_AND_CDKBETACATENIN_ACTIVITY | 0.008505 | 2.070325682 | WP |
| BIOCARTA_FLUMAZENIL_PATHWAY | 0.000613 | 3.212539525 | BIOCARTA |
| BIOCARTA_PTC1_PATHWAY | 0.001042 | 2.982132281 | BIOCARTA |
| BIOCARTA_EFP_PATHWAY | 0.004917 | 2.308299792 | BIOCARTA |
| BIOCARTA_G2_PATHWAY | 0.03252 | 1.487849463 | BIOCARTA |

**Table S9.** Exact data points of Figure 4E

| **Pathway** | **p.adjust** | **-log10(p.adjust)** | **Database** |
| --- | --- | --- | --- |
| HALLMARK_E2F_TARGETS | 2.32E-82 | 81.63460719 | HALLMARK |
| HALLMARK_G2M_CHECKPOINT | 3.73E-64 | 63.42855957 | HALLMARK |
| HALLMARK_MYC_TARGETS_V1 | 4.14E-17 | 16.38282507 | HALLMARK |
| HALLMARK_MITOTIC_SPINDLE | 1.59E-10 | 9.798008758 | HALLMARK |
| REACTOME_CELL_CYCLE | 7.86E-73 | 72.10458258 | REACTOME |
| REACTOME_CELL_CYCLE_MITOTIC | 7.50E-63 | 62.12513282 | REACTOME |
| REACTOME_CELL_CYCLE_CHECKPOINTS | 4.77E-34 | 33.32171291 | REACTOME |
| REACTOME_NEURONAL_SYSTEM | 4.01E-30 | 29.39701214 | REACTOME |
| WP_RETINOBLASTOMA_GENE_IN_CANCER | 1.10E-38 | 37.9601306 | WP |
| WP_DNA_REPLICATION | 1.13E-22 | 21.94604897 | WP |
| WP_CELL_CYCLE | 8.61E-20 | 19.06474559 | WP |
| WP_G1_TO_S_CELL_CYCLE_CONTROL | 2.06E-19 | 18.68714045 | WP |
| KEGG_CELL_CYCLE | 6.97E-21 | 20.15697083 | KEGG |
| KEGG_DNA_REPLICATION | 4.88E-16 | 15.31134778 | KEGG |
| KEGG_RIBOSOME | 6.04E-08 | 7.219221433 | KEGG |
| KEGG_SPLICEOSOME | 1.07E-06 | 5.971602808 | KEGG |
| PID_PLK1_PATHWAY | 5.97E-15 | 14.22382764 | PID |
| PID_E2F_PATHWAY | 2.20E-13 | 12.65708264 | PID |
| PID_ATR_PATHWAY | 1.32E-12 | 11.87996806 | PID |
| PID_FANCONI_PATHWAY | 1.63E-12 | 11.78700932 | PID |
| BIOCARTA_MCM_PATHWAY | 2.21E-09 | 8.65495726 | BIOCARTA |
| BIOCARTA_CELLCYCLE_PATHWAY | 1.65E-06 | 5.781739015 | BIOCARTA |
| BIOCARTA_BARD1_PATHWAY | 4.73E-06 | 5.325556593 | BIOCARTA |
| BIOCARTA_G2_PATHWAY | 2.39E-05 | 4.622321434 | BIOCARTA |

**Figure S1.** The criteria for patients’ inclusion and exclusion


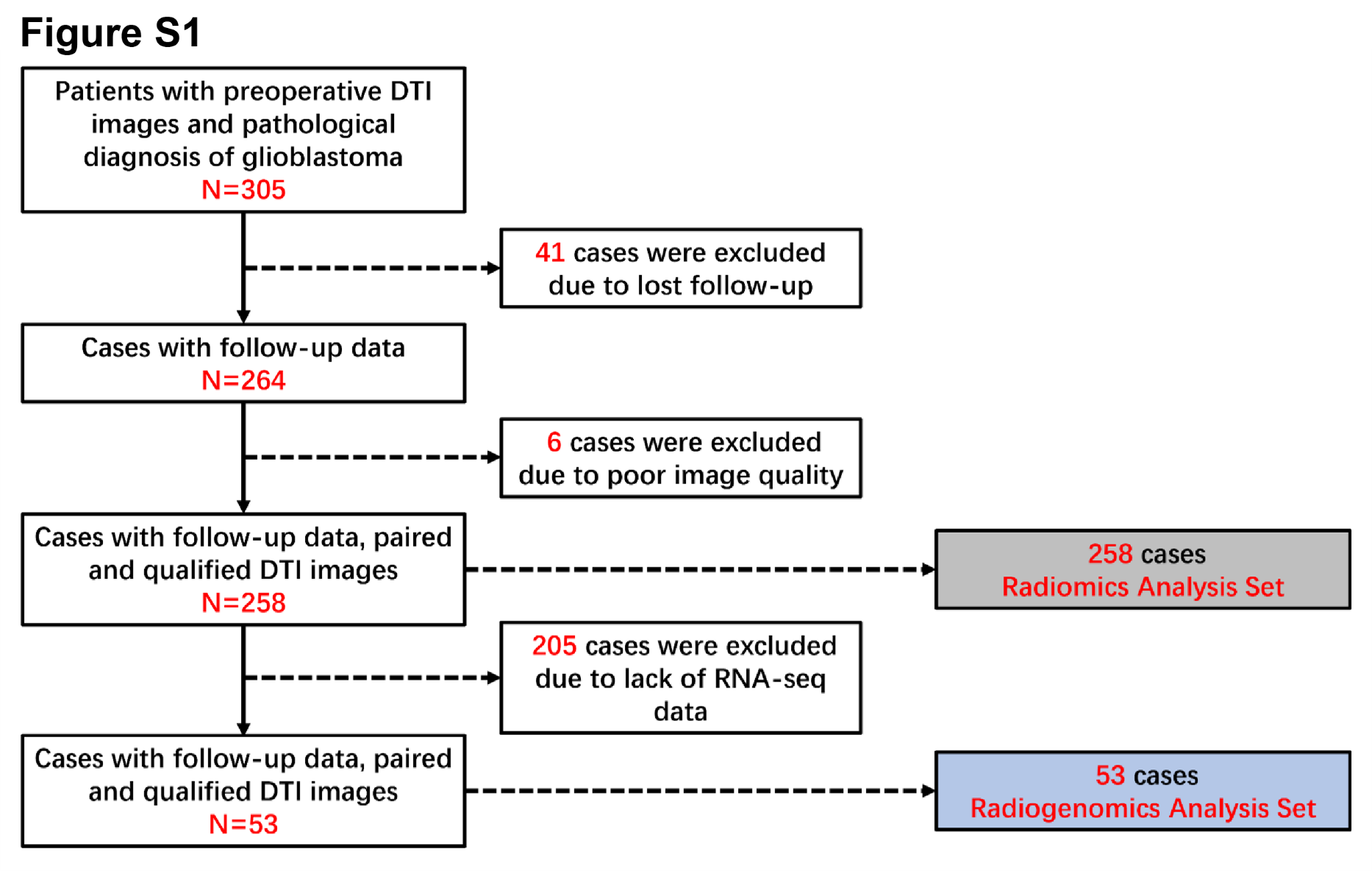


**Figure S2.** Forest plot of prognostic radiomic features

Forest plot showing name, DTI metrics, type, and univariable prognostic performance in terms of concordance index (C index) for each of 14 selected radiomics features.


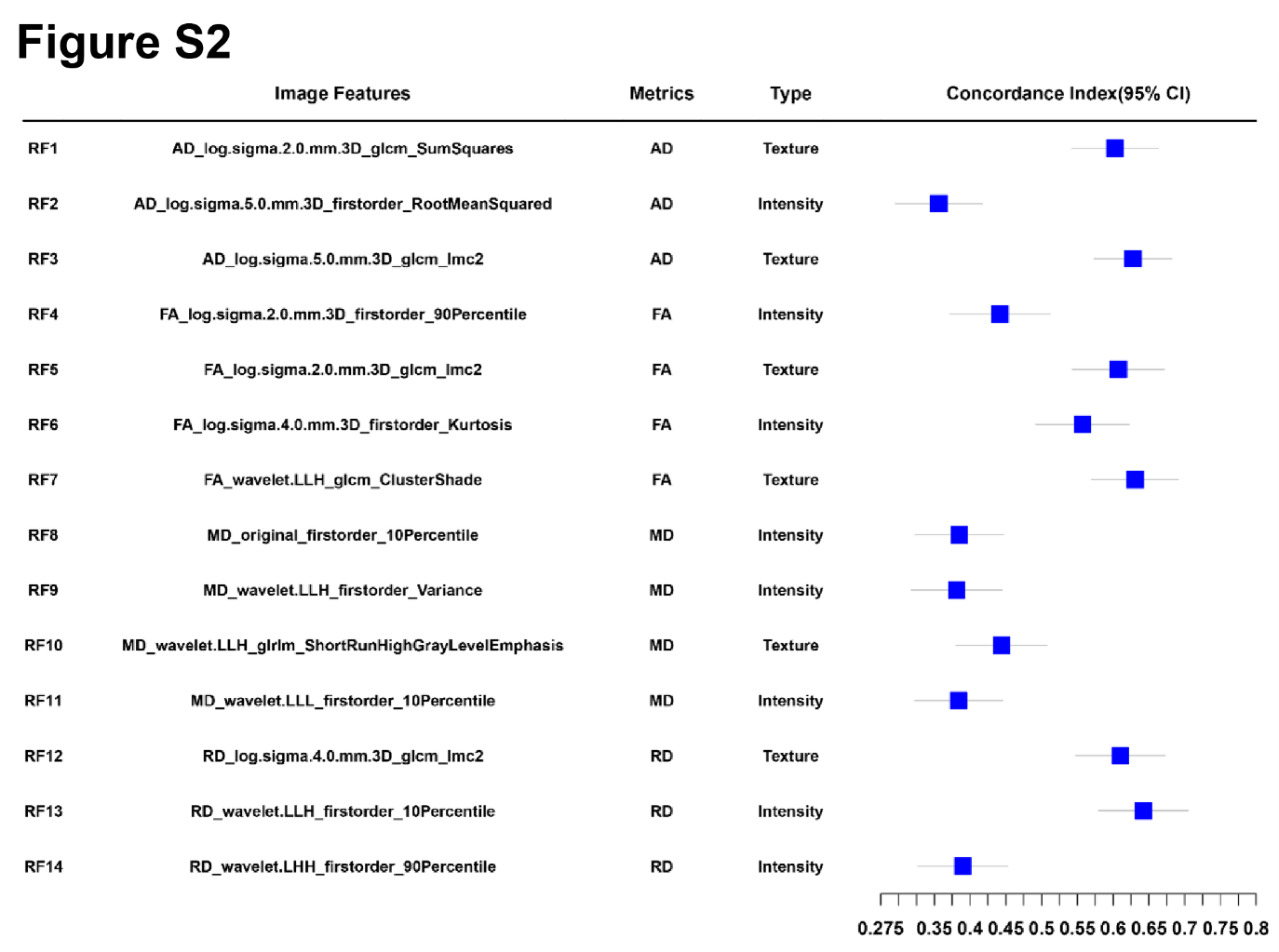


**Figure S3.** Radiomics feature selection 1

Radiomics feature selection by using the LASSO Cox model. Ten-fold cross validation is used to determine the optimal LASSO parameter λ. According to parameter λ, the coefficients of the most irrelevant features were shrunk to zero.


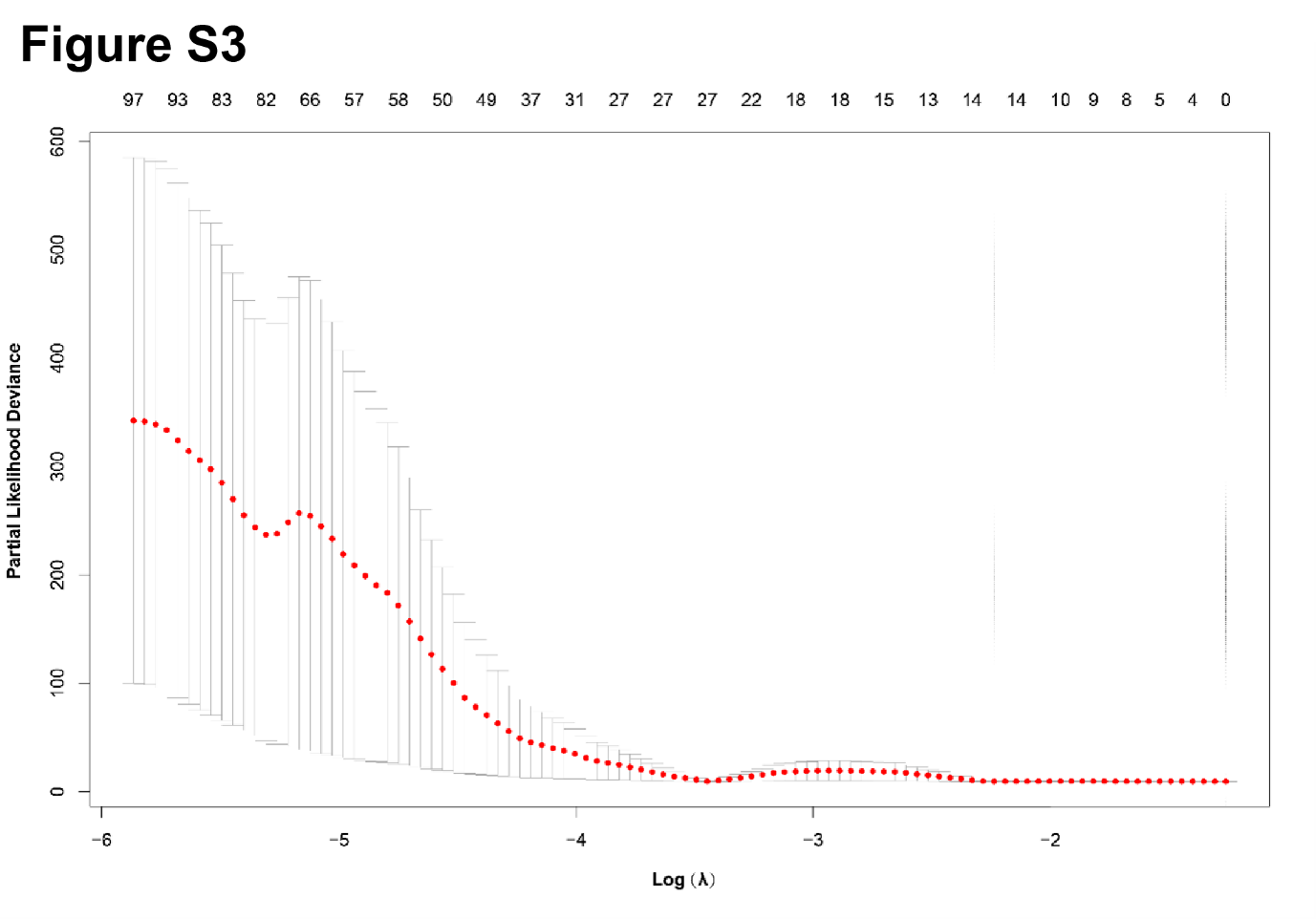


**Figure** S4. Radiomics feature selection 2

Radiomic feature selection by using the LASSO Cox model. The partial likelihood deviance was plotted versus log(λ). According to the dotted vertical lines, the optimal value λ of 0.1068 with log_e_(λ) = -2.236797 was chosen.


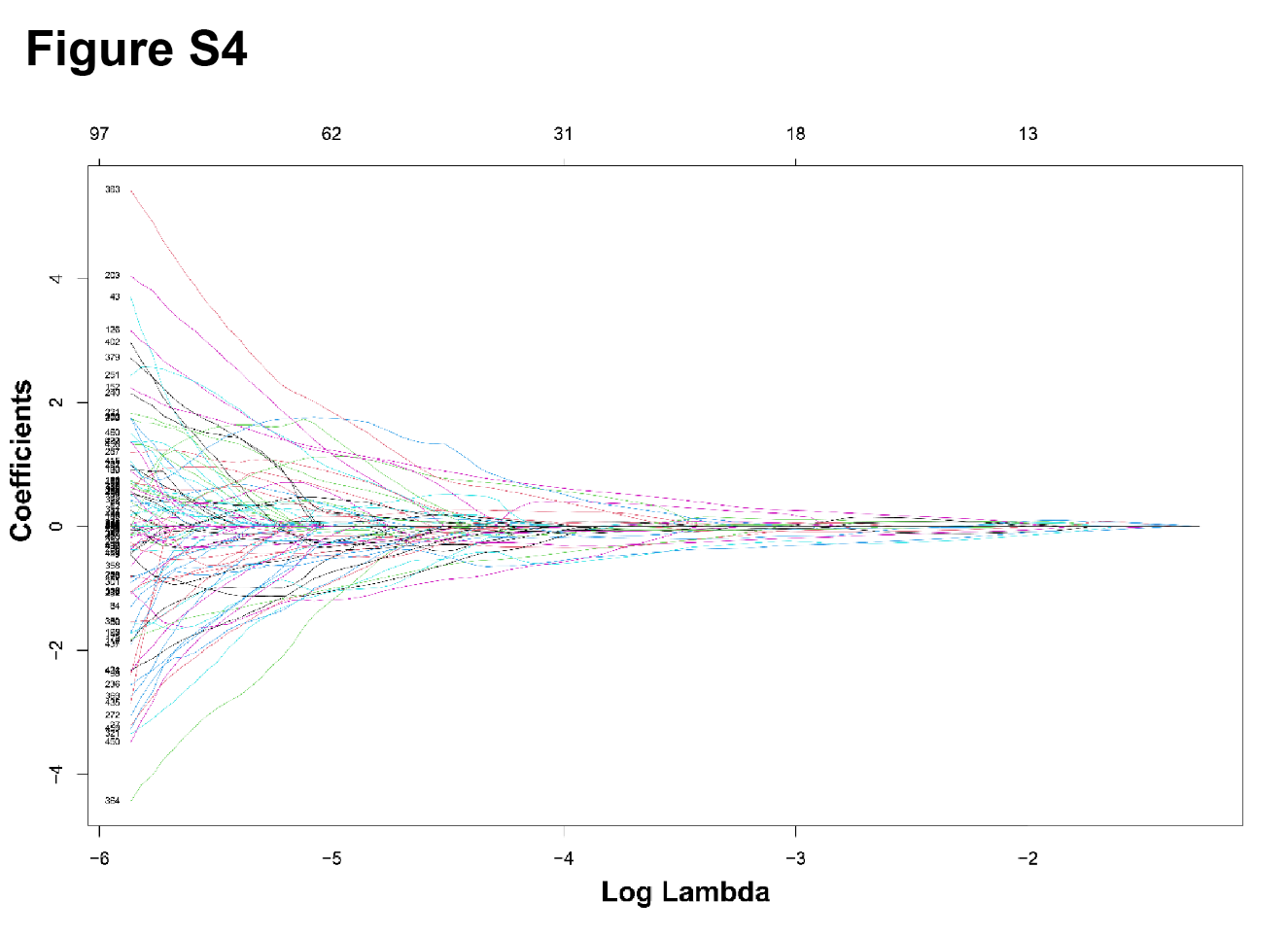


**Figure S5.** Incremental value of radiomic model

The differences between clinical model and radiomics-clinical model, including IDI, continuous NRI, and median improvement.


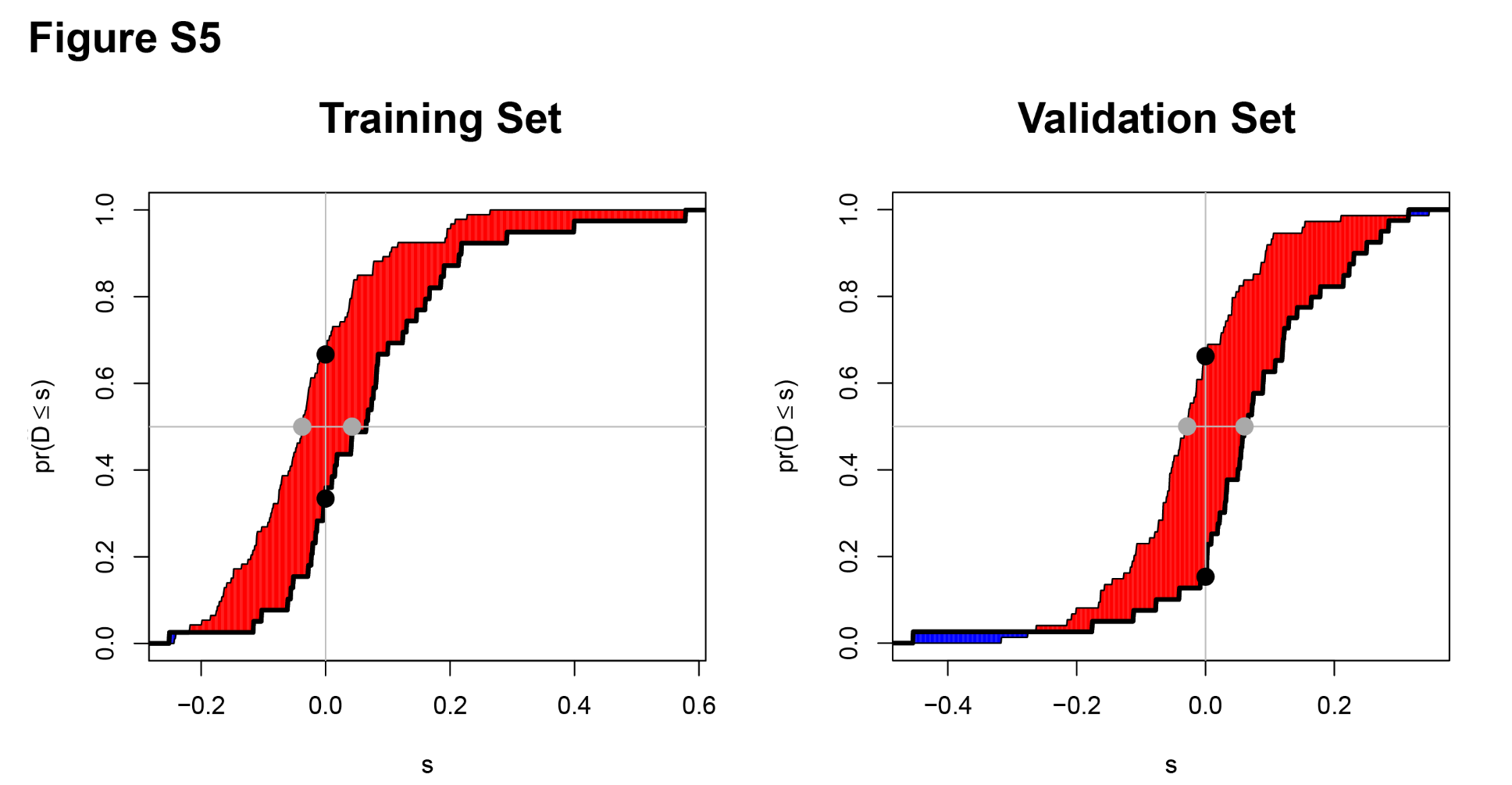


**Figure S6.** Radscore for each patient

Based on the cutoff value of -0.2679513 generated by R package survminer, patients were divided into a high-risk group (Radscore ≥ -0.2679513) and a low-risk group (Radscore ≤ -0.2679513). The status of dead or censorship was marked with different colors.


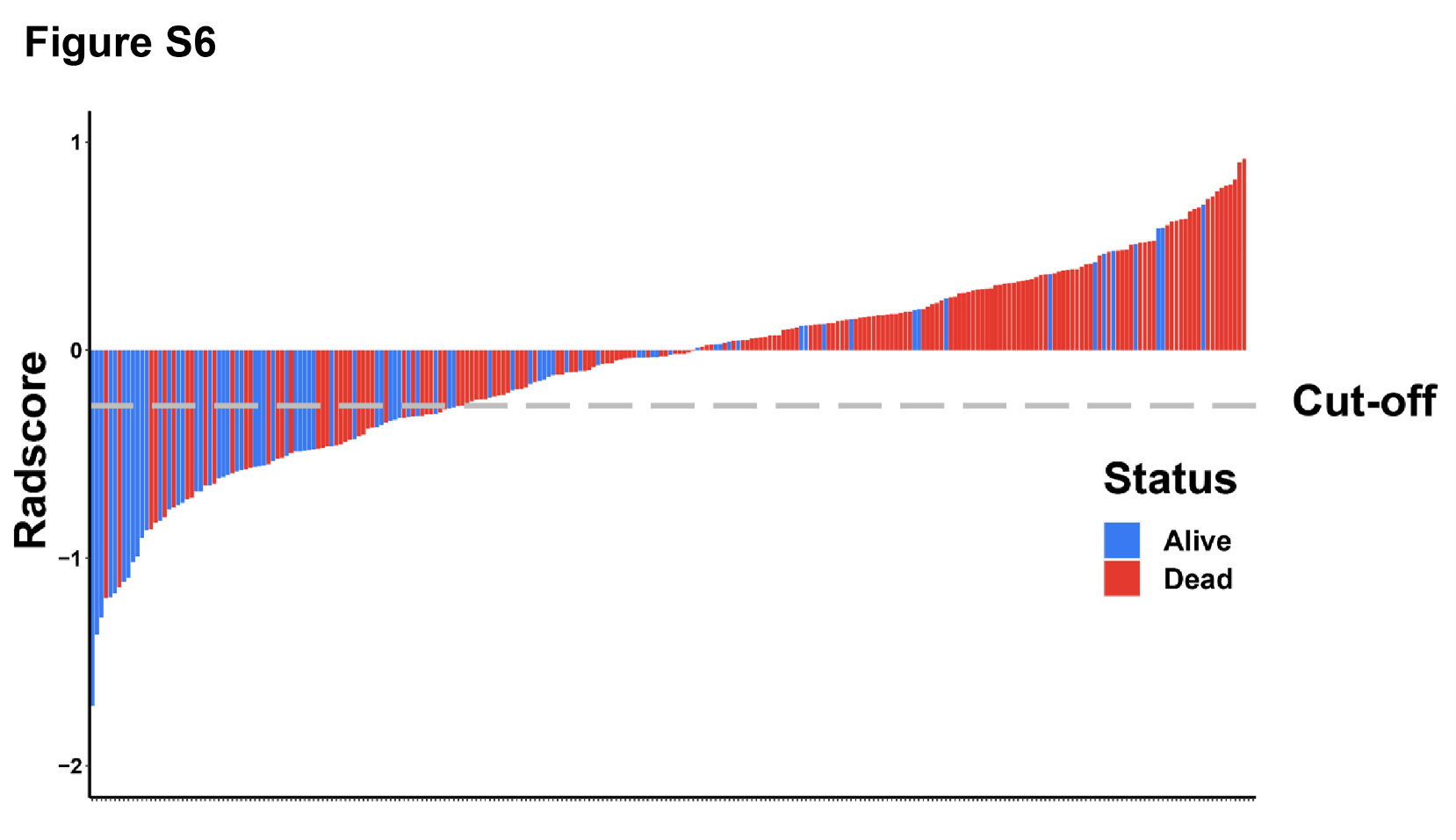


**Figure S7.** Construction of the radiomic model

(A) Kaplan-Meier curves for patients stratified by the radiomic signature in the training set.

(B) Decision curve analysis for radiomic-clinical model nomogram and clinical model nomogram to estimate the OS in the training set. The x-axis represents the threshold probability and the y-axis measures the net benefit.


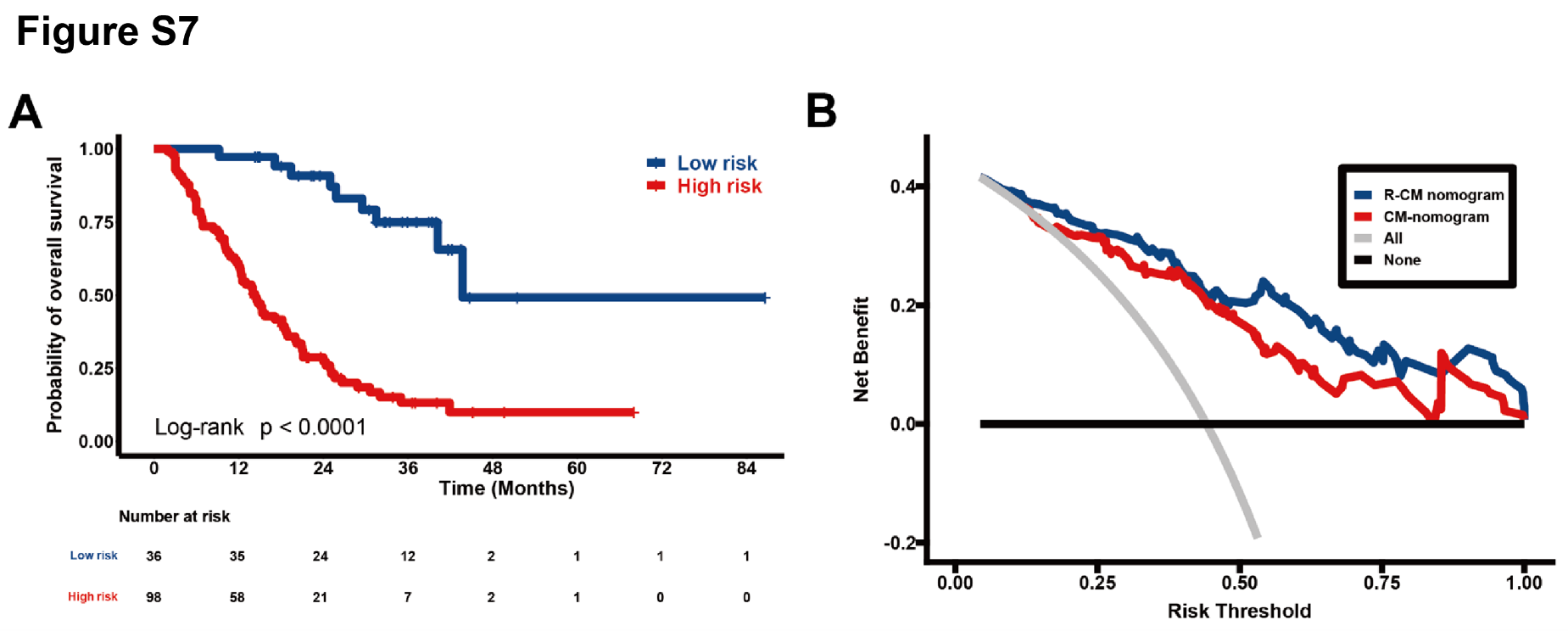


**Figure S8.** The Soft threshold selection process


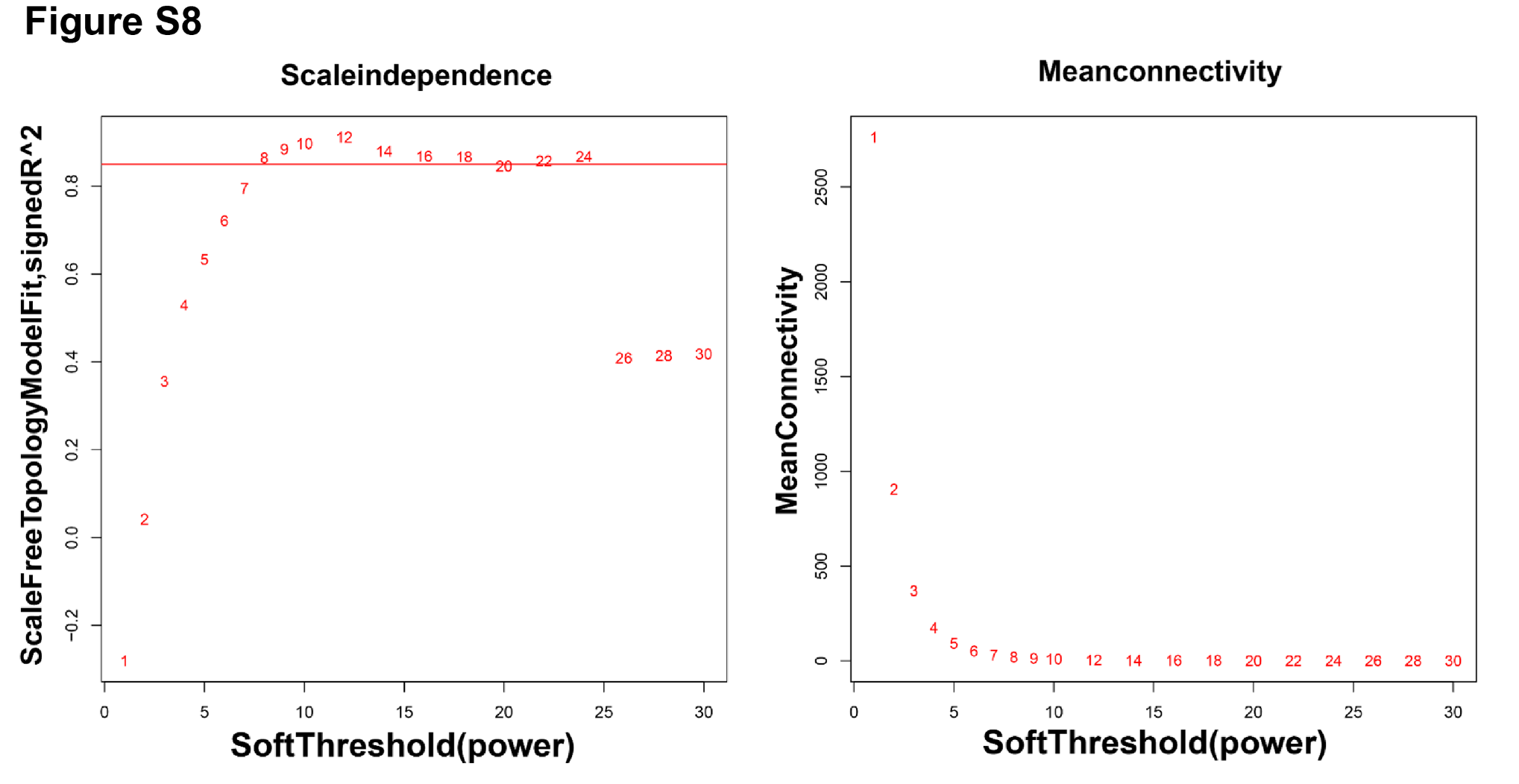


**Figure S9.** The relationship between the prognostic value and the number of pathways

The relationship between the prognostic value of a radiomics feature and the number of associated biological pathways.


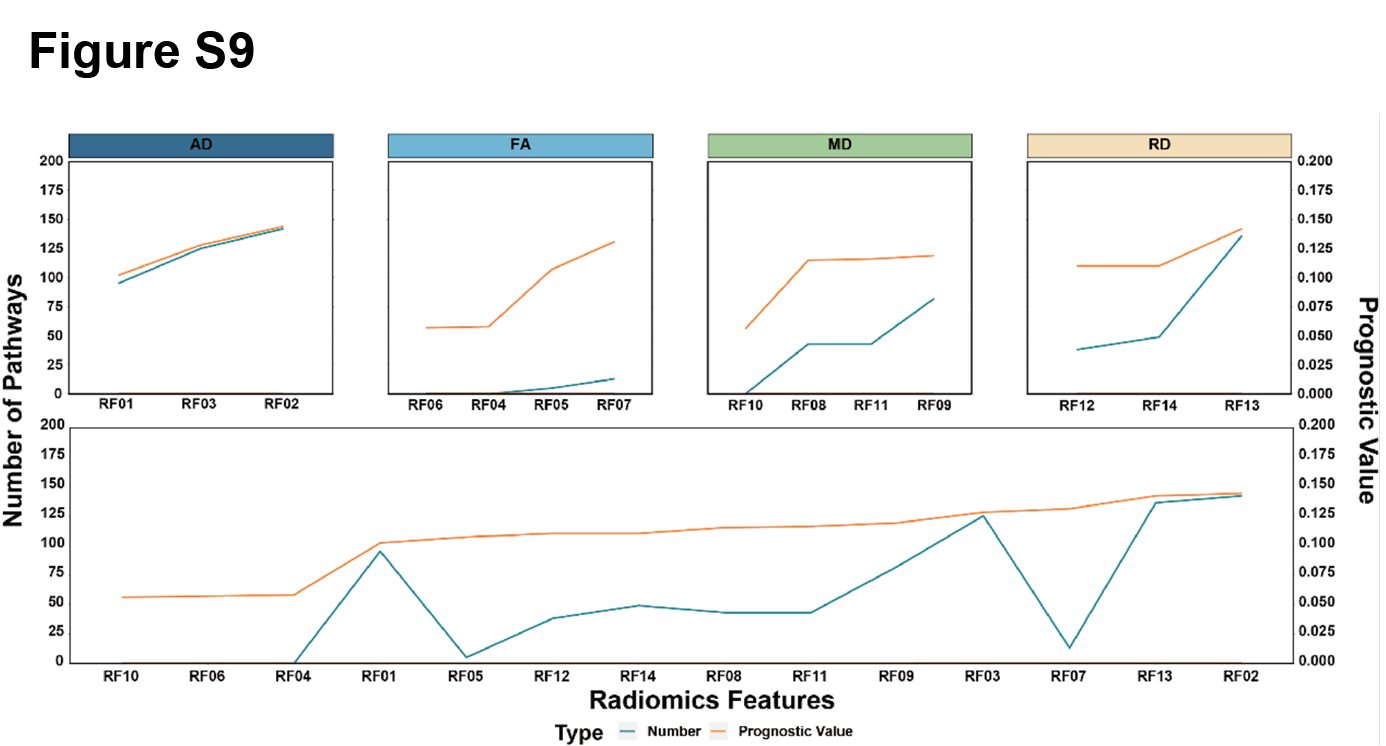

Supplement: Supplementary file 1 — Appendix S1: [file CNS-29-3339-s001.docx]
